# Protein folding, misfolding and aggregation: The importance of two-electron stabilizing interactions

**DOI:** 10.1371/journal.pone.0180905

**Published:** 2017-09-18

**Authors:** Andrzej Stanisław Cieplak

**Affiliations:** 1 Department of Chemistry, Bilkent University, Ankara, Turkey; 2 Department of Chemistry, Yale University, New Haven, Connecticut, United States of America; 3 Department of Chemistry, Brandeis University, Waltham, Massachusetts, United States of America; Russian Academy of Medical Sciences, RUSSIAN FEDERATION

## Abstract

Proteins associated with neurodegenerative diseases are highly pleiomorphic and may adopt an all-α-helical fold in one environment, assemble into all-β-sheet or collapse into a coil in another, and rapidly polymerize in yet another one via divergent aggregation pathways that yield broad diversity of aggregates’ morphology. A thorough understanding of this behaviour may be necessary to develop a treatment for Alzheimer’s and related disorders. Unfortunately, our present comprehension of folding and misfolding is limited for want of a physicochemical theory of protein secondary and tertiary structure. Here we demonstrate that electronic configuration and hyperconjugation of the peptide amide bonds ought to be taken into account to advance such a theory. To capture the effect of polarization of peptide linkages on conformational and H-bonding propensity of the polypeptide backbone, we introduce a function of shielding tensors of the C^α^ atoms. Carrying no information about side chain-side chain interactions, this function nonetheless identifies basic features of the secondary and tertiary structure, establishes sequence correlates of the metamorphic and pH-driven equilibria, relates binding affinities and folding rate constants to secondary structure preferences, and manifests common patterns of backbone density distribution in amyloidogenic regions of Alzheimer’s amyloid β and tau, Parkinson’s α-synuclein and prions. Based on those findings, a split-intein like mechanism of molecular recognition is proposed to underlie dimerization of Aβ, tau, αS and PrP^C^, and divergent pathways for subsequent association of dimers are outlined; a related mechanism is proposed to underlie formation of PrP^Sc^ fibrils. The model does account for: (i) structural features of paranuclei, off-pathway oligomers, non-fibrillar aggregates and fibrils; (ii) effects of incubation conditions, point mutations, isoform lengths, small-molecule assembly modulators and chirality of solid-liquid interface on the rate and morphology of aggregation; (iii) fibril-surface catalysis of secondary nucleation; and (iv) self-propagation of infectious strains of mammalian prions.

## Introduction

To understand protein folding, one needs to understand protein structure. And yet, in spite of the considerable interest and effort, even the most rudimentary issues of proteins conformational behaviour remain unresolved: ‘Surprisingly, the field lacks a physicochemical theory of protein secondary structure’ [1]. Indeed, for the chemist concerned to gain insight, protein study is in want of a theory which would explain what local backbone interactions make a residue a helix-breaker or a helix-former and how that propensity depends on the context—why a residue which is a helix-breaker in water becomes a helix-former in lipid or vacuum or why a residue which is a helix-former in the folding intermediate becomes a sheet-former in the native state. Pressing puzzles such as why certain sequences of helix-breakers and helix-formers may adopt an all-α fold in one environment, all-β fold in another, and collapse into a coil in yet another one, are bound to remain elusive unless these questions are answered.

The need to address the problem of secondary structure is underscored by the emerging evidence that ‘folding is an inherently digital process in which the formative interactions are among backbone elements^’^ [2], and that, given the folding and refolding of liquid lipase and gas-phase apomyoglobin, ‘the exigency of water in determining protein folding could possibly be overstated in current models used to describe this phenomenon.’ [3–6] To understand conformational behaviour of proteins, this evidence suggests, one ought to take into account the effects of main chain bonding—the backbone-backbone H-bonding but also the effects of hyperconjugative interactions of peptide linkages which depend on backbone conformation. The conceptual framework necessary for doing so is readily available in the modern physical organic chemistry [7]. The internal rotation in simple organic molecules was long argued to depend on the stereoelectronic as well as the steric and electrostatic effects [8,9]. The phenomena such as the gauche effect or the anomeric effect are now commonly attributed, at least in part, to hyperconjugation, and often satisfactorily described in terms of two-electron stabilizing interactions of the localized filled and vacant molecular orbitals [7–15]. Since the polypeptide chains comprise groups of orbitals whose through-bond and through-space interactions are altered by the internal rotation, similar effects may also play a role in the conformational equilibria of proteins. Hyperconjugative interactions of the peptide amide bonds have in fact been proposed by a number of authors to contribute to the stability of oligopeptides and proteins [16–22]. Thus, for the chemist to gain a sense of understanding, the theory of protein secondary structure may have to be rooted in the ‘qualitative orbital thinking’ [23,24] and the perturbational molecular orbital (PMO) theory which is uniquely equipped to support that way of thinking [7,25].

In this study, we attempt to construct such a theory. In a departure from the reigning paradigm, we take into account the variation in electronic configuration of the peptide bonds and the concomitant variation in the conformational and H-bonding propensity of the polypeptide backbone. The classical theory of protein structure was born half a century ago out of recognition of the steric and electrostatic constraints imposed by the peptide amide bonds on the internal rotation of the polypeptide chains [26,27]. The peptide linkages themselves, however, are in this theory assumed to be chemically equivalent and any differences in their geometry and bonding are disregarded. In contrast, we argue that the electronic configuration of the peptide amide bonds, and therefore the distribution of backbone density, does vary in the sequence and medium-dependent manner. This variation alters the balance between the backbone-backbone H-bonding and the polylactide-like backbone conjugation and thus underlies the diversity of conformational behaviour of the polypeptide chains.

The relationship between the electronic configuration of the peptide amide bonds and the torsional potential of the protein backbone was first considered in the context of modelling the relay of chiral information via the π-conjugated systems [16]. Subsequently, a theory was proposed linking the secondary structure propensities of the Ala congeners (the Ala* amino acids) to the inductive and resonance effects of the side chains and the stereoelectronic effects [28–31]; the sense and relative importance of these effects was assumed to depend on the location of the peptide amide bonds along the amide rehybridization/polarization path [32]. To explore the theory’s implications, a quantum-mechanical investigation of the single-site substitutions Ala→Lac (Lac≡L-lactic acid) was carried out [33].

We now return to the basic ideas espoused in these earlier studies, taking again advantage of the quantum-mechanical modelling of protein structure. However, we do not try to develop an *ab initio* or DFT simulation of folding—even if successful ‘such an approach would enable one to mimic nature but not necessarily understand her’ [34]. The PMO theory which underlies qualitative models of organic structure and reactivity, suggests the alternative approach: ‘The PMO treatment is concerned with differences in the properties of structurally related molecules, rather than the absolute values of the quantities relating to the individual molecules. Actually, by an appropriate choice of the reference system it will prove possible to obtain the information we require directly, in a very simple and straightforward manner which preserves a close understanding of the chemistry involved’ [35]. Following this protocol, we derive here a theory of encoding the 3D structure of proteins that attempts to address the origin of secondary structure and the mechanism of assembly of its elements into tertiary structure. This effort takes into account a wide range of phenomena such as divergent folding of highly homologous proteins and convergent folding of non-homologous proteins, metamorphic equilibria of lymphotactin, mitotic spindle protein Mad2 and *E*. *coli* virulence regulator RfaH, pH-driven transitions of viral fusion proteins and membrane translocation domains, acid-induced unfolding and fibrillization of transthyretin, synergistic folding of split inteins, and coupled folding and binding of molecular recognition features [36–39].

To be truly comprehensive, however, a theory of folding ought to address misfolding, and not just as a matter of the theory’s elegance. One ‘pressing puzzle’ that presently tests our understanding of protein structure and folding is conformational behaviour of highly pleiomorphic proteins such as Aβ and tau proteins believed to play key role in genesis of Alzheimer’s disease, α-synuclein (αS) associated with Parkinson’s disease, or prion proteins (PrP) playing central role in genesis of transmissible spongiform encephalopathies (TSEs). Polymerization of Aβ, tau, αS or PrP presents especially demanding challenge given the divergence of aggregation pathways, broad diversity of aggregates’ morphology, and apparent ease of proteins’ transitioning through the entire secondary and supersecondary structure manifold. Therefore, to probe the explanatory and predictive power of our theory, we apply its tenets to develop a model for nucleate polymerization of these proteins. The model aims to account for the structural features of oligomers, non-fibrillar aggregates and fibrils—such as annular and tubular aggregation of paranuclei or the symmetry of protofilament assembly, and for the effects of incubation conditions, isoform length, point mutations, assembly modulators, surface catalysis, lipid-raft composition etc. In particular, we are interested in the effects of chirality on the rate and morphology of Aβ fibril formation on the surfaces of *R*(*S*)-cysteine-modified graphene oxide and self-assembled monolayers of *R*(*S*)-N-isobutyrylcysteine on gold [40,41], the differences between Aβ aggregation on hydrophilic mica and hydrophobic graphite [42], and the mechanism of Aβ fibril-surface catalysis of secondary nucleation [43]. Ultimately, however, our goal is to develop structure-based insights into the complexity of brain proteinopathies.

## Computational methods

Our choice of the reference system for the PMO theory-informed analysis is a simplified structure of the polypeptide backbone which can be converted into a complete protein chain by ‘switching on’ three standard perturbations [44]: (1) the geometry perturbation (by changing conformation); (2) the atomic substitution/electronegativity perturbation (by plugging in the side chains); and (3) the intermolecular perturbation (by embedding backbone chain in polarizing or depolarizing environment and allowing for the interactions with co-solutes, other protein chains, surfaces of biopolyelectrolytes etc.). The simplified backbone structure comprises the (-C^α^-NH-C (=O)-)_*n*_ chain and the localized MOs of the peptide amide bonds and their ligands. It is assumed that the sterically-allowed regions of the ψ/φ space are approximately equivalent in terms of energy unless the stereoelectronic and electrostatic interactions are introduced [27,45]. The relative importance of those interactions—which ones actually occur—depends on electronic configuration of the peptide amide bonds which in turn depends on electronic effects of the side chains (atomic substitution/electronegativity perturbation) and on the mutual polarization of the protein and the medium (intermolecular perturbation). Given that each interaction of the peptide amide bonds is maximized in a specific region of the ψ/φ space (geometry perturbation), the amino acid sequence and the medium may determine conformational and H-bonding propensity of the polypeptide backbone by ‘switching on’ and ‘switching off’ certain combinations of those interactions. To assess the effects of these perturbations, we employ (i) *ab initio* and DFT studies of secondary structure (geometry optimizations; NBO analysis of the donor-acceptor interactions of the localized natural bond orbitals using Weinhold’s Δ*E*^(2)^ energies [12]—the BLW-ED energies [46] are lower but the qualitative trends are expected to be the same; SCRF modeling of solvent effects; GIAO calculations of the NMR shielding tensors **σ**(C^α^)^Xaa^ of C^α^ atoms), and (ii) qualitative concepts of two theories of solutions: the Onsager theory of solute-solvent polarization [47] and the Debye-Hückel theory of dilute solutions of strong electrolytes [48]. First, to assess the side chains’ effect on the distribution of backbone density, the NMR shielding tensors **σ**(C^α^)^Xaa^ of the C^α^ atoms were calculated using the models of helix and sheet structures in gas phase (the effect of the medium is treated as a separate perturbation). Thus, two oligopeptides, N-acetyl hexaglycyl N-methylamide AcGGGGGGNHMe and N-acetyl pentaglycyl amide AcGGGGGNH_2_, were initially optimized in the conformations corresponding to the hairpin with the type Ib reverse turn, and 3_10_-helix, respectively, at the B3LYP/D95** level of the theory. The protocol involved folding of the peptide chain into the starting conformer using the standard *φ*_i_ and *ψ*_i_ values and subsequently an unconstrained optimization. The searches for the minima were completed by the default convergence criteria of Gaussian 98, Revisions A.3, A.7, A11.2 [49]. The sixth residue of the hexapeptide hairpin and the second residue of the pentapeptide helix were then systematically varied to generate congener structures with the canonical and covalently modified residues. The side chains were set into the conformations *trans* and–*gauche* about the C^α^-C^β^ bond when needed, both in the neutral and ionized state when appropriate, and a number of structures were partially constrained as noted in S1 Table. Again, all the searches for the minima were completed by the default convergence criteria. The calculations yielded the total of 141 AcGGGGGXaaNHMe and AcGXaaGGGNH_2_ structures. Atomic coordinates of the obtained structures were used to compute the NMR shielding tensors using the B3LYP/D95** and GIAO (Gauge-Independent Atomic Orbital) methods (S1 Table). The mean values of the obtained shielding tensors of the C^α^ atoms were converted into the folding constants σ^Xaa^ as described in Results and Discussion. To assess the effect of geometry perturbation on backbone conjugation and backbone-backbone H-bonding by examination of Weinhold energies Δ*E*^(2)^ of the hyperconjugative interactions which differentiate the sterically-allowed regions of the Ramachandran map, the protocol described above was employed to optimize five models of secondary structure **1**–**5** (B3LYP/6-31G*): the decapeptide AcAAAAAAAAANH_2_ **1** in the 3_10_-helix conformation, the pentadecapeptide AcAAAAAAAAAAAAAANH_2_ **2** in the α-helix conformation, the hexapeptide AcAAAAANHMe **3** in the 2_7_-ribbon conformation, the ternary antiparallel complex of the tripeptide (AcAANHMe)_3_ **4**, and the ternary parallel complex of the tripeptide (AcAANHMe)_3_ **5**. In addition, the binary complexes of the oligopeptides (AcAAANHMe)_2_ **6a**-**6d** and (AcAAAAANHMe)_2_ **7a**-**7b**/**8a**-**8c** were obtained by unconstrained optimization (as described above, at the B3LYP/6-31G* level of the theory, completed by the default convergence criteria of Gaussian98). To assess the effect of a polar dielectric on the distribution of backbone density in a globular protein molecule, the model of TC5b: AcAAAAAAAAGGPAAGAPPPA-NH_2_ **9**, was obtained by the full unconstrained optimization (HF/3-21G, gas phase) of the peptide chain placed in the conformation defined by the φ/ψ angles reported for the NMR ensemble of TC5b [50]. The final gas-phase structure **9a** was re-optimized in water taken as the continuous dielectric (HF/3-21G, the Onsager model as implemented in the Gaussian suite with ε_0_ = 78.39 and the radius of the spherical solvent cavity a_0_ = 8.43 Å) until the default convergence criteria were fully met again to obtain the structure **9b**. The Cartesian atomic coordinates and the total energies of all the secondary and tertiary structure models (156 entries) are included in S1 Dataset.

## Results and discussion

### a. The PMO theory of the secondary and tertiary structure of proteins

#### (i) Electronic configuration of the peptide amide bonds and conformational and H-bonding propensity of the polypeptide backbone

**Fig 1:**
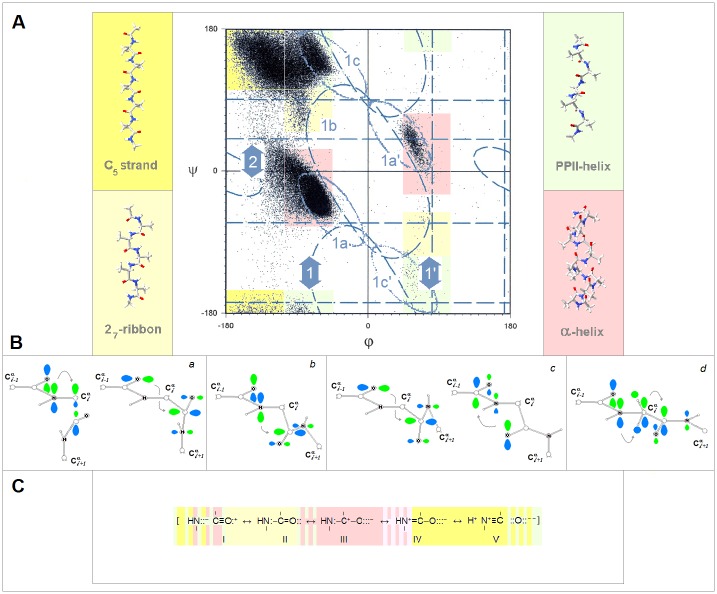
Electronic configuration of the peptide amide bonds and conformational and H-bonding propensity of the polypeptide backbone. **(A)** The dependence of interactions of the adjacent peptide amide bonds on location in the ψ/φ space of the polypeptide backbone (the diagram is adapted from the ref. [45]). The variation in φ_*i*_ changes mutual orientation of the peptide bonds: the bond planes are approximately perpendicular to each other in the helical region **1** (φ_*i*_ = −90°±30°) and approximately coplanar in the extended strand region **2** (φ_*i*_ = −150°±30°). The variation in ψ_*i*_ changes the extent of backbone H-bonding in the helical sub regions **1a**–**1c**. **(B)** Two-electron stabilizing interactions of the peptide amide bonds, depicted using the canonical amide MO’s: (a) the generalized anomeric effect π_2_(N_*i*_‒C’_*i-1*_ = O)→σ*(C^α^_*i*_‒C’_*i*_) which is maximized when the C^α^_*i*_‒C’_*i*_ bond, the best hyperconjugative σ acceptor at C^α^_*i*_, overlaps the N_*i*_ lp that is in the entire helical region **1** (φ_*i*_ = −90°±30°), and homohyperconjugation *n*(C’_*i-1*_ = O)→π_3_*(N_*i+1*_‒C’_*i*_ = O) maximized in the α-helix region **1a** (ψ_*i*_ = ‒30°±30°); (b) homohyperconjugation π_2_(N_*i*_‒C’_*i-1*_ = O)→π_3_*(N_*i+1*_‒C’_*i*_ = O), maximized in the 2_7_-ribbon (C_7eq_ and C_7ax_) region **1b** (ψ_*i*_ = 90°±30°); (c) homohyperconjugation *n*(C’_*i-1*_ = O)→π_3_*(N_*i+1*_‒C’_*i*_ = O) and *n*(C’_*i*_ = O)→π_3_*(N_*i*_‒C’_*i-1*_ = O), maximized in the PP_II_-helix region **1c** (ψ_*i*_ = 150°±30°); (d) the extended (double) hyperconjugation π_2_(N_*i*_‒C’_*i-1*_ = O)→π(C^α^_*i*_RR’)→π_3_*(N_*i+1*_‒C’_*i*_ = O) maximized in the C_5_ region **2** (φ_*i*_ = ‒150°±30°). **(C)** Modern resonance model of the amide bonding and the dependence of conformational and H-bonding propensity of the polypeptide backbone on electronic configuration of the peptide amide bonds, see the text section **a**.(i).

The question how the two-electron stabilizing interactions relate to stability of protein structure can be addressed by considering how backbone hyperconjugation and covalent contributions to backbone-backbone H-bonding vary in the sterically allowed regions of the ψ/φ space, cf. Fig 1A. To maximize secondary orbital overlap [10], backbone hyperconjugation involves primarily pairs of adjacent peptide amide bonds where the ‘upstream’ *i‒1/i* bond is a donor and the ‘downstream’ *i/i+1* bond is an acceptor; the change in their mutual orientation, the ‘geometry perturbation’, changes the nature and magnitude of the interaction. When the planes of these two bonds are approximately perpendicular, φ_*i*_ = −90°±30°, the fold ensures optimal orbital overlap for the generalized anomeric effect [51,52] and the covalent contributions to the N_*i*_*⋯*C_*i*_ = O and C_*i-1*_ = O⋯C_*i*_ = O interactions, see Fig 1B [17–20,33,53–58]; in contrast, orbital overlap for the covalent contributions to backbone-backbone H-bonding is relatively poor [59]. When these two peptide bonds are approximately coplanar, φ_*i*_ = −150°±30°, the situation is reversed. The generalized anomeric effect and homohyperconjugation are diminished due to vanishing overlap and the significance of the alternative hyperconjugative stabilization is limited [21] while the overlap for covalent contributions to backbone-backbone H-bonding is optimal [59]. The estimated energies Δ*E*^(2)^ of the corresponding donor-acceptor interactions in the secondary structure models **1**-**5**, cf. Computational Methods, are listed in S2 Appendix.

**Fig 2:**
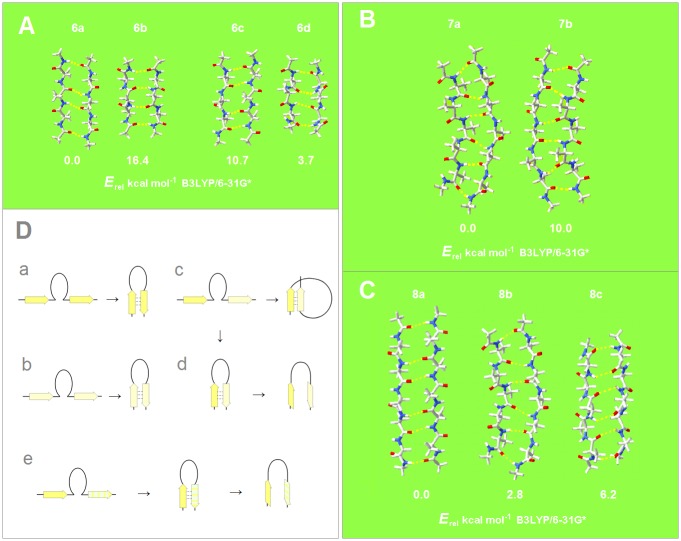
Conformational diversity in the binary complexes of extended oligopeptide strands. The geometries and energies obtained by quantum-mechanical modeling of the two-stranded β-sheets **6**–**8** (Computational Methods). The individual strands in these complexes optimize either to the C_5_ or the C_7eq_ (2_7_-ribbon) geometries, and their conformations are same in the antiparallel complexes (C_5_↑C_5_↓ or C_7eq_↑C_7eq_↓) and mixed in the parallel complexes (C_7eq_↑C_5_↑); *the antiparallel complexes with mixed strand conformations (C*_*7eq*_*↑C*_*5*_*↓) are unstable in unconstrained optimizations*. **(A)** The antiparallel complexes of the tetrapeptides (AcNH-Ala_3_-NH_2_)_2_ displaying the edge-to-edge topoisomerism: the assembly creates either two or one large H-bonded (HB) ring. **1a**: the C_5_↑C_5_↓ complex with two large HB rings; **1b**: the C_7eq_↑C_7eq_↓ complex with one large HB ring; **1c**: the C_5_↓C_5_↑ complex with one large HB ring; **1d**: the C_7eq_↓C_7eq_↑ complex with two large HB rings. **(B)** The parallel complexes of the hexapeptides (AcNH-Ala_5_-NH_2_)_2_ displaying the edge-to-edge topoisomerism: here all the H-bonded rings are equivalent but complex formation involves the edges with either two or three intrachain H-bonds. **2a**: the C_7eq_↓C_5_↓ complex involving the edges with two intrachain H-bonds; **2b**: the C_5_↓C_7eq_↓ complex involving the edges with three intrachain H-bonds. The large difference in the energy of the edge-to-edge topoisomers is not observed in the case of the binary complexes of the oligopeptides with the odd number of the peptide bonds. **(C)** The relative energies of the **3a**: C_5_↓C_5_↑, **3b**(≡**2a**): C_7eq_↓C_5_↓, and **3c**: C_7eq_↑C_7eq_↓ complexes of the hexapeptides (AcNH-Ala_5_-NH_2_)_2_. **(D)** The segments comprising two consecutive strands form stable β-hairpins (antiparallel assembly) when the two strands are either (a) both highly polarized (C_5_↑C_5_↓) or (b) both moderately polarized (C_7eq_↑C_7eq_↓) (color-coding as in Fig 1). In contrast, when one strand is highly polarized and the other is moderately polarized, these segments are expected to form (c) β-solenoid coils (parallel assembly, C_7eq_↑C_5_↑) or (d) unstable β-hairpins (antiparallel assembly C_7eq_↑C_5_↓) which are prone to convert into β-arches; similarly (e) when one strand is highly polarized and the other is least-polarized (the configuration described by a large contribution of the structure I, Fig 1C), the segment may form a hairpin (C_5_↑C_5_*↓) which is also prone to convert into β-arch.

It follows that the peptide bonds which are good N lp donors and poor H-bond acceptors should stabilize the φ_*i*_ = ‒90°±30° fold while the peptide bonds which are poor N lp donors and good H-bond acceptors should stabilize the φ_*i*_ = −150°±30° fold. To refine this picture we take into account computational and experimental evidence of wide variation in electronic configuration of the amide bonds in carboxamides, lactams, oligopeptides and proteins [7,32,60–63]. According to this evidence, one can describe bonding of peptide linkages in terms of varying contributions of the five resonance structures I-V, see Fig 1C. The shift I→II→III→IV→V has the following consequences for the ψ/φ space preferences:

(1) The structure I contributes to the configuration of the least-polarized bonds which display positive r_C = O_ vs. r_C-N_ correlations [32], are relatively poor acceptors of H-bonds, form largely ionic backbone-backbone H-bonds [64,65], and are good π/resonance N lp donors; it is compatible with the PP_II_ helix where backbone-backbone H-bonding is absent as well as the φ_*i*_ = 180°±30°/ψ_*i*_ = 180°±30° fold cf. Fig 1B(d) which is stabilized by the extended N lp hyperconjugation [21]. The least polarized backbone segment is therefore expected to exist as a statistical random coil unless molecular embedding turns it into an extended strand (the C_5_* strand) or a helix (PP_II_-helix or the α*-helix).

(2) The structure II contributes to the configuration of the moderately-polarized bonds which are still relatively poor acceptors of H-bonds but good N lp donors. This configuration is compatible with the 2_7_-ribbon (the C_7eq_ strand) since the φ_*i*_ = ‒90°±30°/ψ_*i*_ = 90°±30° fold is stabilized by the generalized anomeric effect as well as the homohyperconjugation, cf. Fig 1B(a) and 1B(b), and by the relatively effective backbone-backbone H-bonding. Thus, the moderately-polarized segments (largely II) can form β-sheets via assembly of the C_7eq_ strands as reported in Fig 2.

(3) The structure III contributes to the configuration of the polarized bonds which are still good N lp donors and already good H-bond acceptors while being good C’ σ and π acceptors as well. Thus, this configuration is unique in ensuring that the three major interactions which stabilize the α-helix are sufficiently strong at the same time (the general anomeric effect and homohyperconjugation, Fig 1B(a), and the backbone-backbone C = O⋯H−N bonding). The polarized segments (largely III) are therefore expected to form α-helices.

(4) The structures IV and V contribute to the configuration of the highly- and most-polarized bonds which display negative r_C = O_ vs. r_C-N_ correlations [32], are good acceptors and donors of H-bonds and form largely covalent H-bonds (including the backbone-backbone C = O⋯H−C bonding [66,67]), but are poor π donors in the hyperconjugative interactions of N. Thus, the highly-polarized backbone segments will stabilize the φ_*i*_ = ‒150°±30°/ψ_*i*_ = 150°±30° fold i.e. the C_5_ strands which readily assemble into β-sheets as reported in Fig 2. However, the most-polarized segments may also stabilize the PP_II_-helix and turn folds, *vide infra*, via the homohyperconjugation cf. Fig 1B(a) and 1B(c), and thereby destabilize the C_5_ strands.

This analysis implies that *the contribution of the energy of two-electron stabilizing interactions (ΔE*^*(2)*^*stabilization* [12]*) to ΔG*_*coil→helix*_ *has one minimum with respect to charge polarization of the polypeptide backbone while the contribution to ΔG*_*coil→sheet*_ *has two such minima*. Assuming that the Δ*E*^(2)^ contributions are significant, one expects Δ*G*_coil→helix_ and Δ*G*_coil→sheet_ to be quadratic and quartic functions, respectively, of backbone polarization.

According to the data for the models of β structure **6**-**8** in Fig 2, the preferred mode of assembly of the two-stranded β-sheets may also depend on charge polarization of the main chain. The antiperiplanar assembly should be stabilized when two strands are either both highly polarized, C_5_↑C_5_↓, or both moderately polarized, C_7eq_↑C_7eq_↓ (backbone-polarization ‘symmetry’); the parallel assembly should be stabilized when one strand is highly polarized and the other is moderately polarized, C_5_↓C_7eq_↓ (backbone-polarization ‘asymmetry’). This model implies that the segments comprising two consecutive strands form stable β-hairpins (antiparallel assembly) when the two strands are either both highly polarized (C_5_↑C_5_↓), Fig 2D(a), or both moderately polarized (C_7eq_↑C_7eq_↓), Fig 2D(b). In contrast, when one strand is highly polarized and the other is moderately polarized, these segments are expected to form β-solenoid coils (parallel assembly, C_5_↑C_7eq_↑), Fig 2D(c), or unstable β-hairpins (antiparallel assembly C_5_↑C_7eq_↓) which may convert into β-arches, Fig 2D(d). Lastly, when one strand is highly polarized and the other is least-polarized, the segment may form a hairpin (C_5_↑C_5_*↓, see Fig 1C) also prone to convert into β-arch, Fig 2D(e). Thus, in addition to β turns, β bulges, β arcs and small H-bonded rings [68], it is the number, spacing and sequence of the C_5_, C_5_*and C_7eq_ segments that may direct the polypeptide chains to fold into β structure of specific chirality and topology such as β meanders, up-and-down β barrels, Greek-key motifs and β-roll barrels, β sandwiches, β solenoids or β arcades.

This analysis also suggests that the contribution of the energy of two-electron stabilizing interactions to ΔG_coil→turn_ correlates with the *change* in charge polarization along the polypeptide backbone. The juxtaposition of the polarizing and depolarizing residues maximizes the C_*i-1*_ = O⋯C_*i*_ = O and C_*i*_ = O⋯C_*i-1*_ = O interactions (the homohyperconjugation), Fig 1B(a) and 1B(c). Consequently, a steep decrease in charge polarization along a short segment of the polypeptide backbone is expected to stabilize the elements of secondary structure where such interactions are likely to play a role: the 3_10_- and PP_II_-helices as well as β turns, α_R_α_L_ strands, classic β bulges, and β spirals i.e. collagen and elastin among others. The large change in backbone polarization can be achieved either by introduction of the least-polarized segment (with a large contribution of the structure I) or by introduction of the most polarized segment (with a large contribution of the structure V). Thus, β turns are expected to have two distinct electronic markers similarly to β strands.

#### (ii) Primary sequence and the intrinsic pattern of polarization of the peptide amide bonds: Folding potential *FP* of the polypeptide backbone

The intrinsic pattern of charge polarization of the polypeptide backbone of a given protein is determined by the primary sequence as a result of the steric, field/inductive, and resonance effects of the side chains operating in the immediate vicinity of the C^α^ atoms—the ‘atomic substitution/ electronegativity perturbation’. The NMR shielding tensors **σ**(C^α^)^Xaa^ of the C^α^ atoms, calculated using the hairpin with the type Ib reverse turn and the 3_10_-helix as the models (the L-amino acid series, 141 entries in S1 Table) at the B3LYP/D95** level of the theory (see the Computational Methods), are taken as a measure of the cumulative effect of these interactions on the distribution of backbone density. The folding constants σ^Xaa^, listed in Table 1, are derived from the linear normalization of the **σ**(C^α^)^Xaa^ tensor values to the scale where the σ^Pro^ constant for proline is ‒1 and the σ^Gly^ constant for glycine is 1. The amino acid residues can thus be said to be polarizing when σ^Xaa^<0 and depolarizing when σ^Xaa^>0; note that the polarizing effect depends on the ionization state of the side chains i.e. on the polarity and pH of the medium.

**Table 1:** Folding constants σ^Xaa^ of the canonical amino acids:^a^^,^^b^.

Xaa	σ^Xaa^	Xaa	σ^Xaa^
**A**	0.1898	**K**	−0.0772
**C**	−0.4989	**L**	−0.0441
**C[SMe]**	−0.0403	**M**	−0.2143
**D**	0.1293	**N**	0.0296
**D^-^**	−0.1087	**P**	−1
**E**	0.1889	**Q**	−0.2485
**E**^-^	−0.4847	**R**	0.1683
**F**	−0.4289	**S**	−0.4700
**G**	1	**T**	−0.9066
**H**	−0.2917	V	−0.7703
**H**^+^	0.2584	**W**	−0.2704
**I**	−0.7647	**Y**	−0.3981

^a^ Each tensor **σ**(C^α^) is the average of the values obtained with two models of secondary structure, a hairpin (AcGGGGXGNHMe/Ib) and a helix (AcGXGGGNH_2_/3_10_), at the GIAO//B3LYP/D95** level of the theory. The mean values for the *trans* and ‒*gauche* conformers of the side chain about the C^α^-C^β^ bond are taken when appropriate.

^b^ The σ^Xaa^ constants for some covalently modified amino acids are: Ser O^γ^-PO_3_^-2^ ‒1.1584, Met S (=O)_2_ ‒0.1101, Lys N^ξ^-COCH_3_ ‒0.2518, Val C^β^-CH_3_ ‒0.9993, Ala C^β^-F_3_ ‒0.4258, Phe -F_5_ ‒0.0829, Leu C^δ^-F_3_/C^δ^-F_3_ 0.0049, Ala C^β^-CF_3_ 0.2713, Ala C^β^-*n*-CH_2_CH_2_CH_3_ ‒0.2014, Thr C^β^-N^γ^H_2_ ‒0.8477, Gly C^α^-C≡N 0.8893, Gly C^α^-C≡NO 0.8500, Gly C^α^-C≡CH 0.6828.

σ^Xaa^ = {[**σ**(C^α^)^Xaa^(*trans*) + **σ**(C^α^)^Xaa^(‒*gauche*)] ‒ [**σ**(C^α^)^Gly^ + **σ**(C^α^)^Pro^]} / [**σ**(C^α^)^Gly^ ‒ **σ**(C^α^)^Pro^].

Using the folding constants σ^Xaa^, we quantify the relationship between the side chains’ effect and the conformational and H-bonding propensity of the polypeptide backbone by the magnitude and the slope of *the folding potential FP*. The folding potential at the residue *i*, *FP*_*i*_, is defined as the averaged sum of the mean μ_*i*_ and standard deviation σ_*i*_ of the constants σ^Xaa^ within the three-(*i*‒1, *i*, *i*+1) and five-(*i*‒2, *i*‒1, *i*, *i*+1, *i*+2)-residue windows, Eq. (1). The folding constants σ^Xaa^ are averaged over two windows of different width to account for the neighbouring residue effect, and the standard deviation terms are added to account for the effect of juxtaposing the polarizing and depolarizing residues (the weight of the μ_*i*_ and σ_*i*_ terms is arbitrary at this point): *FP*_*i*_ = ½[μ_*i*_(*σ*^Xaa^_*j*_; *j = i*‒1, *i*, *i*+1)+σ_*i*_(*σ*^Xaa^_*j*_; *j = i*‒1, *i*, *i*+1)+μ_*i*_(*σ*^Xaa^_*j*_; *j = i*‒2, *i*‒1, *i*, *i*+1, *i*+2)+σ_*i*_(*σ*^Xaa^_*j*_; *j = i*‒2, *i*‒1, *i*, *i*+1, *i*+2)] (1).

The slope of the folding potential at the residue *i*, Δ*FP*_*i-1→i+1*_, is approximated by the difference of the folding potential at the residues *i−1* and *i+1*: Δ*FP*_*i-1→i+1*_ = *FP*_*i+1*_ –*FP*_*i-1*_ (2). This definitions imply that proteins may tolerate the ‘inverted’ sequences [69].

**Fig 3:**
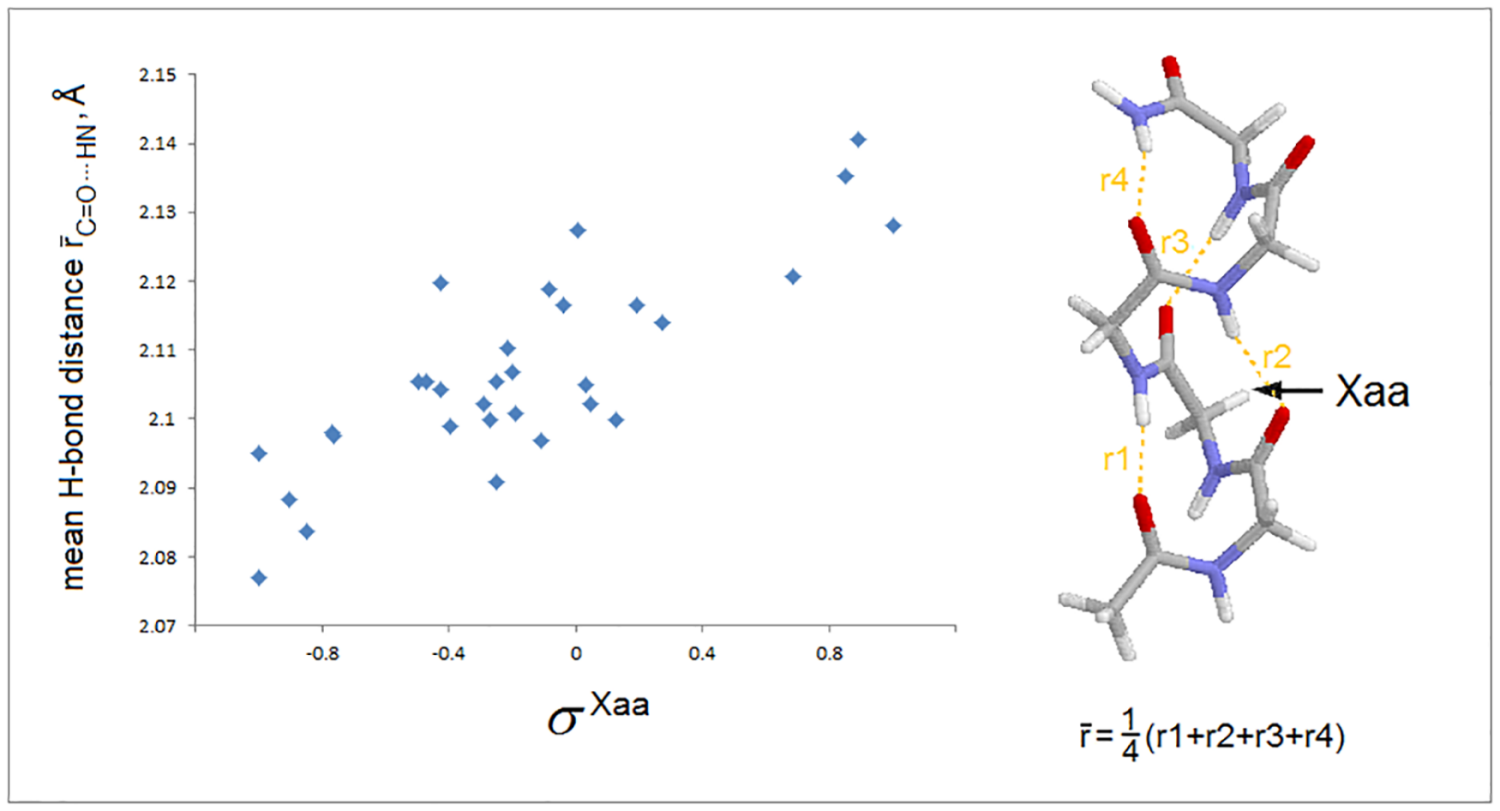
Folding constants σ^Xaa^ and the energy of backbone-backbone H-bonding. The calculated average backbone-backbone H-bond distance in the 3_10_-helices AcNHG(Xaa)GGGNH_2_ (shown in the right hand panel, calculated at the B3LYP/D95** level of the theory, cf. Computational Methods) vs. the folding constants σ^Xaa^ for all except the ionized Xaa residues listed in Table 1.

As shown in Fig 3, the folding constants σ^Xaa^ account for a significant fraction of variation in the averaged distances of backbone-backbone C = O⋯H−N bonds in the AcNHG(Xaa)GGGNH_2_ 3_10_-helices (calculated at the B3LYP/D95** level of the theory, cf. Computational Methods). The correlation confirms that the σ^Xaa^ constants, and hence the folding potential *FP*_*i*_, provide a measure of the intrinsic pattern of backbone polarization of a given protein.

**Fig 4:**
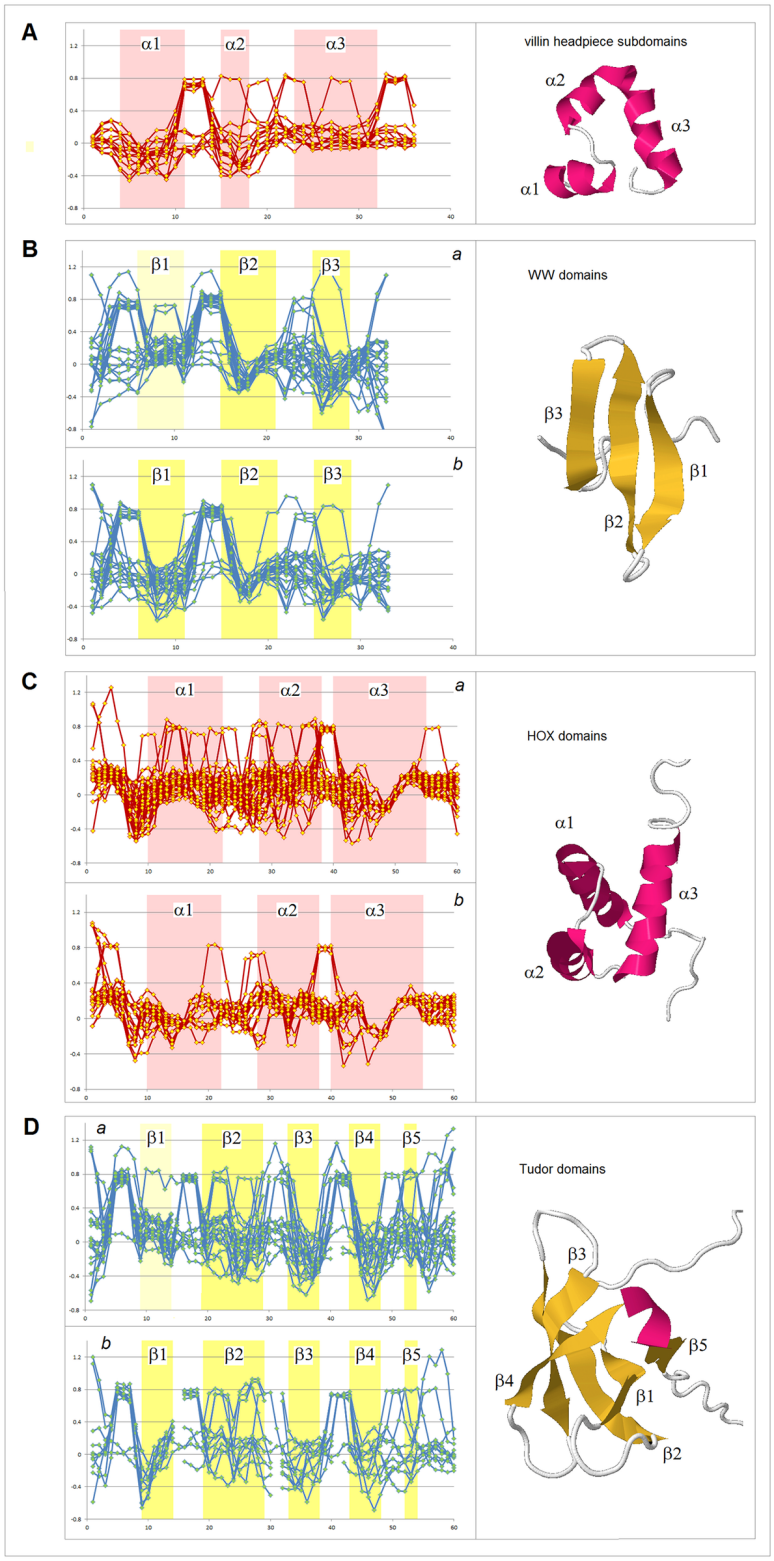
*FP*_*i*_ plots for small all-α and all-β soluble proteins. The folding potential at the residue *i* (*FP*_*i*_, calculated from Eq. (1)), is plotted (Y-axis) against the residue number *i* (X-axis). The multiple alignments are taken from the SMART database (smart.embl-heidelberg.de) and the reference below. Note the characteristic *FP*_*i*_ profiles of the secondary structure elements and the variation in average *FP*_*i*_ values of those elements, $\bar{{\text{FP}}_{\text{i}}}$(α) or $\bar{{\text{FP}}_{\text{i}}}$ (β): **(A)** VHP (villin headpiece) domain, accession # SM00153; **(B)** WW domain, accession # SM00456: (a) $\bar{{\text{FP}}_{\text{i}}}$ (β1) > 0; (b) $\bar{{\text{FP}}_{\text{i}}}$ (β1) < 0; **(C)** HOX (homeobox) domain: (a) $\bar{{\text{FP}}_{\text{i}}}$ (α1) > 0; (b) $\bar{{\text{FP}}_{\text{i}}}$ (α1) < 0 [70]; **(D)** Tudor domain, accession # SM00333: (a) $\bar{{\text{FP}}_{\text{i}}}$ (β1) > 0; (b) $\bar{{\text{FP}}_{\text{i}}}$ (β1) < 0. The helical and extended segments of the protein chain are shaded red, and yellow or light yellow, respectively.

The examples of the *FP*_*i*_ plots for the small soluble domains in Fig 4 suggest that the sequences which support spontaneous formation of the archetypal elements of secondary structure are marked by the specific values of the folding potential and the specific patterns of its slope. For instance, for the solvent-exposed α-helices, the Δ*G*_coil→helix_ minimum seems to occur in the range 0< *FP*_*i*_<0.3.

**Fig 5:**
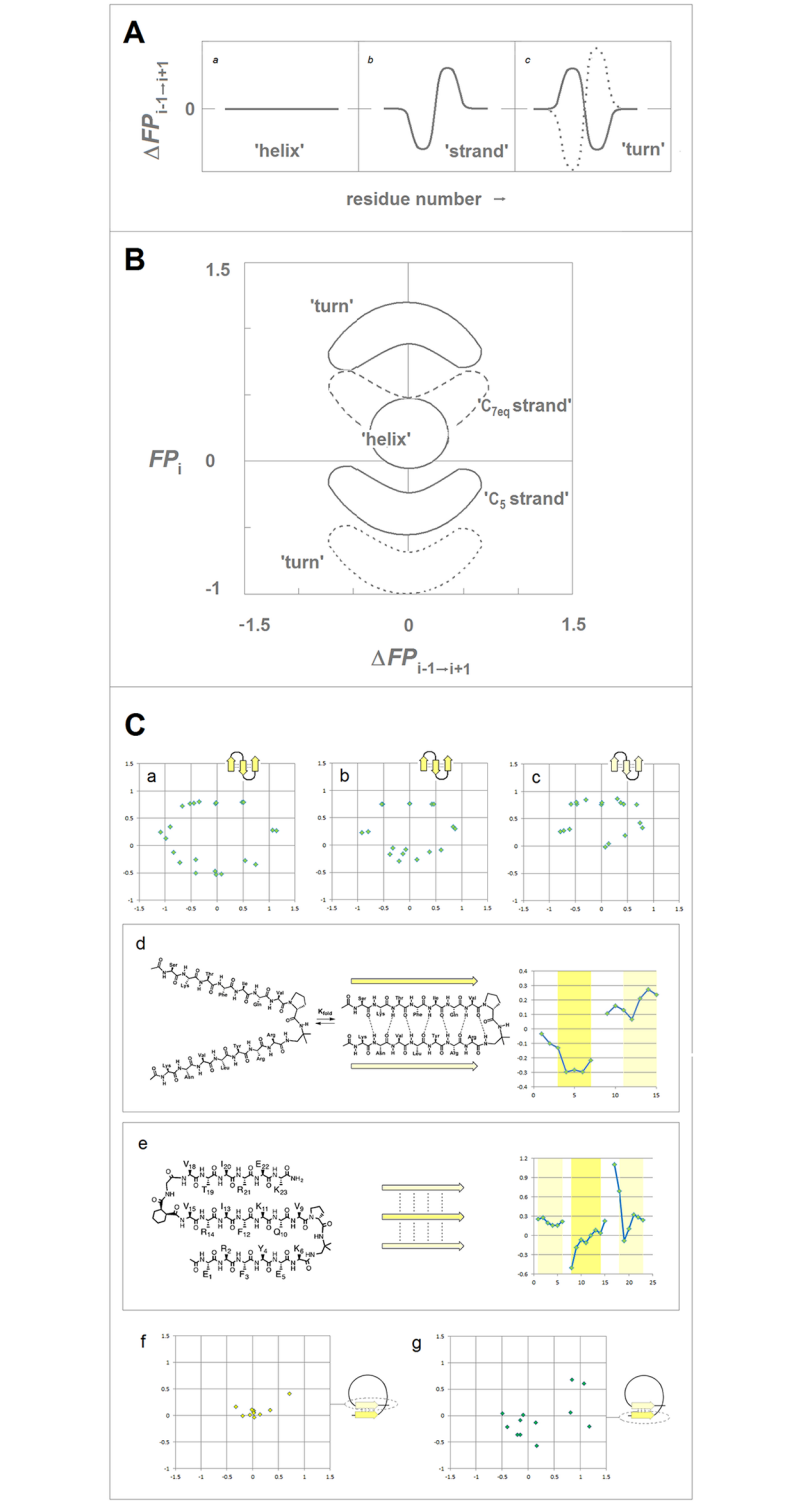
*FP*_*i*_ as a probe of the three-dimensional structure of proteins. **(A)** The patterns in the plots of Δ*FP*_*i-1→i+1*_ (Eq. 2) vs. the residue number, characteristic of the archetypal ‘helix’, ‘strand’ and ‘turn’. **(B)** Characteristic clusters of the data sets in the plots of *FP*_*i*_ vs. the ‘slope’ of *FP*_*i*_, Δ*FP*_*i-1→i+1*_, which correspond to the three archetypal elements of the secondary structure: e.g. the presence of the archetypal ‘helix’ will be marked by a compact cluster of data sets in the center of the plot. The ordinate of this cluster will vary since the optimal *FP*_*i*_ value for ‘helix’ depends on the medium’s capacity to polarize the protein, *vide infra*. Note that ‘strand’ and ‘turn’ have each two avatars: (i) ‘C_5_ strand’ and ‘C_7eq_ strand’, and (ii) ‘ *FP*_*i*_>>0 turn’ (defined here as the three- or five-residue segment that incorporates Gly in the centre) and ‘ *FP*_*i*_<<0 turn’. **(C)** The presence of the archetypal antiparallel ‘sheet’ would be marked by a circular distribution of data sets that combines the ‘C_5_ strand’/‘turn’ or ‘C_7eq_ strand’/‘turn’ clusters while the presence of the parallel ‘sheet’ would be marked by a combination of the ‘C_5_ strand’ and ‘C_7eq_ strand’ clusters, cf. Fig 2. This is illustrated by examples of *de novo* designed three-stranded antiparallel β-sheets (three-stranded β meanders), two- and three-stranded parallel β-sheets, and two-stranded parallel β-sheets embedded in left-handed coils from the C-terminal domains of the penicillin binding protein PBP2x from *Streptococcus pneumoniae*, PDB ID 1k25: (a) KGEWTFVNGKYTVSINGKKITVSI, ~50% in β structure, H_2_O, pH 3, 25°C (C_5_↑C_5_↓C_5_↑-meander) [71]; (b) TWIQNGSTKWYQNGSTKIYT, 20–30% in β structure, H_2_O, pH 3.25, 10°C (C_5_↑C_5_↓C_5_↑-meander) [72]; (c) RGWSLQNGKYTLNGKTMEGR, ~35% in β structure, 10%D_2_O/H_2_O or D_2_O, pH 5, 0–10°C (C_7eq_↑C_7eq_↓C_7eq_↑-meander) [73]; (d) C_5_↑C_7eq_↑-parallel sheet, cf. the *FP*_*i*_ plot. The C-termini of two strands are connected by the D-prolyl-1,1-dimethyl-1,2-diaminoethane unit (diamine linker D-Pro-DADME), ~64% ‘folding-core’ residues (F5-V8 and R11-L14) in β structure at 10°C, 10%D_2_O/H_2_O, 100 mM sodium acetate buffer, pH 3.8 [74]; (e) C_7eq_↑C_5_↑C_7eq_↑-parallel sheet, cf. the *FP*_*i*_ plot. The C-termini of strands 1 and 2 are connected by the diamine D-Pro-DADME while the N-termini of strands 2 and 3 are connected by the diacid formed from (1*R*,2*S*)-cyclohexanedicarboxylic acid (CHDA) and Gly, 4°C, 10%D_2_O/H_2_O, 2.5 mM sodium [D_3_]acetate buffer, pH 3.8 [75]; (f) the C_7eq_ strands from two C_5_↑C_7eq_↑-parallel sheets in the left-handed coils of PBP2x from *Streptococcus pneumoniae*, PDB ID 1k25; (g) the C_5_ strands from two C_5_↑C_7eq_↑-parallel sheets in the left-handed coils of PBP2x, PDB ID 1k25.

The expected Δ*FP*_*i-1→i+1*_ patterns for the archetypal ‘helix’, ‘strand’ and ‘turn’, and the corresponding *FP*_*i*_ vs. Δ*FP*_*i-1→i+1*_ plots are shown in Fig 5, along with the illustrative examples of such plots obtained for the autonomously folding models of β structure [71–75]. As discussed earlier, the archetypal ‘strand’ and ‘turn’ elements have each two avatars: (i) the ‘C_5_ strand’ (highly-polarized segment, cf. structure IV in Fig 1) and the ‘C_7eq_ strand’ (moderately-polarized segment, cf. structure II in Fig 1, see also Fig 2), and (ii) the ‘ *FP*_*i*_>>0 turn’ (least-polarized segment, cf. structure I in Fig 1) and the ‘ *FP*_*i*_<<0 turn’ (most-polarized segment, cf. structure V in Fig 1). Since the optimal *FP*_*i*_ values for each secondary structure element depend on the medium’s capacity to polarize the protein, *vide infra*, the ordinates of the characteristic clusters in Fig 5 will change with the environment, including microenvironment of molecular embedding.

#### (iii) Polarization of the polypeptide backbone by the medium: Folding basin’s gradient of permittivity and organization of tertiary structure

**Fig 6:**
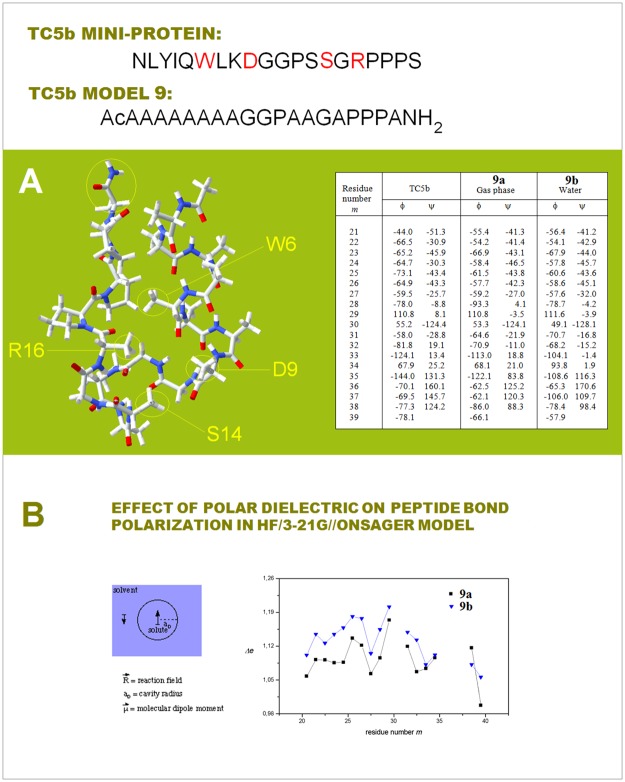
Effect of a polar dielectric on peptide bond polarization in a model of TC5b mini-protein. The structure of the simplified model of TC5b, **9**: AcAAAAAAAAGGPAAGAPPPA-NH_2_, obtained by full unconstrained optimization (HF/3-21G, gas phase) of the peptide chain placed in the conformation defined by the φ and ψ angles reported for the NMR ensemble of TC5b; the final structure was re-optimized in water taken as a continuous dielectric (the Onsager model as implemented in the Gaussian suite, cf. Computational Methods), until the default convergence criteria were fully met again. The backbone torsion angles of the TC5b NMR structure PDB ID 1l2y [50] and the *ab initio* structures **9a** (in gas phase) and **9b** (in a polar dielectric) are compared in the table on the right-hand side of the panel. **(B)** Dependence of charge polarization of the secondary peptide bonds Δ*e* (the difference (au) in H and O Mulliken populations of the *m* peptide bond [33]) on *m*—that is on bond location along the polypeptide chain in the models **9a** and **9b** of the mini-protein TC5b. The immersion of the TC5b model in a polar solvent results in the increase in Δ*e* along the entire chain i.e. in a considerable increase in charge polarization of the polypeptide backbone.

In addition to the side chains’ impact, electronic configuration of the polypeptide backbone is affected by the mutual polarization of the protein and its environment—the ‘intermolecular perturbation’ [47]. The mutual polarization with a continuous dielectric engages peptide bond dipoles as well as molecular electric moments e.g. the helix macrodipole. The interaction results, among others, in the change in the free energy barrier to internal rotation about the amide C-N bond; this change was shown to correlate with the dielectric constant function (ε−1)/(2ε+1) [76]. This correlation implies that the helix or cross-β arrays of H-bonded peptide linkages become more polarized upon the transfer from a non-polar to a polar medium, and our *ab initio* study of the model of TC5b mini-protein [50] supports this conclusion, see Fig 6 and the Computational Methods.

Thus, the polarization of those H-bonded networks is expected to increase with the increase in relative permittivity of the surrounding medium in the order: gas phase, lipid matrix of the phospholipid bilayer, the interior of a globular protein or DNA duplex [77,78], the interface of a phospholipid bilayer, a micellar interface, nematic phase of unspun silk, ‘Teflon-coating’ of the polypeptide chain in dilute water/(TFE or HFIP) solutions [79], the ~4 M in KCl cytosol of extreme halophilic *Archea* [80], and the cytosol or blood serum under the standard physiological conditions. The screening effect of the medium on the coulombic contribution to backbone-backbone H-bonding may be important in the case of the least-polarized backbone segments that are akin to the low-MW secondary amides in terms of electronic structure. Both experimental and computational evidence suggest that the enthalpy of H-bonding between such amides goes nearly to zero in water [64,65]. However, covalent contribution to H-bonding cannot be neglected even in the case of the water dimer in liquid water [81]. It seems reasonable to expect that the screening effect is negligible in the case of largely covalent backbone-backbone H-bonding of the polarized peptide bonds [12].

**Fig 7:**
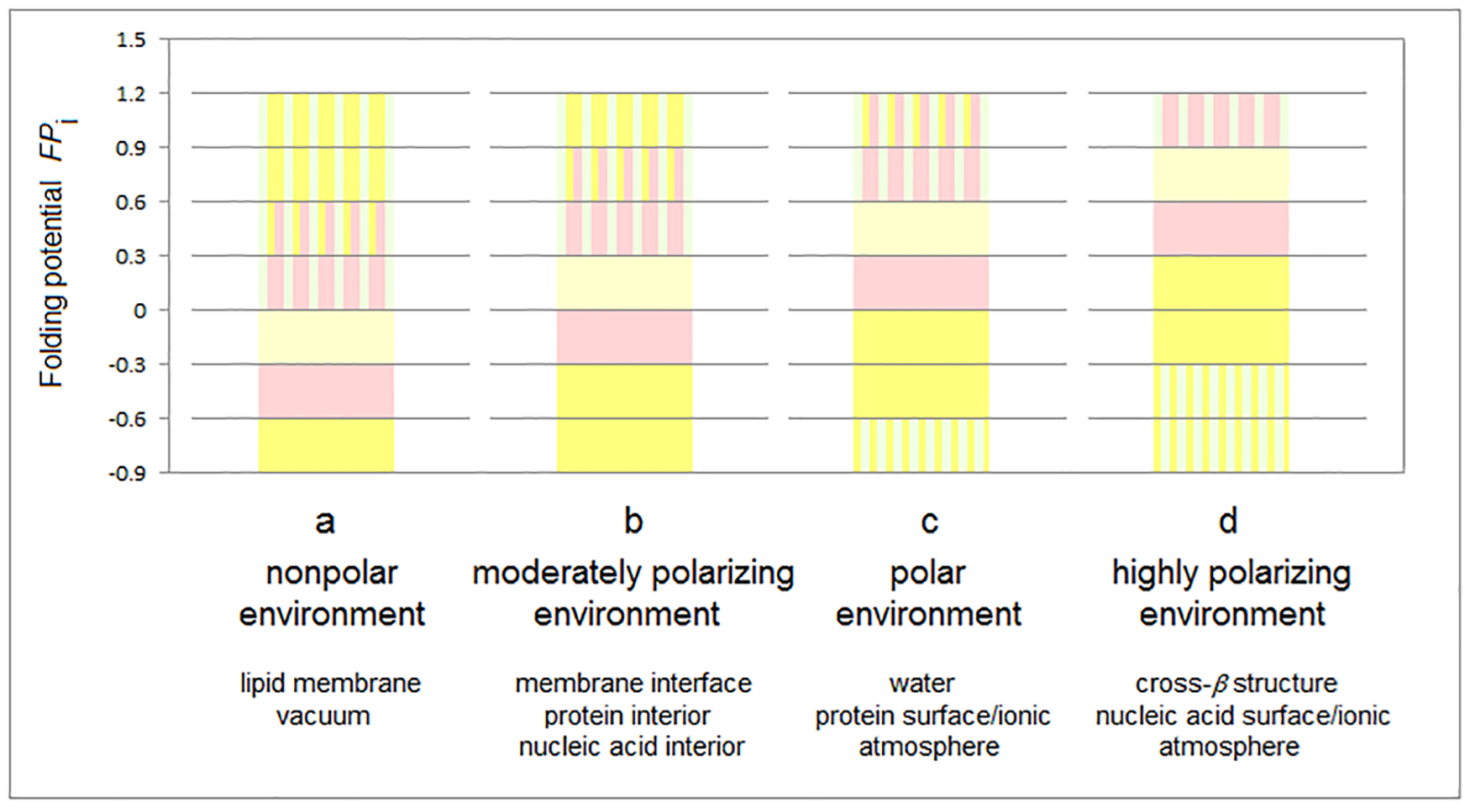
Folding potential, medium properties and secondary structure preferences of the polypeptide backbone. **(a)** The *FP*_*i*_ values that ensure stability of the periodic secondary structure in a non-polar environment such as the lipid matrix of the bilayer membrane or vacuum: the optimal *FP*_*i*_ range for the α-helix’ is ‒0.6-‒0.3 (color-coding as in Fig 1) and the optimal *FP*_*i*_ ranges for β structure is <‒0.6 (C_5_ strand) and ‒0.3–0 (C_7eq_ strand). The less polarized segments are malleable in a non-polar aprotic medium and may adopt helical (3_1_-helix, PP_II_-helix, α*-helix) folds while the least polarized segments of the polypeptide backbone, e.g. a sequence of consecutive ‘ *FP*_*i*_>>0 turns’ (Fig 5), may adopt the extended (C_5_* strand) folds depending on molecular embedding. **(b)** The *FP*_*i*_ values that ensure stability of the periodic secondary structure in a moderately polarizing environment such as the bilayer membrane interface, the interior of a soluble protein globule or the interior of the DNA duplex: the optimal *FP*_*i*_ range for the α-helix’ is ‒0.3–0 and the optimal *FP*_*i*_ ranges for β structure is <‒0.3 (C_5_ strand) and 0–0.3 (C_7eq_ strand). **(c)** The *FP*_*i*_ values that ensure stability of the periodic secondary structure in a polar medium such as the physiological 1:1 electrolyte solution: the range of the optimal *FP*_*i*_ values for the α-helix is now 0–0.3 while the somewhat less and more polarized segments are likely to form β-sheets. The most polarized segments are now likely to form ‘ *FP*_*i*_<<0 turns’ or PP_II_-helix. The sequence of consecutive ‘ *FP*_*i*_>>0 turns’ forms a random coil in an aqueous buffer unless it is stabilized by molecular embedding in helical (3_1_-helix, PP_II_-helix, α*-helix) or extended (C_5_* strand) folds. **(d)** The *FP*_*i*_ values that ensure stability of the periodic secondary structure in the hypothetical highly polarizing environment such as the pre-organized ionic grid e.g. on the surface of a DNA or RNA strand (the sequence of consecutive ‘ *FP*_*i*_>>0 turns’ is likely to form here an α*-helix), or the microenvironment of the extended β structure of β solenoid or amyloid filament, *vide infra*.

It follows that the secondary structure propensity is a function of both the intrinsic pattern of main-chain polarization, defined here by the folding potential *FP*_*i*_, and the capacity of the environment to polarize the polypeptide backbone. The expected trend is shown in Fig 7: as the polarizing capacity of the environment increases on going from vacuum and lipids to aqueous buffers and cross-β structure, the values of *FP*_*i*_ which are optimal for the stability of a given element of secondary structure become more positive. For instance, the free energy Δ*G*_coil→helix_ has one minimum with respect to the folding potential, cf. the *FP*_*i*_ region color-coded red in the diagram. It is expected that a negative value of *FP*_*i*_ is required to ensure helix stability in nonpolar environments, Fig 7(a) and 7(b), and that upon the transfer to an aqueous medium the position of the Δ*G*_coil→helix_ minimum shifts to a positive value of *FP*_*i*_, Fig 7(c).

We propose that the tendency to maintain congruity of the folding potential, medium and secondary structure drives the organization of the tertiary structure. Upon the collapse of the polypeptide chain or its segment into a compact conformation, the emerging shell→core depression or elevation of dielectric permittivity generates *the folding basin FB*. The position of an element of secondary structure within the folding basin, i.e. either in the interior or on the surface of the compact structure, depends on the deviation of its folding potential from the optimal ‘helix’ or ‘strand’ value. For instance, in the aqueous buffers, the helix with the more *negative* than optimal folding potential, e.g. incorporating the ‘C_5_ strand’ segment, will be stabilized when it is buried in the compact structure’s interior which has lower relative permittivity than the bulk of the solvent (the folding basin is a depression of relative permittivity with respect to the aqueous buffer). On the other hand, the helix with the more *positive* than optimal folding potential, e.g. incorporating the ‘ *FP*_*i*_>>0 turn’ or ‘C_7eq_ strand’ segment, will anchor the structure in the matrix of the ionic atmosphere, *vide infra*. In the lipid environment, the folding basin is an elevation of relative permittivity: the surface→core gradient of relative permittivity associated with the compact structure of a transmembrane protein is opposite to that associated with the soluble globules. Here the helix incorporating the ‘C_5_ strand’ segment will be stable when it is exposed to the lipid bilayer while the helix incorporating the ‘ *FP*_*i*_>>0 turn’ segment will be stabilized when it is buried, e.g. by oligomerization. In this view, globular fold of a soluble protein develops to bury backbone segment whose folding potential *FP*_*i*_ is fine-tuned to make a well-structured fold unstable in solvent but stable in a less polar environment of protein interior; architecture and stabilization of tertiary structure are brought about by selective destabilization of secondary structure.

#### (iv) The effects of charge separation in the medium: Folding template and bounds of tertiary structure

**Fig 8:**
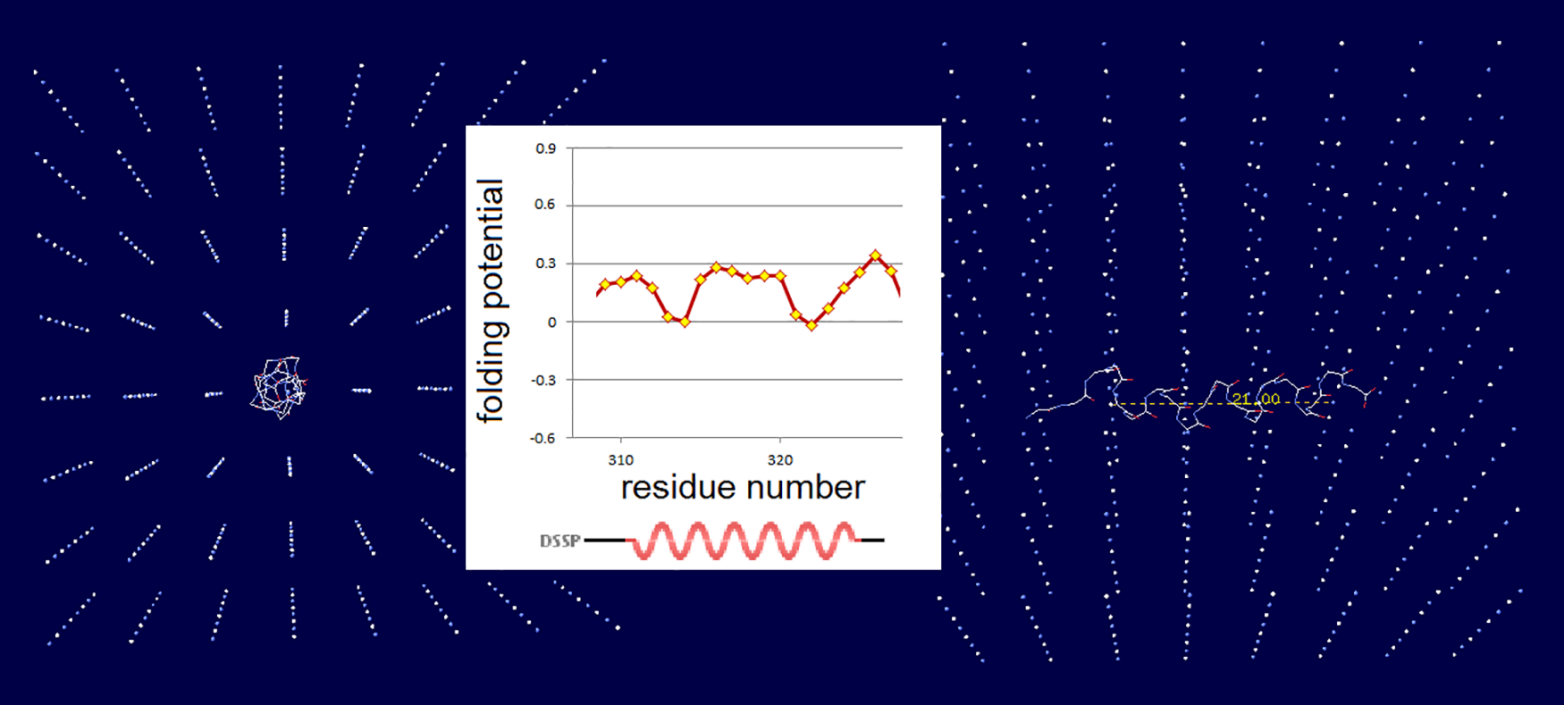
Folding potential, folding template and three-dimensional structure of soluble globular proteins. The insert shows the plot of the folding potential *FP*_*i*_ for the segment of the polypeptide backbone which has high helical propensity in the aqueous environment: the 14-residue site which triggers coiled-coil formation in cortexillin I [85]. In the physiological 1:1 electrolyte solution, this segment is stabilized by the mutual polarization of the α-helix and the transient ionic matrix with the lattice constant of 7 Å (the Ghosh-Debye-Hückel matrix, see the text and S1 Appendix). The effect of polarization is maximized when the helix termini replace the corresponding salt ions in the vertices of the lattice which are separated by the distances [(7Δ*x*_ij_)^2^+(7Δ*y*_ij_)^2^+(7Δ*z*_ij_)^2^]^1/2^ (Å) where Δ*x*_ij_, Δ*y*_ij_, Δ*z*_ij_ are whole numbers and the sum |Δ*x*_ij_|+|Δ*y*_ij_|+|Δ*z*_ij_| is odd. Thus the ‘allowed’ α-helix is a vector whose length is defined by the |Δ*x*_ij_|,|Δ*y*_ij_|,|Δ*z*_ij_| combinations equal to: {1,0,0}/7 Å, {1,1,1}/12 Å, {2,1,0}/15.6 Å etc. The length of the α-helix in the diagram is 21 Å which fulfils the above condition when the helix fits into the matrix along the grid line (|Δ*x*_ij_|,|Δ*y*_ij_|,|Δ*z*_ij_| = {3,0,0}, |τ_min_| = 0°), as shown here in both projections, or along the diagonal of the 2×2 segment of 4 unit cells (|Δ*x*_ij_|,|Δ*y*_ij_|,|Δ*z*_ij_| = {2,2,1}, |τ_min_| = 48°) (where |τ_min_| is the smallest vector/grid-line angle).

The effect of mutual polarization of the protein and its environment also depends on the intrinsic charge separation in the medium which may act as *the folding template* FT. The phospholipid bilayer can be thought of as such a folding template but beyond the obvious effect of low-permittivity environment of the lipid matrix it is not clear how its complex structural and physicochemical features would affect polarization of the polypeptide backbone. On the other hand, charge separation in the medium of cytosol or blood serum etc., i.e. in the 1:1 electrolytes (e.g. KCl, NaCl) under the standard physiological conditions, is better understood [82]. Here, the function of the folding template may be performed by the transient quasi cubic lattice of ionic atmosphere with the constant of 7 Å, the length that the Bjerrum distance and the Debye radius converge to in such solutions. The notion that the crystal-like lattice persists in dilute salt solutions was introduced by Gosh a century ago to explain nonideality of such solutions [83,84]. and was later refined by Debye and Hückel (see S1 Appendix) [48], hence the said lattice is here referred to as the Ghosh-Debye-Hückel matrix. The protein/electrolyte system is stabilized when the key surface charges are placed in the vertices of this ionic matrix; two charges *e*_i_ and *e*_j_ ought to be separated by the distance [(7Δ*x*_ij_)^2^+(7Δ*y*_ij_)^2^+(7Δ*z*_ij_)^2^]^1/2^ (Å) to fit into the lattice, where Δ*x*_ij_, Δ*y*_ij_, Δ*z*_ij_ are whole numbers and the sum |Δ*x*_ij_|+|Δ*y*_ij_|+|Δ*z*_ij_| is odd if the *e*_i_, *e*_j_ signs are opposite, and even if the *e*_i_, *e*_j_ signs are same, see Fig 8. The key surface charges may be the charges carried either by the ends of helices and cross-β arrays of the H-bonded peptide bonds (capped by helix turns, reverse turns or β bulges), or by the side chains (E^‒^, K, R). Note the corollary inference that soluble globular proteins and their environment may have evolved to take advantage of nonideality of dilute 1:1 electrolyte solutions. The hypothesis that “charged cytoplasmic macromolecules are stabilized electrostatically by their ionic atmosphere” and that the geometrical dimensions of biopolyelectrolytes and their polar functionalities may be related to the physiological ionic strength, was previously advanced in the context of modelling the organization of cytoplasm [86].

#### (v) Two-electron stabilizing interactions and the folding pathways of globular proteins

**Fig 9:**
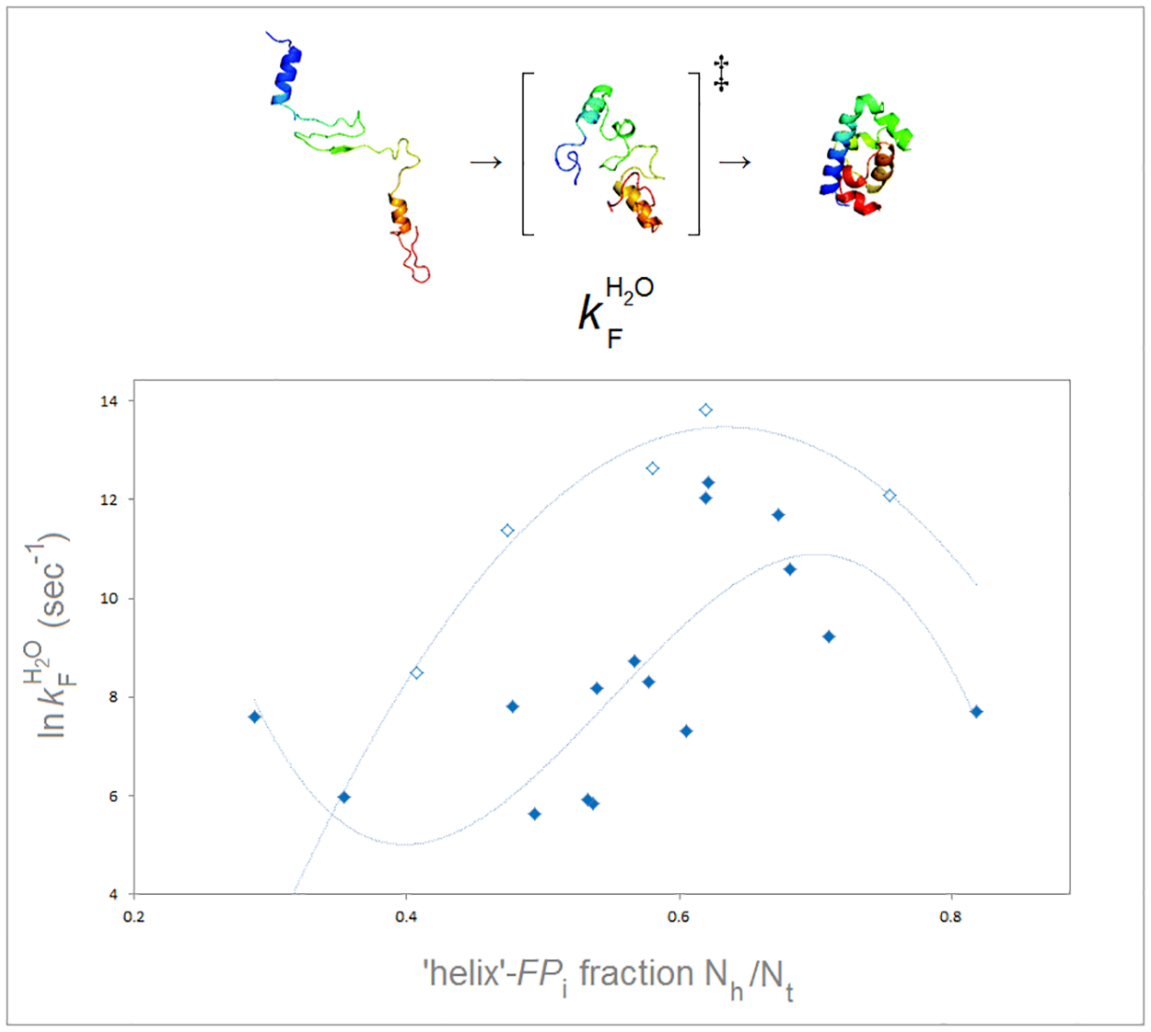
Electronic configuration of the polypeptide backbone and rate of folding of helix-bundle proteins. The folding rate constants ln *k*_*f*_ (sec^-1^) vs. *N*_h_/*N*_t_ (‘helix’- *FP*_*i*_ fraction) where *N*_h_ is the number of residues with the ‘helix’ *FP*_*i*_: 0±0.05–0.3±0.05, cf. Fig 7(c), and *N*_t_ is the total number of residues in the helix-bundle domains, counted from the N-terminal residue of the first α-helix to the C-terminal residue of the last α-helix as defined by the DSSP protocol implemented in the RCSB PDB database. The present set includes the data for 16 small proteins with the natural, wild-type sequences and for 5 domains modified or engineered for fast folding [87–100] *N*_t_ ≤ ~80 aa, PDB ID’s: 1ayi, 1ba5, 1fex, 1imp, 1mbk, 1prb, 1ss1, 1st7, 1uzc, 1yrf, 2a3d, 2abd, 2jws, 2jwt, 2no8, 2wqg, 3kz3: ♦ wild-type domains; ◊ the domains modified/engineered for fast folding.

Taken together, the results of our modelling studies and the inferences discussed above suggest that the following factors define how an amino acid sequence ‘selects’ the backbone fold: (1) the intrinsic pattern of backbone polarization imprinted by the side chains’ electronic effects (*FP*), (2) the emerging shell→core elevation or depression of relative permittivity and the placement and sequestration of side chains’ charges (*FB*), and (3) the constraints of an ionic or lipoid matrix (*FT*). Each factor’s effect varies along the folding pathway in a manner dependent on the contributions of the other factors, and each factor impacts the conformation of the protein by controlling, directly or indirectly, electronic configuration and bonding interactions of the peptide amide linkages. Thus, we propose that the pathway of folding of a globular protein comprises a sequence of conformational transitions driven by changes in the free energy of the polypeptide backbone which, to a considerable degree, are determined by the two-electron stabilizing interactions such as the generalized anomeric effect, homohyperconjugation of peptide linkages and covalent contributions to backbone-backbone H-bonding. The well-studied folding of the small soluble helix-bundle domains presents a system which seems to behave in this way: the ‘helix’ propensity of the domain, defined by the folding potential *FP*_*i*_, appears to have considerable impact on the folding rate constant *k*_f_^H2O^, Fig 9 [87–100]. This is consistent with the transition state ensemble having an approximately native topology but no fixed shell→core gradient of relative permittivity and no effective interaction with the transient lattice of the ionic atmosphere, so that the helices which incorporate the ‘strand’ and ‘turn’ segments are not fully stabilized in the transition state. Such a stabilization is eventually achieved in the native state and the dependence of the free energy of folding Δ*G*_U-F_^H2O^ on ‘helix’ propensity is obscured.

### b. Mechanism and principles of encoding the 3D structure of proteins: Explanatory and predictive power of the PMO model

#### (i) Stability of secondary structure as quadratic or quartic function of the folding potential *FP*

**Fig 10:**
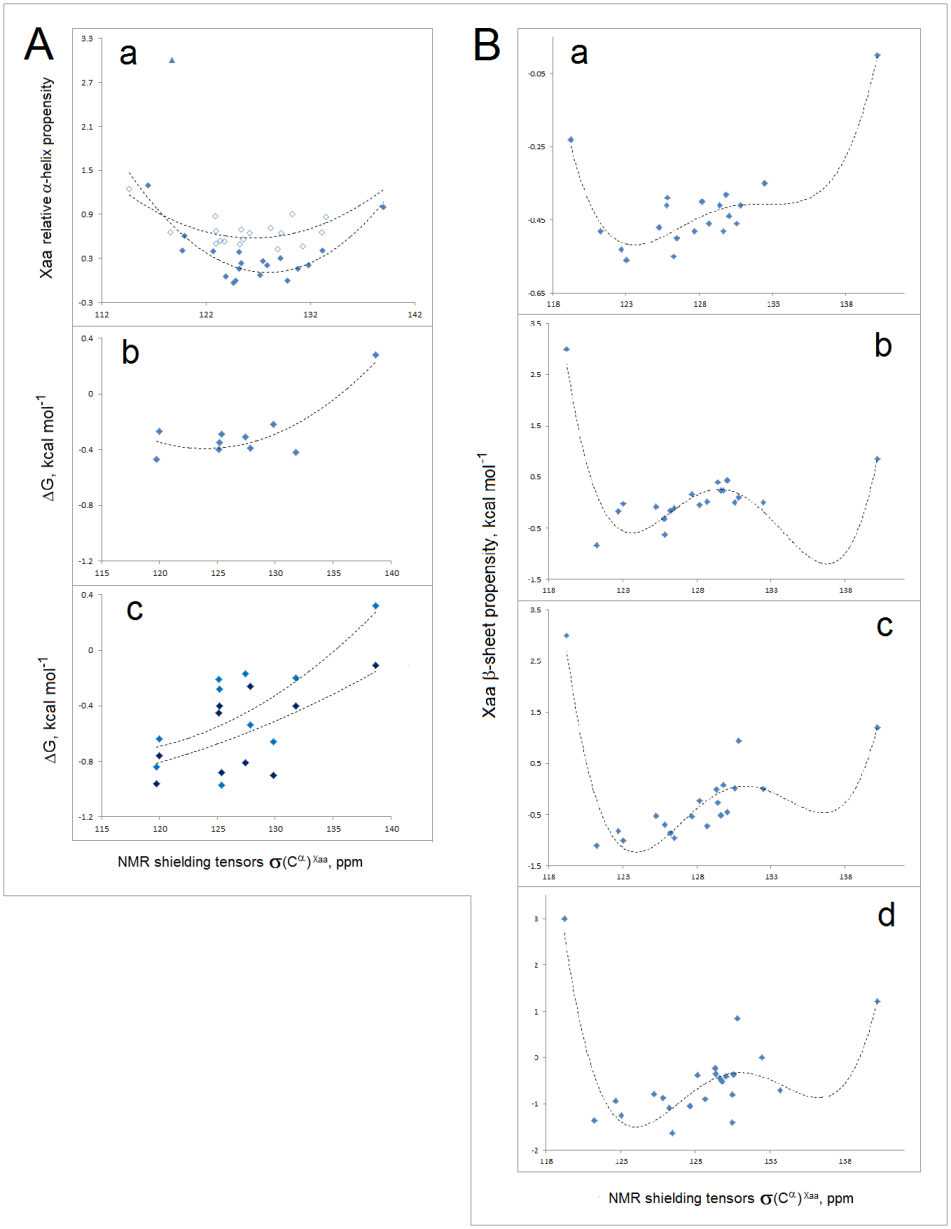
Electronic configuration of the polypeptide backbone and secondary structure propensity. **(A) Experimental α-helix propensities**: (a) The averaged relative α-helix propensity data obtained in the site-directed mutagenesis studies of both peptides and proteins, adjusted so that Δ(Δ*G*_f_) = 0 for Ala and Δ(Δ*G*_f_) = 1 for Gly [101–108], vs. the NMR shielding tensors **σ**(C^α^)^Xaa^ (3_10_-helix AcG(Xaa)GGGNH_2_; GIAO//B3LYP/D95**, cf. Computational Methods and S1 Table): ♦ glycine and amino acids whose C^β^ and C^γ^ are the methyl, methylene or methine groups, r^2^ = 0.83; ▲proline; ◊ any other amino acids including three highly fluorinated amino acids, r^2^ = 0.52 [107]; trendlines obtained by fitting 2^nd^ order polynomial functions; (b) The Lifson-Roig propagation free energies for the amino acids whose C^β^ and C^γ^ are the methyl, methylene or methine groups, in 88% methanol-water [109]; (c) The Lifson-Roig propagation free energies for the same set of amino acids in 40% (cyan) and 90% (navy) trifluoroethanol-water [109]. The propensities are determined at the sites in the helices interior. **(B) Experimental β-sheet propensities** from site-directed mutagenesis (kcal mol^-1^, Δ(Δ*G*_f_) = 0 for Gly in (D) and Δ(Δ*G*_f_) = 0 for Ala in (E), (F) and (G)) vs. calculated NMR shielding tensors **σ**(C^α^)^Xaa^ (AcGGGGGXaaNHMe in β-hairpin (Ib turn); GIAO//B3LYP/D95**, cf. Computational Methods and S1 Table): (a) zinc-finger β-hairpin, site 3, r^2^ = 0.89 (edge strand, the guest site is not H-bonded) [110]; (b) Ig binding B1 domain of streptococcal protein G, r^2^ = 0.83 (variant E42A/D46A/T53A, site 44, edge strand, the guest site is H-bonded) [111]; (c) Ig binding B1 domain of streptococcal protein G, r^2^ = 0.84 (variant I6A/T44A/T51S/T55/S, site 53, central strand) [112]; (d) Ig binding B1 domain of streptococcal protein G, r^2^ = 0.76 (I6A/T44A, site 53, central strand) [113,114]. Δ(Δ*G*_f_) for Pro in (b), (c) and (d) set at the minimum value of 3 kcal mol^-1^ [112]; trendlines obtained by fitting 4^th^ order polynomial functions.

To probe the dependence of the free energy Δ*G*_coil→helix_ and Δ*G*_coil→sheet_ on the electronic configuration of the polypeptide backbone, we examine a range of phenomena including single-site mutagenesis, amide-ester substitution, amyloidogenic propensity, and molecular recognition of PDZ domains. In Fig 10, thermodynamic secondary structure propensities [101–114] are plotted against the calculated tensors **σ**(C^α^)^Xaa^ (see the Computational Methods); these plots are consistent with the expected quadratic and quartic dependence for the α and β structure respectively.

**Fig 11:**
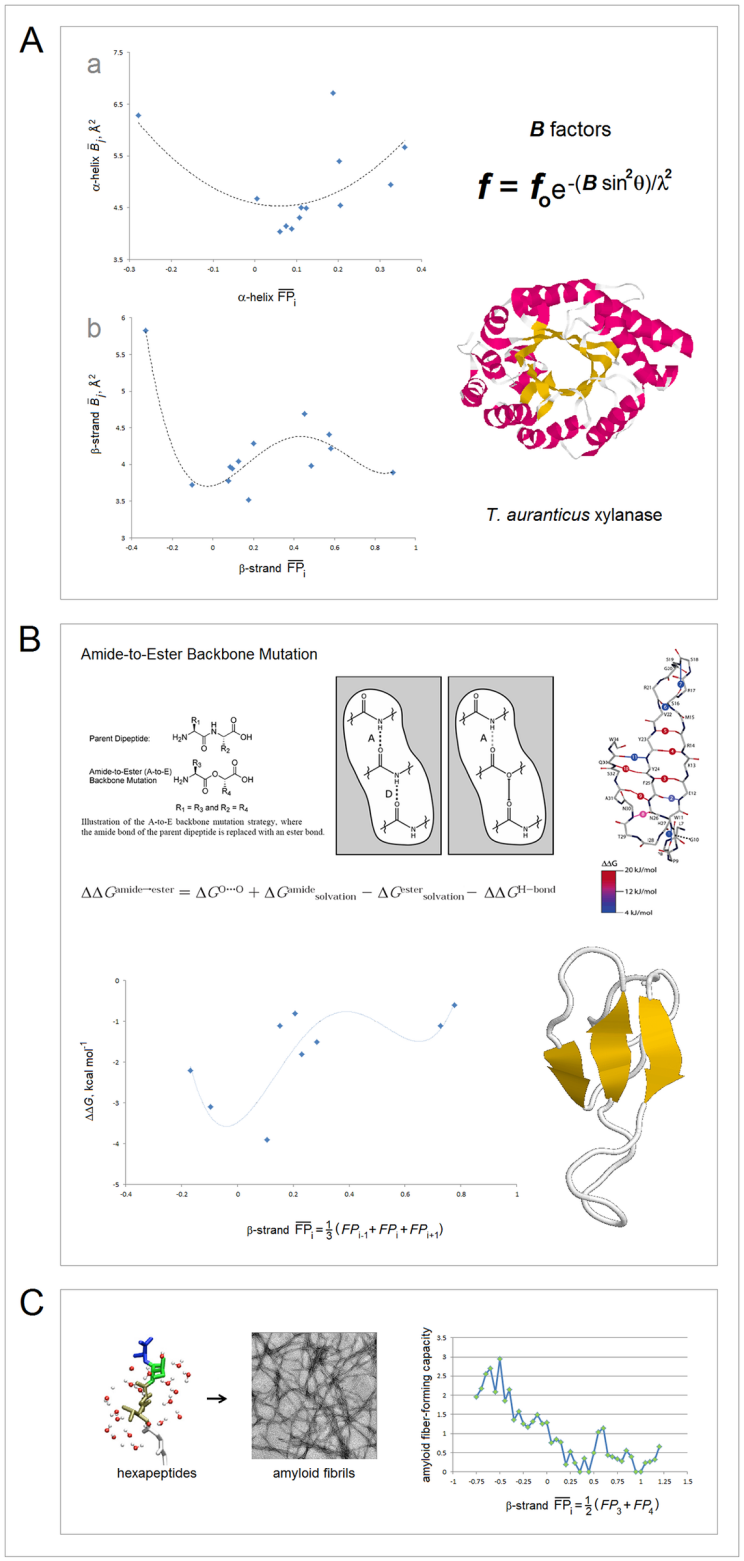
$\bar{{\text{FP}}_{\text{i}}}$ as a measure of conformational and H-bonding propensity of the polypeptide backbone. **(A**) $\bar{{\text{FP}}_{\text{i}}}$ **as a probe of backbone mobility**: (a) The mean temperature factors *B*_*i*_ of the backbone N atoms in α-helices vs. mean *FP*_*i*_ of those helices, $\bar{{\text{FP}}_{\text{i}}}$ (α), in the xylanase from *Thermoascus auranticus*, PDB ID 1i1wA [115]. Helical residues are assigned according to the Swiss-PDBViewer: helix **A** 6–12, **B** 24–27, **C** 32–38, **D** 51-54, **E** 64–76, **F** 93–96, **G** 101–117, **H** 143–147, **I** 151–163, **J** 182–197, **K** 215–227, **L** 245–257, **M** 292-301; (b) The mean temperature factors *B*_*i*_ of the backbone N atoms in β strands vs. mean *FP*_*i*_of those strands, $\bar{{\text{FP}}_{\text{i}}}$ (β). The strand residues are assigned according to the Swiss-PDBViewer and DSSP protocol implemented in the RCSB PDB database: **N** 17–22, **O** 41–46, **P** 79–81, **Q** 124–127, **R** 132–134, **S** 138–140, **T** 168–173, **U** 202–206, **V** 208–210, **W** 232–236, **X** 239–242, **Y** 264–266, **Z** 279–281. The trendlines are obtained by fitting 2^nd^ and 4^th^ order polynomial functions. **(B) $\bar{{\text{FP}}_{\text{i}}}$ and the energy of backbone-backbone H-bonding**. Δ(Δ*G*_f_) vs. *FP*_*i*_ for the single-site amide-to-ester X(i)ξ substitutions (Δ(Δ*G*_f_) = Δ*G*_f,WT_‒Δ*G*_f,X(i)ξ_) in Pin1 WW domain. The data shown for the mutants in which the perturbed amide donates, but does not accept, a hydrogen bond (thermal, GdnHCl, pH 7.0; PDB ID 2kcf) [116,117]. The trendline is obtained by fitting 4^th^ order polynomial function. **(C) $\bar{{\text{FP}}_{\text{i}}}$ and the amyloid fibril-forming capacity of oligopeptides**. The data for the total of 942 unique hexapeptide structures are taken from the WALTZ-DB database of amyloid forming peptides [118]; excluding the Pro-containing peptides, the sample comprises 240 amyloidogenic and 702 non-amyloidogenic hexapeptides. The mean *FP*_*i*_ of each hexapeptide, $\bar{{\text{FP}}_{\text{i}}}$ (peptide), is defined as the mean of *FP*_*i*_ of the two central residues. The amyloid fibril-forming capacity of the hexapeptides with $\bar{{\text{FP}}_{\text{i}}}$ within a specific 0.05 range (‒0.750±0.025 etc.) is defined as the frequency of the amyloidogenic peptides within this $\bar{{\text{FP}}_{\text{i}}}$ range in the entire amyloidogenic sample (240 entries), normalized by the frequency of both amyloidogenic and non-amyloidogenic peptides within the same $\bar{{\text{FP}}_{\text{i}}}$ range in the total hexapeptide sample (942 entries).

In Fig 11, we test the average value of the *FP*_*i*_ function, $\bar{{\text{FP}}_{\text{i}}}$, as a measure of the conformational and H-bonding propensity of a segment of the polypeptide chain. Several lines of evidence confirm that the $\bar{{\text{FP}}_{\text{i}}}$ values may indeed carry such information: (1) the plots of average temperature factors *B*_*i*_ indicate one minimum of backbone mobility with respect to $\bar{{\text{FP}}_{\text{i}}}$ in α-helices, and two such minima in the strands of β structure, Fig 11A(a) and 11A(b) [115]; (2) the Δ(Δ*G*_f_) data on the amide-to-ester substitutions suggest that the energy of backbone-backbone H-bonding in the β-sheet of Pin1 WW domain has two minima with respect to $\bar{{\text{FP}}_{\text{i}}}$ at the site of substitution, Fig 11B [116,117]; and (3) amyloidogenic propensity of linear hexapeptides appears to display two maxima with respect to the peptide $\bar{{\text{FP}}_{\text{i}}}$ [118], Fig 11C.

**Fig 12:**
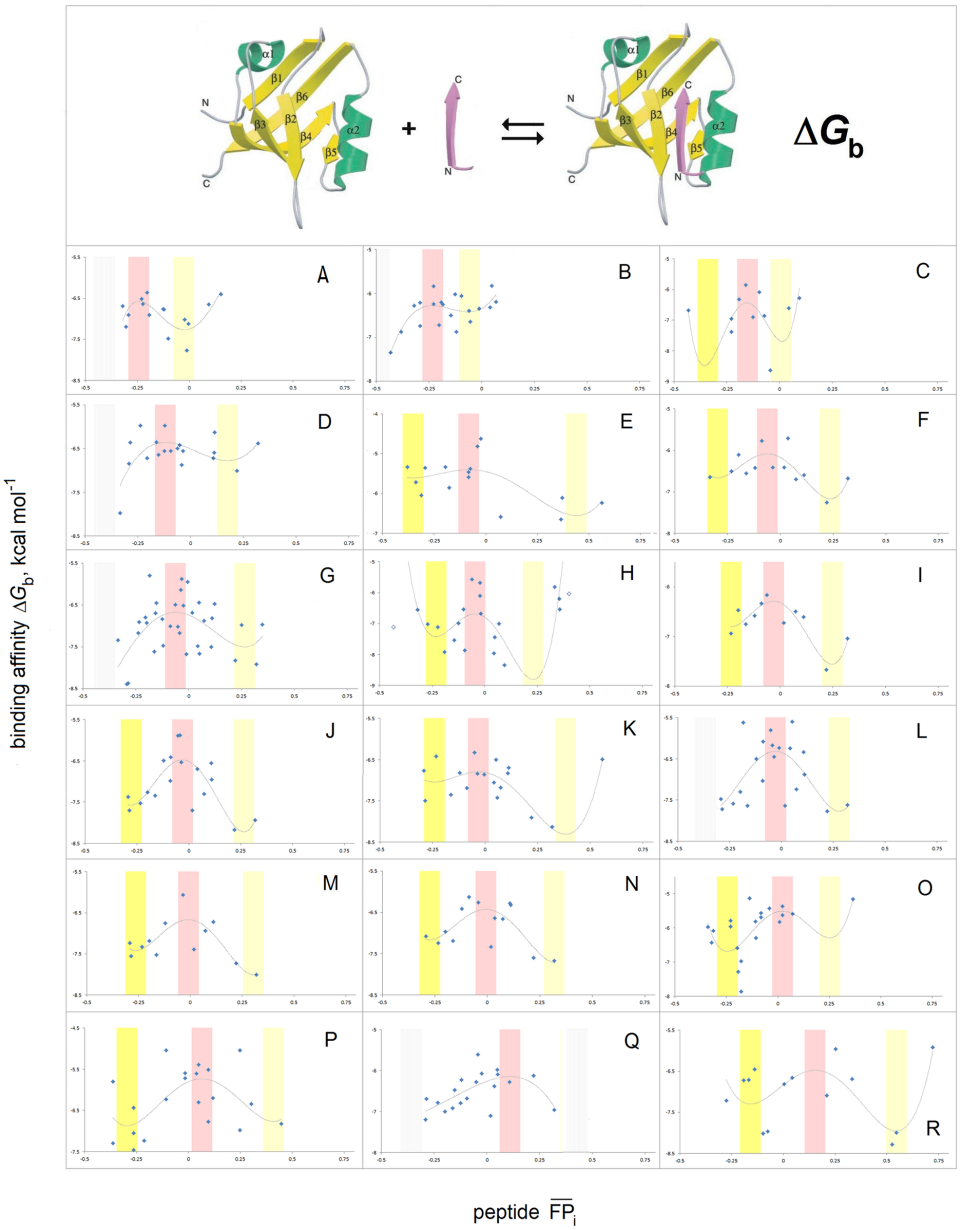
Electronic configuration of the polypeptide backbone and canonical peptide recognition by the PDZ domains. Binding affinities Δ*G*_b_ of the PDZ domains vs. mean *FP*_*i*_ of the peptide ligands $\bar{{\text{FP}}_{\text{i}}}$ (Δ*G*_b_ in kcal mol^-1^, the width of the *FP*_*i*_ averaging window is given in the brackets). The data from S2 and S3 Tables in ref. [120] unless indicated otherwise, for 18 out of 33 PDZ domains identified in the literature to bind at least a dozen or so peptides with *K*_d_<100 μmol (no discernible dependence of Δ*G*_b_ on $\bar{{\text{FP}}_{\text{i}}}$ in 3 cases, r^2^<0.25 in 11 cases, and in 1 case the fit depends on an outlier) [120–123]: **(A)** MAGI2 PDZ2, r^2^ = 0.52 (5); **(B)** PTP-BL, r^2^ = 0.52 (5); **(C)** MAGI1 PDZ6, r^2^ = 0.32 (8); **(D)** Lin7C, r^2^ = 0.38 (8); **(E)** AF6, r^2^ = 0.42 (5) [121]; **(F)** SAP97 PDZ1, r^2^ = 0.55 (8); **(G)** OMP25, r^2^ = 0.27 (8); **(H)** Scrb1 PDZ3, r^2^ = 0.31 (5); **(I)** PSD95 PDZ1, r^2^ = 0.77 (8); **(J)** Chapsyn110 PDZ2, r^2^ = 0.60 (8); **(K)** MAGI3 PDZ1, r^2^ = 0.53 (8); **(L)** SAP102 PDZ2, r^2^ = 0.42 (8); **(M)** PSD95 PDZ2, r^2^ = 0.59 (8); **(N)** SAP97 PDZ2, r^2^ = 0.53 (8); **(O)** Erbin, r^2^ = 0.40 (5) [121]; **(P)** TIAM1+TIAM2, r^2^ = 0.34 (5) [122]; **(Q)** γ-Syntrophin1, r^2^ = 0.42 (8); **(R)** RGS3, r^2^ = 0.48 (5). The $\bar{{\text{FP}}_{\text{i}}}$ regions associated with peptides’ propensities for the α-helix, C_5_ and C_7eq_ folds are shaded red, yellow and light yellow, respectively, cf. Fig 1; the regions shaded grey mark the putative Δ*G*_b_ minima.

The characterization of the conformational and H-bonding propensity of a segment of the polypeptide backbone in terms of $\bar{{\text{FP}}_{\text{i}}}$ may also be valid in more complex systems. The canonical binding of oligopeptides by the PDZ domains [119] involves extension of the domain’s β structure: the oligopeptide is inserted into the binding pocket as the edge strand of the antiparallel β sheet. The plots of binding affinity Δ*G*_b_ [120–123] against the average *FP*_*i*_ value of the peptide ligand, $\bar{{\text{FP}}_{\text{i}}}$ (peptide), seem to confirm the expected quartic dependence of Δ*G*_b_ with respect to $\bar{{\text{FP}}_{\text{i}}}$, see Fig 12.

**Fig 13:**
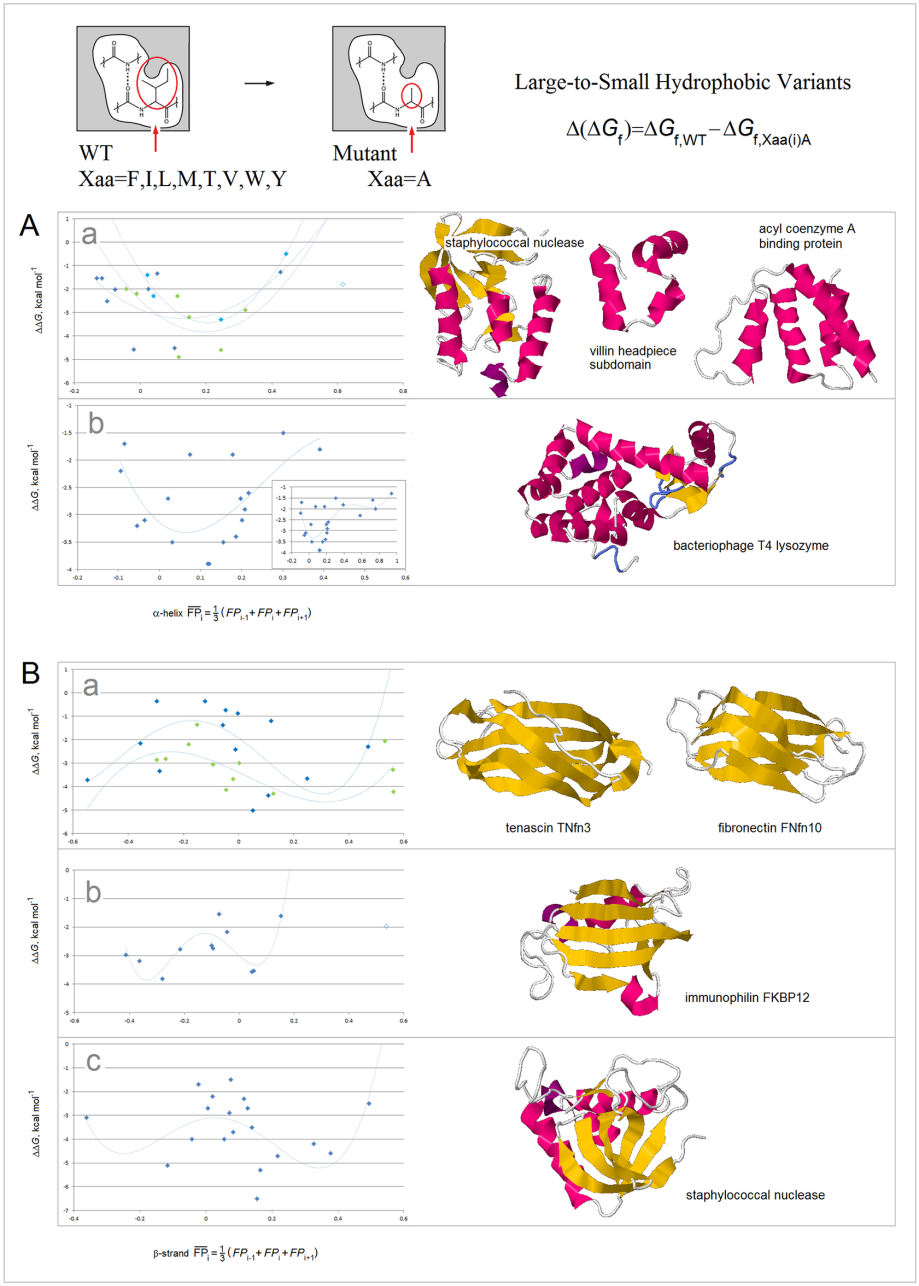
Electronic configuration of the polypeptide backbone and stability of large-to-small hydrophobic variants. The figure presents the Δ(Δ*G*_f_) vs. $\bar{{\text{FP}}_{\text{i}}}$ plots for the single-site Xaa(i)Ala mutations in helices and sheets (Δ(Δ*G*_f_) = ΔG_f,WT_‒Δ*G*_f,Xaa(i)Ala_, $\bar{{\text{FP}}_{\text{i}}}$ = ⅓ (*FP*_*i-1*_+ *FP*_*i*_ + *FP*_*i+1*_); Xaa = F, I, L, M, T, V, W, Y). The ‘helix’ and ‘strand’ residues are assigned based on the DSSP protocol implemented in the RCSB PDB database; the trendlines are obtained by fitting polynomial functions [125–131]: **(A)** The Δ(Δ*G*_f_) data for the α-helices in: (a) staphylococcal nuclease (GdnHCl, pH 7.0 [125], PDB ID 1nuc), villin headpiece subdomain (thermal, pH 7.0 [126], PDB ID 1yri, the data set of the highest $\bar{{\text{FP}}_{\text{i}}}$ (shown) is not included in the calculation of the trendline), and acyl coenzyme A binding protein (GdnHCl, pH 5.3 [87], PDB ID 2abd); (b) bacteriophage T4 lysozyme (thermal, pH 3.0 [127,128], PDB ID 2lzm, the complete scattergram, including the four data sets of the highest $\bar{{\text{FP}}_{\text{i}}}$, is shown in the insert). **(B)** The Δ(Δ*G*_f_) data for the β-sheet strands in: (a) fibronectin type III domains of human tenascin TNfn3 (3^rd^ module, PDB ID 1ten) and fibronectin FNfn10 (10^th^ module, PDB ID 1fnf) (thermal, urea, GdnHSCN, pH 5.0) [129,130]; (b) immunophilin FKBP12 (urea, pH 7.5 [131], PDB ID 2ppn, the data set of the highest $\bar{{\text{FP}}_{\text{i}}}$ (shown) is not included in the calculation of the trendline); (c) staphylococcal nuclease (GdnHCl, pH 7.0 [125], PDB ID 1nuc, the data set of the highest $\bar{{\text{FP}}_{\text{i}}}$ (not shown) is not included in the calculation of the trendline).

Lastly, the Δ(Δ*G*_f_) differences in stability of the large-to-small hydrophobic variants—used to estimate the free energy of hydrophobic interactions [124], appear to stem in good part from the changes in the conformational and H-bonding propensity of the main chain as well, see Fig 13. The Xaa→Ala mutation (Xaa = F, I, L, M, T, V, W, Y) may destabilize the native state by changing, among others, backbone’s folding potential *FP*_*i*_. The deleterious effect of such a change is expected to be particularly significant when the mutation occurs in the region of high congruence of the folding potential, environment and secondary structure, i.e. in the well-ordered segment that anchors the fold. Thus, the difference Δ(Δ*G*_f_) in stability of the Xaa**→**Ala hydrophobic variants should have one minimum with respect to *FP*_*i*_ in α-helices but two such minima in β-sheet strands. The available data seem to support this notion: the plots in Fig 13 show that Δ(Δ*G*_f_) has one minimum with respect to $\bar{{\text{FP}}_{\text{i}}}$ at the mutation site in α-helices [87,125–128] (Fig 13A), and two such minima in β-sheets [125,129–131] (Fig 13B).

#### (ii) Stability of secondary structure as a function of environment: Continuous dielectric

The plots of Lifson-Roig propagation free energies obtained in the 88%methanol-water and 40% and 90% trifluoroethanol-water mixtures, Fig 10A(b) and 10(c), when compared to the plots of α-helix propensities in water in Fig 10A(a), confirm that the expected shift of secondary structure propensity, cf. Fig 7, indeed takes place. These data are consistent with the data for other nonpolar environments e.g. lipid micelles and vesicles [132], and the gas phase, where Val and Ile are better helix-formers than Ala [133].

#### (iii) Stability of secondary structure as a function of microenvironment: Extended β structure

Polarization of the polypeptide backbone, and therefore secondary structure propensity, will be affected by the interactions with charges and partial charges within molecular embedding. Indeed, H-bonding interactions of the peptide amide bonds with ions e.g. within the ‘capping’ motifs or within the pre-organized ionic grid/ionic atmosphere of the DNA duplex surface [134,135], are expected to be highly polarizing. However, given the cooperativity of backbone-backbone H-bonding [136], the cross-β H-bonded arrays of extended β-structure elements, including asparagine and glutamine ladders, may also create a highly polarizing environment; the extraordinary dielectric properties of low-MW secondary amides are attributed to the formation of such extended arrays in bulk liquids [137]. Investigations of the amide I region in FTIR indicate that the microenvironment of amyloid fibrils does strengthen backbone H-bonding [138,139]; the maxima of amyloidogenic propensity, and especially the location of the ‘helical’ region (the minimum) at the $\bar{{\text{FP}}_{\text{i}}}$ (peptide) values between 0.25 and 0.50, Fig 11C, suggest that the amyloid’s microenvironment is indeed highly polarizing, cf. Fig 7(d).

**Fig 14:**
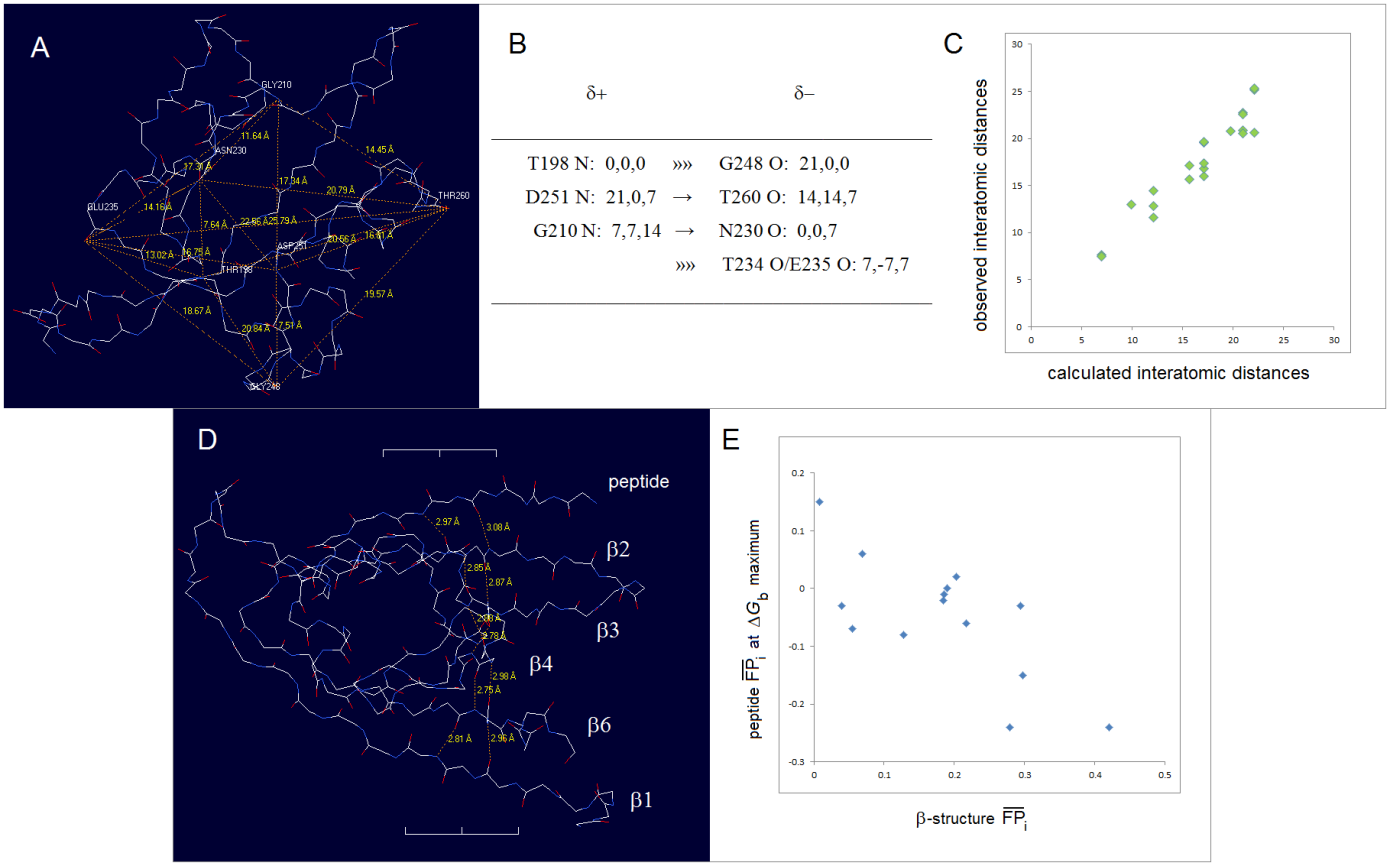
Electronic configuration of the polypeptide backbone and canonical peptide recognition by the PDZ domains. **(A)** Folding template *FT* and the 3D structure of the PDZ domains: the putative ‘key’ surface charges of the PDZ fold are the termini of the α1 helix-[CO_2_^−^ loop] and the α2 helix arrays (→), and the cross-β arrays (»») capped by a reverse turn (G248 (C’ = O)) and a bulge (T234 (C’ = O)/E235 (C’ = O)). The structure in the diagram is the second PDZ domain of syntenin, PDB ID 1r6j. **(B)** The projected fit of the putative key surface charges δ^+^ and δ^‒^ into the Ghosh-Debye-Hückel matrix. **(C)** The predicted by the folding-template model and the observed interatomic distances (Å) between the key surface charges δ^+^ and δ^−^ (the average distances to the T234 O/E235 O atoms in the bulge are used). **(D)** Two cross-β {β1-β6-β4-β3-β2} arrays of peptide bonds that anchor the oligopeptide ligand via backbone-backbone H-bonds to N-H of the residue *i* = ‒*2* and to C’ = O of the residue *i* = ‒*4*. **(E)** The oligopeptide $\bar{{\text{FP}}_{\text{i}}}$ values at the Δ*G*_b_ maximum (determined from Fig 12 for the 14 entries with β sheet register confirmed by the X-ray or NMR structure determination) plotted against $\bar{{\text{FP}}_{\text{i}}}$ (PDZ/sheet) i.e. the average *FP*_*i*_ values of the 15 residues involved in the two cross-β arrays which are shown in the panel D. For a detailed account of the model of peptide recognition by the PDZ domains see S3 Appendix.

The canonical binding of oligopeptides by the PDZ domains [119] previously examined in Fig 12, seems to be controlled by this effect. The data in Fig 12 are insufficient to fully define the minima in all the plots of the binding affinity Δ*G*_b_ vs. $\bar{{\text{FP}}_{\text{i}}}$ (peptide) but the positions of the Δ*G*_b_ maximum are accurate enough to say that these maxima shift between the $\bar{{\text{FP}}_{\text{i}}}$ (peptide) values of ca. ‒0.25 and 0.25. We expect, cf. Fig 7, that the shift occurs because the binding pockets of the PDZ domains differ in the capacity to polarize the peptide ligands; the correlation of the $\bar{{\text{FP}}_{\text{i}}}$ (peptide) values at the Δ*G*_b_ maximum with the *FP*_*i*_ of the PDZ cross-β structure, see Fig 14 and S3 Appendix, validates this notion.

#### (iv) Stability of tertiary structure as a function of the folding potential *FP* and the folding basin *FB*

The architecture and stabilization of tertiary structure are brought about, we have proposed in section **a**.(iii), by selective destabilization of secondary structure that aims to take advantage of the permittivity gradient of the protein’s folding basin *FB*. For instance, to form a compact bundle of α-helices in an aqueous buffer, protein sequence ought to comprise some backbone segments which have ‘helical’ Δ*FPi*_*-1→i+1*_ profiles and optimal ‘helical’ $\bar{{\text{FP}}_{\text{i}}}$ values (0–0.3 in water, cf. Figs 5 and 7) and will fold into solvent-exposed helices. At the same time, however, such a sequence also ought to incorporate backbone segments which have ‘helical’ Δ*FP*_*i-1→i+1*_ profiles but ‘non-helical’ *depressed* $\bar{{\text{FP}}_{\text{i}}}$ values (≤0, cf. Fig 7) and will fold into helices only in a low-permittivity environment e.g. when they are buried in the protein interior. The presence of those ‘destabilized-helix’ segments is necessary to organize and stabilize the tertiary structure of such a bundle.

**Fig 15:**
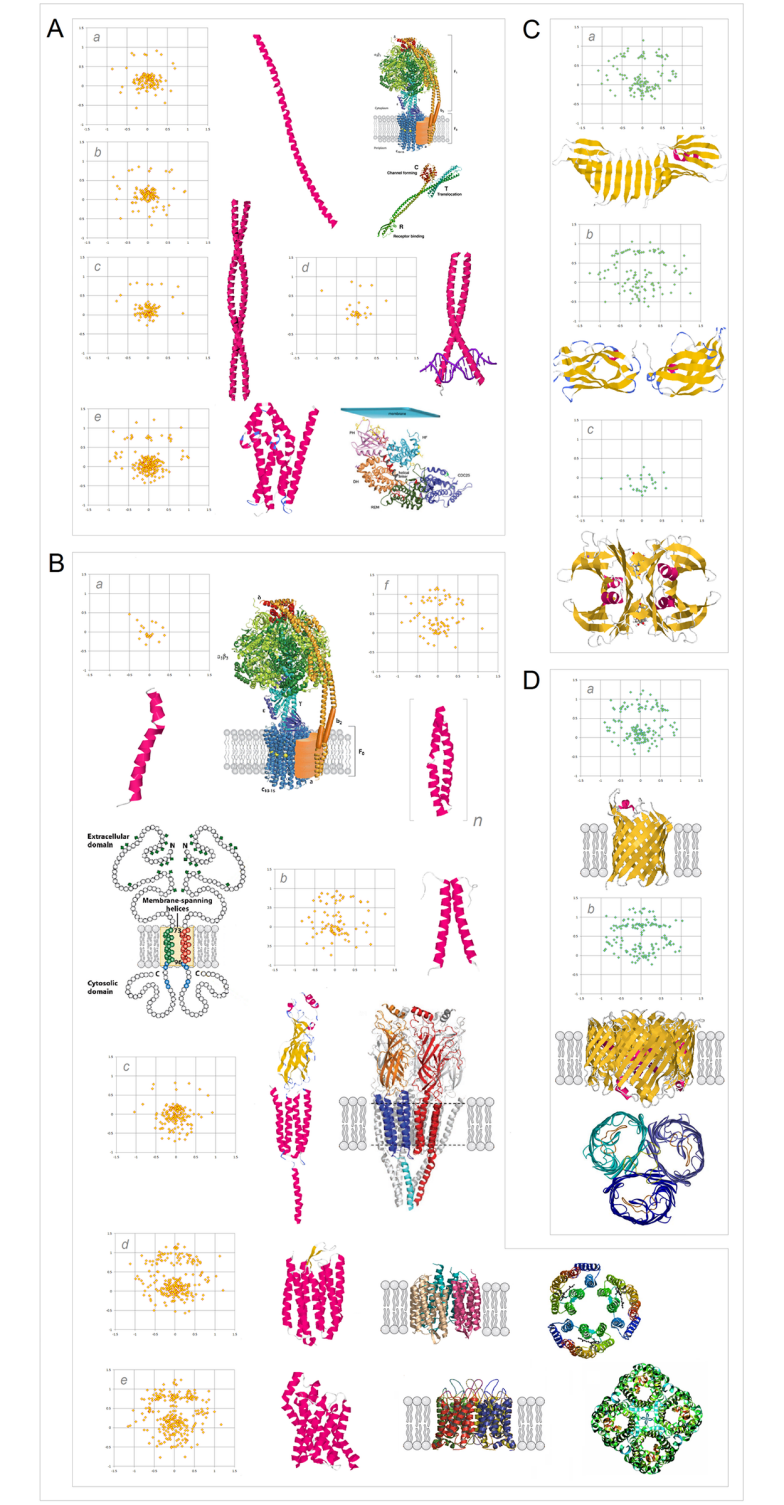
Electronic configuration of the polypeptide backbone versus complexity of tertiary structure and order of oligomerization in soluble and integral membrane proteins. The *FP*_*i*_ vs. Δ*FP*_*i-1→i+1*_ plots, cf. Fig 5, may characterize the relationship between distribution of backbone density and organization of globular structure. For α-helices, narrow distribution of the folding potential values and the slope values, Δ*FP*_*i-1→i+1*_ ~0, generates compact clusters of the data points. As the complexity of α structure increases and the exposure to the medium decreases, this clusters shift from *FP*_*i*_ >0 to *FP*_*i*_ <0 region in the case of soluble proteins, see the panels in **(A)**, and in the opposite direction, from *FP*_*i*_ <0 to *FP*_*i*_ >0 region in the case of integral membrane proteins, see the panels in **(B)**. The same trends are discernible in the *FP*_*i*_ vs. Δ*FP*_*i-1→i+1*_ plots for the antiparallel β sheets that assemble ‘C_5_ strands’ or ‘C_7eq_ strands’ and ‘*FP*_*i*_>>0 turns’, panels **(C)** and **(D)**, even though large differences in the folding potential values and wide distribution of the slope values, from Δ*FP*_*i-1→i+1*_ ~ ‒1 to Δ*FP*_*i-1→i+1*_ ~ 1, generate in this case circular distribution of data points. **(A)** (a) The peripheral stalk helix of F_0_F_1_ ATP synthase from *E*. *coli* (b_2_ subunit in the top diagram in the right-hand panel), UniProt # P0ABA0: residues A32-K122 (the hinge region, the dimerization region, and the C-terminal δ-domain-binding region); (b) One of the two 160Å-long helices of colicin Ia (residues R351-K470) that span the periplasmic space, linking the receptor-binding domain to the other domains, PDB ID 1cii; (c) The parallel α-helical coiled coil cortexillin I, PDB ID 1d7m; (d) The Leu-zipper segment of the GCN4 bZIP protein PDB ID 2dgc: the ‘helix’ cluster is shifted to the *FP*_*i*_ ~0 region; (e) The pattern of the multi-helix bundle which functions as an enzyme within a heterooligomeric complex: the ‘helix’ cluster is partly shifted into the *FP*_*i*_<0 region. The structure shown is the catalytic domain of the guanine nucleotide exchange factor (Ddl homology (DH) domain) of human PAK-interacting exchange protein, PDB ID 1by1. **(B)** (a) Transmembrane segment of the peripheral stalk of F_0_F_1_ ATP synthase from *E*. *coli* (b_2_ subunit in the diagram in the centre, color-coded orange), UniProt # P0ABA0: residues A11-A31; (b) Transmembrane α-helices of human glycophorins A, B, C and E: UniProt #’s P02724, P06028, P15421, and P04921. The glycophorin helices apparently are brought together in the membrane by the dimerization of the extra-membrane domains; (c) The transmembrane α subunit of the membrane associated acetylcholine receptor from *Torpedo marmorata*, PDB ID 2bg9; the five transmembrane subunits of this receptor do not form a tight oligomer structure and are held in place by the extra-membrane subunits; (d) Bacteriorhodopsin, a membrane protein (light-driven proton pump) from *Halobacterium salinarum*; the biological assembly is the homotrimer, PDB ID 1fbb; (e) Glycerol facilitator (GlpF), a membrane channel protein of the aquaporin family; the biological assembly is a homotetramer, PDB ID 1fx8; (f) Subunit *c* of F_0_F_1_ ATP synthase from *E*. *coli* (color-coded blue in the diagram in the centre): the biological assembly is a homodecamer, PDB ID 1ijp. **(C)** (a) The single-sheet protein, the central β-sheet (residues A81-D205) of the *Borrelia burgdorferi* spirochete antigen, outer surface protein A (OspA), PDB ID 2g8c; (b) The β sandwich C-terminal domain of the α-amylase from *Geobacillus stearothermophilus*, PDB ID 1qho; (c) The strands βF(H88-A97) and βH(S115-T123) of the homotetramer of transthyretin, buried in the tetramer interior, PDB ID 5l4j. **(D)** (a) 16-stranded β barrel, the monomeric integral outer-membrane porin OmpF from *E*. *coli*, PDB ID 1opr; (b) 16-stranded β barrel, the trimeric integral outer-membrane porin OmpG from *E*. *coli*, PDB ID 2f1c.

Thus, as the complexity of the native fold and the order of oligomerization of *soluble* proteins increase (the folding basin *FB* increases in size), backbone’s folding potential *FP* is expected to become more negative so that the *FP*_*i*_ averages for the major secondary structure elements are expected to shift to lower values. In contrast, cf. section **a**.(iii) and Fig 7, in the case of the *integral membrane* proteins, the *FP*_*i*_ averages of those elements are expected to shift to higher values as the complexity of structure and the size of the protein’s folding basin *FB* increase. A survey of the representative soluble and membrane proteins seems to validate this concept, see the *FP*_*i*_ vs. Δ*FP*_*i-1→i+1*_ plots in Fig 15.

**Fig 16:**
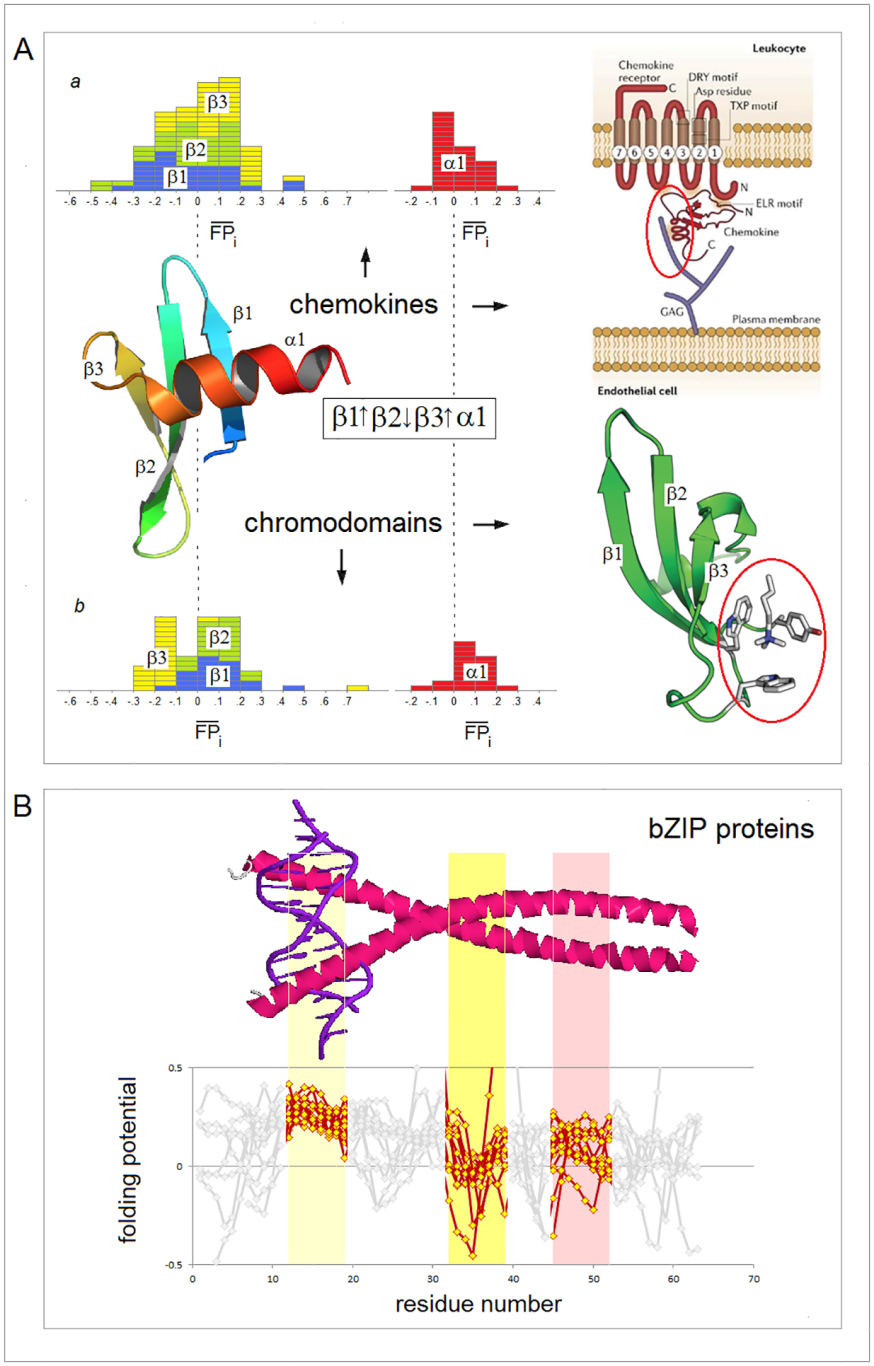
Electronic configuration of the polypeptide backbone versus complexity of tertiary structure and order of oligomerization. **(A)** The histograms compare the distribution of the α-helix and β-sheet $\bar{{\text{FP}}_{\text{i}}}$ in the chemokine and CHROMO domains [140,141]. The βββα folds of these two domains are very similar but the roles of the N-terminal and C-terminal segments are reversed: the chemokines use the C-terminal helix to bind the substrates while the CHROMO domains use the N-terminal β-hairpin. The expected difference in the $\bar{{\text{FP}}_{\text{i}}}$ distribution is indeed found (β1 cyan, β2 light cyan, β3 yellow, α1 red): (a) In chemokines, the binding function involves the C-terminal β3 and α1, and indeed the N-terminal β1 and β2 are the archetypal ‘C_5_ strands’ ($\bar{{\text{FP}}_{\text{i}}}$ largely <0) and anchor the structure while the C-terminal α1 will only be stable when it is partially buried ($\bar{{\text{FP}}_{\text{i}}}$ tends to be <0, PDB IDs: 1b3a, 1cm9, 1dok, 1eih, 1el0, 1eot, 1esr, 1f2l, 1f9p, 1g2t, 1g91, 1ha6, 1il8, 1j9o, 1m8a, 1mgs, 1ncv, 1nr4, 1o80, 1qg7, 1qnk, 1rhp, 2hcc, 2kol, 2kum, 2l4n, 2q8r, 2ra4, 3n52, 3ona, 3tn2, 4hcs, 4hsv); (b) In the CHROMO domains (binding involves the N-terminal β1 and β2), the C-terminal α1 is the archetypal ‘helix’ (0<$\bar{{\text{FP}}_{\text{i}}}$<0.2) and anchors the structure while the N-terminal β1 and β2 strands (also 0<$\bar{{\text{FP}}_{\text{i}}}$<0.2) will be stabilized when they are either partially buried and therefore less polarized (turning C_7eq_), or attached to a cross-β structure and therefore more polarized (turning C_5_) (PDB IDs: 1ap0, 1g6z, 1kna, 1pdq, 2b2v, 2d9u, 2dnt, 2dnv, 2dy8, 2ee1, 2epb, 2fgg, 2k1b, 2rsn, 2rso, 3fdt, 3gv6, 3i91, 3mts, 3r93, 3tzd). **(B)** The *FP*_*i*_ plots for bZIP proteins (PDB IDs: 1ci6, 1dgc, 1dh3, 1gd2, 1jnm, 1nwq, 1t2k, 1s9k, 2c9l, 2e43). The multiple alignment suggests that $\bar{{\text{FP}}_{\text{i}}}$ and function of each distinct segment of bZIP helix are related. Based on the assignments in Fig 7(c), the basic N-terminal segment has the ‘C_7eq_ strand’ $\bar{{\text{FP}}_{\text{i}}}$, the mid-region segment has the ‘C_5_ strand’ $\bar{{\text{FP}}_{\text{i}}}$, and the C-terminal segment has the ‘helix’ $\bar{{\text{FP}}_{\text{i}}}$. Thus, the folding potential *FP*_*i*_ of each segment appears optimized to ensure stability of the helical fold in three different environments created in the complex of bZIP dimer and DNA duplex.

In principle, protein-ligand interaction may also be described in terms of reorganization of the folding basin *FB*. The complex is likely to change backbone conformation and relative permittivity of binding site’s environment, and the $\bar{{\text{FP}}_{\text{i}}}$ s of the segments directly involved in the binding may deviate from the optimal values to accommodate such change. The $\bar{{\text{FP}}_{\text{i}}}$ variation in chemokines, chromodomains, and bZIP proteins, is consistent with this notion, see Fig 16. The chemokine and CHROMO domains have similar β1-β2-β3-α1 folds but the roles of the N-terminal and C-terminal segments are reversed: chemokines use the helix to bind to GAGs [140], while CHROMO domains use the β1-β2 hairpin to bind to N-Me_3_^+^ Lys of histones [141]. Accordingly, chemokines tend to have low-$\bar{{\text{FP}}_{\text{i}}}$ helices which will be stabilized in a low-permittivity environment and stable C_5_↑C_5_↓ hairpins, Fig 16A(a), while chromodomains tend to have stable solvent-exposed helices and low-$\bar{{\text{FP}}_{\text{i}}}$ C_7eq_↑C_7eq_↓ hairpins which will be stabilized in a low-permittivity environment, Fig 16A(b).

The bZIP proteins are long helical dimers which bind to the major groove of DNA duplex: the basic N-terminal segment interacts with DNA while the C-terminal leucine zipper domain triggers formation of the coiled-coil structure [85] and stabilizes the dimer. The *FP*_*i*_ plots for a multiple alignment of bZIP proteins show how each function is related to a distinct $\bar{{\text{FP}}_{\text{i}}}$ value, see Fig 16B (the assignments based on Fig 7): (1) the N-terminal segment has the ‘C_7eq_ strand’-*FP*_*i*_ i.e. the *FP*_*i*_ of a helix that is stable in a highly polarizing environment rather than in water; (2) the central segment has the ‘C_5_ strand’-*FP*_*i*_ i.e. the *FP*_*i*_ of a helix that is stable in a nonpolar environment rather than in water and therefore drives the aggregation of the coiled coil; and (3) the C-terminal segment has the ‘helix’-*FP*_*i*_ i.e. it is expected to form a stable in water α-helix which triggers formation of the coiled-coil structure [85].

#### (v) Stability of tertiary structure as a function of the folding template *FT*

**Fig 17:**
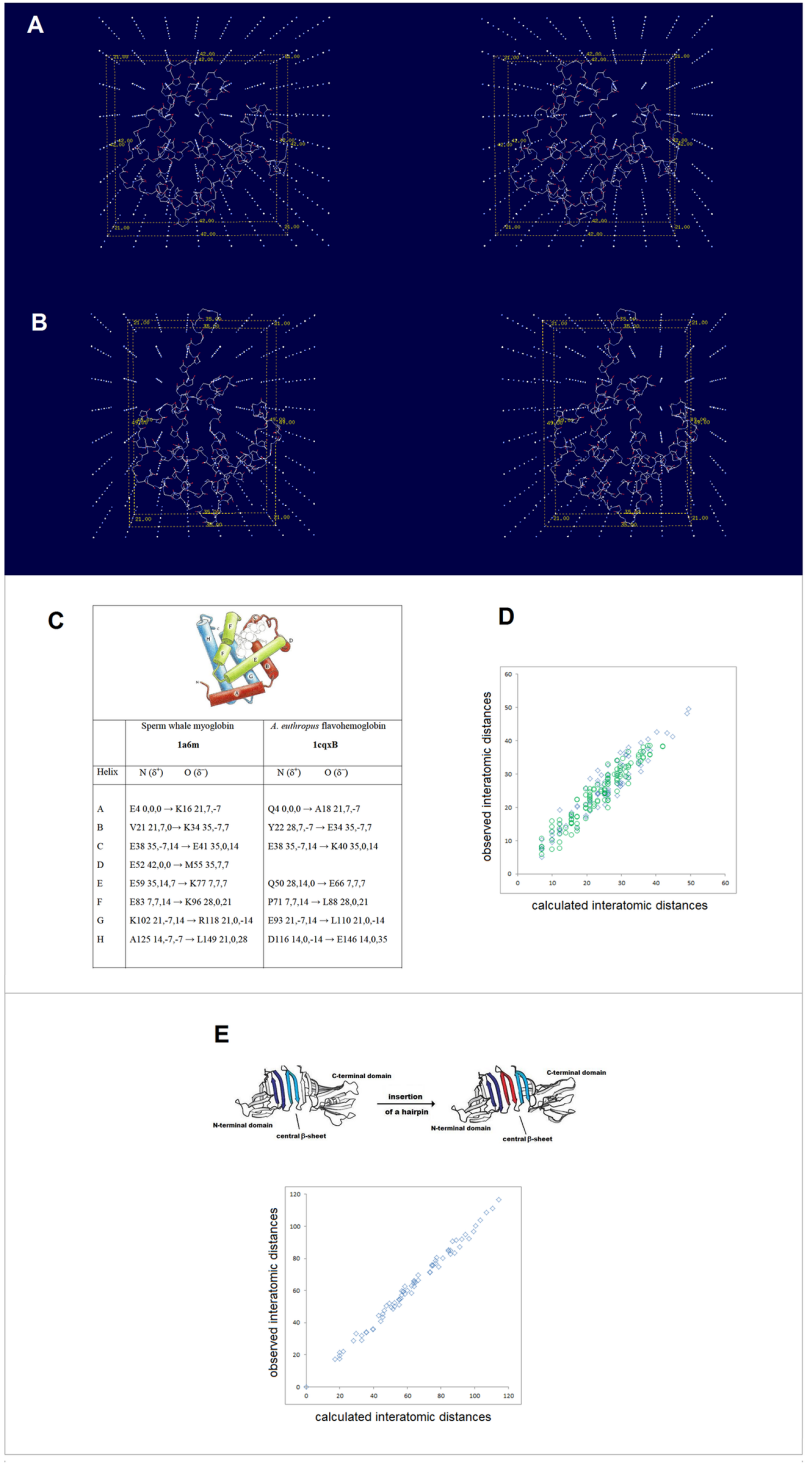
Folding template and tertiary structure of soluble globular proteins. **(A)** The interaction of sperm whale apomyoglobin, PDB ID 1a6m, with the transient cubic lattice of the ionic atmosphere in the physiological 1:1 electrolyte solution: mutual polarization of the polypeptide backbone and the Ghosh-Debye-Hückel matrix stabilizes the protein/electrolyte system when the ends of the helices A-H of the globin fold replace the corresponding salt ions in the vertices of the matrix with the 7 Å lattice constant. The ‘template box’ has dimensions 42×42×21 (Å). **(B)** In the case of *Alcaligenes euthropus* flavohemoglobin, PDB ID 1cqxB, the ‘template box’ has dimensions are 49×35×21 (Å) and it is polar along each Cartesian axis and so it is chiral. **(C)** Manual search for the best fit of the termini of the A-H helices of both globins into the Ghosh-Debye-Hückel matrix yields the coordinates shown in this panel (partial charges δ^+^ and δ^−^ of the helix termini are represented by the N and O atoms of the residues assigned as the terminal helix residues by the Swiss-PDBViewer with one exception of the N terminus of the helix B in 1a6m where PDBViewer assignment is erroneous; the Cartesian coordinate system is left-handed). **(D)** The calculated and the observed matrices of the interatomic distances (Å) between the helix termini in: ○ sperm whale apomyoglobin, PDB ID 1a6m, and ◊ *Alcaligenes euthropus* flavohemoglobin, PDB ID 1cqxB (based on the coordinates listed in the panel C). **(E)** The calculated and the observed matrices of the interatomic distances (Å) between the N^ξ^ atoms of the ‘reporter’ lysines in the congeners of *Borrelia* spirochete antigen OspA (PDB IDs 2af5, 2fkg, 2fkjA, 2fkjB, 2fkjC, 2g8c, 2hkd, 2i5v, 2i5z, 2ol6, 2ol7A, 2ol7B, 2ol8, 2oy1, 2oy5, 2oy7, 2oy8, 2oyb, 2pi3, 3ckaA, 3ckaB, 3ckf, 3ckg, 3ec5, 3eexA, 3eexB). See S4 Appendix for the definition of the plotted data.

As was argued in section **a**.(iv), mutual polarization of the protein solute and the transient cubic lattice of the ionic atmosphere in the physiological 1:1 electrolyte solution may stabilize the system if the key surface charges of the protein replace the corresponding salt ions in the vertices of the ionic matrix. For instance, in the case of globins, the protein/electrolyte system is likely to be stabilized when the termini of the α-helices are placed in those vertices, while in the case of the congeners of *Borrelia* spirochete antigen (outer surface protein A) the protein/electrolyte system is likely to be stabilized when the alkyl ammonium ions of the lysine side chains replace the salt cations in the vertices of the ionic matrix. The expected arrangement is indeed found, see Fig 17 and S4 Appendix. The network of backbone-backbone H-bonding in PDZ domains also appears to fit into this matrix, see Fig 14 and S3 Appendix.

#### (vi) Stability of tertiary structure and metamorphic equilibria: Refolding upon reorganization of the folding basin *FB*

The preceding discussion implies in what circumstances one amino acid sequence can support multiple folds. The ‘C_5_ strand’, ‘C_7eq_ strand’ and ‘turn’ segments that are incorporated into the α-helices to organize and stabilize globular tertiary structure or to ensure function-required mobility, may form bona fide elements of a stable β structure. On the other hand, the ‘helix’ segments may form the ‘C_7eq_ strands’ in a non-polar environment or the ‘C_5_ strands’ in a highly polarizing environment. All such relatively short segments would be identified as the *chameleon* sequences [142].

**Fig 18:**
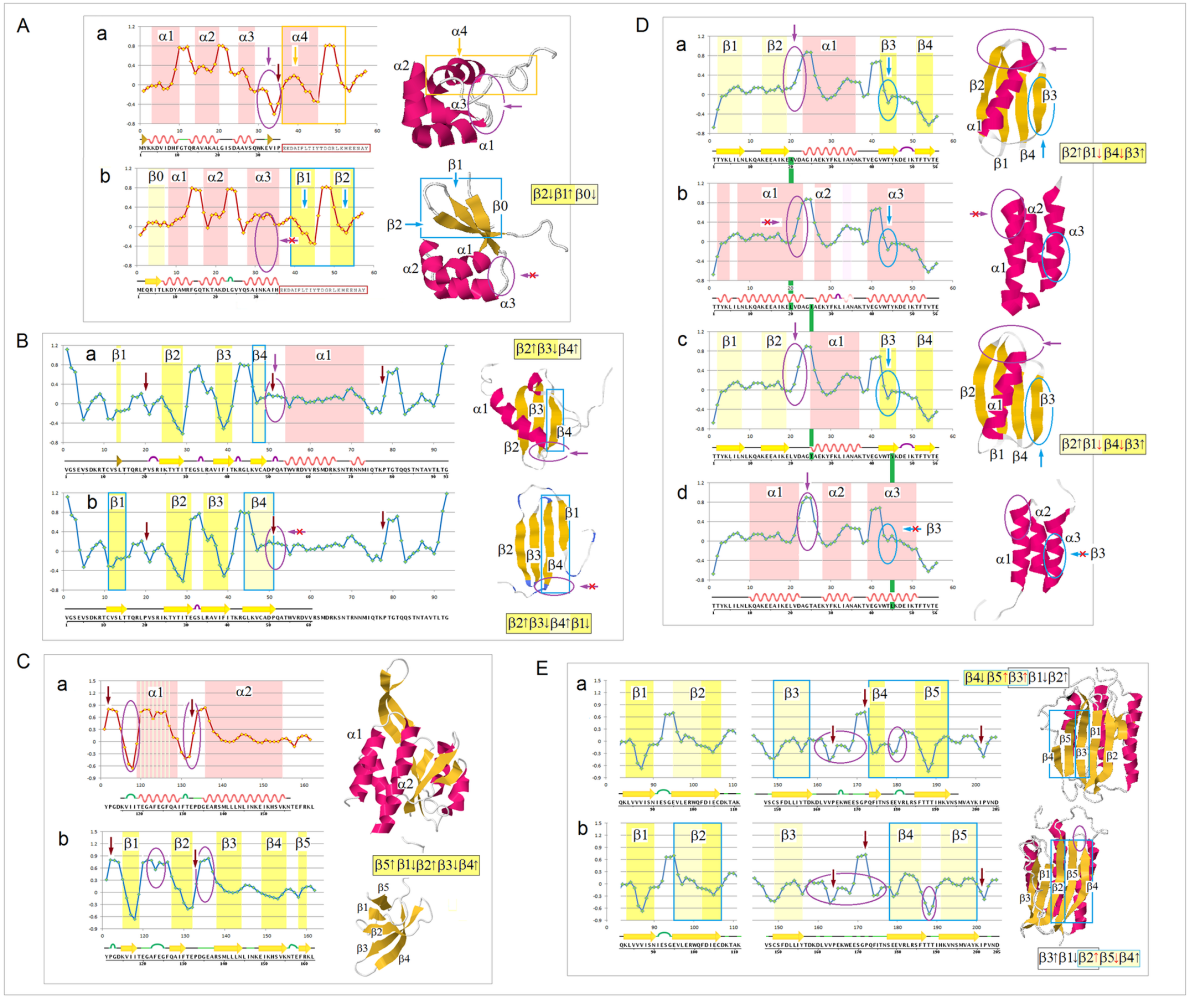
Electronic configuration of the polypeptide backbone and metamorphic equilibria. **(A) Chameleon sequence SASF2 attached to N-terminal segments of λ Cro and p22 Cro repressors**. When attached to the 35-residue N-terminal segments of p22 Cro (PDB ID 1rzs) or λ Cro (PDB ID 5cro) repressors, the sequence SASF2 mimics the conformations of the native C-terminal segments of these repressors, folding into an α-helix in the first case and into a β-hairpin in the second case [143]: (a) The *FP*_*i*_ plot for the p22 Cro/SASF2 conjugate. The chameleon sequence SASF2 is preceded by a flexible ‘hinge’ formed by the short C_5_ strand (W30-E32, $\bar{{\text{FP}}_{\text{i}}}$ = ‒0.1080) and the PP_II_ helix (V33-P35, $\bar{{\text{FP}}_{\text{i}}}$ = ‒0.4542), which extends back towards the helices α1, α2 and α3 (PDB ID 1rzs) so that the main chain makes U-turn placing SASF2 in the helix bundle; (b) The *FP*_*i*_ plot for the λ Cro/ SASF2 conjugate. The segment preceding SASF2 is the long helix α3 (S28-H35, $\bar{{\text{FP}}_{\text{i}}}$ = 0.1787) (PDB ID 5cro) and the backbone cannot make U-turn. **(B) Human lymphotactin**. Under physiological conditions, human lymphotactin exists in the equilibrium between the canonical chemokine monomer PDB ID 1j8i and the homodimer of the Greek-key β-sheet PDB ID 2jp1 [144,145]: (a) The *FP*_*i*_ plot for the monomeric chemokine-like conformer. The misalignment of the antiparallel C_5_ and C_7eq_ strands (β3 and β4) creates a flexible ‘hinge’ at the end of β4 so that the low-$\bar{{\text{FP}}_{\text{i}}}$ helix α1 can bury itself on the concave surface formed by the β structure and the unstructured, ‘S-S attached’ N-terminal segment; (b) The *FP*_*i*_ plot for the dimeric Greek-key conformer of lymphotactin. The stabilization of α1 is not possible when the extension of the three-stranded β2↓β3↑β4↓ sheet into the four-stranded one β2↓β5↑β4↓β1↑ obliterates the ‘hinge’ (β4 is lengthened due to additional alignment of β1) and flattens the lymphotactin surface (the N-terminal segment folds into β1). **(C) *E*. *coli* transcription factor RfaH**. The N-terminal (NTD) and C-terminal (CTD) domains of RfaH are connected by a flexible linker which allows either for tight interaction or complete separation of the two domains. The arrangement determines the fold of CTD: (a) The *FP*_*i*_ plot for the CTD partially buried in the NTD, PDB ID 2oug; (b) The *FP*_*i*_ plot for the autonomous CTD isolated in the aqueous environment, PDB ID 2lcl. **(D) G**_**B**_**98/G**_**A**_**98 variants of the streptococcal albumin- and immunoglobulin-binding proteins**. The *FP*_*i*_ plots for the 56-residue GB1 congeners and the alternation of folds produced in each case by the replacement of a single residue (mutation sites are marked by the green vertical bars) [146,147]: (a) The *FP*_*i*_ plot for the variant PDB ID 2lhe (G_B_98-T25I/L20A); (b) The *FP*_*i*_ plot for the variant PDB ID 2lhg (G_B_98-T25I); (c) The *FP*_*i*_ plot for the variant PDB ID 2lhd (G_B_98); (d) The *FP*_*i*_ plot for the variant PDB ID 2lhc (G_A_98≡G_B_98-Y45L). **(E) Human mitotic spindle protein Mad2** [148]: (a) The *FP*_*i*_ plot for the five-stranded β sheet of Mad2 with the C-terminal hairpin in the C_5_↓C_5_↑ configuration, PDB ID 1duj; (b) The *FP*_*i*_ plot for the five-stranded β sheet of Mad2 with the C-terminal hairpin in the C_7eq_↓C_7eq_↑ configuration, PDB ID 1s2h.

A much longer polypeptide chain can adopt more than one well-structured fold when a molecule comprises ‘malleable’ backbone segments which (1) have ambiguous conformational propensities (lack strong *FP*_*i*_ bias), (2) are amenable to the polarizing/depolarizing effects of the molecular embedding and the medium, and (3) are connected by a ‘hinge’ that controls their mutual orientation. A change in the conformation of the ‘malleable’ segment may then occur as a result of reorganization of the protein’s folding basin *FB* brought about by a change in the conformation of the ‘hinge’. When these two conformational transitions are coupled, the protein may exist in the metamorphic equilibrium [37]. The concept of the ‘hinge’-controlled reorganization of the folding basin *FB* and the concomitant metamorphic equilibration is illustrated in Fig 18.

In the first three cases [143–145], Fig 18A–18C, the common feature is the C-terminal segment which has ‘helical’ Δ*FP*_*i-1→i+1*_ profile but *depressed* $\bar{{\text{FP}}_{\text{i}}}$ (≤0) and may or may not be in close contact with the N-terminal subdomain depending on the conformation of the preceding ‘hinge’. Consequently, this segment folds into the α-helix in a low-permittivity environment i.e. when the C- and N-terminal subdomains are brought into close contact and the folding basin *FB* increases in size, cf. section **b**.(iv); otherwise, it may be incorporated into β structure or remain unstructured when exposed to solvent. In the fourth case, Fig 18D, the 56-residue protein switches folds back and forth between β grasp (ββαββ) and helix-bundle (ααα), each time as a result of replacement of a single residue in one of the two ‘hinges’ in the molecule [146,147]. In the β grasp fold, Fig 18D(a), the N-terminal subdomain of this protein forms the ‘C_7eq_ strands’ and β1-β2 hairpin, even though it lacks the ‘turn’ segment and has distinctly ‘helical’ Δ*FP*_*i-1→i+1*_ profile and optimally ‘helical’ $\bar{{\text{FP}}_{\text{i}}}$. To stabilize this fold: (1) the β1-β2 hairpin is placed in a low-permittivity environment by the contact with the mid-region α-helix buried on its surface, which requires that the connecting ‘hinge’ (~residues 20–25) is sufficiently long and stable; and (2) the β1-β2 hairpin is incorporated into extended β structure via parallel C_7eq_↑C_5_↑ alignment with the C-terminal β3-β4 hairpin. Thus, the A20L mutation, (a)→(b) in Fig 18D, apparently shortens the ‘hinge’ so that the helical propensity of the N-terminal segment prevails and prompts the transition to the three-helix-bundle fold; the compact all-α fold is stabilized by the incorporation of the ‘C_5_ strand’ segments into the helices α2 and α3. The I25T mutation, (b)→(c) in Fig 18D, stabilizes the required bend (increases the steep *change* in *FP*_*i*_ in the segment 20–25) and restores the β grasp fold. This fold, however, cannot be stable without the stable C-terminal C_5_↑C_5_↓ hairpin, and so the Y45L mutation that abolishes the C_5_ propensity of β3 strand also prompts the conversion into the helix bundle, see (c)→(d) in Fig 18D.

#### (vii) Stability of tertiary structure and metamorphic equilibria: Refolding of β structure that involves reorganization of the folding basin *FB* and *FP*-directed molecular recognition

The last example in Fig 18 is the refolding of β structure of the spindle checkpoint protein Mad2 [148]. Mad2 comprises the ‘fixed’ {β3↑β1↓β2↑}-sheet and the ‘malleable’ C-terminal β-hairpin {β4↑β5↓} attached by an unstructured loop. The interconversion between two distinct folded conformations of Mad2 involves reconfiguration of the 7-residue segment (S185-H191) which has highly negative $\bar{{\text{FP}}_{\text{i}}}$ (<<0). Based on the assignments in Fig 7, these 7 residues may adopt the ‘C_5_ strand’ configuration in a low-permittivity environment i.e. when they are buried, and the ‘*FP*_*i*_<<0 turn’ configuration when they are exposed to solvent. When such a configuration switch occurs, the C-terminal hairpin shifts its position along the chain, changing loop’s length and ‘strand’ propensities: it forms either (1) the ‘short loop’-C_5_↑C_5_↓ hairpin, Fig 18E(a), or (2) the ‘long loop’-C_7eq_↑C_7eq_↓ hairpin, Fig 18E(b). The shift reorganizes the folding basin *FB* because it determines how the {β3-β1-β2} and {β4-β5} fragments are attached to each other. In the first case, the arrangement is {β4-β5}-{β3-β1-β2}: the S185-H191 residues are buried as a part of the β5 ‘C_5_ strand’ which is parallel to the proximal β3 (the preferred alignment C_7eq_↓C_5_↓ for β3-β5 cf. Figs 2 and 5). In the second case, the arrangement is {β3-β1-β2}-{β5-β4}: the S185-H191 residues are exposed to solvent as the ‘turn’ of the β4-β5 hairpin, while the β5 ‘C_7eq_ strand’ is antiparallel to the distal β2 (the preferred alignment C_7eq_↑C_7eq_↓ for β2-β5). Thus, the reorganization of Mad2 β-sheet appears to be a case of the *FP*-directed molecular recognition. The difference in the β-sheet organization is also consistent with the difference in the loop’s reach (length).

#### (viii) Coupled folding and binding: Formation of α structure upon reorganization of the folding basin *FB*

**Fig 19:**
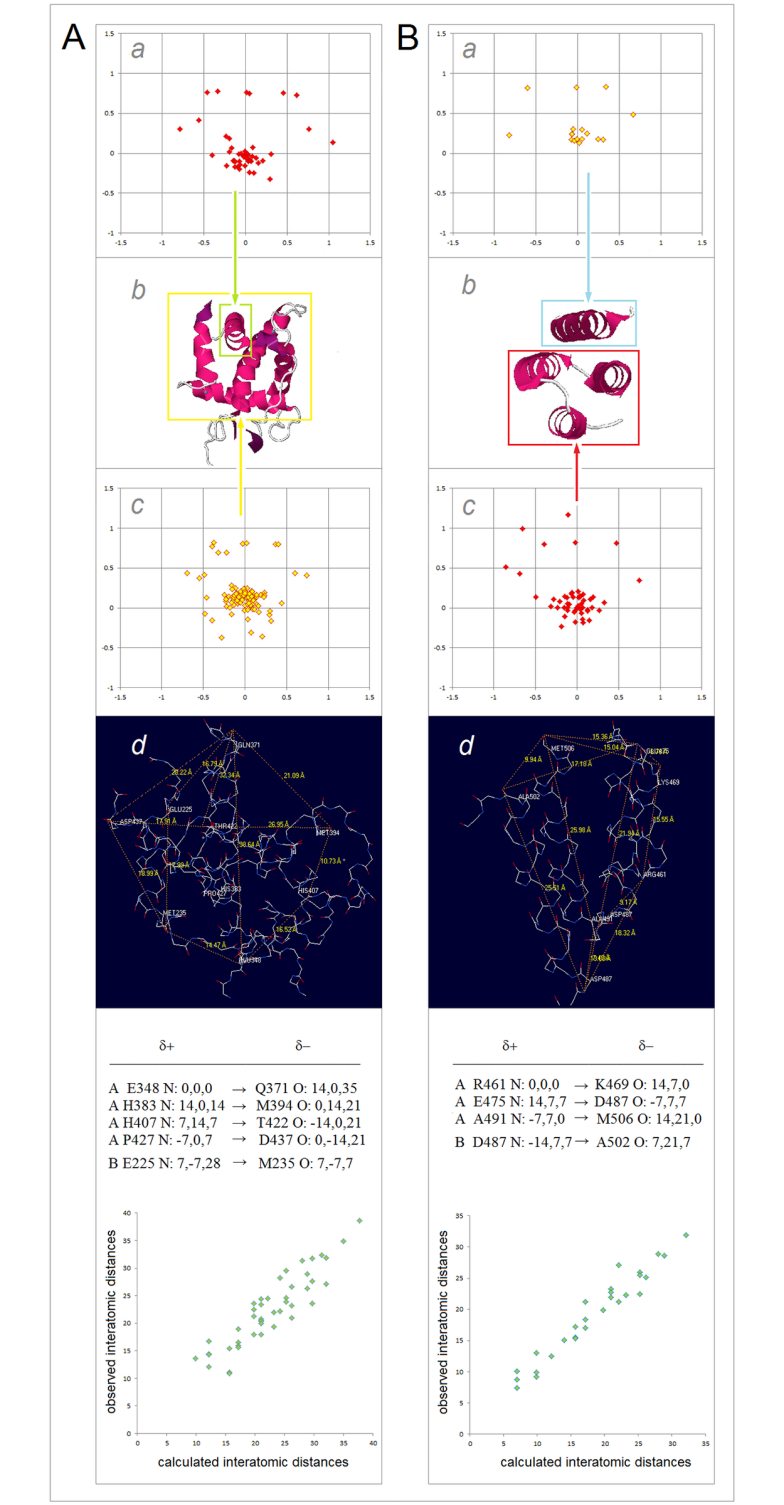
Electronic configuration of the polypeptide backbone and molecular recognition in formation of α structure: Folding of α-MoRFs [149–152]. **(A)** The complex between the TAZ1 domain of the transcriptional coactivator CREB-binding protein (CBP, the 4-helix-bundle IDP receptor) with the activation domain of CITED2 (IDP), PDB ID 1r8u [150]: (a) The *FP*_*i*_ vs. Δ*FP*_*i-1→i+1*_ plot for the activation domain of CITED2. The domain is unstructured in the free form in aqueous solution as expected given its *FP*_*i*_*/*Δ*FP*_*i-1→i+1*_ pattern: *FP*_*i*_<0; (b) The N-terminal segment of the activation domain of CITED2 is helical in the complex with TAZ1 where it is deeply buried (cf. the helix within the green outline) while the C-terminal segment, exposed to solvent, remains unstructured; (c) The *FP*_*i*_ vs. Δ*FP*_*i-1→i+1*_ plot for the CBP TAZ1 domain shows pronounced archetypal ‘helix’ propensity, cf. Fig 5. Note that the α-helices of this domain do not incorporate ‘C_5_ strand’ segments which would be expected to stabilize the bundle structure—in fact, the assembly is stabilized by the chelation of three Zn^2+^ ions; (d) The projected fit of the putative key surface charges δ^+^ and δ^−^ (the termini of the five helices shown in the Swiss-PDBViewer projection) into the Ghosh-Debye-Hückel matrix, and the correlation between the predicted by the folding-template model and the observed interatomic distances (Å) between the charges δ^+^ and δ^−^. **(B)** The complex between the α-MoRF of the measles virus N protein and nucleocapsid-binding domain of the measles virus P protein, PDB ID 1t6o [151,152]: (a) The *FP*_*i*_ vs. Δ*FP*_*i-1→i+1*_ plot for the α-MoRF. The *FP*_*i*_ profile is helical (approximately constant *FP*_*i*_) but $\bar{{\text{FP}}_{\text{i}}}$~0.3 is too high for the aqueous environment and the domain is disordered in solution; (b) Given its *FP*_*i*_*/*Δ*FP*_*i-1→i+1*_ pattern, the measles virus α-MoRF is expected to fold into a helix not when it is buried but when it remains in the solvent shell of the binding protein, benefiting from the synergistic stabilization of the ionic matrix, and this is indeed found in the complex, cf. the helix in the blue outline; (c) The *FP*_*i*_ vs. Δ*FP*_*i-1→i+1*_ plot for the nucleocapsid-binding domain shows pronounced ‘helix’ propensity characteristic of helix bundles, cf. Fig 15A; (d) The projected fit of the putative key surface charges δ^+^ and δ^−^ (the termini of the four helices shown in the Swiss-PDBViewer projection) into the Ghosh-Debye-Hückel matrix, and the correlation between the predicted by the folding-template model and the observed interatomic distances (Å) between the charges δ^+^ and δ^−^.

Molecular recognition features (MoRFs) [38,149], just like the ‘malleable’ segments of metamorphic proteins, can change conformation as a result of mere change in environment’s capacity to polarize the polypeptide backbone. Transfer of a disordered protein segment from water into a complex with another protein is one way to achieve such as change, see Fig 19. The *FP*_*i*_ vs. Δ*FP*_*i-1→i+1*_ plots show distinctly helical Δ*FP*_*i-1→i+1*_ profiles for the activation domain of CITED2 and the α-MoRF of the measles virus N protein but neither can fold in solution since $\bar{{\text{FP}}_{\text{i}}}$ is too low in the first case and too high in the second case, cf. Fig 19A(a) and 19B(a) [150–152]. To form a well-folded helix, the CITED2 domain needs to be buried in a protein ligand i.e. placed in a low-permittivity environment, and this is indeed found in the complex with the TAZ1 domain of the transcriptional coactivator CREB-binding protein (CBP) (Chart 11A(b), PDB ID 1r8u) [150]. In contrast, the viral MoRF, which forms a complex with the nucleocapsid-binding domain of the measles virus P protein (PDB ID 1t6o), remains bound in the polarizing environment of the protein surface/ionic atmosphere shell, Fig 19B(b) [151,152]. The corresponding difference in the organization of tertiary structure of the two all-α ligands is quite apparent in the *FP*_*i*_/Δ*FP*_*i-1→i+1*_ plots, see Fig 19A(c) and 19B(c). However, both complexes seem optimally set up for the interaction with the transient lattice of ionic atmosphere at the physiological conditions, see Fig 19A(d) and 19B(d).

#### (ix) Coupled folding and binding: Formation of β structure via *FP*-directed molecular recognition

**Fig 20:**
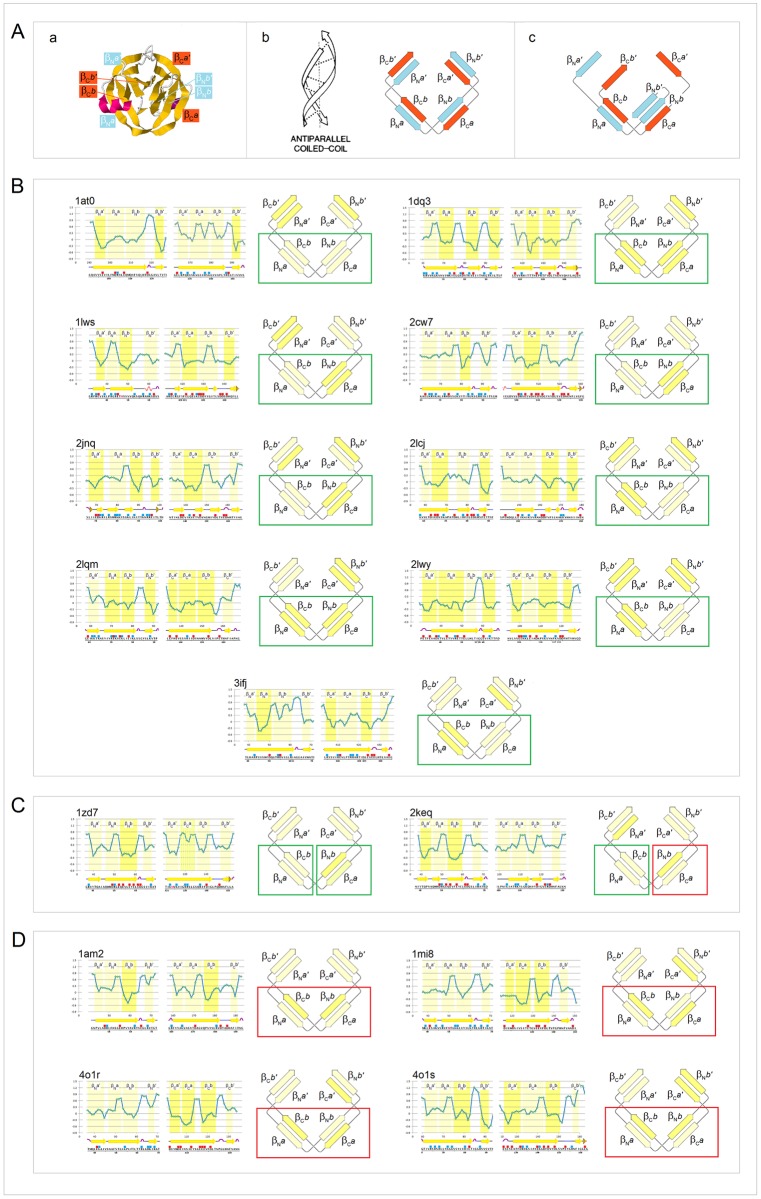
Electronic configuration of the polypeptide backbone and molecular recognition in formation of β structure: Folding of inteins [153,156]. The *FP*_*i*_ assignment of secondary-structure propensity vs. distribution of side chain charges in the two-stranded antiparallel β-sheet β_N_↑β_C_↓ of 15 inteins and intein-like domains of the known 3D structure. **(A)** The architecture of the horseshoe-like fold of inteins: (a) the antiparallel assembly of the 30–35 residue-long strands β_N_ and β_C_ into the β_N_↑β_C_↓ sheet constitutes the major feature of the HINT fold. Each strand comprises four segments, β_N_*a’*-β_N_*a*-β_N_*b*-β_N_*b’* and β_C_*a’*-β_C_*a*-β_C_*b*-β_C_*b’* (cf. the labels color-coded blue and red, respectively), which are marked by the changes in the direction of the main chain (produced by the inherent right-handed twist of the polypeptide backbone and the insertion of turns); (b) the putative assembly of the full-length β_N_ and β_C_ strands into the β_N_↑β_C_↓ sheet. The interactions of the terminal-segment pairs, β_N_*a’*-β_C_*b’* and β_N_*b’*-β_C_*a’*, may contribute to the stabilization of the correct register in the initial stages of folding; (c) in the native state, the β_N_↑β_C_↓ sheet comprises the middle-segment pairs β_N_*a*-β_C_*b* and β_N_*b*-β_C_*a* since the C-terminal segments of both strands, β_N_*b’* and β_C_*b’*, are folded towards the protein interior. **(B)** The *FP*_*i*_ assignment of secondary-structure propensity vs. distribution of the side chain charges in the case of the full ‘symmetry’ of backbone polarization of the middle-segment pairs β_N_*a*-β_C_*b* and β_N_*b*-β_C_*a*, shown within the green rectangles in the schematic representation of the β_N_↑β_C_↓ sheet. The PDB ID-labeled diagrams show *FP*_*i*_ plots, DSSP assignments, and the sequences of the 30–35 residue-long β_N_ and β_C_ strands with D, E marked by red squares and K, R marked by blue squares. The four segments of each β_N_ and β_C_ strand are defined based on the DSSP/Swiss-Prot assignments and the inspection of the 3D structures, and are shaded yellow or light-yellow in the *FP*_*i*_ plots depending on the secondary-structure preferences; the latter are assigned based on Figs 5 and 7. **(C)** The *FP*_*i*_ assignment of secondary-structure propensity vs. distribution of side chain charges in the case of the partial ‘symmetry’ of backbone polarization of the middle-segment pairs β_N_*a*-β_C_*b* and β_N_*b*-β_C_*a*. The ‘matching’ β_N_*a*-β_C_*b* pairs are shown within the green rectangles and the ‘mismatched’ β_N_*b*-β_C_*a* pair within the red rectangles in the schematic representation of the β_N_↑β_C_↓ sheet. **(D)** The *FP*_*i*_ assignment of secondary-structure propensity vs. distribution of side chain charges in the absence of the ‘symmetry’ of backbone polarization of the middle-segment pairs β_N_*a*-β_C_*b* and β_N_*b*-β_C_*a*, shown within the red rectangles in the schematic representation of the β_N_↑β_C_↓ sheet. For a detailed report on the discussed data see S5 Appendix.

Coupled folding and binding may also involve formation of β structure. Autonomous folding of a stable β sheet requires precise location and matching of distinct ‘strand’ and ‘turn’ propensities, see Figs 2 and 5. Thus, a polypeptide chain which comprises mismatched or lacking strong *FP*_*i*_ bias ‘strand’ and ‘turn’ segments will remain disordered unless the *anticipated* ‘strand’ configurations are stabilized in an extended intramolecular or intermolecular β structure. The synergistic folding of split inteins reveals a mechanism of molecular recognition that is likely to underlie such processes, in this case formation of the two-stranded antiparallel β-sheet β_N_↑β_C_↓ that anchors the horseshoe-like fold [153]. The β_N_ and β_C_ segments are 30-35 residue-long and remain disordered in solution but form upon binding an extensive left-handed coiled-coil which is a variation of the common fold of two-stranded antiparallel sheets expected to display ‘extraordinary flexibility’ [154]. In inteins, the coiled-coil has a non-uniform twist so that each strand comprises four distinct segments, β_N_*a’*-β_N_*a*-β_N_*b*-β_N_*b’* and β_C_*a’*-β_C_*a*-β_C_*b*-β_C_*b’*, and has, in addition, a left-handed superhelical twist, Fig 20A. This architecture appears consistent with the intrinsic right-handed twist of the extended strands, distribution of secondary-structure propensities and insertion of turn sequences, and length limitations on the cooperativity of backbone-backbone H-bonding [155]. According to our model, see Figs 2 and 5, molecular recognition in the assembly of the β_N_↑β_C_↓ sheet should be aided by the ‘symmetry’ of backbone polarization of β_N_ and β_C_, in particular by ‘matching’ of the middle-segment pairs β_N_*a*-β_N_*b* and β_C_*a*-β_C_*b*. The *FP*_*i*_ plots for the 15 inteins of known three-dimensional structure, see Fig 20B–20D, do show such a trend: the complete ‘symmetry’ of backbone polarization of the β_N_↑β_C_↓ middle segments is found in 9 structures, partial ‘symmetry’ in 2 structures (split inteins), and none in 4 structures, which yields the total of 20 *FP*_*i*_-‘matching’ and 10 *FP*_*i*_-‘mismatched’ middle-segment pairs. In principle, complementarity of the electrostatic charges carried by β_N_ and β_C_ could also aid folding of this coiled-coil as the folding-template effect, and it was indeed invoked to explain the kinetics of split-intein folding [156]. According to the present data, such assistance does appear to operate in some cases but it is not required: the β_N_ and β_C_ strands carry complementary charges in 11 cases, in one case both strands carry negative charge, and one or both strands are neutral in 3 cases. However, in the split inteins PDB IDs 1zd7 and 2keq, complementarity of *multiple* charges is found in the β_N_*b*-β_C_*a* segment pairs that lack the ‘symmetry’ of backbone polarization while in the symmetry-wise ‘matching’ β_N_*a*-β_C_*b* segment pairs both segments are either neutral (1zd7) or both carry negative charges (2keq), cf. Fig 20C. This trend is reproduced in the total sample: among the 20 ‘matching’ β_N_*a*-β_C_*b* and β_N_*b*-β_C_*a* pairs, both segments carry *the same charge* in 4 cases, in 11 cases one of the two segments is neutral, and the charges are complementary in 5 cases (with just a single charge on one of the segments), Fig 20B and 20C. On the other hand, among the 10 middle-segment pairs that lack the ‘symmetry’ of backbone polarization, the charges are complementary in 8 cases (with *multiple* charges on both segments in 4 cases), and only in 2 cases one of the two segments is neutral, Fig 20C and 20D. These findings are consistent with the notion that the side chain-side chain interactions may facilitate the initial stages of intein folding via the ‘electrostatic capture’ [156], or stabilize the 3D structure once the backbone fold is established, but neither effect is necessary to assembly the two-stranded antiparallel β-sheet of inteins in the correct register. The discrete architecture of the β_N_↑β_C_↓ sheet seems to depend on the complex pattern of backbone polarization, and the ‘symmetry’ of polarization of the β_N_*a*-β_C_*b* and β_N_*b*-β_C_*a* segments appears to be an important feature of this pattern. For the detailed account of the discussed data see S5 Appendix.

#### (x) pH-Driven equilibria: Transformation of secondary structure

**Fig 21:**
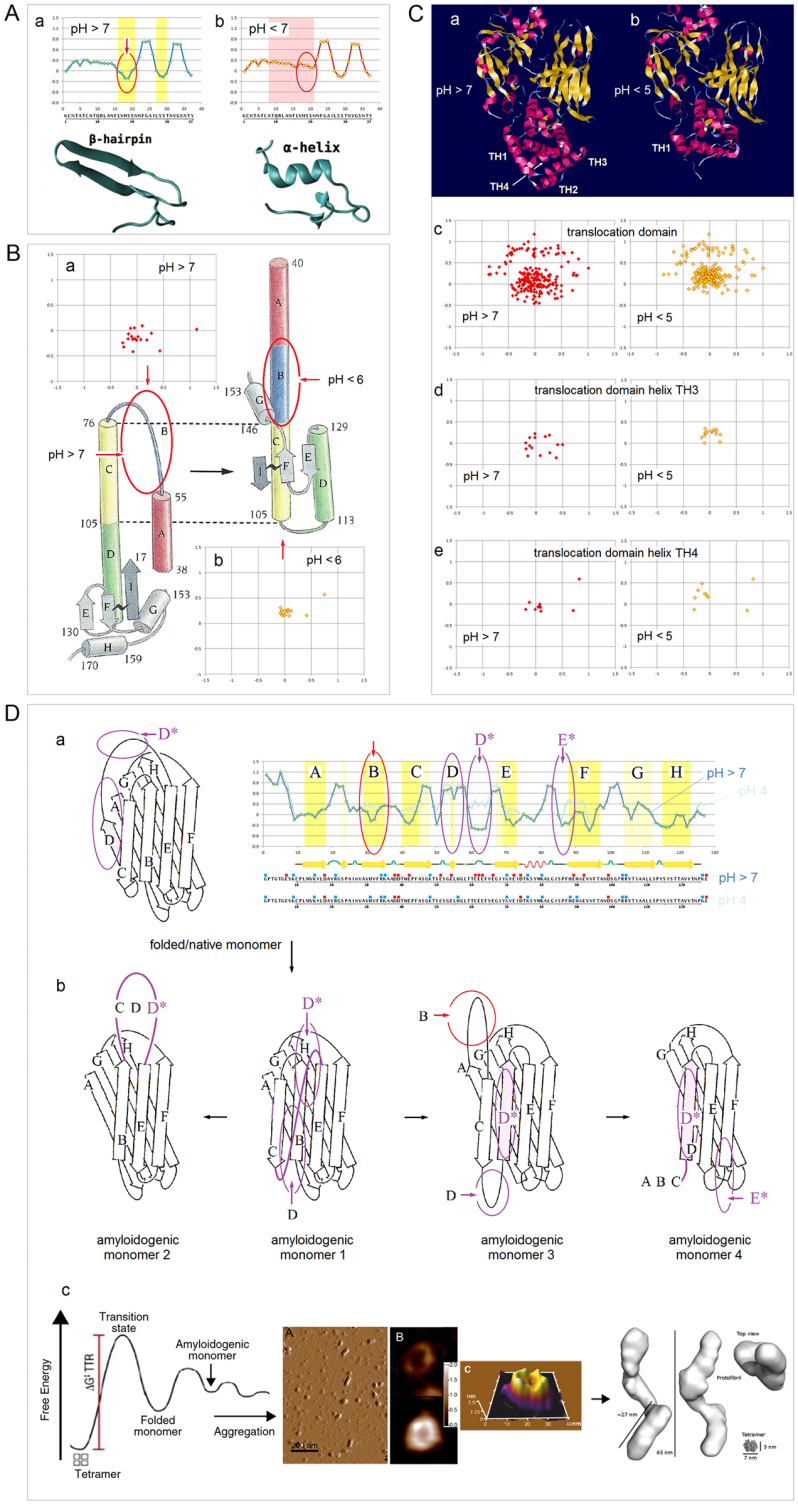
Effect of pH on conformational and H-bonding propensity of the polypeptide backbone. **(A) Conformational equilibria of islet amyloid polypeptide (amylin)** [157]: (a) The *FP*_*i*_ plot of amylin at high pH and the putative β-sheet conformation of the peptide. Assuming σ^His^≡σ^H°^ in Table 1, the segment L16-T30 has β-hairpin potential in the aqueous buffer: L16-N21 $\bar{{\text{FP}}_{\text{i}}}$ = ‒0.0690 (C_5_ strand), N22-I26 $\bar{{\text{FP}}_{\text{i}}}$ = 0.6667 (turn), and L27-T30 $\bar{{\text{FP}}_{\text{i}}}$ = ‒0.0964 (C_5_ strand); (b) The *FP*_*i*_ plot for amylin at low pH and the putative helical conformation of the peptide. Assuming σ^His^≡σ^H+^ in Table 1, the segment T9-N22 has unambiguous α-helical potential in the aqueous buffer, $\bar{{\text{FP}}_{\text{i}}}$ = 0.1746. **(B) Acid-induced loop-to-helix transition in influenza hemagglutinin** [158]. The trimeric glycoprotein hemagglutinin from influenza virus acts as a fusogen at low pH of endocytic vesicles. The activity is contingent on a large-scale structural rearrangement crucial for delivering the viral contents into host cells. The rearrangement involves inter alia conversion of B-loop of hemagglutinin (residues 55–76) into a long α-helix: (a) The *FP*_*i*_ vs. Δ*FP*_*i-1→i+1*_ plot for B-loop at high pH. The segment has a helical *FP*_*i*_ profile but very low $\bar{{\text{FP}}_{\text{i}}}$ and it is accordingly unstructured; (b) The *FP*_*i*_ vs. Δ*FP*_*i-1→i+1*_ plot for B-loop at low pH. The helical data cluster is now shifted into the stable ‘helix’ region, $\bar{{\text{FP}}_{\text{i}}}$ > 0. **(C) Partial unfolding of the translocation-domain helices of the α-pore forming diphteria toxin at low pH** [159]. The toxin invades a cell by crossing the endosome membrane via the process mediated by the C-terminal segment of the all-α domain T (translocation domain) and triggered by the reduced pH in the endosomal lumen: (a) At the standard physiological pH, the toxin is well-structured and the C-terminal segment of the domain T is buried under the helices TH1-TH4, PDB ID 1f0l; (b) At low pH, the TH2, TH3 and TH4 helices become completely disordered, PDB ID 4ow6 [159]. As a result, the C-terminal helices are exposed and can interact with the membrane; (c) The *FP*_*i*_ vs. Δ*FP*_*i-1→i+1*_ plot for the domain T at the neutral pH. The plot shows the characteristic pattern of a soluble multi-helix bundle, cf. Fig 15A(e), where α-helices incorporate ‘C_5_ strand’ segments necessary to stabilize the compact tertiary structure; (d) The *FP*_*i*_ vs. Δ*FP*_*i-1→i+1*_ plot for the domain T at low pH: the folding potential *FP*_*i*_ becomes more positive for the entire domain (the ‘C_5_ strand’ segments are ‘titrated out’ which destabilizes the tertiary structure); (e)-(h) The *FP*_*i*_ vs. Δ*FP*_*i-1→i+1*_ plots for the α-helices TH3 and TH4 at the neutral pH, panels (e) and (g), and at the low pH, panels (f) and (h). In both cases the folding potential shifts from *FP*_*i*_≤0 in panels (e) and (g) (the α-helices which are stable in the interior of a compact structure) to *FP*_*i*_ ≥0.3 in panels (f) and (h) (the α-helices which are unstable except in a highly polarizing environment cf. Fig 7). **(D) Acid-induced unfolding and aggregation of transthyretin** [38,162–172]: (a) The superposed *FP*_*i*_ plots for transthyretin at pH >7 and pH 4, and the DSSP assignments for the native monomer (homotetramer subunit, β sandwich fold). The red and purple outlines mark the strand and turn segments most affected by the reduction of pH: *FP*_*i*_ of the V30-F33 segment (βB strand) shifts from ‘C_5_ strand’ to ‘helix’ propensity, and the two ‘turn’ segments (D-E≡D* and E-F≡E*) are destabilized. In addition, *FP*_*i*_ of βC and βE segments shifts from ‘C_5_ strand’ to ‘C_7eq_ strand’ propensity. These changes destabilize the outer β sheet of transthyretin βC↓βB↑βE↓βF↑; (b) Putative amyloidogenic monomers of transthyretin generated by step-by-step dismantling and rearrangement of transthyretin β sandwich consistent with the destabilizing *FP*_*i*_ shifts and the solid state NMR [162–164], spin-labelling [165,166], immunoreactivity [167] and H/D exchange (HXMS) [168,169] studies; (c) The pathway of acid-induced unfolding and aggregation of transthyretin [38], and the topology of annular octamers [170–172] and protofibrils [169].

Conformational propensity of a backbone segment may be altered by the change in pH. For instance, amylin adopts β-hairpin fold in a neutral or basic solution where the C-terminal segment has the Δ*FP*_*i-1→i+1*_ profile ‘C_5_ strand’-‘turn’-‘C_5_ strand’, but it becomes helical in acidic solution [157] where, due to the protonation of His-18, the entire N-terminal segment acquires ‘helical’ Δ*FP*_*i-1→i+1*_ profile (T9-N22: $\bar{{\text{FP}}_{\text{i}}}$ = 0.1746), Fig 21A. On the other hand, the B-loop of influenza hemagglutinin has a ‘helical’ Δ*FP*_*i-1→i+1*_ profile but very low $\bar{{\text{FP}}_{\text{i}}}$ and is therefore unstructured at pH>7, cf. Fig 21B. At low pH, however, B-loop residues do form a long helix [158] and indeed the *FP*_*i*_ vs. Δ*FP*_*i-1→i+1*_ plot at pH<5 reveals a considerable shift of its ‘helical’ cluster to *FP*_*i*_ > 0.

#### (xi) pH-Driven equilibria: Loss of tertiary structure and reorganization of the folding basin *FB*

In the case of the α-pore forming diphteria toxin, the crucial unfolding of several α-helices of its translocation domain T is triggered by the reduced pH in the endosomal lumen (pH 4.5–5). By attenuating polarization of the polypeptide backbone, low pH may increase mobility of the α-helices of the diphteria toxin and ‘neutralize’ the ‘C_5_ strand’ segments which ought to be incorporated into these α-helices to stabilize the compact tertiary structure of the T domain bundle, cf. sections **a**.(iii) and **b**.(iv). The *FP*_*i*_ vs. Δ*FP*_*i-1→i+1*_ plots in Fig 21C suggest that this is indeed the underlying effect. In particular, the folding potential *FP*_*i*_ of the two helices that completely unfold under the acidic conditions, TH3 and TH4 [159], shifts from *FP*_*i*_≤0 (the α-helices which are stable in the interior of a compact structure) to *FP*_*i*_ >0.3 (the α-helices which are unstable except in a highly polarizing environment), see Fig 21C(d) and 21C(e).

It follows that the shift in backbone density distribution produced by protonation of D^‒^, E^‒^ and H may destabilize compact tertiary structure and trigger the transition into an ‘acid molten globule’ which can in turn initiate fibrillization of a globular protein. The acid-induced unfolding and fibrillization of transthyretin illustrates this point. Based on the *FP*_*i*_ plots, several segments of the polypeptide backbone of transthyretin considerably change conformational and H-bonding propensity at pH 4, see Fig 21D(a): (1) the *FP*_*i*_ profile in the βB region becomes ‘helical’ due to the protonation of His-31, cf. similar ‘strand’→‘helix’ transition in amylin; (2) the pronounced ‘C_5_ strand’ propensity of the βC, βE and βF strands is lost; (3) the high ‘turn’ propensity of the DE loop (D* segment) is lost (the C_5_-like, negative $\bar{{\text{FP}}_{\text{i}}}$ of D* becomes positive and C7_eq_-like); (4) the high ‘turn’ propensity of the EF loop (E* segment) is lost [160,161]. These changes destabilize the outer β sheet of transthyretin and lead to step-by-step unravelling of the β sandwich fold beginning with ‘unmasking’ of the βA strand. Subsequent ‘unmasking’ of the βB strand, or alternatively the extrusion of the βB strand and insertion of the βD* strand in its place, generate three other partially folded monomers as shown in Fig 21D(b). The seemingly contradictory results of the solid state NMR [162–164], spin-labelling [165,166], immunoreactivity [167], and H/D exchange (HXMS) [168,169] investigations can be reconciled by assuming that all four putative aggregation intermediates are competent to assemble into annular octamers [170–172], protofibrils and fibrils, see Fig 21D(c), and the actual course of fibrillization depends on the incubation conditions.

### c. The PMO theory of misfolding and aggregation of pleiomorphic proteins associated with neurodegenerative diseases

Misfolding of several proteins is believed to play a key role in genesis of neurodegenerative diseases including Alzheimer’s amyloid β (Aβ) and tau proteins, Parkinson’s α-synuclein and TSEs’ mammalian prions. These proteins are highly pleiomorphic and readily undergo a series of conformational transitions along the aggregation pathways [173]. Based on the preceding considerations and findings, and taking into account extensive experimental evidence [173–370], we develop here a model of pleiomorphism of these proteins focusing initially on the best-documented case, the polymerization of Aβ.

#### (i) Antiparallel coiled-coil as a model of Aβ dimer structure

**Fig 22:**
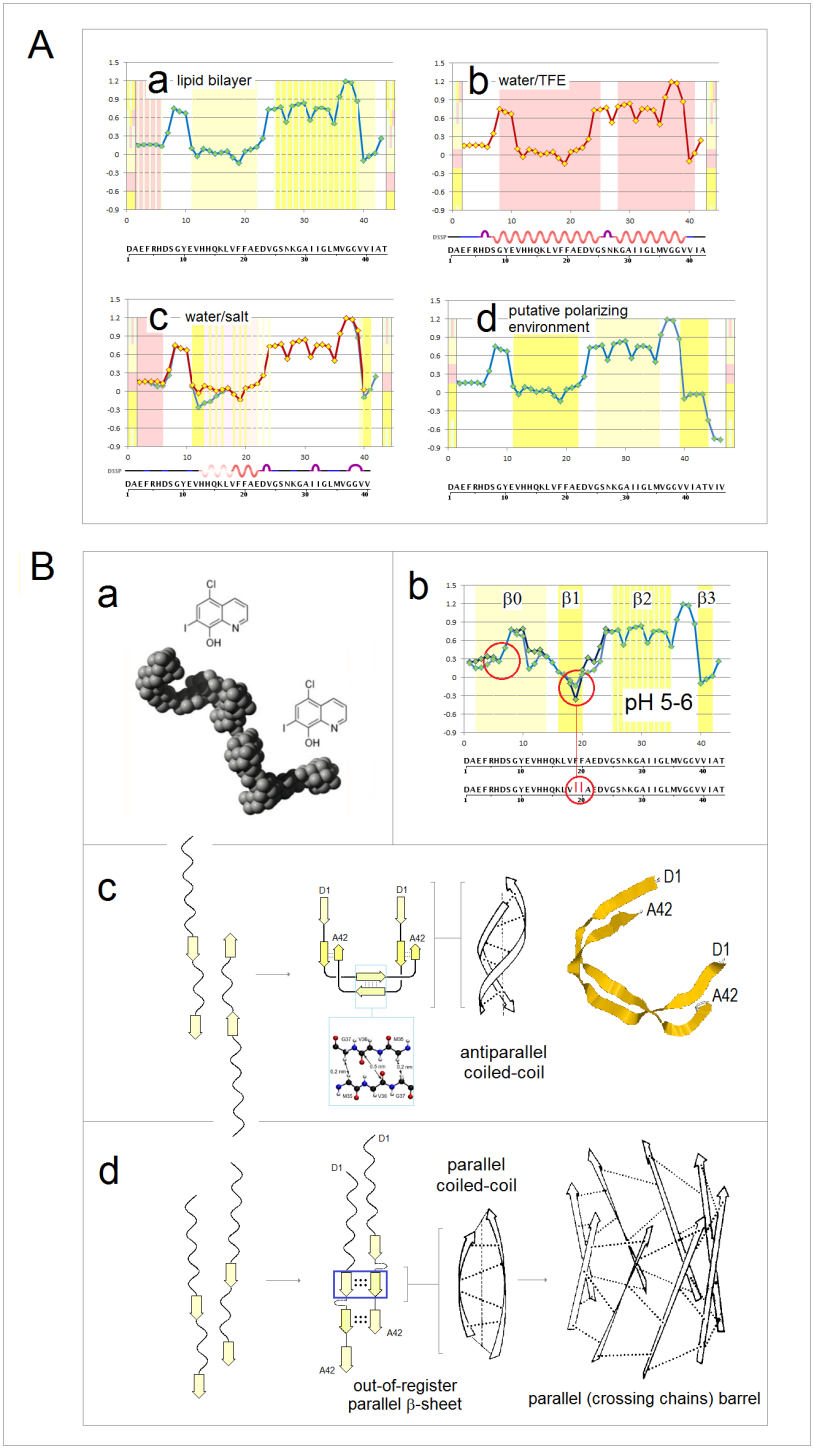
Electronic configuration of the polypeptide backbone and conformational behavior of Aβ proteins. **(A) Folding potential, medium effect and secondary structure of monomeric Aβ**. The *FP*_*i*_ plots for Aβ peptides are juxtaposed with the color-coded bars representing medium effects cf. Fig 7 (note that at pH 7, σ^His^ is average of the H^0^ and H^+^ constants since p*K*_a_ values of Aβ histidines are in the 6.8-7.0 range [174,175]): (a) **Nonpolar environment**: e.g. bilayer membrane/lipid matrix (*FP*_*i*_ plot for Aβ^1–43^). Elements of the E11-E22 and C-terminal segments are expected to adopt the C_7eq_ fold while the array of consecutive ‘turns’ D23-M35 may be stabilized as the ‘C_5_* strand’; in contrast, the E3-D7 segment may adopt a helical conformation. The G25-V39 segment of Aβ^1–40^ bound to DMPC bilayer (multilamella vesicles composed of 1,2-dimyristoyl-sn-glycero-3-phosphocholine, high P/L ratio) is indeed reported to fold into a parallel β structure [176,177]. On the other hand, the E3-D7 residues buried in the CDR loops of the complexes with monoclonal antibodies are found to be helical: PP_II_- (PDB ID’s 2ipu, 3bae) or 3_10_- (PDB ID 4hix) [178]. (b) **Moderately polarizing environment**: e.g. H_2_O/TFE (HFIP) solutions or micelle interfaces (*FP*_*i*_ plot for Aβ^1-42^). The E11-E22 and D23-M35 segments are expected to display α-helix and α*-helix propensities, respectively; the E3-D7 segment is expected to remain disordered since it lacks a well-defined conformational propensity. Several reports confirm that Aβ peptides adopt an all-α fold in the environments of micellar interfaces or aqueous solutions of fluorinated alcohols (see e.g. PDB ID 1iyt) [179–184]. (c) **Polar environment**: e.g. aqueous buffers (red *FP*_*i*_ plot for Aβ^1–40^ at pH 7; cyan *FP*_*i*_ plot for Aβ^1–43^ at pH 8–9). In view of the *FP*_*i*_ patterns, the E11-E22 and D23-M35 segments are expected to remain disordered in a neutral aqueous buffer. However, the E11-E22 segment may sample helical conformations while the CHC (L17-A21), E11-Q15, and C-terminal residues may sample either the C_5_ (pH 8, longer isoforms) or the C_7eq_ conformations. In agreement with these expectations, experimental evidence suggests that Aβ monomers form in aqueous solution collapsed coils [185–188], devoid of secondary structure [189,190], but the evidence of stabilization of residual secondary structure of Aβ in some conditions is also reported (e.g. PDB ID 2lfm and 2otk) [191–194]. (d) **Highly polarizing environment**: e.g. the environment within an amyloid fibril (*FP*_*i*_ plot for Aβ^1-46^). The E11-E22 and D23-M35 segments are now expected to adopt C_5_ and C_7eq_ conformations respectively. The distribution of amyloid-fiber forming capacities, cf. Fig 11C, is consistent with this expectation. **(B) Electronic Configuration of the Polypeptide Backbone and Aggregation of Aβ**. (a) The Aβ^1-42^ dimer obtained by modelling studies based on the SAXS data for the stoichiometric Aβ^1-42^ complexes with 8-hydroxyquinolines [200]; (b) The *FP*_*i*_ plot shows the anticipated ‘strand’ configurations in the Aβ homodimer at pH 5-6 i.e. under the conditions that accelerate fibrillization (light blue curve H^+^/E^−^, blue curve H^+^/E^0^) [203,204]; (c) The antiparallel coiled coil as a model of Aβ dimer structure: the favoured antiparallel dimerization of Aβ in aqueous solutions matches the CHC and C-terminal segments that both adopt either the C_7eq_ or C_5_ conformations. The antiparallel assembly could in addition be stabilized by H-bonding between the segments of consecutive ‘turns’ V24-G37 which may adopt the C_5_* fold in the dimer. The *intermolecular* residue contacts implied by this model are observed in some Aβ aggregates as shown in the insert [201]. By analogy to the two-stranded antiparallel β-sheet of inteins, cf. Fig 20, the antiparallel Aβ dimer is expected to fold into a left-handed coiled-coil, shown in the diagram with a left-handed superhelical twist; (d) The parallel coiled coil as a model of Aβ dimer structure: the favoured parallel dimerization of Aβ in aqueous solutions would match the CHC and ‘turns’ segments, the C_7eq_ and C_5_* folds respectively, in both combinations. This mode of assembly yields out-of-register parallel β-sheets expected to fold with ease into parallel β-barrels. The *intermolecular* residue contacts implied by this model, e.g. F19 vs. I31 or L34, are in fact observed in some Aβ oligomers and aggregates [202]. However, neither parallel dimer nor parallel barrel are likely to form and persist in absence of a stabilizing molecular ‘template’.

The Aβ fold strongly depends on medium and molecular embedding, and the *FP*_*i*_ plots in Fig 22A correctly anticipate this dependence: secondary structure propensities assigned according to Fig 7 are consistent with the available data [174–194]. The ‘helix’ and ‘strand’ segments of Aβ lack the *FP*_*i*_ bias needed to stabilize the α or β structure in water, Fig 22A(c), and indeed the monomeric Aβ exists in water as a collapsed coil devoid of secondary structure. However, the anticipated ‘strand’ configurations of Aβ may be stabilized in an extended intermolecular β sheet i.e. as a result of oligomerization. Since the experimental evidence points to the importance of the simplest oligomer, the Aβ homodimer, in aetiology of neurodegeneration [195–199], we assume that this dimer is the basic building block of polymerization. The SAXS data for the stoichiometric Aβ^1–42^ complexes with 8-hydroxyquinolines suggest that such complexes involve specific extended dimers of Aβ^1–42^ which are at least partially folded [200], Fig 22B(a). The technique does not allow to establish the mode of alignment; we propose below how such extended dimers are aligned by invoking the *FP*-directed molecular recognition, cf. sections **a**.(i) and **b**.(vii) and (ix), and the solid-state NMR data for the off-pathway Aβ oligomers 150±30 kDa [201,202].

Fig 22B(b) shows the ‘strand’ configurations in the homodimer that favor fibrillization [203,204]. The sequence β0(C_7eq_)-β1(C_5_)-β2(C_5_*)-β3(C_5_) is crucial to the *FP*-directed molecular recognition in dimerization and subsequent pleiomorphic behavior of Aβ. Some of the observations consistent with this proposition are the following: (1) the FAD English H6R, Taiwanese D7H and Tottori D7N mutations which promote aggregation [205,206] would stabilize the C7_eq_ conformation of β0 while the A2T and A2V mutations which also influence aggregation [207–209] would stabilize the C_5_ fold; (2) the CHC mutations that stabilize the C_5_ conformation of β1 (e.g. F19I/F20I shown here in the *FP*_*i*_ plot) appear to increase the rate of primary nucleation [210–213], although the evidence is not unequivocal [214], while the A21G mutant (Flemish FAD) does not undergo standard fibrillization [215]; (3) conservative hydrophobic mutations that preserve low permittivity environment required to stabilize the β2(C_5_*) H-bonding, cf. section **a**.(iii), do not seem to affect the initial oligomerization [216]; (4) VPV mutations (including G38V) which considerably increase C_5_ propensity of β3, and increase threefold stability of dimers [217]; (5) C-terminal extensions of the Aβ length that increase C7_eq_/C_5_ propensity of β3 (e.g. in Aβ^1–41^, Aβ^1–42^, Aβ^1–43^ or Aβ^1–46^ cf. the *FP*_*i*_ plots in the panels A(a)-(d), are reported, albeit with some exceptions [218], to facilitate oligomerization [219–228].

Thus, the first step of aggregation of Aβ is coupled folding and binding resulting in formation of the staggered antiparallel dimer, Fig 22B(c). By analogy to the two-stranded antiparallel β-sheet that anchors the horseshoe-like fold of inteins, cf. section **b**.(ix) and Fig 20, the antiparallel Aβ dimer is expected to fold into a left-handed coiled-coil that has in addition a left-handed superhelical twist. The antiparallel assembly is stabilized by matching the CHC and C-terminal ‘C_5_ strand’ segments, and by H-bonding between the segments of consecutive ‘turns’ which may adopt the C_5_* fold. The D1-K16 and E22-K28 segments are not involved in β structure of the homodimer, the first remaining ‘free’, as the unattached ‘C_7eq_’ strand, and the other forming a loop. The *intermolecular* contacts of the C_5_* segments implied by the dimer’s register are observed in some Aβ aggregates as shown in the inserted diagram [201].

To ensure parallel dimerization of Aβ via *FP*-directed molecular recognition, the β1 and β3 segments would have to adopt the C_7eq_ configuration while the β2 segments adopt the C_5_* configuration as shown in Fig 22B(d). This mode of assembly yields out-of-register parallel β-sheets expected to fold with ease into parallel β-barrels. The *intermolecular* residue contacts implied by this model, e.g. F19 vs. I31 or L34, are in fact observed in some Aβ oligomers and aggregates [202]. However, neither parallel dimer nor parallel barrel are likely to form and persist in absence of a stabilizing molecular ‘template’.

#### (ii) A model for *in vitro* dimerization of Aβ peptides: Effects of concentration, small-molecule modulators, and surface/interface support

**Fig 23:**
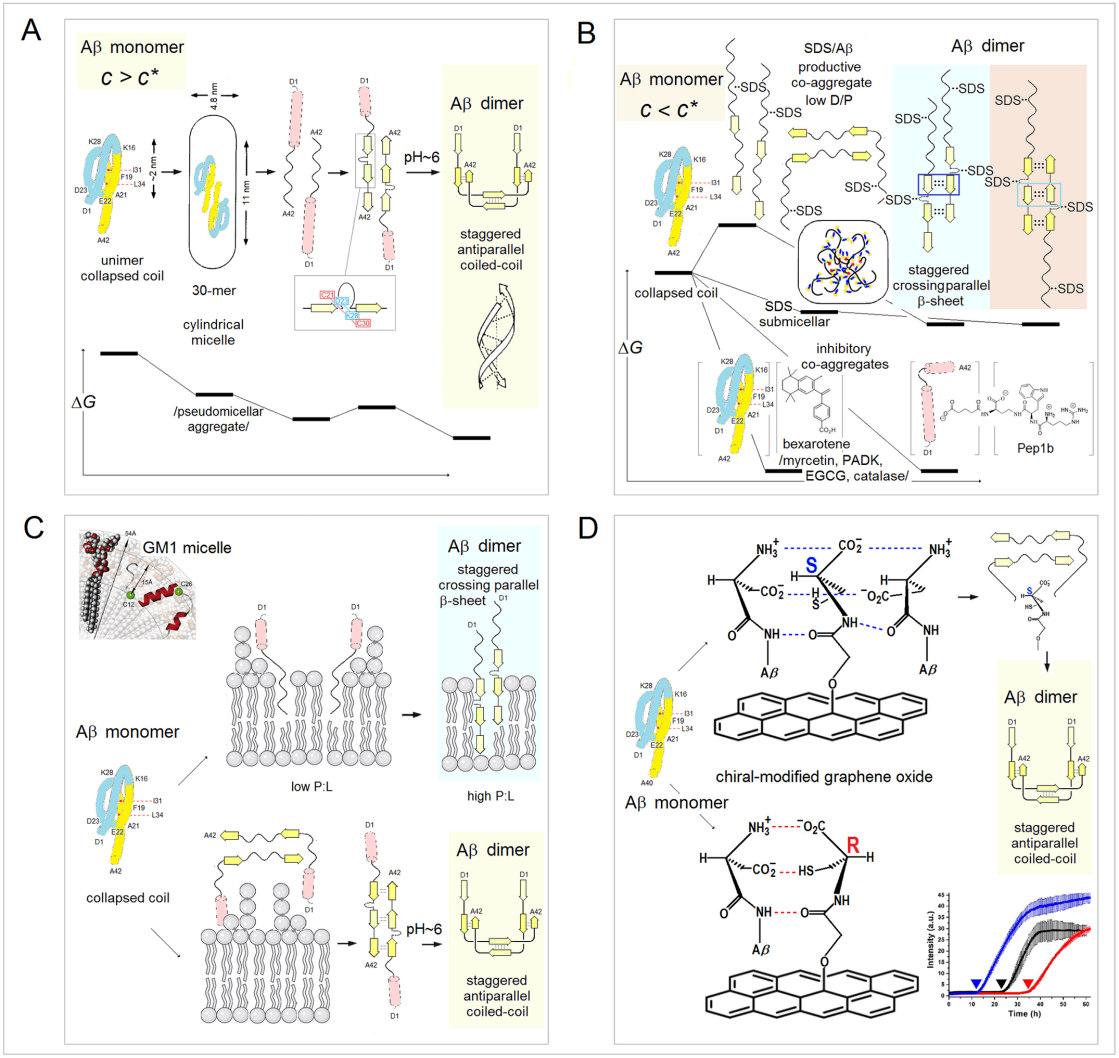
A model for *in vitro* dimerization of Aβ peptides: Effects of concentration, small-molecule modulators, and surface/interface support. Formation of the dimer described in Fig 22B is contingent on the ‘unfolding’ of the collapsed-coil ensemble of Aβ. This transition will be facilitated by transferring the protein from an aqueous solution to a less polar environment which drives the N-terminal half of Aβ into a helical conformation. Such a transfer can be achieved in several ways: **(A)** If Aβ concentration in an aqueous buffer is sufficiently high, c>c*, the protein initially forms micelle-like aggregates [229–231]. The protein is thus buried in a low-permittivity folding basin and its N-terminal half adopts a helical conformation which ‘unwinds’ the collapsed coil. **(B)** If Aβ concentration is too low to generate pseudomicellar aggregates, c<c*, dimerization may be catalysed by the detergents and detergent-like amphipathic co-solutes [236–238]. E.g. SDS at submicellar concentrations is shown to form Aβ co-aggregates [239] in which protein’s N-termini are assembled in the center of the aggregate while the C-terminal chain segments protrude into solution. On the other hand, however, small-molecule co-solutes may also stabilize the collapsed-coil ensemble or the helical conformer of Aβ and thereby prevent dimerization [240–246]. **(C)** Binding of Aβ in a water/micelle or water/lipid membrane interface transfers the protein from an aqueous buffer into a less polar environment. The expected conformational transition is well documented, cf. Fig 22A(a) and 22A(b) [176,177,181–184], and may well contribute to the catalysis of polymerization by membranes and lipid rafts [247–254]. **(D)** (a) The (*S*)-cysteine can bring together two Aβ molecules via three-point binding of Asp-1, in spite of a degree of steric strain in either chelation mode; (b) The (*R*)-cysteine can tightly bind one Aβ molecule in a complex free of strain, but bringing in the second Aβ molecule is impossible due to prohibitive steric hindrance; (c) Thus, the (*S*)-cysteine would promote formation and release of the Aβ↑Aβ↓ dimer and therefore accelerate polymerization (the blue curve in the insert), while the (*R*)-cysteine would immobilize isolated Aβ molecules on the graphene surface and therefore slow down polymerization (the red curve in the insert).

Formation of the dimer described in Fig 22B is contingent on the ‘unfolding’ of the collapsed-coil ensemble of Aβ. This transition will be facilitated by transferring the protein from an aqueous solution to a less polar environment which drives the N-terminal half of Aβ into a helical conformation, cf. Fig 22A(b). Such a transfer can be achieved in several ways, see Fig 23:

1. If Aβ concentration in an aqueous buffer is sufficiently high, c>c*, the protein initially forms micelle-like aggregates, Fig 23A [229–231]. The protein is thus buried in a low-permittivity folding basin *FB* and its N-terminal half adopts helical conformation which ‘unwinds’ the collapsed coil. Experimental evidence suggests that the α-helical Aβ conformer is indeed an obligatory intermediate under these conditions [232,233]. Since the D23-K28 segment forms a side loop in the two-stranded staggered antiparallel β-sheet, stabilization of this loop by a lactam or a disulfide bridge will also facilitate dimerization [234,235].
2. If Aβ concentration is too low to generate pseudomicellar aggregates, c<c*, dimerization of Aβ may be catalysed by the detergents and detergent-like amphipathic co-solutes [236–238]. E.g. SDS at submicellar concentrations is shown to form Aβ co-aggregates [239] in which protein’s N-termini are assembled in the center of the aggregate while the C-terminal chain segments protrude into solution and may form either parallel or, more likely, antiparallel β-sheets, Fig 23B. However, small-molecule co-solutes may also stabilize the collapsed-coil ensemble or the helical conformer of Aβ and thereby prevent dimerization [240–246].
3. Binding of Aβ in a water/micelle or water/lipid membrane interface transfers the protein from an aqueous buffer into a less polar environment, Fig 23C. The expected conformational transition is well documented, cf. Fig 22A(a) and 22A(b) [176,177,181–184], and may well contribute to the catalysis of polymerization by membranes and lipid rafts [247–254].

Binding of Aβ on the cysteine-modified graphene oxide surface either accelerates or slows down fibrillization depending on the chirality of cysteine [40]. The proposed model of Aβ dimerization suggests that this effect can be explained by the difference in how the N-terminal Asp residue (Asp-1) of ‘unfolded’ Aβ (in water/graphene interface) interacts with the (*S*)- and (*R*)-cysteine, marked in blue and red respectively in Fig 23D. The (*S*)-cysteine can bring together two Aβ molecules via three-point binding of Asp-1, in spite of a degree of steric strain in either chelation mode, Fig 23D(a). On the other hand, the (*R*)-cysteine can tightly bind one Aβ molecule in a complex free of strain, but bringing in a second Aβ molecule is impossible due to prohibitive steric hindrance, Fig 23D(b). Thus, the (*S*)-cysteine would promote both formation and release of the Aβ↑Aβ↓ dimer and therefore accelerate polymerization (the blue curve in the insert, Fig 23D(c)), while the (*R*)-cysteine would immobilize isolated Aβ molecules on the graphene surface and therefore slow down polymerization (the red curve in the insert).

#### (iii) Aggregation of Aβ dimers into paranuclei by domain swapping

**Fig 24:**
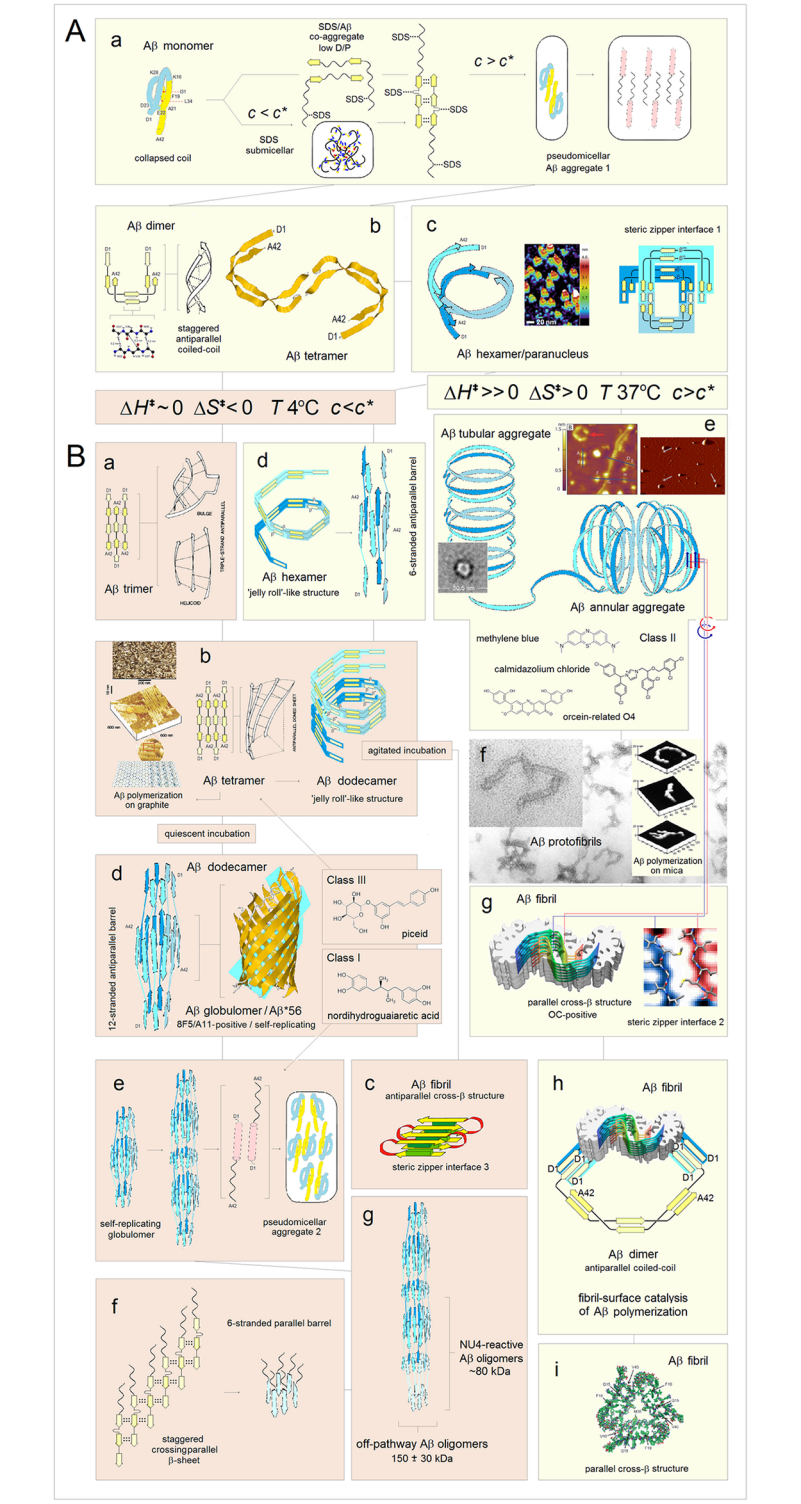
A model for nucleated polymerization of Alzheimer’s Aβ proteins. **(A) Domain swapping: Paranuclei and fibrils comprising parallel β-sheets**. (a) The mechanism of conversion of the Aβ collapsed coils into out-of-register antiparallel dimers via split intein-like mechanism of molecular recognition, (1) at the Aβ concentration greater than the critical micelle concentration *c* > c*, and (2) at the Aβ concentration *c* < c*, in the presence of submicellar concentrations of SDS; (b) The staggered antiparallel Aβ dimer as the left-handed coiled-coil that has in addition left-handed superhelical twist, and the Aβ tetramer obtained via antiparallel association of the ‘free’ N-terminal segments (domain swapping); (c) Further aggregation via domain swapping yields a disk-shaped hexamer—paranucleus [204,264–268]. The circular complex places two dimers on top of each other. The characteristic trapezoid appearance of paranuclei in high-resolution AFM images [266], see the insert, appears to be consistent with the wedge-like shape. The strands stacked on top of each other are parallel; (d) The circular hexamer complex may also be stabilized by the edge-to-edge H-bonding within a ‘jelly-roll’-like structure which may convert into a 6-stranded antiparallel β barrel; (e) The limiting modes of stacking of the paranuclei which yield either tubular (Aβ amyloid pore [271]) or annular aggregates (Aβ nanoglobules [272]). The high-resolution AFM image of the initial stages of aggregation of the paranuclei shows stacking of disk-shaped hexamers and formation of a ring structure (red arrow). Catalysis of fibrillization by the intercalating cations and anions e.g. methylene blue, calmidazolium chloride, orcein-related O4 [276–278] suggests that conformational conversion of the antiparallel sheets β↑β’↓ and β”↑β”‘↓ of the paranuclei into parallel cross-β structure occurs in such stacks; (f) Morphological diversity of polymerization on mica support [42] appears to reflect two modes of paranuclei aggregation, and so does the structure of protofibril ‘on-path’ intermediates of fibrillization in solution [279–282]; (g) The cryoEM-derived structures of Aβ fibrils shows the expected assembly of two protofilaments comprising parallel cross-β sheets and aligned in the antiparallel fashion [283,284]; (h) Mechanism of fibril-surface catalysis of secondary nucleation [43,285,286]: formation of the Aβ dimers is facilitated by the binding of Aβ monomers along the fibril edges via antiparallel interlocking of the N-terminal segments; (i) The trimeric fibril which may be formed by remodeling of the fibril shown in panel (g) [288]. **(B) Edge-to-edge assembly: Off-pathway oligomers, self-replicating non-fibrillar aggregates and fibrils comprising antiparallel β-sheets**. (a) The Aβ trimer obtained by the edge-to-edge assembly. In contrast to the dimer, the trimer cannot fold into a coiled coil [154]; (b) Morphology of polymerization of Aβ on graphite [41] is consistent with the model of edge-to-edge assembly and *FP*-directed molecular recognition, see the text. The edge-to-edge Aβ tetramer cannot fold into a coiled coil either [154] but its polymerization via domain swapping may produce high-order oligomers that retain the staggered antiparallel alignment of strands [261,289–291]. By a combination of twist, bend and rise, such oligomers can fold into ‘jelly-roll’-like cylindrical structures; (c) Reversal of domain swapping and fragmentation within the ‘jelly-roll’-like folds of higher-order oligomers can yield fibrils comprising antiparallel cross-β structure [292,293]; (d) The alternative conversion of the ‘jelly-roll’-like cylinder involves reversal of domain swapping and the edge-to-edge closing of a β barrel. The homotrimer of tetramers can form in this way the 12-stranded antiparallel barrel which we believe represents the structure of the neuropathological Aβ globulomer [258,294] also isolated as the brain Aβ*56 oligomer [262,295,296]; (e) The 12-stranded antiparallel barrel can ‘capture’ Aβ dimers and tetramers via antiparallel interlocking of the ‘free’ N-terminal segments. This is likely the mechanism of self-replication of the off-pathway Aβ oligomers as shown in the diagram [260,297–299]. Note that the low-permittivity environment of the interior of a larger aggregate may promote strand→helix conversion in reversal of the initial stages of dimerization of Aβ [301]; (f) The alternative edge-to-edge aggregation of Aβ, the staggered (out-of-register) parallel assembly; (g) The 12-stranded antiparallel barrel may also ‘capture’ Aβ monomers that form a staggered parallel assembly and fold into 6-stranded out-of-register parallel barrel. This type of aggregation would account for the formation of the off-pathway Aβ oligomers 150±30 kDa [201,202] and the NU4-reactive Aβ oligomer ~80 kDa [300] (30-mer and 18-mer, respectively), and for the solid state NMR evidence of *both antiparallel and parallel intermolecular* contacts in the 150±30 kDa oligomers [201,202].

Several lines of evidence support the notion of independent pathways of oligomerization and fibrillization of Aβ proteins [237,255–262]. The staggered antiparallel structure implies that the initial association of two Aβ dimers can proceed either (1) by domain swapping between twisted coiled-coil conformers i.e. antiparallel interlocking of the ‘free’ N-terminal segments, Fig 24A(b), or (2) by the edge-to-edge assembly of the extended conformers, Fig 24B(b). The difference in conformational behavior of the resulting tetramers gives rise to those independent pathways of polymerization: domain swapping leads to parallel cross-β structure, Fig 24A(g), while edge-to-edge assembly leads to antiperiplanar β barrel and antiparallel cross-β structure, Fig 24B(c) and 24B(d).

The tetramers obtained by domain swapping are stabilised by the increased C_7eq_ propensity of these segments and by the terminal salt-bridges hence the domain-swapping pathway will be favoured at low pH and low salt; these conditions were in fact shown to accelerate fibrillization [202,203,263,269]. Further aggregation via domain swapping yields a disk-shaped hexamer—paranucleus [204,264–268], see Fig 24A(c). The key feature of this circular hexamer is the superposition of two out of the three antiparallel coiled-coil dimers which produces a wedge-like shape and the characteristic trapezoid appearance in the high-resolution AFM images [266], see the insert in Fig 24A(c). The superposition places the C_5_* strands, viz β/β*'* and β*''*/β*‴* strands in the β structure diagram in Fig 24A(c), on top of each other in the parallel alignment so that the paranucleus is stabilized by the ‘parallel steric zipper’ interface. Consequently, a rotation about the strand axis would be sufficient to convert the antiparallel sheets β↑β*'*↓ and β*''*↑β*‴*↓ into parallel sheets β↑β*''*↑ and β*'*↓β*‴*↓. This conformational conversion would encounter a considerable activation energy barrier in spite of the positive entropy of activation: Δ*H*^‡^>>0, Δ*S*^‡^>0 [202,203,269,270].

#### (iv) Aggregation of paranuclei, conversion of antiparallel β structure into parallel cross-β-structure, and fibril-surface catalysis of secondary nucleation of Aβ

There are, given the wedge-like profile of the disk-shaped paranucleus, two limiting modes of self-aggregation of paranuclei that can be stabilized by the ‘steric zipper’ interfaces: the annular stacking—all paranuclei ‘wedge out’ in the same direction, and the tubular stacking—the neighboring paranuclei ‘wedge out’ in opposite directions. These limiting modes of stacking of the paranuclei yield either tubular (Aβ amyloid pore [271]) or annular aggregates (Aβ nanoglobules [272]). The high-resolution AFM image of the initial stages of aggregation of the paranuclei, shown in the central insert in Fig 24A(e), is consistent with this notion: ‘Features A, B, and C indicate Aβ hexamers, and D, E, and F indicate Aβ dodecamers consisting of two stacked hexamers’, and the red arrow points to an annular aggregate [273]. The morphology of Aβ polymerization on mica [42], where the paranuclei would be held on the hydrophilic surface via backbone H-bonding i.e. bound upright, is also consistent with the two modes of paranuclei stacking: the aggregates form either large ring structures or elongated bar structures, see the inserts in Fig 24A(f).

Formation of a stack facilitates conformational conversion of the antiparallel sheets β↑β*'*↓ and β*''*↑β*‴*↓ of the paranuclei into parallel cross-β structure [272] which is stabilized within the pre-organized architecture of the aggregates [274,275]. This process is also facilitated by the intercalation of the aromatic cations or anions e.g. methylene blue [276], calmidazolium chloride [277] or orcein-related O4 [278], see Fig 24A(e), and the relatively low H-bonding propensity of the ‘C_5_* strands’ i.e. the least polarized backbone segments.

Morphological diversity of the protofibril ‘on-path’ intermediates of fibrillization in solution [279–281] suggests that both paths of paranuclei assembly are also followed in the intermediate stages of polymerization [282], Fig 24A(f). However, the annular aggregates of paranuclei might more readily undergo the conformational conversion. Such a conversion would yield fibrils consisting of two protofilaments that comprise parallel cross-β sheets in antiparallel alignment and this arrangement is indeed observed in the cryoEM-derived structures of Aβ fibrils [283,284], Fig 24A(g). These fibrils can catalyze formation of the staggered antiparallel dimers by ‘unwinding’ collapsed-coil Aβ monomers via antiparallel interlocking of the N-terminal segments (domain swapping) along the fibril’s edges and by ensuring monomers’ proximity [43,285,286], Fig 24A(h). Lastly, folding of the cross-β sheets shown in Fig 24A(g) into β arcades may initiate separation of the two protofilaments into monomeric fibrils [287], and remodeling of those fibrils into the dimeric and trimeric fibrils [288], Fig 24A(i).

#### (v) Edge-to-edge assembly: Off-pathway oligomers, self-replicating non-fibrillar aggregates and fibrils comprising antiparallel β-sheets

The alternative pathway of aggregation of Aβ proteins [236–238,255–264,289–301] begins, we propose, as the edge-to-edge complexation of extended dimers and monomers to form staggered antiparallel trimers, Fig 24B(a) (the trimer previously identified as a highly toxic oligomer [259] may have this structure), or tetramers, Fig 24B(b). The morphology of polymerization of Aβ on graphite results from this type of assembly: the extended Aβ chains are held on graphite by the sidechains’ interactions with the hydrophobic surface and consequently assemble edge-to-edge via backbone H-bonding. The long antiparallel monolayer stacks of Aβ are ~19 nm wide [42] i.e. the chains are staggered (extended peptide is ~15 nm long). Thus, these stacks can catalyze alongside polymerization via domain swapping; this could be the origin of the characteristic clusters of parallel stacks on graphite, see the inserts in Fig 24B(b).

The oligomers obtained by the edge-to-edge assembly cannot fold into coiled coils [154] but further polymerization via domain swapping may produce high-order oligomers that retain the staggered antiparallel alignment of strands [261,289–291]. By a combination of twist, bend and rise, such oligomers can fold into ‘jelly-roll’-like cylindrical structures. Fragmentation within the cylindrical folds of higher-order oligomers can yield fibrils comprising antiparallel cross-β structure [292,293], Fig 24B(c). The alternative conversion of the ‘jelly-roll’-like cylinder may involve the edge-to-edge closing of a β barrel and a concomitant reversal of domain swapping. The homotrimer of tetramers can form in this way the 12-stranded antiparallel barrel which we believe represents the structure of the neuropathological Aβ globulomer [258,294], also isolated as the brain Aβ*56 oligomer [262,295,296], Fig 24B(d). Class III assembly modulators apparently promote the dissociation of such barrels while Class I modulators promote the formation of pseudomicellar aggregates [276].

The proposed structure suggests a mechanism of self-replication of the off-pathway oligomers [260,297–299]. The N-terminal segments of Aβ, which are protruding from the 12-stranded antiparallel barrel, are not expected to form stable in-register 6-stranded parallel barrels. Thus, these N-terminal segments can ‘capture’ the staggered antiparallel Aβ dimers or tetramers via domain swapping and catalyze in this way the assembly of the identical 12-stranded barrel, Fig 24B(e).

In principle, the edge-to-edge aggregation of Aβ may also result in the staggered (out-of-register) parallel assembly, Fig 24B(f). This type of β structure will have a tendency to form a parallel β barrel, cf. Fig 22B(d), as long as it is stabilized e.g. by the antiparallel barrel serving as a molecular ‘template’ as shown in Fig 24B(g). Here, the N-terminal segments on one side of the 12-stranded barrel bind just 6 Aβ monomers which can then form stable 6-stranded out-of-register parallel barrel. This type of aggregation would account for the formation of the off-pathway Aβ oligomers 150±30 kDa [201,202] and the NU4-reactive Aβ oligomer ~80 kDa [300] (30-mer and 18-mer, respectively), and for the presence of *both antiparallel and parallel intermolecular* residue contacts in the 150±30 kDa oligomers [201,202]. Note that the low-permittivity environment of the interior of such large aggregates may promote strand→helix conversion in a reversal of the initial stages of dimerization of Aβ [301].

#### (vi) Morphology of Aβ aggregation on the NIBC monolayers on gold: Combined effects of cysteine chirality and wedge-like shape of Aβ paranuclei

**Fig 25:**
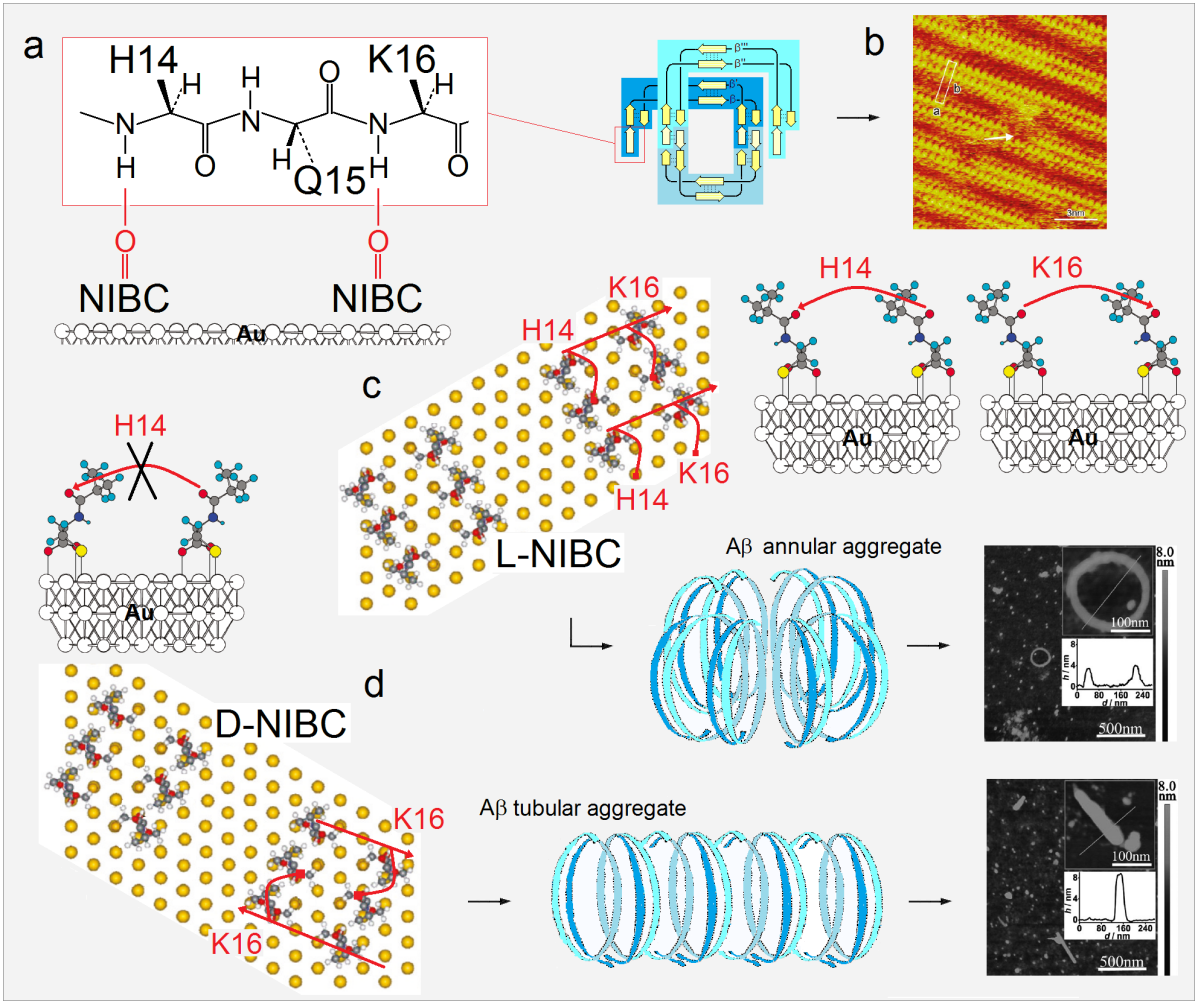
Morphology of Aβ aggregation on the NIBC monolayers on gold: The combined effects of cysteine chirality and wedge-like shape of Aβ paranuclei. **(a)** The putative complex of the Aβ paranucleus on the monolayer surface involves backbone H-bonding of the extended H14-K16 segment to the isobutyryl carbonyls of N-isobutyrylcysteines [41]. The positively charged side chains of His-14 and Lys-16 extend in the same direction. **(b)** The topology of the self-assembled cysteine monolayer on gold [302]: The monolayer comprises pairs of long files of N-isobutyrylcysteines. **(c)** A model of the surface of *L*-NIBC monolayer [302], H14 and K16 side chain interactions with the *L*-NIBC isobutyryl carbonyls, and the putative tight packing of two Aβ paranuclei. The binding of the H14 side chain to the isobutyryl C = O of the neighbouring row of cysteines is unimpeded; the K16 side chain encounters an impediment but its length and flexibility make the binding possible. Thus, the tight packing of paranuclei is achieved via parallel alignment of the H14-K16 segments which directs all the complexed paranuclei to ‘wedge out’ in one direction i.e. promotes annular stacking and formation of large ring structures. **(d)** A model of the surface of *D*-NIBC monolayer [302], H14 and K16 side chain interactions with the *D*-NIBC isobutyryl carbonyls, and the putative tight packing of two Aβ paranuclei. Here the H14 side chain cannot bind to the isobutyryl C = O of the neighbouring row of cysteines; it encounters an impediment and lacks flexibility and length needed to overcome the hindrance. Thus, the tight packing on this surface is achieved via antiparallel alignment of the H14-K16 segments which directs the neighbouring paranuclei to ‘wedge out’ in opposite directions and thereby promotes tubular stacking and formation of elongated bar structures.

The notion of the wedge-shape control of aggregation morphology may explain the effect of chirality on the assembly of Aβ(1–40) on the self-assembled monolayers of *R*(*S*)-N-isobutyrylcysteine (NIBC) on gold [41]. By analogy to Aβ polymerization on mica, section **b**.(iv) and Fig 24A(f), aggregation of Aβ(1–40) on NIBC monolayers produces large ring structures and elongated bar structures. However, the course of aggregation depends on the configuration of the amino acid: large rings form on *L*-NIBC monolayers, and elongated bars form on *D*-NIBC monolayers, see the inserts in Fig 25. Binding of the H14-K16 segment of Aβ to the cysteine monolayer, Fig 25(a), was shown to be crucial to observe this effect [41]. Given that the NIBC monolayers comprise pairs of long files of N-isobutyrylcysteines [302], Fig 25(b), we assume that the putative complex of the Aβ paranucleus on the monolayer surface involves backbone H-bonding of the extended H14-K16 segment to the isobutyryl carbonyls of one file of N-isobutyrylcysteines in the pair (i.e. paranucleus is bound upright). The positively charged side chains of His-14 and Lys-16 extend on the same side of the strand and can stabilize the complex by binding to the isobutyryl carbonyls of the second file of N-isobutyrylcysteines in the pair. On the surface of *L*-NIBC monolayer [302], binding of the H14 side chain to the isobutyryl C = O of the neighbouring row of cysteines is unimpeded; the K16 side chain encounters an impediment but its length and flexibility make the binding possible, see Fig 25(c). Thus, the tight packing of paranuclei is achieved via parallel alignment of the H14-K16 segments which directs all the complexed paranuclei to ‘wedge out’ in one direction and promotes annular stacking and formation of large ring structures. In contrast, on the surface of *D*-NIBC monolayer [302], H14 side chain cannot bind to the isobutyryl C = O of the neighbouring row of cysteines: it encounters an impediment and lacks the flexibility and length needed to overcome the hindrance, Fig 25(d). Thus, the tight packing on this surface is achieved via antiparallel alignment of the H14-K16 segments which directs the neighbouring paranuclei to ‘wedge out’ in opposite directions and thereby promotes tubular stacking and formation of elongated bar structures.

#### (vii) Fibrillization of tau protein and α-synuclein via antiparallel coiled-coil pathway

**Fig 26:**
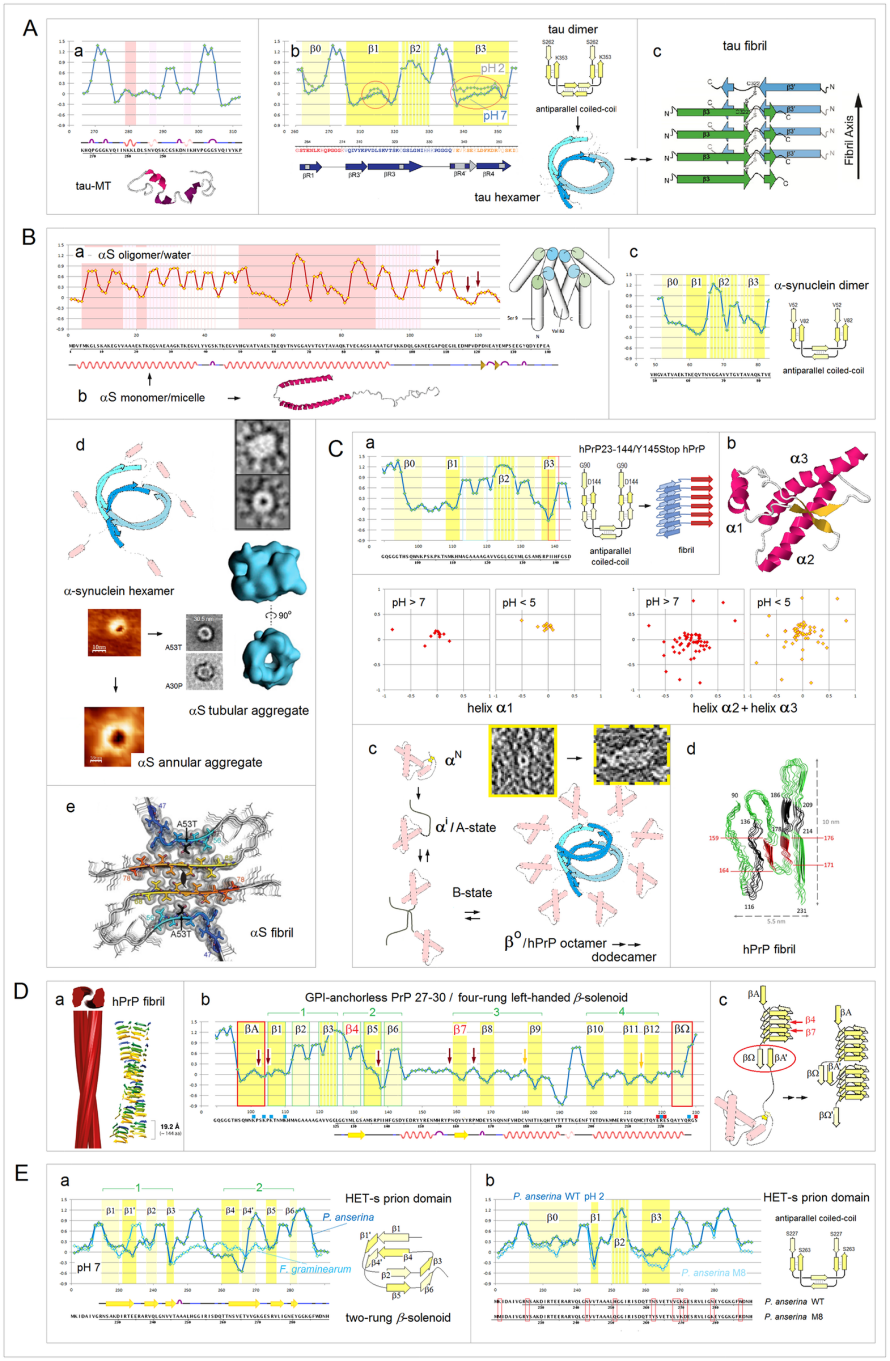
Electronic configuration of the polypeptide backbone and molecular recognition in formation of β structure: Mechanism of aggregation of tau, α-synuclein, human prion hPrP and HET-s PFD. **(A) Tau proteins**: (a) The *FP*_*i*_ plot and secondary structure of the 46-residue fragment of Tau(267–312) bound to microtubules PDB ID 2mz7 [307]; (b) The *FP*_*i*_ profile of the repeat domain construct K19 (R1-R3R4) at pH 7 is similar to the *FP*_*i*_ profile of Aβ at pH 5. The *FP*_*i*_-based assignment of secondary structure is consistent with the solid-state NMR data on the K19 amyloid fibrils [310]. Note that at pH 5 the V337-E342 segment (VEVKSE) has α-helix propensity rather than C_5_ propensity required to stabilize the coiled-coil dimer, and K19 actually does not aggregate at low pH [310]. Nucleated polymerization, assumed to be the main pathway of fibrillization of tau [311–314], may involve disk-shaped hexameric intermediate analogical to the paranucleus of Aβ; (c) The annular assembly of the disk-shaped paranuclei and the subsequent conformational conversion (antiparallel→parallel β-structure) would yield fibrils consisting of two protofilaments which comprise parallel cross-β sheets in the antiparallel alignment. The solid-state NMR structure of β-sheet core of tau paired helical filaments confirms the expected arrangement [315]. **(B) α-Synuclein**: (a) The *FP*_*i*_ plot shows secondary structure assignment for a putative tetramer (colour shading reflects helix propensity based on solution NMR data) [318,319], but the existence of such a tetramer is questioned [320,321]; (b) The micelle-bound monomer is helical PDB ID 1xq8 [322,323]; (c) The *FP*_*i*_ profile of the ~35–40 residue-long amyloidogenic region of human α-synuclein which incorporates the NAC sequence essential to fibrillization of this protein. The pattern of backbone polarization corresponds to the C_7eq_-C_5_-C_5_*-C_5_ sequence of the anticipated ‘strand’ configurations. Thus, like Aβ and tau, α-synuclein is expected to form the antiparallel, out-of-register, coiled-coil dimer shown in the diagram; (d) The tubular (amyloid pore) [271,324,325] and annular (cf. the insert, diameter ~ 120 nm) [324,326] aggregates of α-synuclein may be formed by the stacking of the wedge-like disk-shaped hexamers analogical to the paranuclei of Aβ. Persistent oligomers of MW ~80 kDa and circular appearance (cf. the insert, diameter ~10 nm) are indeed observed during the polymerization in the presence of heme [326]; (e) Conformational conversion (antiparallel→parallel β structure) within the annular stacks of the paranucleus-like hexamers would yield fibrils consisting of two protofilaments which comprise parallel cross-β sheets in the antiparallel alignment. A recently proposed model of α-synuclein protofibril is consistent with this expectation [332]. **(C) Prion proteins (PrP), conversion to parallel cross-β structure**: (a) The *FP*_*i*_ plot for the recombinant polypeptide hPrP23-144 (a model for Y145Stop variant of human prion, the mutation eliminates the entire α-helical region of hPrP) which undergoes a spontaneous conversion from a monomeric disordered state to the in-register parallel cross-β fibrillar form [335–337]. Ignoring the palindromic segment, see the text, the anticipated β structure configuration of the amyloidogenic region is the sequence C_7eq_-C_5_-C_5_*-C_5_ (β0-β1-β2-β3) by analogy to Aβ, tau, and α-synuclein. Thus, this region may also form the antiparallel, out-of-register, coiled-coil dimers which assemble via domain swapping into superhelical oligomers and yield fibrils comprising sheets of parallel cross-β structure; (b) The *FP*_*i*_ vs. Δ*FP*_*i-1→i+1*_ plots for the native helices of hPrP at high and low pH. The plots suggest that the helix α1 is destabilized by reduction of pH while the helices α2 and α3 are becoming stable as the compact structure unravels at low pH; (c) The putative pathway of assembly and structure of the octameric paranucleus obtained at low pH [338–348]. The insert shows hPrP aggregates [349] which could be formed by tubular stacking of such octamers or similar higher-order oligomers; (d) The single-sheet parallel in-register cross-β structure of PrP [350,351]. **(D) Prion proteins (PrP), conversion to β-solenoid fibrils**. (a) The structure of fibrils of GPI-anchorless PrP 27–30 [352,353]. The protofilaments are formed by stacking four-rung left-handed solenoids that include nearly entire length of the protein; (b) The *FP*_*i*_ plot for hPrP (omitting most of the N-terminal domain) and the proposed four-rung solenoid structure. The assignment of the strands marked by the green outlines follows the two-rung model [354] proposed as a revision of the original model of the left-handed β helix [355]; the strands β7 through β12 are assigned based on the *FP*_*i*_ minima indicating the ‘C_5_ strand’ propensity. The proposed structure places Cys179 and Cys214 in register (orange arrows) and all Pro residues (brown arrows) in turns and loops. This structure is also consistent with the anticipated β structure configuration of the amyloidogenic region which can be described as the sequence of two ‘asymmetrically’ complementary arrays of alternant incipient strands: C_5_-C_7eq_-C_5_* followed by C_7eq_-C_5_-C_7eq_ (β1-β2-β3 followed by β4-β5-β6 i.e. including the palindromic segment); (c) The model of self-propagation of the infectious prion: the head-to-tail domain swapping between the native conformer and the β solenoid conformer that involves interlocking of the βΑ and βΩ strands (marked by red outlines in *FP*_*i*_ plot) via assembly of the parallel β sheet C_7eq_↓C_5_↓. **(E) HET-s prion forming domain**. (a) The superposed *FP*_*i*_ plots for HET-s prion forming domains from *P*. *anserina* and *F*. *graminearum*, and the two-rung left-handed β solenoid fold that the domain from *P*. *anserina* adopts in amyloid fibrils [356–358]. The anticipated β structure configuration of the *P*. *anserina* domain is the sequence of two ‘asymmetrically’ complementary arrays of alternant incipient strands: C_7eq_-C_5_-C_7eq_-C_5_ followed by C_5_-C_7eq_-C_5_-C_7eq_ (β1-β1'-β2-β3 followed by β4-β4'-β5-β6); (b) The superposed *FP*_*i*_ plots for the HET-s prion forming domain from *P*. *anserina* at low pH which induces formation of a generic amyloid fold [363–367], and the M8 variant of this domain that forms toxic amyloids comprising antiparallel β structure [368–370].

Tau is a highly soluble, intrinsically disordered protein [303,304]. Its sequence includes the ‘repeat domain’ which binds to lipid membrane surfaces [305,306] and microtubules [307,308] via short helices, Fig 26A(a), folds into β-hairpins upon binding to β-wrapin TP4 [309], and forms amyloid fibrils. The *FP*_*i*_ profile of the ‘repeat domain’ construct K19 (R1-R3R4) at pH 7 shows Aβ-like pattern of backbone polarization which corresponds to the sequence C_7eq_-C_5_-C_5_*-C_5_ of anticipated ‘strand’ configurations, Fig 26A(b); the *FP*_*i*_-based assignment of secondary structure is consistent with the solid-state NMR data on the K19 amyloid fibrils [310]. The *FP*_*i*_ plot also shows that the ‘C_5_ strand’ propensity of the V337-E342 segment (VEVKSE) is lost at pH 2, and indeed K19 does not forms fibrils at low pH [310]. Thus, nucleated polymerization, assumed to be the main pathway of fibrillization of tau [311–314], may involve the staggered antiparallel dimers and disk-shaped hexameric intermediate analogical to the paranucleus of Aβ. The annular assembly of the disk-shaped paranuclei and the subsequent conformational conversion (antiparallel→parallel β-structure) would yield fibrils consisting of two protofilaments which comprise parallel cross-β sheets in the antiparallel alignment. The solid-state NMR structure of β-sheet core of tau paired helical filaments confirms the expected arrangement, Fig 26A(c) [315]; fracture, reassembly and growth of those fibrils may be the source of polymorphism [316].

α-Synuclein is a highly pleiomorphic protein as well [317–323], Fig 26B. The *FP*_*i*_ plot for the ~35-40 residue-long amyloidogenic region of human α-synuclein which incorporates the NAC sequence essential to fibrillization of this protein, is shown in Fig 26B(c). Again, the pattern of backbone polarization corresponds to the familiar sequence of the anticipated ‘strand’ configurations C_7eq_-C_5_-C_5_*-C_5_. Thus, like Aβ and tau, α-synuclein is expected to form the antiparallel, out-of-register, coiled-coil dimer. The tubular (amyloid pore) [271,324,325] and annular (cf. the insert, diameter ~120 nm) [324,326] aggregates of α-synuclein may be formed by the stacking of the wedge-like disk-shaped hexamers analogical to the paranuclei of Aβ, Fig 26B(d). Persistent oligomers of the expected MW ~80 kDa and circular appearance (cf. the insert in Fig 26B(d), diameter ~10 nm) are indeed observed during the polymerization in the presence of heme [326]. On the other hand, the available evidence also points to the existence of independent alternative pathways of aggregation and antiparallel β-structure of oligomers [327–331]. The annular assembly of the disk-shaped paranucleus-like hexamers and the subsequent conformational conversion (antiparallel→parallel β-structure) would yield fibrils consisting of two protofilaments which comprise parallel cross-β sheets in the antiparallel alignment. A recently proposed model of α-synuclein protofibril is consistent with this expectation [332], Fig 26B(e); the β arcade fold of each protofilament may render the fibril prone to dissociation and remodelling [333,334].

#### (viii) Fibrillization of prions via the antiparallel coiled-coil pathway

Fibrillization of the disordered amyloidogenic region of mammalian prions appears to be controlled by the C-terminal well-structured half of the molecule. This part of the molecule is altogether missing in the recombinant polypeptide hPrP23-144 which corresponds to the human PrP Y145Stop variant (the mutation eliminates the entire α-helical region of hPrP), and hPrP23-144 undergoes a spontaneous conversion from the monomeric disordered state to a parallel, in-register, cross-β fibrillar form. The C-terminal I138-F141 segment (red outline in the *FP*_*i*_ plot in Fig 26C(a)), is essential to this conversion but the palindromic M113-A120 segment (blue outline) is not [335–337]. Thus, the anticipated β structure configuration of the amyloidogenic region can be described by the C_7eq_-C_5_-C_5_*-C_5_ sequence of ‘strands’ by analogy to Aβ, tau, and α-synuclein, see the β0-β1-β2-β3 strands in Fig 26C(a). This pattern suggests that hPrP23-144 also forms the antiparallel coiled-coil dimers which assemble via domain swapping into superhelical paranuclei and yield fibrils comprising sheets of parallel cross-β structure.

In the full-length molecule, the same mode of folding can be induced by reduction of pH. The *FP*_*i*_ vs. Δ*FP*_*i-1→i+1*_ plots for the native helices of hPrP at high and low pH suggest that the helix α1 is destabilized by reduction of pH while the helices α2 and α3 are becoming stable as the compact structure unravels at low pH. Disruption of the native β-sheet and unfolding of the helix α1 ‘unmasks’ the C_7eq_-C_5_-C_5_*-C_5_ sequence, see the α^i^/A-state in Fig 26C(c). Thus, at low pH, hPrP also forms the antiparallel coiled-coil dimers (B-state) which assemble via domain swapping into superhelical paranuclei like the octamer (β°) shown in Fig 26C(c) [338–348]. The insert shows hPrP aggregates [349] formed, we believe, by tubular stacking of such octamers or similar higher-order oligomers. The stacks of superhelical paranuclei may convert to fibrils comprising two antiparallel sheets of parallel cross-β structure, and the dissociation of such fibrils, combined with conversion of the helices α2 and α3 into extended strands, eventually yields the single-sheet parallel cross-β structure [350,351], Fig 26C(d).

#### (ix) Fibrillization of prions via the β solenoid pathway

The fibril of GPI-anchorless PrP 27-30 comprises a pair of protofilaments which are formed by stacking four-rung left-handed solenoids that include nearly entire length of the protein [352,353], Fig 26D(a). Thus, the alternative direction of folding of hPrP involves realignment of the native two-stranded β-sheet, and refolding of the three C-terminal helices into a β solenoid while the disulfide bond is intact. The *FP*_*i*_ plot for hPrP (omitting most of the N-terminal domain) suggests that there is indeed the second way to describe the anticipated β structure configuration of the amyloidogenic region, Fig 26D(b). The strands marked by the green outlines in the plot: {β1-β2-β3} followed by {β4-β5-β6} (i.e. including the palindromic segment) form the sequence of two ‘asymmetrically’ complementary arrays of alternant incipient strands: C_5_-C_7eq_-C_5_* followed by C_7eq_-C_5_-C_7eq_. Such a sequence is compatible with the intramolecular parallel rather than antiparallel assembly of β structure, cf. section **a**.(i) and Figs 2 and 5, and is expected to fold into a two-rung β solenoid. The assignment of the strands marked by the green outlines actually follows the two-rung model [354] proposed as a revision of the original model of the left-handed β helix [355]. The strands β7 through β12 (the ‘C_5_ strand’ segments in the *FP*_*i*_ plot) complete our assignment of the four-rung β solenoid structure which places Cys179 and Cys214 (orange arrows) in register, and all Pro residues (brown arrows) in turns and loops. The assignment implies that the strands β4 and β7 which form the antiparallel β sheet C_7eq_↑C_5_↓ in the native structure, do indeed realign into the more stable parallel β sheet C_7eq_↑C_5_↑ in the solenoid. The two strands marked by the red outlines, βΑ and βΩ, have the C_7eq_ and C_5_ configurations. The head-to-tail domain swapping between the native conformer and the β solenoid conformer that produces parallel β sheet βΑ(C_7eq_)↓βΩ(C_5_)↓ (*FP*-directed molecular recognition), may underlie self-propagation of infectious strains of mammalian prions, see Fig 26D(c).

#### (x) Fibrillization of HET-s prion forming domains via β solenoid and antiparallel coiled-coil pathways

The HET-s prion forming domain (PDF) from *P*. *anserina* adopts the two-rung left-handed β solenoid fold in amyloid fibrils [356–358], Fig 26E(a). The *FP*_*i*_ plot at pH 7 confirms that the strands {β1-β1'-β2-β3} and {β4-β4'-β5-β6} of this fold constitute the sequence of two ‘asymmetrically’ complementary arrays of the alternant incipient strands: C_7eq_-C_5_-C_7eq_-C_5_ followed by C_5_-C_7eq_-C_5_-C_7eq_. As was argued previously, such a sequence is compatible with the intramolecular parallel rather than antiparallel assembly of β structure, and is expected to fold into a two-rung β solenoid as long as such a solenoid fold is supported by the outer side chains’ ladders and interior side chains’ packing [359–362]. The PFD domain from *F*. *graminearum* adopts the same fold in spite of limited sequence identity [362] and the *FP*_*i*_ plot reveals similar pattern of the incipient β structure propensity with one exception of the strand β4.

In contrast, low pH induces formation of a generic amyloid fold of the *P*. *anserina* domain [363–367] and the M8 variant of this domain forms toxic amyloids comprising antiparallel β structure [368–370]. The *FP*_*i*_ plots in Fig 26E(b) suggest that the wt PFD loses the ‘asymmetric’ complementarity of the ‘strand’ sequence, while its variant M8 loses both ‘asymmetric’ complementarity and the asparagine (Asn) ladders [359–362]. Rather than fold into a solenoid, the protonated or modified HET-s PFD is likely to behave like the C_7eq_-C_5_-C_5_*-C_5_ sequence and polymerize via aggregation of the antiparallel dimers [363–370].

## Concluding remarks

The qualitative PMO theory of organic structure and reactivity provides simplified models which, inevitably, sacrifice some aspects of reality. The folding potential function, *FP*_*i*_, that we employed in this study is designed to capture the effect of distribution of charge density on the conformational and H-bonding propensity of the polypeptide backbone; it does not carry any information about the constraints introduced e.g. by the hydrophilic residues or by proline, and obviously does not take into account any side chain-side chain interactions. Indeed, on several occasions in the preceding discussion, it was clearly useful to complement the *FP*_*i*_ plots by showing distribution of the ionized side chains and potential ‘steric zipper’ segments or the presence of proline and cysteine residues in the sequence. However, simplification may also underlie the strength of the PMO theory models. And so, surprisingly, the *FP*_*i*_ and *FP*_*i*_ vs.Δ*FP*_*i-1→i+1*_ plots are found to identify basic features of secondary and tertiary structure, establish sequence correlates of metamorphic and pH-driven equilibria, relate binding affinities and folding rate constants to secondary-structure preferences, *and manifest common backbone polarization patterns in amyloidogenic regions* of Alzheimer’s Aβ and tau, Parkinson’s α-synuclein and TSEs’ prions. Thus, by stripping away the side chain-side chain interactions, we discover that the main chain H-bonding and hyperconjugation, described in terms of two-electron stabilizing interactions of the filled and vacant molecular orbitals [7–15], make significant and sometimes decisive contributions to the stability of both native and transition states. The new vocabulary and grammar of encoding the 3D structure of proteins which emerge amidst these findings readily define hitherto elusive pathways of misfolding and aggregation associated with brain proteinopathies. One cannot account, it appears, for a wide range of folding phenomena without taking into consideration the stereoelectronic effects such as the generalized anomeric effect and the homohyperconjugation of peptide linkages [16–22,28–33,51–58]. This conclusion is in line with the arguments of backbone-based theory of protein folding [2], and particularly concerns conformational behaviour of highly pleiomorphic proteins associated with neurodegenerative diseases.

## Supporting information

S1 Appendix Folding template and three-dimensional structure of soluble globular proteins. (PDF)

S2 Appendix Electronic configuration of the peptide amide bonds and conformational and H-bonding propensity of the polypeptide backbone. (PDF)

S3 Appendix Electronic configuration of the polypeptide backbone and canonical peptide recognition by the PDZ domains. (PDF)

S4 Appendix Folding template and tertiary structure of soluble proteins: The congeners of *Borrelia* spirochete antigen OspA. (PDF)

S5 Appendix Electronic configuration of the polypeptide backbone and molecular recognition in formation of β structure: Folding of inteins. (PDF)

S1 Table NMR shielding tensors of C^α^ atoms of Xaa σ(C^α^)^Xaa^ (ppm, GIAO//BLYP3/D95**). (PDF)

S1 Dataset Cartesian atomic coordinates and total energies: Helix & Hairpin models (141 entries), secondary structure models 1–5 (5 entries), β structure models 6–8 (8 entries), and Trp-Cage mini-protein model 9 (2 entries). (PDF)
