# Supplementary material for: Protein folding, misfolding and aggregation: The importance of two-electron stabilizing interactions
Source: PLoS One. 2017 Sep 18;12(9):e0180905. doi: 10.1371/journal.pone.0180905 (PMC5603215; doi:10.1371/journal.pone.0180905)
Supplement: S1 Appendix — (PDF) [file pone.0180905.s001.pdf]

Appendix 1

Folding Template and Three-Dimensional Structure of Soluble Globular Proteins. According to the early hypothesis of Ghosh explaining nonideality of electrolyte solutions, the solution of 1:1 electrolyte is thought of as an expanded, solvent-filled quasi-crystal of the NaCl type as shown in the diagram [83,84].

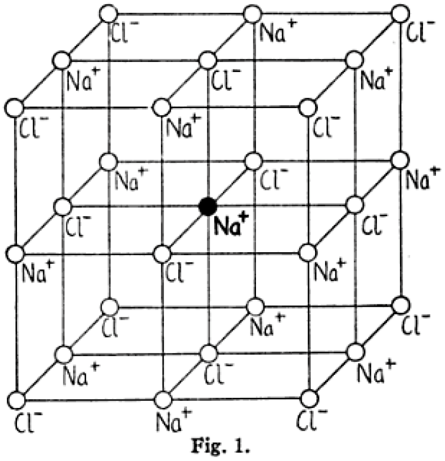

Fig. 1.

The Ghosh theory was soon superseded by the theory proposed by Debye and Hückel [48]. In spite of the different approach, Debye and Hückel accepted the cubic-lattice model of the 1:1 electrolyte solution with one caveat: the lattice order in a crystal is fixed while in a solution it is determined by the thermodynamic equilibrium between the attractive coulombic interactions and the thermal energy of ion motion.

"Trotzdem wir es somit ablehnen müssen, von einer Gitterstruktur des Elektrolyten im landläufigem Sinne zu sprechen und es […] zu unzulässigen Fehler führt, falls man das Bild zu wörtlich nimmt, is doch ein Kern von Wahrheit in ihm enthalten. Um dieses klar zu machen, führe man die folgenden zwei Gedankenexperimente aus. Erstens nehme man ein Raumelement und denke sich dieses viele Male hintereinander an beliebigen Stellen des Elektrolyten verlegt. Es ist klar, daß man dann bei einem binären Eelektrolyten ebensooft ein positives wie ein negatives Ion in ihm vorfinden wird. Zweitens aber nehme man dasselbe Raumelement und lege es wieder viele Male hintereinander in den Eelektrolyten hinein, nun aber nicht ganz beliebig, sonder immer so, daß es z. B. von einem übrigens jeweilig beliebig gewählten positiven Ion stets

um eine bestimmte Strecke (von einigen Å-Einheiten) entfernt ist. Nunmehr wird man nicht mehr gleich oft positive wie negative Ladungen vorfinden, sondern es werden die negativen an Zahl überwiegen. Darin, daß also in der unmittelbaren Umgebung jedes Ions die entgegengesetzt geladenen im Mittel an Zahl überwiegen, kann man mit Recht eine Analogie zum Kristallbau des *NaCl*-Typus sehen, wo jedes *Na*-Ion unmittelbar von 6 *Cl*-Ionen und jedes *Cl*-Ion von 6 *Na*-Ionen umgeben ist. Nur ist als wesentlicher Punkt bei Elektrolytlösung zu beachten, daß das Maß jener Ordnung bestimmt wird durch das thermische Gleichgewicht zwischen anziehenden Kräften und Temperaturbewegung, während es beim Kristall fest vorgegeben ist."

The Debye-Hückel theory does not define the mean interionic distance but rather the thickness of ionic atmosphere, a diffuse shell of net charge of opposite sign surrounding any given ion in solution. Here we assume that in the dilute solutions the thickness of ionic atmosphere, the so-called Debye radius, approximates the mean interionic distance and can be taken as the constant of the cubic quasi-lattice of the 1:1 electrolyte solution (the Ghosh-Debye-Hückel ionic matrix). Under the physiological conditions of ionic strength and temperature, the Debye radius approaches 7 Å i.e. the Bjerrum distance in water - the distance at which the Coulomb energy for two unit charges equals the thermal energy $k_B T$ [82]. It means that the lattice of 1:1 electrolyte solution has optimal stability under the physiological conditions: it is more diffuse at lower concentrations, and may be compromised by the formation of ion pairs at higher concentrations. Given the relatively robust Ghosh-Debye-Hückel matrix, the protein-solvent system should be stabilized by placing protein's key surface charges in the appropriate vertices of the ionic lattice. Thus, the soluble globular proteins and their environment might have evolved to take advantage of the nonideality of dilute 1:1 electrolyte solutions [86].
