# Supplementary material for: Protein folding, misfolding and aggregation: The importance of two-electron stabilizing interactions
Source: PLoS One. 2017 Sep 18;12(9):e0180905. doi: 10.1371/journal.pone.0180905 (PMC5603215; doi:10.1371/journal.pone.0180905)
Supplement: S2 Appendix — (PDF) [file pone.0180905.s002.pdf]

Appendix 2

Electronic Configuration of the Peptide Amide Bonds and Conformational and H-Bonding Propensity of the Polypeptide Backbone. The energy of the two-electron stabilizing interactions that contribute to the stereoelectronic control of the backbone conformation is the energy of the donor-acceptor interactions of the localized natural bond orbitals $\Delta E^{(2)}$ obtained from the NBO analysis [12] of the polyalanine models of secondary structure **1-5** at the B3LYP/6-31G* level of the theory (cf. Computational Methods).

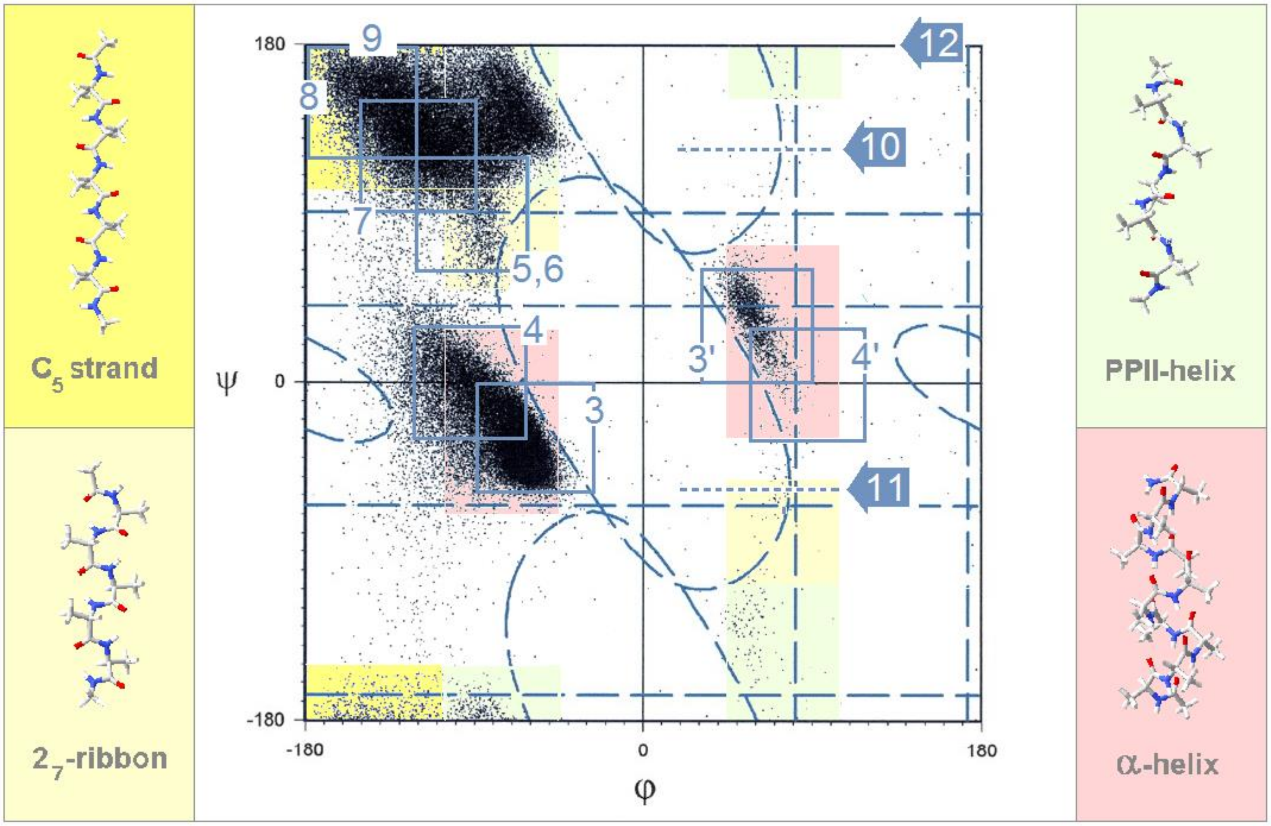

(i) **The α-helix/3_{10}-helix/β turn region**:

1. The $n(N_i)\rightarrow\sigma^*(C^\alpha_i–C'_i)$ hyperconjugation, the generalized anomeric effect, maximized when the $C^\alpha_i–C'_i$ bond, the best hyperconjugative σ acceptor at C^α_i, is antiperiplanar to the N_i lp ($-90°<\varphi_i<-60°$ or $60°<\varphi_i<90°$; 9-12 kcal mol^{-1}).

2. The C_{i-1}=O···C_i=O homohyperconjugation $n(C'_{i-1}=O)\rightarrow\pi_3^*(N_{i+1}–C'_i=O)$ maximized when the $C^\alpha_i–C'_i$ bond eclipses the $N_i–C'_{i-1}$ bond, $\varphi_i=0°$ and $\psi_i=90°$ or $-90°$ i.e. in the sterically forbidden regions; in the allowed regions the effect is maximized when ψ_i and φ_i vary from $\varphi=-30°/\psi=-60°$ to $\varphi=-90°/\psi=0°$, **2a** and in the same fashion in the mirror image region **2a'** (cf. Chart 1, note the ψ_i/φ_i correlations); the two-electron contribution is often not detectable [33] by the NBO analysis of helical structures.

3. The $n/\pi(C'_i=O)\rightarrow\sigma^*(N_{i+4}–H)$ homohyperconjugation (the α-helix backbone-backbone H-bond C'_i=O···H–N_{i+4}, includes coulombic as well as covalent contributions), maximized when

the four consecutive residues have torsion angles in the range φ=–60°±30°/ψ=–60°±30°; 7-8.5 kcal mol^{-1} (total for the three covalent contributions).

4. the $n(N_i)\rightarrow\sigma^*(N_{i+1}–H)$ homohyperconjugation [22] (the backbone-backbone H-bond $N_i\cdots H–N_{i+1}$, includes a coulombic as well as a covalent contribution), maximized when the C^α_i –C'$_i$ bond is perpendicular to the N_i–C'$_{i-1}$=O plane and the N_{i+1}–H bond eclipses the C^α_i –N_i bond φ_i=–90°±30°/ψ_i=0°±30° and φ_i=90°±30°/ψ_i=0°±30°; 0.5-0.7 kcal mol^{-1}.

11. The $\sigma(C^\alpha_i–H)\rightarrow\sigma^*(C'_i–N_{i+1})$ hyperconjugation, maximized when the C^α_i–H bond is antiperiplanar to the C'$_i$–N_{i+1} bond, ψ_i=–60°±30°; 3.2-4.2 kcal mol^{-1}.

(ii) **The 2_7-ribbon (C_{7eq} strand) region**:

1. The $n(N_i)\rightarrow\sigma^*(C^\alpha_i–C'_i)$ hyperconjugation, *vide supra*.

5. The $n(C'_i=O)\rightarrow\sigma^*(N_{i+2}–H)$ homohyperconjugation **5a** (the 2_7-ribbon backbone-backbone H-bond C'$_i$=O···H–N_{i+2}, including a coulombic as well as a covalent contribution), maximized when two consecutive residues have torsion angles in the range φ=–90°±30°/ψ=90°±30° (8.0-9.5 kcal mol^{-1}), and the $n(C'_i=O)\rightarrow\sigma^*(N_j–H)$ homohyperconjugation **5b** (the C_{7eq} inter-chain backbone-backbone H-bonds C'$_i$=O···H–N_j, including coulombic as well as covalent contributions, 11.5 kcal mol^{-1}).

6. The $n(N_i)\rightarrow\pi_3^*(N_{i+1}–C'_i=O)$ homohyperconjugation, maximized when the C^α_i –N_i bond is perpendicular to the N_{i+1}–C'$_i$=O plane and the N_i lp eclipses the C^α_i –C'$_i$ bond, φ_i=–90°±30° or 90°±30° and ψ_i=–90°±30° or 90°±30°; 0.5-2.2 kcal mol^{-1}.

(iii) **The PP_{II}-helix region**:

1. The $n(N_i)\rightarrow\sigma^*(C^\alpha_i–C'_i)$ hyperconjugation, *vide supra*.

2. The C_{i-1}=O···C_i=O homohyperconjugation, *vide supra*, cf. **2b** in Chart 1.

10. The $\sigma(C^\alpha_i–H)\rightarrow\sigma^*(C'_i–O)$ hyperconjugation, maximized when the C^α_i–H bond is antiperiplanar to the carbonyl group, ψ_i=120°±30°; 2.5-4.5 kcal mol^{-1}.

(iv) **The C_5 strand region**:

7. The extended hyperconjugation: the $\sigma(C^\alpha_i–H)\rightarrow\sigma^*(C'_i–O)$ hyperconjugation **10**, *vide supra*, enhanced by the $lp(O_{i-1})\rightarrow\sigma^*(C^\alpha_i–H)$ homohyperconjugation, maximized when the C^α_i–H bond eclipses the N_i–C'$_{i-1}$ bond, φ_i=–120°±30°(~0.7 kcal mol^{-1}).

8. The $n(C'_i=O)\rightarrow\sigma^*(N_i–H)$ homohyperconjugation (the C_5 backbone-backbone H-bond C'$_i$=O···H–N_i), maximized when φ_i=–150°±30°/ψ_i=150°±30°; 0.6-2.0 kcal mol^{-1}.

9. The $n(C'_i=O)\rightarrow\sigma^*(N_j–H)$ and $n(C'_i=O)\rightarrow\sigma^*(C^\alpha_{j-1}–H)$ [66,67] homohyperconjugation **9a** and **9b**, respectively (the C_5 inter-chain backbone-backbone H-bonds C'$_i$=O···H–N_j and C'$_i$=O···H–C^α_{j-1}, including coulombic as well as covalent contributions), maximized when φ_i=–150°±30°/ψ_i=150°±30°; 14.5-17.2 kcal mol^{-1} and ~1.5-2.5 kcal mol^{-1}, respectively.

12: The extended hyperconjugation $\pi_2(N_i–C_{i-1}=O)\rightarrow\pi(C^\alpha_iRR')\rightarrow\pi_3^*(N_{i+1}–C'_i=O)$[21] involving N_i lp as a π donor, π orbital of the C^α_i ligand as the 'relay' orbital, and $\pi^*(C_i=O)$ as an acceptor, maximized when φ_i=180°±30°/ψ_i=180°±30°; the $\pi(C^\alpha_iRR')\rightarrow\pi_3^*(N_{i+1}–C'_i=O)$ interaction alone is also maximized when ψ_i=0°±30°. The hyperconjugative stabilization reported in ref. [21] is ~3.0-5.5 kcal mol^{-1}.

The Weinhold's energy of the $lp(O_{i-1})\rightarrow\sigma^*(N_i–C'_{i-1})$ hyperconjugation which generates the structure I in Chart 1B is in the range of ~19-23 kcal mol^{-1} in the set of secondary structure models **1-8**, cf. Computational Methods; the energy of the $\sigma(H–N_i)\rightarrow\sigma^*(C'_{i-1}–O)$ hyperconjugation which generates the structure V is ~4.4-5.6 kcal mol^{-1}.
