# Supplementary material for: Protein folding, misfolding and aggregation: The importance of two-electron stabilizing interactions
Source: PLoS One. 2017 Sep 18;12(9):e0180905. doi: 10.1371/journal.pone.0180905 (PMC5603215; doi:10.1371/journal.pone.0180905)
Supplement: S3 Appendix — (PDF) [file pone.0180905.s003.pdf]

Appendix 3

Electronic Configuration of the Polypeptide Backbone and Canonical Peptide Recognition by the PDZ Domains. The proposed theory implies that the ΔG_b maxima in Fig 12 are found at different $\overline{FP}_i$ values because the binding pockets of the PDZ domains differ in the capacity to polarize the peptide ligands. For instance, the pockets of the MAGI2/2, PTP-BL and Lin7C domains, Figs 12A, 12B and 12C, are, according to the model, non-polar and therefore poorly bind the relatively polarized peptides with $\overline{FP}_i$ in the range from –0.15 to –0.25 which tend to adopt helical conformations in non-polar environments, cf. Fig 7(b). On the other hand, the pockets of the TIAM1&2, γ-syntrophin1 and RGS3 domains, Figs 12P, 12Q and 12R, are, according to the model, polar and therefore poorly bind the less polarized peptides with $\overline{FP}_i$ in the range from 0.10 to 0.20. The layout of the secondary structure elements suggests that the major factor is the charge polarization of the polypeptide backbone. The two helices and the cross-*β* peptide-bond array of the PDZ fold are set up to fit the Ghosh-Debye-Hückel matrix with the lattice constant of 7 Å as shown in the panels **(A)-(C)**. Binding of the C-terminal peptide ligand, cf. Fig 12, augments this set-up and stabilizes the protein/electrolyte system. Two peptide amide bonds of the bound ligand join the cross-*β* arrays that extend through the five strands *β*1-*β*6-*β*4-*β*3-*β*2 as shown below. Thus, the differences in the polarizing effect of the binding-pocket are likely to be caused by the variation in charge polarization of these two cross-*β* arrays. The average FP_i value of the residues involved in the two arrays in question, $\overline{FP}_i$(PDZ/sheet), can be taken as the measure of their charge polarization and thus the capacity of the binding pocket to polarize the bound oligopeptide. One expects then to find a correlation between the $\overline{FP}_i$(peptide) values at the ΔG_b maximum and the $\overline{FP}_i$(PDZ/sheet) values; as shown in panel (**E**) these two parameters do seem to correlate.

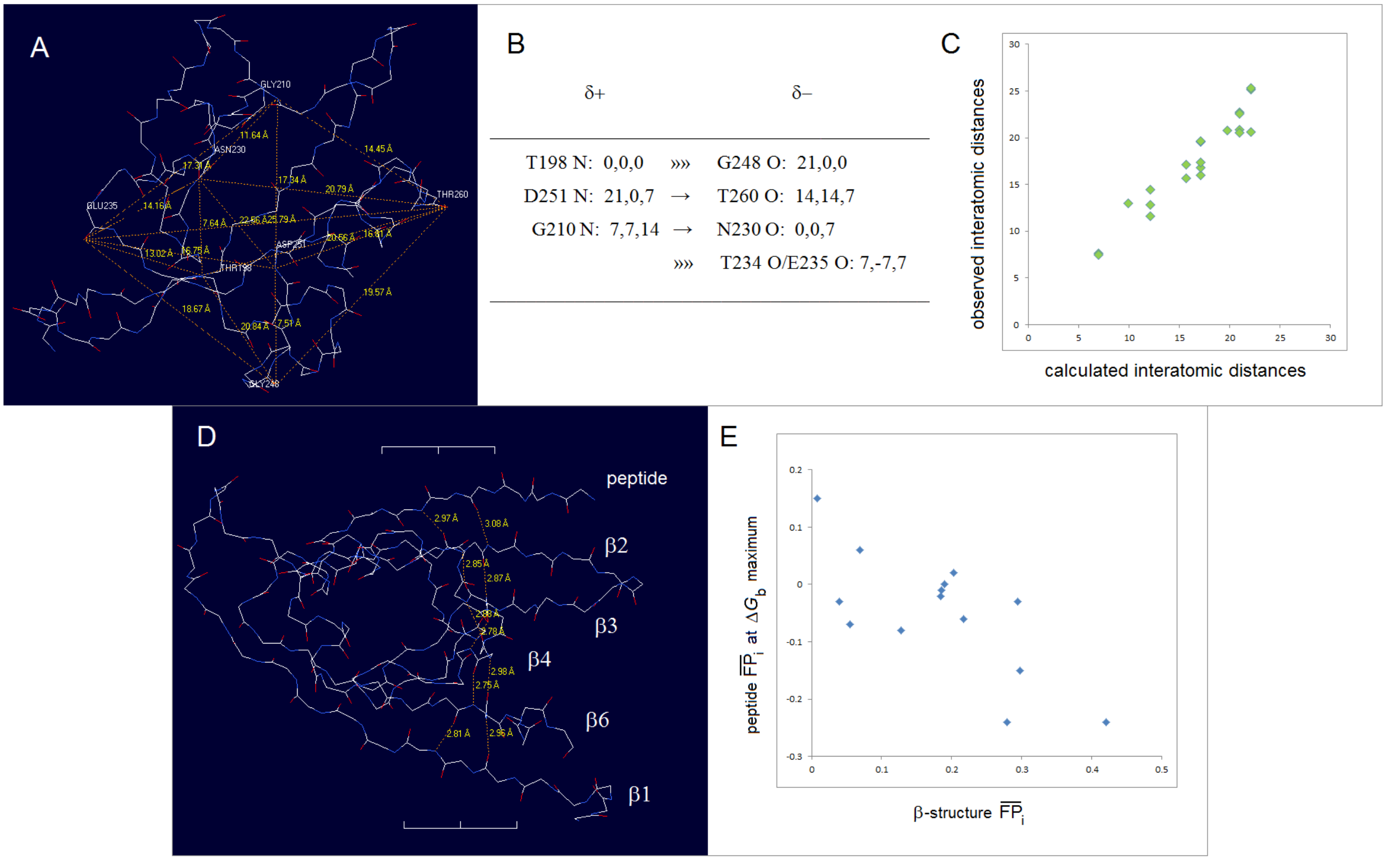

(A) Folding template and 3D structure of the PDZ domains: the putative ‘key’ surface charges of the PDZ fold are the termini of the α1 helix-[CO_2^- loop] and the α2 helix arrays (→), and the cross-β arrays (»») capped by a reverse turn (G248 (C’=O)) and a bulge (T234 (C’=O)/E235 (C’=O)). The structure in the diagram is the second PDZ domain of syntenin, PDB ID 1r6j. **(B)** The projected fit of the putative key surface charges δ^+ and δ^- into the Ghosh-Debye-Hückel matrix. **(C)** The predicted by the folding-template model and the observed interatomic distances (Å) between the key surface charges δ^+ and δ^- (the average distances to the T234 O/E235 O atoms in the bulge are used). **(D)** Two cross-β (β1-β6-β4-β3-β2) arrays of peptide bonds that anchor the oligopeptide ligand by the backbone-backbone H-bonds to N-H of the residue i=-*2* and to C’=O of the residue i=-*4*. **(E)** The oligopeptide $\overline{FP}_i$ values at the ΔG_b maximum (determined from Fig 12 for the 14 entries with β sheet register confirmed by the X-ray or NMR structure determination) plotted against $\overline{FP}_i$(PDZ/sheet) i.e. the average FP_i values of the 15 residues involved in the two cross-β arrays which are shown in the panel **(D)**.
