# Supplementary material for: Protein folding, misfolding and aggregation: The importance of two-electron stabilizing interactions
Source: PLoS One. 2017 Sep 18;12(9):e0180905. doi: 10.1371/journal.pone.0180905 (PMC5603215; doi:10.1371/journal.pone.0180905)

Appendix 4

Folding Template and Tertiary Structure of Soluble Proteins: the Congeners of *Borrelia* Spirochete Antigen OspA. Nearly two dozen variants of the crystalline mutant of OspA were obtained by the deletion and insertion of strands into the central β-sheet, by multiple mutations in the central β-sheet, and by modification and replacement of its turns. The protein/electrolyte system is in this case likely to be stabilized when the alkyl ammonium ions of the lysine side chains replace the salt cations in the vertices of the ionic matrix. Manual search for the best fit of the set of six "reporter" lysines into this matrix (K69, K88 and K112 in the N-terminal globular domain, and the three C-terminal-domain lysines equivalent to K193, K211 and K230 of the parent OspA structure) identifies eight types of the OspA variants (PDB ID's: **1** 3ckf; **2** 3ckg; **3** 2g8c, 2i5v, 2i5z, 2ol6, 2ol8, 2oy1, 2oy5, 2oyb, 2pi3; **4** 2ol7A, 2ol7B; **5** 2af5; **6** 2fkg, 2hkd, 2oy7, 2oy8, 3ec5; **7** 3eexA, 3eexB, 3ckaA, 3ckaB; **8** 2fkjA, 2fkjB, 2fkjC) and yields the coordinates of the N_ζ atoms shown in the panel (**A**). The parent OspA structure and an example of its modification are shown in the panel (**B**). The matrix of the interatomic distances (between, on the one hand, the N_ζ atoms of K69, K88 and K112 in the N-terminal globular domain, and, on the other hand, the N_ζ atoms of the three C-terminal-domain lysines equivalent to K193, K211 and K230 of the parent OspA structure) calculated using the coordinates from panel (A), are plotted against the observed distances in the panel (**C**).

A

Lysines in N-terminal domain	Lysines in C-terminal domain	PDB ID
+ + +	+ + +	
**1.** 69 [0,0,0]/85 [0,28,0]/112 [14,14,0]	170 [14,14,0]/188 [42,21,21]/207 [42,7,35]	3ckf
**2.** 69 [0,0,0]/85 [0,28,0]/112 [14,14,0]	178 [21,14,21]/196 [42,21,35]/215 [49,35,28]	3ckg
**3.** 69 [0,0,0]/85 [0,28,0]/112 [14,14,0]	193 [28,7,-7]/211 [56,7,7]/230 [63,-7,14]	2g8c, 2i5v 2i5z, 2oy5, 2oyb, 2pi3
	192 [28,7,-7]/210 [56,7,7]/229 [63,-7,14]	2ol6, 2oy1
	191 [28,7,-7]/209 [56,7,7]/228 [63,-7,14]	2ol8
**4.** 69 [0,0,0]/85 [0,28,0]/112 [14,14,0]	193 [28,0,0]/211 [56,0,14]/230 [56,-14,28]	2ol7A, 2ol7B
**5.** 69 [0,0,0]/85 [0,28,0]/112 [14,14,0]	239 [42,14,28]/257 [49,21,56]/276 [49,7,70]	2af5
**6.** 69 [0,0,0]/85 [0,28,0]/112 [14,14,0]	262 [56,14,28]/280 [63,21,56]/299 [63,7,70]	2fkg, 2hkd 2oy7, 2oy8, 3ec5
**7.** 69 [0,0,0]/85 [0,28,0]/112 [14,14,0]	262 [56,0,0]/280 [84,-7,7]/299 [84,-21,21]	3eexA, 3eexB 3ckaA, 3ckaB
**8.** 69 [0,0,0]/85 [0,28,0]/112 [14,14,0]	308 [70,14,28]/326 [91,28,49]/345 [91,28,63]	2fkjA, 2fkjB 2fkjC

[a] The Cartesian coordinate system is right-handed.

B

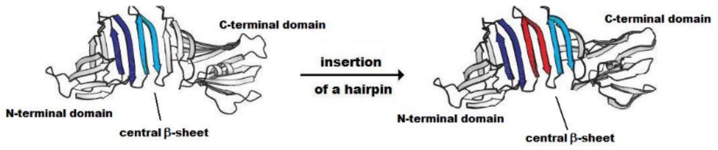

C

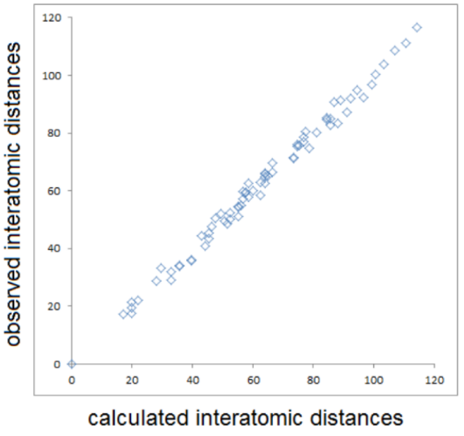

Supplement: S4 Appendix — (PDF) [file pone.0180905.s004.pdf]
