# Supplementary material for: Protein folding, misfolding and aggregation: The importance of two-electron stabilizing interactions
Source: PLoS One. 2017 Sep 18;12(9):e0180905. doi: 10.1371/journal.pone.0180905 (PMC5603215; doi:10.1371/journal.pone.0180905)
Supplement: S5 Appendix — (PDF) [file pone.0180905.s005.pdf]

Appendix 5

Electronic Configuration of the Polypeptide Backbone and Molecular Recognition in Formation of $\boldsymbol{\beta}$ Structure: Folding of Inteins. The FP_i assignment of secondary-structure propensity vs. distribution of side chain charges in the two-stranded antiparallel β-sheet $\beta_N\uparrow\beta_C\downarrow$ of 15 inteins and intein-like domains of the known 3D structure. **(A)** The architecture of the horseshoe-like fold of inteins: (a) the antiparallel assembly of the 30-35 residue-long strands β_N and β_C into the $\beta_N\uparrow\beta_C\downarrow$ sheet constitutes the major feature of the HINT fold. Each strand comprises four segments, $\beta_N a'$-$\beta_N a$-$\beta_N b$-$\beta_N b'$ and $\beta_C a'$-$\beta_C a$-$\beta_C b$-$\beta_C b'$ (cf. the labels color-coded blue and red, respectively), which are marked by the changes in the direction of the main chain (produced by the inherent right-handed twist of the polypeptide backbone and the insertion of turns); (b) the putative assembly of the full-length β_N and β_C strands into the $\beta_N\uparrow\beta_C\downarrow$ sheet. The interactions of the terminal-segment pairs, $\beta_N a'$-$\beta_C b'$ and $\beta_N b'$-$\beta_C a'$, may contribute to the stabilization of the correct register in the initial stages of folding; (c) in the native state, the $\beta_N\uparrow\beta_C\downarrow$ sheet comprises the middle-segment pairs $\beta_N a$-$\beta_C b$ and $\beta_N b$-$\beta_C a$ since the C-terminal segments of both strands, $\beta_N b'$ and $\beta_C b'$, are folded towards the protein interior. **(B)** The FP_i assignment of secondary-structure propensity vs. distribution of the side chain charges in the case of the full 'symmetry' of backbone polarization of the middle-segment pairs $\beta_N a$-$\beta_C b$ and $\beta_N b$-$\beta_C a$, shown within the green rectangles in the schematic representation of the $\beta_N\uparrow\beta_C\downarrow$ sheet. The PDB ID-labeled diagrams show FP_i plots, DSSP assignments, and the sequences of the 30-35 residue-long β_N and β_C strands with D, E marked by red squares and K, R marked by blue squares. The four segments of each β_N and β_C strand are defined based on the DSSP/ Swiss-Prot assignments and the inspection of the 3D structures, and are shaded yellow or light-yellow in the FP_i plots depending on the secondary-structure preferences; the latter are assigned based on Figs 5 and 7. This information is summarized below in the list of the PDB ID's, the total charges carried by the β_N and β_C strands, and the segment assignments in the format $<\beta_N a|\beta_C b>=<\beta_N b|\beta_C a>$ which includes: (i) the residue numbers, (ii) the C_5, C_{7eq} or C_5* preferences (in bold for a 'matching' pair), and (iii) the charges carried by each individual segment (in bold for opposite charges, in italics for same charges).

1at0 (β_N −3/ β_C +4): $<^{(299-307)}\mathbf{C_{7eq}}/-1|^{(386-380)}\mathbf{C_{7eq}}/0>-<^{(308-317)}\mathbf{C_{7eq}}/0|^{(379-370)}\mathbf{C_{7eq}}/+2>$

1dq3 (β_N +6/ β_C −3): $<^{(68-75)}\mathbf{C_5}/0|^{(438-430)}\mathbf{C_5}/-2>-<^{(81-88)}\mathbf{C_5}/0|^{(428-422)}\mathbf{C_5}/-1>$

1lws (β_N +5/ β_C −6): $<^{(40-45)}\mathbf{C_{7eq}}/\mathbf{+1}|^{(437-428)}\mathbf{C_{7eq}}/\mathbf{-3}>-<^{(46-52)}\mathbf{C_5}/0|^{(427-420)}\mathbf{C_5}/-1>$

2cw7 (β_N +8/ β_C −9): $<^{(70-77)}\mathbf{C_{7eq}}/0|^{(520-512)}\mathbf{C_{7eq}}/-2>-<^{(79-84)}\mathbf{C_5}/\mathbf{+1}|^{(511-503)}\mathbf{C_5}/\mathbf{-4}>$

2jnq (β_N +8/ β_C −6): $<^{(76-84)}\mathbf{C_{7eq}}/\mathbf{+4}|^{(156-149)}\mathbf{C_{7eq}}/\mathbf{-1}>-<^{(86-92)}\mathbf{C_5}/\mathbf{+2}|^{(148-140)}\mathbf{C_5}/\mathbf{-1}>$

2lcj (β_N −3 / β_C 0): $<^{(63-71)}\mathbf{C_5}/\ \underline{-2}\ |^{(169-161)}\mathbf{C_5}/\ \underline{-2}\ >-<^{(75-81)}\mathbf{C_{7eq}}/0|^{(158-151)}\mathbf{C_{7eq}}/+3>$

2lqm (β_N +4/ β_C −4): $<^{(67-74)}\mathbf{C_5}/0|^{(156-148)}\mathbf{C_5}/-1>-<^{(75-81)}\mathbf{C_5}/+1|^{(147-139)}\mathbf{C_5}/0>$

2lwy (β_N $\underline{-4}$/ β_C $\underline{-2}$): $<^{(39-51)}\mathbf{C_5}/\mathbf{-1}|^{(118-109)}\mathbf{C_5}/\mathbf{+2}>-<^{(52-57)}\mathbf{C_{7eq}}/0|^{(108-102)}\mathbf{C_{7eq}}/0>$

3ifj (β_N +1 / β_C 0): $<^{(44-51)}\mathbf{C_5}/\ \underline{-1}\ |^{(426-421)}\mathbf{C_5}/\ \underline{-2}\ >-<^{(52-61)}\mathbf{C_{7eq}}/\ \underline{+1}\ |^{(419-410)}\mathbf{C_5}/\ \underline{+3}\ >$

(C) The FP_i assignment of secondary-structure propensity vs. distribution of side chain charges in the case of the partial 'symmetry' of backbone polarization of the middle-segment pairs $\beta_N a$-$\beta_C b$ and $\beta_N b$-$\beta_C a$. The 'matching' $\beta_N a$-$\beta_C b$ pairs are shown within the green rectangles and the 'mismatched' $\beta_N b$-$\beta_C a$ pairs within the red rectangles in the schematic representation of the $\beta_N\uparrow\beta_C\downarrow$ sheet.

1zd7 (β_N −4/ β_C +3): $<^{(46-52)}\mathbf{C_{7eq}}/0|^{(142-136)}\mathbf{C_{7eq}}/0>-<^{(53-61)}C_5/\mathbf{-4}|^{(135-128)}C_5^*/\mathbf{+2}>$

(D) The FP_i assignment of secondary-structure propensity vs. distribution of side chain charges in the case of the full 'symmetry' of backbone polarization of the middle-segment pairs $\beta_N a$-$\beta_C b$ and $\beta_N b$-$\beta_C a$, shown within the red rectangles in the schematic representation of the $\beta_N\uparrow\beta_C\downarrow$ sheet.

1am2 (β_N 0 / β_C 0): $<^{(48-55)}C_{7eq}/\mathbf{-1}|^{(182-175)}C_5/\mathbf{+1}>-<^{(56-63)}C_5/\mathbf{+1}|^{(174-166)}C_{7eq}/\mathbf{-1}>$

1mi8 (β_N +8/ β_C −7): $<^{(47-53)}C_{7eq}/\mathbf{+2}|^{(138-131)}C_5/\mathbf{-3}>-<^{(54-61)}C_{7eq}/\mathbf{+2}|^{(129-122)}C_5/\mathbf{-1}>$

4o1r (β_N +5/ β_C −8): $<^{(48-53)}C_{7eq}/0|^{(124-117)}C_5/-3>-<^{(54-61)}C_{7eq}/\mathbf{+1}|^{(114-108)}C_5/\mathbf{-1}>$

4o1s (β_N +6/ β_C −6): $<^{(68-74)}C_{7eq}/\mathbf{+3}|^{(151-144)}C_5/\mathbf{-2}>-<^{(75-83)}C_5/0|^{(174-166)}C_{7eq}/+1>$

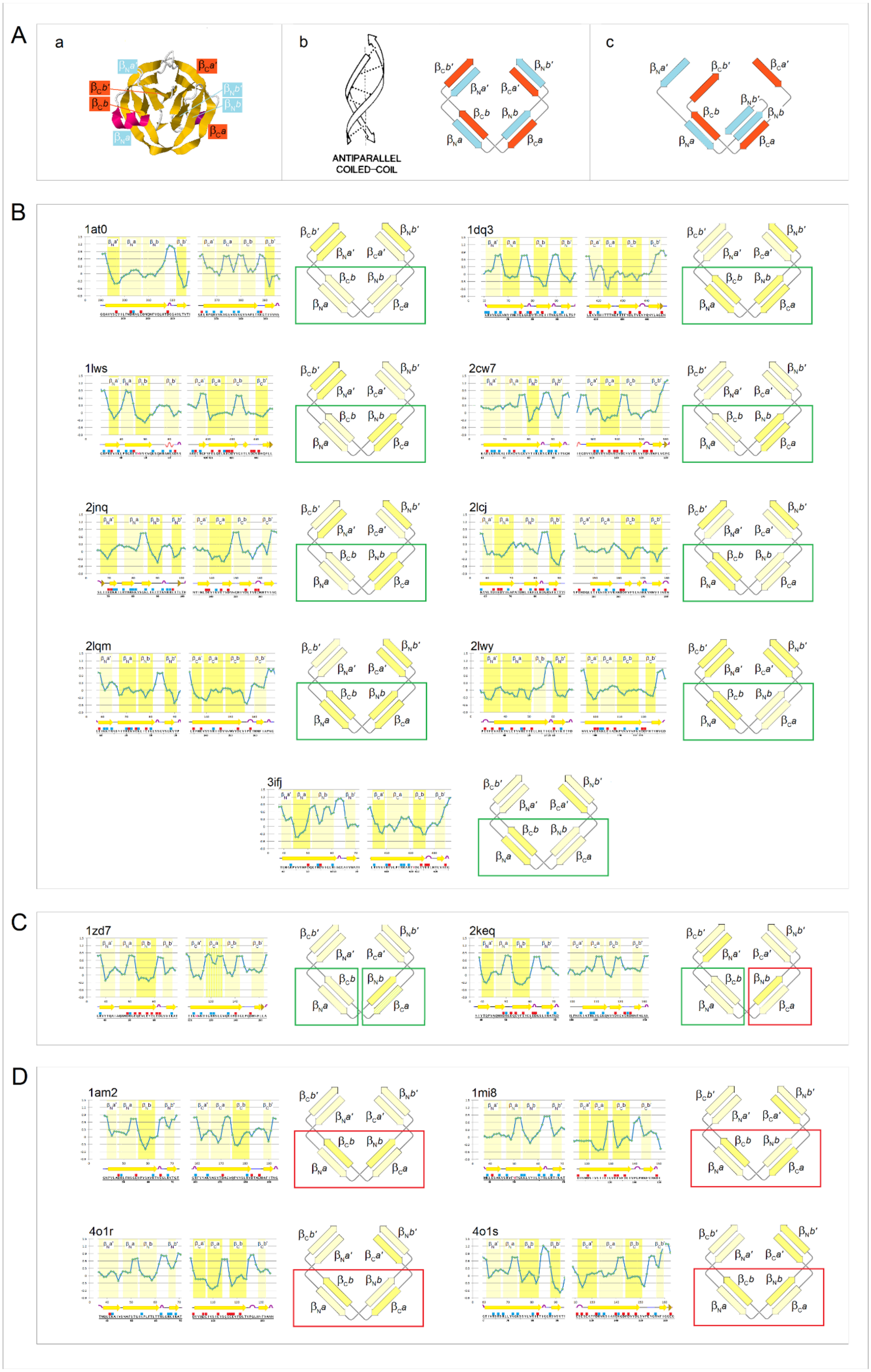
A
a
b
c
ANTIPARALLEL
COILED–COIL
B
1at0
1dq3
1lws
2cw7
2jnq
2lcj
2lqm
2lwy
3ifj
C
1zd7
2keq
D
1am2
1mi8
4o1r
4o1s

These findings can be summarized as follows. The four-segment long β_N and β_C strands carry complementary charges except in 4 structures, in panel **B**: 2lcj (one neutral strand), 2lwy (both strands carry negative charge) and 3ifj (one neutral strand), and in panel **D**: 1am2 (both strands are neutral). The complete 'symmetry' of backbone polarization of the $\beta_N\uparrow\beta_C\downarrow$ middle segments ($\beta_N a$-$\beta_C b$ and $\beta_N b$-$\beta_C a$ segment pairs, cf. panel **A**) is found in 9 structures (panel **B**), partial 'symmetry' in 2 structures (split inteins in panel **C**), and none in 4 structures (panel **D**) which yields the total of 20 FP_i-'matching' and 10 FP_i-'mismatched' (cf. Fig 2) middle-segment $\beta_N a$-$\beta_C b$ and $\beta_N b$-$\beta_C a$ pairs. Among the 20 'matching' $\beta_N a$-$\beta_C b$ and $\beta_N b$-$\beta_C a$ pairs, both segments carry *the same charge* in 4 cases, in 11 cases one of the two segments is neutral, and the charges are complementary in 5 cases (with just a single charge on one of the segments). On the other hand, among the 10 middle-segment pairs that lack the 'symmetry' of backbone polarization, the charges are complementary in 8 cases (with multiple charges on both segments in 4 cases), and only in 2 cases one of the two segments is neutral. In the split inteins (PDB ID's 1zd7 and 2keq), the complementarity of *multiple* charges is found in the $\beta_N b$-$\beta_C a$ segment pairs that lack the 'symmetry' of backbone polarization while in the 'matching' $\beta_N a$-$\beta_C b$ segment pairs both segments are either neutral (1zd7) or carry negative charges (2keq) cf. panel **C**. These findings are consistent with the notion that the side chain-side chain interactions may facilitate the initial stages of intein folding via the 'electrostatic capture', or stabilize the 3D structure once the backbone fold is established, but neither effect is necessary to assembly the two-stranded antiparallel β-sheet of inteins in the correct register. The discrete architecture of the $\beta_N\uparrow\beta_C\downarrow$ sheet (panel **A**) seems to depend on the complex pattern of backbone polarization, and the 'symmetry' of polarization of the middle-segment pairs $\beta_N a$-$\beta_C b$ and $\beta_N b$-$\beta_C a$ appears to be an important feature of this pattern.

The following inteins and intein-like domains are included in the study (PDB ID, function, organism, optimal growth temperature): (1) 1am2, GYRA intein, *Mycobacterium xenopi* (42°C); (2) 1at0, 17 kDA fragment of hedgehog C-terminal autoprocessing domain, *Drosophila melanogaster*; (3) 1dq3, archeal intein-encoded homing endonuclease PI-PfuI, *Pyrococcus furiosus* (~100°C); (4) 1lws, VMA1-derived

intein-encoded homing endonuclease PI-SceI, *Sacharomyces cerevisiae*; (5) 1mi8, DnaB intein, *Synechocystis* sp. PCC6803 (32-38°C); (6) 1zd7, DnaE split intein, *Synechocystis* sp. PCC6803 (32-38°C); (7) 2cw7, intein-encoded homing endonuclease II, *Thermococcus kodakarensis* (~85°C); (8) 2jnq, KlbA intein precursor, *Methanocaldococcus jannaschii* (~85°C); (9) 2keq: DnaE split intein, *Nostoc punctiforme*; (10) 2lcj, Pol II intein, *Pyrococcus abyssi* (~100°C); (11) 2lqm, RadA intein, *Pyrococcus horikoshii* (~100°C); (12) 2lwy, intein-like domain, *Chlostridium thermocellum* (60°C); (13) 3ifj, RecA intein, *Mycobacterium tuberculosis*; (14) 4o1r, DnaB intein, *Nostoc punctiforme*; (15) 4o1s, VMA intein, *Thermoplasma volcanium* (~60°C).
