# Supplementary material for: Protein folding, misfolding and aggregation: The importance of two-electron stabilizing interactions
Source: PLoS One. 2017 Sep 18;12(9):e0180905. doi: 10.1371/journal.pone.0180905 (PMC5603215; doi:10.1371/journal.pone.0180905)
Supplement: S1 Table — (PDF) [file pone.0180905.s006.pdf]

Table S1. NMR shielding tensors of C^{α} atoms of Xaa $\boldsymbol{\sigma}(C^{\alpha})^{Xaa}$ (ppm, GIAO//B3LYP/D95**).

Xaa	Helix AcG(Xaa)GGGNH$_2$/3$_{10}$			Hairpin AcGGGGG(Xaa)NHMe/Ib		
		–gauche	*trans*		*–gauche*	*trans*
A	129.8242			132.4588		
C		123.7072	122.1902[a]		126.6682	123.8432
D		128.9660	128.8014[a,c]		131.8015	132.5247
D$^-$		125.8097	124.9120[a,c,d]		129.9424	131.6990
E		123.7904	126.6269		130.0360	128.6303
E$^-$		123.5413	121.8813		127.4964[c]	124.0724[c]
F		123.8277	122.9585		127.1465	125.3391
G	138.6835			140.1612		
H		127.5916	123.5788		126.9764	126.7362
H$^+$		133.4182[b,d]	132.8260		130.7327	130.3937
I		119.5667	119.9058			123.0349
K		126.7650[c,d]	128.0968[c,d]		128.8763[c]	129.9112[c]
L		128.1296	127.6058		130.4657	128.8044
M		125.0847	125.6258		129.3598	127.9765
N		128.1104	130.3074		130.6071	128.9936
P	118.7353			119.2283		
Q		124.6273	125.6827		128.3718	127.9666
R		130.8786[b,c,d]	132.7277		130.5578[c]	129.5208[c]
S		122.5110	123.4178[a]		125.7306	125.9329
T		118.6245			120.2276	122.2653
V		118.9485	120.9960		122.6846	122.6846

W		127.2616	123.1853		128.8015	126.5019
Y		123.9992	123.5910		127.3391	125.6026
Atp	122.2039			127.4957		
Atb		130.3203	130.2224		134.8122	132.5465
Hfl		126.5119	129.8819		130.1052	130.8361
Pff		124.9485	127.4826		130.6195	130.3239
$Ser(P)^{-1}$		122.9059[e]				
$Ser(P)^{-2}$		114.6663[e]			120.0575	
AcK		124.9695	125.6697		127.3681	128.5047
$M(O_2)$		126.2322	126.9593		130.8778	128.2380
C(SMe)		128.3050	127.0340		130.4402	129.3812
Abu		123.6958	124.1238			
Nva		124.4077	124.9118			
Nle		124.4836	125.1795			
Tle	116.4503			121.5251		
Dpr		126.7504[b,c,d]	140.2855[a,c,d]			
Dab		130.9720[b,c,d]	135.3227[a,c,d]			
Orn		129.1371[b,c,d]	129.1010[a,c,d]			
Agp		128.1910[b,c,d]	134.3964[a,c,d]			
Agb		131.1411[b,c,d]	130.5099[a,c,d]			
Agh		126.3978[b,c,d]	128.0060[a,c,d]			
Thr/NH_2		120.9042			119.1258	121.2151
Gly-CN	136.5446			140.0365		
Gly-CNO	135.8620			139.9161		
Gly-CCH	134.1002			138.2613		

[a] Fixed *trans* rotamer.

[b] Fixed −*gauche* rotamer.

[c] Fixed sidechain extension.

[d] Fixed helix turn.

[e] Optimized to +*gauche* rotamer.

The standard one-letter code for the canonical amino acids is used. The modified sidechains have the following structures, in the order of listing in the table:

Atp	CF_3	Nva	$CH_2CH_2CH_3$
Atb	CH_2CF_3	Nle	$CH_2CH_2CH_2CH_3$
Hfl	$CH_2CH(CF_3)_2$	Tle	$C(CH_3)_3$
Pff	$CH_2C_6F_5$	Dpr	$CH_2NH_3^+$
Ser(P)-1	$CH_2OPO_3H^-$	Dab	$CH_2CH_2NH_3^+$
Ser(P)-2	$CH_2OPO_3^{-2}$	Orn	$CH_2CH_2CH_2NH_3^+$
AcK	$CH_2CH_2CH_2CH_2NHCOCH_3$	Agp	$CH_2NHC(NH_2)_2^+$
M(O2)	$CH_2CH_2SO_2CH_3$	Agb	$CH_2CH_2NHC(NH_2)_2^+$
C(SMe)	CH_2SSCH_3	Agh	$CH_2CH_2CH_2CH_2NHC(NH_2)_2^+$
Abu	CH_2CH_3		
