# Supplementary material for: Protein folding, misfolding and aggregation: The importance of two-electron stabilizing interactions
Source: PLoS One. 2017 Sep 18;12(9):e0180905. doi: 10.1371/journal.pone.0180905 (PMC5603215; doi:10.1371/journal.pone.0180905)
Supplement: S1 Dataset — (PDF) [file pone.0180905.s007.pdf]

Helix & Hairpin Models: Cartesian coordinates and total energies (in hartrees, B3LYP/D95**) (–*gauche* and *trans* designations refer to the conformation of the side chain about the C^{α}-C^{β} bond); the symbols and the listing order the same as in Table S1.

A Helix

C	-1.882336	3.334373	-0.611433
H	-0.972398	3.930162	-0.505370
H	-2.442969	3.396886	0.329639
H	-2.489103	3.767618	-1.414427
H	-6.950782	0.097261	1.000981
C	-6.099275	-0.238824	1.600967
C	-4.848722	-0.499865	0.782008
N	-5.004549	-0.504249	-0.584923
C	-3.954332	-1.030339	-1.455480
C	-2.638955	-0.240184	-1.451947
N	-2.704149	1.062086	-1.074347
C	-1.503991	1.885005	-0.939295
C	-0.496728	1.344167	0.097348
N	-0.957399	0.481867	1.040510
C	-0.034912	-0.137022	1.978653
C	1.030515	-1.053111	1.350721
N	0.810419	-1.485137	0.082165
C	1.819261	-2.251181	-0.631910
C	3.112157	-1.496952	-1.002622
N	3.108096	-0.144320	-0.826729
C	4.316504	0.645599	-0.967782
C	5.051591	0.993479	0.341447
N	4.599690	0.392272	1.476879
H	-6.373776	-1.165654	2.116900
H	-5.873603	0.508280	2.366900
O	-3.746393	-0.684587	1.302853
H	-5.947239	-0.469982	-0.950638
H	-3.680586	-2.052759	-1.173852
H	-4.331397	-1.049162	-2.483489
O	-1.591788	-0.796196	-1.791075
H	-3.593322	1.425239	-0.755395
H	-0.951762	1.855715	-1.884420
O	0.681873	1.707827	0.034951
H	-1.921324	0.155748	1.027342
H	0.526542	0.623060	2.530097
H	-0.610759	-0.726768	2.698489
O	2.034367	-1.352321	2.005339
H	-0.013418	-1.179673	-0.429148
H	2.146907	-3.105103	-0.031650
H	1.379752	-2.633941	-1.557684
O	4.071905	-2.135140	-1.431264
H	2.271104	0.324058	-0.490924
H	5.023735	0.089081	-1.589191
H	4.098273	1.596510	-1.463964
O	5.998098	1.778882	0.315694
H	3.830200	-0.269448	1.496992
H	5.102015	0.586646	2.331905

-1288.8722653

A Hairpin

H	-6.258013	2.988429	-1.201032
C	-5.686984	3.172647	-0.285340
C	-4.489141	2.239161	-0.262242
N	-3.270542	2.822695	-0.193647
O	-4.626227	1.006156	-0.305826
H	-5.411591	4.230569	-0.239469
H	-6.336074	2.932221	0.562788
H	-3.145096	3.829406	-0.141932
C	-2.053699	2.041531	-0.155176
H	-2.047775	1.365021	0.708278
H	-1.956573	1.410506	-1.045625
C	-0.850935	2.985940	-0.064104
N	0.341083	2.345258	-0.055694
O	-0.985959	4.213094	-0.004284
H	0.357915	1.320236	-0.091607
C	1.586172	3.071630	0.059407
H	1.843735	3.578879	-0.882535
H	1.491135	3.856071	0.822697
C	2.703881	2.116131	0.479940
N	3.928770	2.362746	-0.084770
O	2.521805	1.177149	1.253572
H	4.036330	3.181639	-0.669147
C	5.125022	1.667796	0.383350
H	6.006714	2.172386	-0.023388
H	5.188603	1.702206	1.475621
C	5.237101	0.175928	0.015850
N	4.373668	-0.281315	-0.937792
O	6.084995	-0.515767	0.569776
H	3.708561	0.373109	-1.327944
C	4.266052	-1.685235	-1.295735
H	4.327521	-1.821881	-2.380114
H	5.112608	-2.206438	-0.837912
C	2.972557	-2.386966	-0.841772
N	2.310331	-1.815642	0.203211
O	2.617082	-3.426603	-1.396141
H	2.598628	-0.905878	0.552402
C	1.045408	-2.340761	0.683516
H	1.018046	-3.403905	0.424209
C	-0.138255	-1.624879	0.000054
N	-1.268818	-2.346778	-0.130261
O	-0.065362	-0.435211	-0.350315
H	-1.321914	-3.321644	0.153245
C	-2.515999	-1.776507	-0.606991
H	-2.586600	-0.727273	-0.318046
H	-2.573990	-1.820033	-1.704316
C	-3.684161	-2.589548	-0.035385
N	-4.855091	-1.910419	0.012836
O	-3.522797	-3.767084	0.304131
H	-4.841514	-0.906736	-0.168426
C	-6.091662	-2.524332	0.472141
H	-5.918709	-3.595106	0.597614
H	-6.410782	-2.102607	1.433886
H	-6.892498	-2.370217	-0.260743
C	0.935869	-2.155761	2.208687
H	1.752822	-2.688040	2.706029
H	-0.015456	-2.547529	2.583198
H	1.002500	-1.093729	2.470581

-1536.2528868

C Helix/-gauche

H	-5.519330	-1.075441	2.439612
C	-5.677476	-1.658922	1.528313
C	-4.433253	-1.568871	0.663997
N	-4.635349	-1.268282	-0.662395
O	-3.300430	-1.740802	1.120445
H	-5.816384	-2.703799	1.827044
H	-6.584460	-1.310164	1.024716
H	-5.586731	-1.257205	-1.005785
C	-3.555898	-1.409171	-1.636130
H	-3.161805	-2.431056	-1.653260
H	-3.951739	-1.179318	-2.631398
C	-2.340518	-0.507903	-1.384597
N	-2.566035	0.640520	-0.699077
O	-1.226509	-0.850367	-1.788995
H	-3.521161	0.924063	-0.509810
C	-1.492923	1.565481	-0.368944
H	-1.006376	1.925262	-1.283416
C	-0.355854	0.911476	0.448164
N	-0.672143	-0.182791	1.186530
O	0.769388	1.418263	0.425236
H	-1.592015	-0.613288	1.107157
C	0.357046	-0.884714	1.937830
H	0.852238	-0.208185	2.640755
H	-0.116999	-1.686575	2.512062
C	1.491815	-1.502036	1.100752
N	1.268808	-1.665130	-0.227889
O	2.550121	-1.814196	1.656411
H	0.394481	-1.356183	-0.646345
C	2.325986	-2.132538	-1.110158
H	2.770951	-3.053009	-0.720708
H	1.893446	-2.351677	-2.090772
C	3.510491	-1.168870	-1.324150
N	3.361989	0.102600	-0.852704
O	4.517244	-1.579534	-1.898302
H	2.492802	0.381381	-0.406396
C	4.467789	1.041192	-0.831758
H	5.207069	0.722336	-1.571898
H	4.125072	2.045420	-1.100570
C	5.211239	1.177453	0.511583
N	4.872251	0.294790	1.491923
O	6.062693	2.054776	0.647693
H	4.188308	-0.445709	1.372112
H	5.387188	0.350797	2.359658
C	-2.026145	2.763944	0.439165
H	-1.205351	3.462732	0.611450
H	-2.394602	2.431153	1.415974
S	-3.456684	3.642595	-0.315216
H	-2.828148	4.030745	-1.445753

-1687.0559022

C Helix/trans

H	-5.889383	-0.270267	2.269241
C	-6.000841	-1.032562	1.493335
C	-4.699114	-1.148479	0.722078
N	-4.805891	-1.186438	-0.650392
O	-3.601963	-1.192284	1.282324
H	-6.189796	-1.987781	1.995979
H	-6.861235	-0.790187	0.861693
H	-5.733814	-1.272587	-1.044700
C	-3.677060	-1.606086	-1.479709
H	-3.298424	-2.585887	-1.168940
H	-4.017818	-1.687053	-2.517235
C	-2.457013	-0.675182	-1.457042
N	-2.674551	0.608982	-1.074861
O	-1.350421	-1.111182	-1.778782
H	-3.610813	0.856449	-0.779332
C	-1.583922	1.562994	-0.855261
H	-1.063361	1.742888	-1.802117
C	-0.486589	1.023085	0.089920
N	-0.887235	0.191105	1.089187
O	0.686288	1.358094	-0.081615
H	-1.838103	-0.170978	1.114226
C	0.088178	-0.373472	2.009402
H	0.649553	0.423390	2.506540
H	-0.445149	-0.942612	2.776980
C	1.156779	-1.294957	1.394977
N	0.932941	-1.772464	0.144346
O	2.168672	-1.560007	2.052385
H	0.119951	-1.467541	-0.383981
C	1.952726	-2.537972	-0.554633
H	2.303487	-3.364728	0.069726
H	1.514058	-2.959021	-1.464118
C	3.225518	-1.767556	-0.959724
N	3.188723	-0.410165	-0.831979
O	4.198796	-2.397169	-1.370364
H	2.339169	0.049289	-0.515437
C	4.375528	0.403819	-1.012467
H	5.091078	-0.155960	-1.621367
H	4.129144	1.331347	-1.538854
C	5.113639	0.815182	0.276520
N	4.684894	0.245238	1.436871
O	6.040729	1.621314	0.214060
H	3.936737	-0.438897	1.485586
H	5.191576	0.478923	2.279410
C	-2.221559	2.878000	-0.358389
H	-2.745544	2.703491	0.589085
H	-2.962336	3.202756	-1.098350
S	-1.071412	4.258031	-0.001949
H	-0.402967	4.211623	-1.173146

-1687.0479287

C Hairpin/-gauche

H	5.471719	3.263636	1.794757
C	4.954460	3.649164	0.910243
C	3.887336	2.650354	0.496399
N	2.614521	3.104985	0.468162
O	4.177572	1.479642	0.200731
H	4.553482	4.642721	1.132398
H	5.691886	3.729580	0.105157
H	2.370316	4.062630	0.701538
C	1.510294	2.251778	0.082740
H	1.716986	1.753918	-0.869428
H	1.344208	1.454116	0.818420
C	0.238236	3.096701	-0.028924
N	-0.829767	2.419815	-0.517991
O	0.208089	4.282517	0.320071
H	-0.709939	1.448889	-0.824134
C	-2.123573	3.059390	-0.663231
H	-2.310583	3.707641	0.200219
H	-2.162557	3.696687	-1.560214
C	-3.194270	1.980571	-0.821982
N	-4.297188	2.104261	-0.020242
O	-3.062097	1.039971	-1.606648
H	-4.377020	2.919824	0.572762
C	-5.460192	1.238511	-0.197125
H	-6.283751	1.627548	0.409332
H	-5.782646	1.233942	-1.243288
C	-5.268929	-0.243830	0.175252
N	-4.153256	-0.546607	0.902353
O	-6.107734	-1.066417	-0.175993
H	-3.518870	0.206382	1.133768
C	-3.753293	-1.909847	1.201307
H	-3.534801	-2.033404	2.266504
H	-4.594024	-2.562341	0.943511
C	-2.508923	-2.401407	0.441345
N	-2.293959	-1.865126	-0.792586
O	-1.785076	-3.269686	0.936232
H	-2.796061	-1.027953	-1.077972
C	-1.095790	-2.216225	-1.538640
H	-0.946345	-3.298528	-1.488537
H	-1.237923	-1.918561	-2.582392
C	0.122338	-1.452474	-0.994987
N	1.183963	-2.197557	-0.619741
O	0.089665	-0.218037	-0.900352
H	1.188161	-3.196981	-0.790904
C	2.380999	-1.640288	-0.007511
H	2.292877	-0.554336	-0.038303
C	3.617173	-2.119002	-0.794212
N	4.615242	-1.205987	-0.873455
O	3.656321	-3.266191	-1.251330
H	4.460561	-0.269442	-0.498233
C	5.887772	-1.499484	-1.514722
H	5.895818	-2.554563	-1.796031
H	6.024296	-0.889993	-2.416992
H	6.720234	-1.300576	-0.829193
C	2.529492	-2.103260	1.460911
H	2.652769	-3.189962	1.502947
S	1.162970	-1.594680	2.578855
H	3.430373	-1.649515	1.885753
H	0.203734	-2.381270	2.038500

-1934.4358577

C Hairpin/trans

H	5.860438	3.556300	0.030933
C	5.054611	3.705384	-0.695157
C	3.988859	2.648858	-0.460946
N	2.754512	3.100013	-0.140874
O	4.244990	1.439157	-0.555832
H	4.676188	4.728102	-0.606099
H	5.478635	3.562064	-1.694033
H	2.527072	4.089153	-0.098587
C	1.646750	2.201775	0.101416
H	1.502573	1.520402	-0.745803
H	1.828565	1.578216	0.985792
C	0.374089	3.030239	0.304612
N	-0.735350	2.283478	0.527980
O	0.390698	4.264729	0.264912
H	-0.667190	1.262559	0.475390
C	-2.039442	2.889331	0.647450
H	-2.333856	3.015905	1.701056
H	-1.996221	3.892304	0.204330
C	-3.097466	2.060860	-0.087865
N	-4.369777	2.172484	0.415508
O	-2.837203	1.328099	-1.040051
H	-4.540997	2.848570	1.148930
C	-5.511131	1.619693	-0.309298
H	-6.432227	2.009891	0.134614
H	-5.487126	1.920420	-1.361607
C	-5.619175	0.083669	-0.325677
N	-4.845807	-0.583886	0.580888
O	-6.383309	-0.459937	-1.115649
H	-4.249444	-0.035948	1.186587
C	-4.730701	-2.030578	0.603522
H	-4.900885	-2.426388	1.609951
H	-5.506844	-2.432945	-0.055085
C	-3.376930	-2.595149	0.138442
N	-2.609572	-1.775273	-0.637600
O	-3.057558	-3.743099	0.444454
H	-2.888155	-0.812743	-0.806704
C	-1.309208	-2.198268	-1.098854
H	-1.265140	-3.289925	-1.037682
H	-1.171845	-1.905300	-2.148153
C	-0.152666	-1.574017	-0.300014
N	1.036240	-2.202708	-0.430579
O	-0.290770	-0.535810	0.364071
H	1.123330	-3.084467	-0.929112
C	2.273743	-1.691659	0.152927
H	2.255172	-0.601006	0.124839
C	3.421224	-2.254631	-0.705962
N	4.446637	-1.403718	-0.933695
O	3.363265	-3.424019	-1.107467
H	4.337816	-0.415873	-0.695994
C	5.602606	-1.792489	-1.730207
H	5.694354	-2.880569	-1.714430
H	5.498282	-1.468062	-2.773975
H	6.507335	-1.339528	-1.312033
C	2.397622	-2.152486	1.626313
H	2.565155	-3.232503	1.670513
S	3.666945	-1.265063	2.611940
H	1.455384	-1.928483	2.134601
H	4.742721	-1.678022	1.909605

-1934.435424

D Helix/-gauche

H	5.314817	-1.455172	-2.443276
C	5.465782	-2.041171	-1.532220
C	4.239124	-1.901245	-0.649905
N	4.468524	-1.568886	0.661617
O	3.094638	-2.063888	-1.082942
H	5.561568	-3.091442	-1.828362
H	6.392276	-1.722445	-1.044682
H	5.424885	-1.528639	0.987227
C	3.397181	-1.619445	1.650760
H	2.956521	-2.620346	1.711516
H	3.815030	-1.370126	2.632098
C	2.222443	-0.672664	1.374309
N	2.494393	0.431913	0.636737
O	1.097820	-0.942621	1.805212
H	3.457463	0.668471	0.415624
C	1.451349	1.381988	0.278094
H	0.980597	1.788178	1.181094
C	0.286320	0.746764	-0.514866
N	0.549167	-0.399561	-1.190976
O	-0.811980	1.309706	-0.522396
H	1.449781	-0.866537	-1.092554
C	-0.515162	-1.095476	-1.897740
H	-0.978873	-0.441358	-2.642138
H	-0.081962	-1.954066	-2.419794
C	-1.675249	-1.602514	-1.022232
N	-1.451302	-1.703820	0.312264
O	-2.752743	-1.888406	-1.554945
H	-0.557073	-1.424425	0.709398
C	-2.523402	-2.071982	1.223191
H	-3.013959	-2.990148	0.886547
H	-2.094806	-2.257477	2.212424
C	-3.659766	-1.043153	1.389222
N	-3.455372	0.191935	0.847140
O	-4.680518	-1.373634	1.989820
H	-2.577253	0.403789	0.382141
C	-4.516091	1.178789	0.777861
H	-5.264914	0.936817	1.537329
H	-4.125139	2.179116	0.989299
C	-5.260674	1.275331	-0.568260
N	-4.968889	0.325401	-1.499596
O	-6.071868	2.182271	-0.749337
H	-4.317697	-0.437555	-1.342841
H	-5.485850	0.357969	-2.367326
C	2.025182	2.539760	-0.572064
H	1.229607	3.264907	-0.750377
H	2.368452	2.145717	-1.536264
C	3.222186	3.206829	0.078693
O	4.282405	2.642680	0.304886
O	3.006188	4.501467	0.381737
H	3.824006	4.844421	0.787283

-1477.4876496

D Helix/trans

H	-5.842296	-0.666074	2.220150
C	-5.879059	-1.467312	1.477273
C	-4.553906	-1.520516	0.739311
N	-4.625773	-1.658703	-0.629264
O	-3.470763	-1.432164	1.320856
H	-6.010870	-2.411497	2.017801
H	-6.739020	-1.316965	0.817034
H	-5.535158	-1.844628	-1.032330
C	-3.450459	-2.039269	-1.411724
H	-2.994070	-2.955210	-1.020678
H	-3.765085	-2.231783	-2.442665
C	-2.313363	-1.008807	-1.454521
N	-2.634541	0.267767	-1.127646
O	-1.177620	-1.368704	-1.767196
H	-3.591872	0.448004	-0.853069
C	-1.637924	1.333004	-0.967468
H	-1.156993	1.561069	-1.923290
C	-0.482414	0.884017	-0.029951
N	-0.841604	0.116353	1.040121
O	0.677878	1.215600	-0.256785
H	-1.773695	-0.287441	1.099260
C	0.165308	-0.346704	1.983367
H	0.699907	0.504609	2.416215
H	-0.338798	-0.877195	2.797012
C	1.268415	-1.269842	1.435613
N	1.078166	-1.831877	0.215753
O	2.279056	-1.458758	2.121154
H	0.269701	-1.577986	-0.345207
C	2.138544	-2.591570	-0.427430
H	2.511727	-3.369595	0.244599
H	1.730526	-3.076902	-1.319013
C	3.386359	-1.792779	-0.855683
N	3.289733	-0.433472	-0.815196
O	4.394231	-2.404343	-1.206401
H	2.412842	0.009298	-0.553710
C	4.444197	0.417989	-1.030587
H	5.182181	-0.134409	-1.618937
H	4.158067	1.313469	-1.591079
C	5.167463	0.908196	0.239231
N	4.736697	0.397517	1.426516
O	6.082479	1.724306	0.140220
H	4.009196	-0.305708	1.505423
H	5.238771	0.678958	2.257104
C	-2.414924	2.555628	-0.442115
H	-3.203049	2.820002	-1.159628
H	-2.922509	2.304745	0.498728
C	-1.616116	3.823718	-0.182529
O	-0.430660	3.993705	-0.339459
O	-2.453426	4.794878	0.280243
H	-1.900382	5.581470	0.436518

-1477.4734491

D Hairpin/-gauche

H	4.830597	3.337938	2.490241
C	4.413127	3.787443	1.583378
C	3.502744	2.773914	0.911834
N	2.208330	3.132568	0.761008
O	3.933106	1.674416	0.524671
H	3.902845	4.718922	1.846356
H	5.247562	4.009922	0.910739
H	1.854017	4.034829	1.062828
C	1.244589	2.260216	0.121224
H	1.647622	1.866969	-0.816626
H	1.001992	1.395210	0.754166
C	-0.041030	3.042804	-0.151225
N	-0.918255	2.380862	-0.950008
O	-0.239087	4.162489	0.333251
H	-0.665718	1.451538	-1.300078
C	-2.217148	2.932200	-1.291791
H	-2.392572	3.804150	-0.655502
H	-2.247869	3.256428	-2.341384
C	-3.280253	1.844219	-1.129549
N	-4.227818	2.053834	-0.166042
O	-3.251143	0.811319	-1.803064
H	-4.233309	2.937907	0.325409
C	-5.364260	1.149753	-0.016226
H	-6.065725	1.583534	0.703219
H	-5.888753	1.025922	-0.969171
C	-5.038797	-0.277937	0.455689
N	-3.806447	-0.468700	1.013964
O	-5.880716	-1.160983	0.333237
H	-3.177850	0.320684	1.086938
C	-3.329031	-1.777793	1.407940
H	-2.893165	-1.749097	2.411464
H	-4.186862	-2.457511	1.424192
C	-2.256834	-2.398174	0.503490
N	-2.099260	-1.884545	-0.738733
O	-1.593755	-3.355614	0.934588
H	-2.628557	-1.059356	-1.016933
C	-0.999909	-2.300193	-1.604791
H	-0.799776	-3.361481	-1.437315
H	-1.295836	-2.141383	-2.646106
C	0.234316	-1.424169	-1.318309
N	1.343809	-2.057704	-0.870002
O	0.161740	-0.195535	-1.453275
H	1.361996	-3.069773	-0.825210
C	2.461857	-1.342841	-0.267606
H	2.271095	-0.282192	-0.418843
C	3.791172	-1.752664	-0.925960
N	4.754955	-0.801459	-0.836541
O	3.931265	-2.869232	-1.429767
H	4.523133	0.088510	-0.395355
C	6.111219	-1.021131	-1.313674
H	6.178137	-2.042274	-1.694789
H	6.361727	-0.322665	-2.121932
H	6.836612	-0.891247	-0.501151
C	2.555631	-1.616483	1.272418
H	3.129772	-2.527549	1.462667
H	3.045279	-0.761883	1.745670
C	1.145979	-1.785326	1.811227
O	0.837281	-3.079984	1.979885
O	0.356728	-0.865619	1.978635
H	-0.147181	-3.183134	1.947301

-1724.86975

D Hairpin/trans

H	-0.431986	6.330242	-2.193606
C	-0.318698	6.206876	-1.112018
C	-0.639526	4.768581	-0.751066
N	0.334098	4.074016	-0.120582
O	-1.749568	4.279040	-1.027295
H	0.688663	6.513104	-0.814822
H	-1.052666	6.858598	-0.627545
H	1.232589	4.493610	0.099898
C	0.163298	2.712844	0.348091
H	-0.719575	2.632398	0.993995
H	0.008264	2.017245	-0.486624
C	1.418467	2.302620	1.124765
N	1.324511	1.110322	1.753985
O	2.423656	3.030969	1.165317
H	0.580485	0.452509	1.494359
C	2.419985	0.579895	2.554132
H	2.947991	1.417221	3.021226
H	2.012169	-0.075273	3.326909
C	3.394840	-0.263100	1.719047
N	4.140198	0.439885	0.815915
O	3.460951	-1.490587	1.828068
H	4.046072	1.452425	0.809585
C	5.159879	-0.221167	0.009371
H	5.709367	0.540911	-0.552555
H	5.872803	-0.757093	0.643937
C	4.641645	-1.269581	-0.993469
N	3.332650	-1.136971	-1.362352
O	5.394058	-2.135095	-1.427001
H	2.799794	-0.386284	-0.942641
C	2.632668	-2.119384	-2.170851
H	2.300615	-1.699437	-3.126184
H	3.338760	-2.930592	-2.379186
C	1.376613	-2.683331	-1.489618
N	1.444643	-2.864101	-0.137165
O	0.371167	-2.959019	-2.145317
H	2.243768	-2.521503	0.390880
C	0.240671	-3.220644	0.589484
H	-0.216215	-4.110988	0.145695
H	0.515307	-3.442286	1.626200
C	-0.761249	-2.054652	0.602589
N	-2.049435	-2.344337	0.350797
O	-0.386323	-0.892952	0.843938
H	-2.372402	-3.283804	0.135484
C	-3.049497	-1.294314	0.290969
H	-2.888404	-0.617569	1.135342
C	-4.436699	-1.946591	0.430491
N	-5.435483	-1.049419	0.614219
O	-4.577104	-3.170851	0.355255
H	-5.174489	-0.063041	0.621253
C	-6.836290	-1.433498	0.701721
H	-6.897377	-2.520613	0.619600
H	-7.267153	-1.122803	1.661114
H	-7.418826	-0.979832	-0.109433
C	-2.928342	-0.465870	-1.024553
H	-3.689627	-0.775250	-1.751409
H	-1.955313	-0.640089	-1.486120
C	-3.095187	1.021485	-0.760018
O	-3.849353	1.469457	0.099222
O	-2.317544	1.764096	-1.542183
H	-2.305868	2.730939	-1.273498

-1724.8774441

D⁻ Helix/-gauche

H	6.217024	0.143766	-0.457327
C	6.511487	-0.905737	-0.344554
C	5.333348	-1.762332	0.100561
N	4.513008	-1.147361	1.017861
O	5.150093	-2.914934	-0.296984
H	6.892047	-1.299310	-1.289711
H	7.310254	-0.967601	0.405127
H	4.640560	-0.143672	1.127805
C	3.232068	-1.716393	1.393485
H	3.186126	-2.730210	0.983547
H	3.129472	-1.794403	2.483614
C	2.001919	-0.927061	0.915246
N	2.247644	0.084906	0.061285
O	0.869686	-1.252483	1.327086
H	3.234892	0.476906	-0.056872
C	1.320125	1.199844	-0.130009
H	1.017894	1.607460	0.843840
C	0.011189	0.789044	-0.803802
N	0.083491	-0.310663	-1.610065
O	-1.044274	1.409900	-0.627978
H	0.956155	-0.824640	-1.602181
C	-1.110711	-0.899442	-2.180950
H	-1.644357	-0.168474	-2.796246
H	-0.825384	-1.737528	-2.825582
C	-2.149540	-1.407043	-1.164221
N	-1.727374	-1.602306	0.106087
O	-3.316616	-1.601258	-1.536616
H	-0.762572	-1.395761	0.387248
C	-2.669885	-1.939023	1.157833
H	-3.263134	-2.817412	0.885301
H	-2.105107	-2.173547	2.065072
C	-3.702033	-0.850510	1.511998
N	-3.474297	0.396541	1.011523
O	-4.669138	-1.143177	2.217152
H	-2.639465	0.586636	0.459712
C	-4.461724	1.450057	1.130737
H	-5.059709	1.280351	2.030417
H	-3.965161	2.420975	1.224580
C	-5.458469	1.578423	-0.038303
N	-5.346722	0.665779	-1.040683
O	-6.296794	2.481816	-0.028341
H	-4.668792	-0.092318	-1.044231
H	-6.010472	0.733413	-1.799267
C	2.058120	2.300185	-0.939065
H	1.457140	3.211597	-0.939835
H	2.162534	1.963220	-1.980229
C	3.505893	2.652652	-0.434468
O	4.242995	1.641333	-0.106142
O	3.831432	3.845841	-0.444896

-1476.977113

D⁻ Helix/trans

H	-5.891004	-0.118602	1.828398
C	-5.996893	-1.098330	1.350798
C	-4.665661	-1.475682	0.719780
N	-4.657618	-1.587041	-0.649251
O	-3.646169	-1.644891	1.389564
H	-6.228029	-1.825077	2.135444
H	-6.826314	-1.058147	0.636957
H	-5.529630	-1.490526	-1.150210
C	-3.469819	-2.035714	-1.367767
H	-3.069679	-2.945161	-0.908156
H	-3.748031	-2.269201	-2.400531
C	-2.287914	-1.046363	-1.405647
N	-2.559943	0.218364	-1.037506
O	-1.174982	-1.469717	-1.743417
H	-3.518413	0.442445	-0.803574
C	-1.588378	1.326315	-0.901013
H	-1.098118	1.542625	-1.853024
C	-0.460738	0.907887	0.069334
N	-0.812473	0.018148	1.056379
O	0.713540	1.232630	-0.094206
H	-1.772099	-0.290503	1.160132
C	0.179728	-0.454802	2.000394
H	0.684996	0.382485	2.493844
H	-0.319855	-1.052815	2.769882
C	1.309312	-1.308129	1.401242
N	1.109539	-1.809260	0.157936
O	2.343796	-1.503597	2.055888
H	0.275797	-1.546098	-0.365790
C	2.180958	-2.467503	-0.565306
H	2.606908	-3.279794	0.031571
H	1.769117	-2.896713	-1.483790
C	3.384218	-1.584727	-0.955526
N	3.238203	-0.241182	-0.802660
O	4.410545	-2.126211	-1.373909
H	2.347904	0.159826	-0.504531
C	4.363800	0.658587	-0.961673
H	5.029284	0.287144	-1.745548
H	3.999370	1.648438	-1.253898
C	5.241536	0.863540	0.289458
N	4.814649	0.272777	1.438273
O	6.260777	1.552496	0.216005
H	3.992898	-0.322969	1.496334
H	5.385862	0.402473	2.261171
C	-2.440054	2.529224	-0.423053
H	-3.220388	2.700267	-1.178548
H	-2.955447	2.247054	0.507282
C	-1.760912	3.932494	-0.154808
O	-0.527408	4.009079	-0.347120
O	-2.602188	4.787638	0.223086

-1476.927283

D^- Hairpin/-gauche

H	-5.982492	2.313993	-1.852273
C	-5.736134	2.617893	-0.829465
C	-4.545221	1.796878	-0.352644
N	-3.445099	2.492059	-0.006433
O	-4.612892	0.557284	-0.290809
H	-5.552231	3.696718	-0.809081
H	-6.599404	2.385573	-0.197368
H	-3.377222	3.501143	-0.114904
C	-2.209681	1.857815	0.411400
H	-2.352283	1.267955	1.324313
H	-1.804564	1.190579	-0.360602
C	-1.161454	2.946413	0.640735
N	0.010944	2.475078	1.125549
O	-1.399864	4.133431	0.373272
H	0.066980	1.476366	1.334166
C	1.178063	3.314430	1.301659
H	0.875024	4.353254	1.149800
H	1.564749	3.227818	2.324712
C	2.388596	3.065498	0.378625
N	2.297335	2.050892	-0.522322
O	3.377783	3.789649	0.524428
H	1.417988	1.530308	-0.684489
C	3.402973	1.760881	-1.433478
H	2.987583	1.567299	-2.430120
H	4.072402	2.622517	-1.458136
C	4.234091	0.541667	-0.980112
N	3.590044	-0.649805	-1.145906
O	5.360686	0.656526	-0.489516
H	2.673638	-0.706226	-1.642282
C	4.136073	-1.901419	-0.665769
H	4.189331	-2.648414	-1.464967
H	5.147979	-1.716636	-0.287750
C	3.302452	-2.546168	0.456351
N	2.622151	-1.652699	1.238070
O	3.285142	-3.764599	0.639205
H	2.613958	-0.691101	0.913854
C	1.578104	-2.057249	2.167191
H	1.593294	-3.150060	2.220167
H	1.753724	-1.636733	3.163509
C	0.240037	-1.502310	1.656857
N	-0.410578	-2.287220	0.768037
O	-0.136931	-0.370777	2.002649
H	-0.045754	-3.211446	0.567018
C	-1.517668	-1.828195	-0.064241
H	-1.809041	-0.851528	0.318655
C	-2.682540	-2.824944	0.037597
N	-3.915070	-2.256644	-0.039111
O	-2.477570	-4.040284	0.139392
H	-4.006177	-1.241277	-0.101115
C	-5.130806	-3.049217	-0.059366
H	-4.849691	-4.104511	-0.030400
H	-5.764323	-2.823750	0.808814
H	-5.711122	-2.853368	-0.970431
C	-1.075675	-1.689906	-1.541632
H	-0.844066	-2.675477	-1.958114
H	-1.908157	-1.258744	-2.112212
C	0.161459	-0.767158	-1.678947
O	0.061597	0.386571	-1.160729
O	1.170271	-1.244718	-2.268459

-1724.3645296

D^- Hairpin/trans

H	-8.382348	-2.368678	0.488635
C	-7.992596	-1.345522	0.474221
C	-6.528874	-1.375623	0.908795
N	-5.648095	-0.868161	0.010192
O	-6.189604	-1.836659	2.002998
H	-8.143319	-0.916437	-0.521851
H	-8.562206	-0.764220	1.206827
H	-5.937050	-0.457554	-0.873908
C	-4.223856	-0.783807	0.250681
H	-4.004911	-0.164853	1.130828
H	-3.792817	-1.774178	0.443131
C	-3.579427	-0.149412	-0.994375
N	-2.231174	-0.110136	-0.961763
O	-4.282550	0.263736	-1.926596
H	-1.732163	-0.467513	-0.147011
C	-1.436783	0.548331	-1.977854
H	-0.651174	-0.114939	-2.355005
H	-2.102744	0.796257	-2.811201
C	-0.827529	1.871421	-1.480811
N	0.409949	2.142684	-1.992765
O	-1.430313	2.638104	-0.725747
H	0.936461	1.386509	-2.479223
C	0.974688	3.468592	-1.825813
H	1.797925	3.590077	-2.539951
H	0.222743	4.235249	-2.037045
C	1.531768	3.828923	-0.436029
N	1.912277	2.766428	0.337481
O	1.654476	4.999904	-0.081342
H	1.904908	1.850667	-0.096887
C	2.550760	2.929471	1.632076
H	3.644770	2.950411	1.560865
H	2.223290	3.890948	2.043633
C	2.209851	1.783395	2.594924
N	0.906824	1.352357	2.558947
O	3.061982	1.299345	3.338308
H	0.315732	1.742774	1.829725
C	0.555411	0.010932	3.029680
H	1.278922	-0.275432	3.796562
H	-0.457361	0.007215	3.441865
C	0.591222	-0.900783	1.794189
N	1.780526	-1.457341	1.500030
O	-0.400959	-0.968600	1.049491
H	2.558484	-1.445250	2.157019
C	2.112230	-1.974816	0.173453
H	1.307372	-2.630966	-0.173036
C	3.413606	-2.793633	0.336500
N	3.811160	-3.401685	-0.797556
O	3.997558	-2.841806	1.431864
H	3.292644	-3.115550	-1.663279
C	5.058163	-4.138442	-0.878769
H	5.473538	-4.242738	0.126773
H	4.890280	-5.136139	-1.304713
H	5.788349	-3.614638	-1.511116
C	2.256739	-0.789964	-0.819849
H	3.226446	-0.297476	-0.659946
H	1.471957	-0.070938	-0.570905
C	2.110510	-1.079813	-2.343634
O	2.396707	-2.235185	-2.764069
O	1.716040	-0.095980	-3.035790

-1724.3491477

E Helix/-gauche

H	4.975979	-1.690227	-2.359609
C	5.033484	-2.423029	-1.548783
C	3.815809	-2.259935	-0.658531
N	4.057598	-1.956963	0.654907
O	2.664635	-2.373610	-1.092231
H	5.001477	-3.419479	-2.000548
H	5.982466	-2.296926	-1.018703
H	5.015223	-1.903708	0.972823
C	2.981660	-1.922660	1.637207
H	2.450426	-2.879666	1.671404
H	3.414404	-1.740271	2.626538
C	1.898017	-0.864800	1.384460
N	2.247524	0.198238	0.621966
O	0.769932	-1.019279	1.864426
H	3.197818	0.286317	0.267410
C	1.269789	1.205467	0.231787
H	0.805425	1.631324	1.129724
C	0.075922	0.629398	-0.562119
N	0.253604	-0.556413	-1.195988
O	-0.983176	1.265378	-0.597890
H	1.122518	-1.079455	-1.094784
C	-0.859720	-1.195829	-1.878976
H	-1.279257	-0.534537	-2.642969
H	-0.492096	-2.100545	-2.372788
C	-2.048799	-1.586331	-0.982752
N	-1.819697	-1.669511	0.352628
O	-3.151194	-1.800672	-1.497954
H	-0.903911	-1.444529	0.734910
C	-2.908112	-1.918965	1.283578
H	-3.475739	-2.804556	0.982629
H	-2.485023	-2.105992	2.274947
C	-3.954077	-0.795130	1.425011
N	-3.651740	0.401816	0.845042
O	-4.993776	-1.021030	2.041822
H	-2.762621	0.528005	0.369094
C	-4.622808	1.475375	0.759429
H	-5.382961	1.318390	1.530069
H	-4.144025	2.442579	0.942388
C	-5.368392	1.604515	-0.583086
N	-5.186596	0.599677	-1.483852
O	-6.087454	2.581976	-0.786933
H	-4.600637	-0.211753	-1.313540
H	-5.701009	0.659973	-2.351535
C	1.914397	2.336037	-0.591727
H	2.362421	1.922029	-1.503296
H	1.093383	2.987724	-0.905205
C	2.950036	3.198123	0.151422
H	2.658053	3.358470	1.198556
H	3.004755	4.198255	-0.293439
C	4.363380	2.648293	0.159467
O	4.704342	1.511208	-0.135881
O	5.258835	3.582979	0.542165
H	6.137405	3.161896	0.534482

-1516.8130869

E Helix/trans

H	-5.598174	-1.259274	2.293633
C	-5.671159	-2.047164	1.538651
C	-4.384449	-2.081595	0.735635
N	-4.519030	-2.093560	-0.633381
O	-3.273148	-2.081421	1.270750
H	-5.773273	-3.000894	2.068004
H	-6.563270	-1.884122	0.926003
H	-5.448563	-2.213724	-1.014299
C	-3.381554	-2.410723	-1.494706
H	-2.935228	-3.373907	-1.224964
H	-3.735197	-2.476902	-2.529070
C	-2.227431	-1.400168	-1.454964
N	-2.530897	-0.139933	-1.054329
O	-1.092396	-1.753281	-1.783219
H	-3.479541	0.051727	-0.757175
C	-1.508127	0.888073	-0.870685
H	-0.965691	1.022521	-1.813208
C	-0.418205	0.484837	0.144707
N	-0.734215	-0.452165	1.074349
O	0.692808	1.025309	0.086357
H	-1.633720	-0.928909	1.052641
C	0.276482	-0.932648	2.004174
H	0.696797	-0.105157	2.583321
H	-0.195946	-1.632303	2.700490
C	1.485896	-1.636875	1.362739
N	1.338821	-2.092937	0.092385
O	2.529641	-1.758530	2.011873
H	0.475426	-1.925943	-0.418217
C	2.461106	-2.671907	-0.628647
H	2.932883	-3.458922	-0.032921
H	2.089104	-3.121558	-1.553921
C	3.603431	-1.706211	-1.003090
N	3.368238	-0.375201	-0.819393
O	4.655009	-2.168580	-1.441624
H	2.466648	-0.060509	-0.471781
C	4.419405	0.613622	-0.964321
H	5.217549	0.182876	-1.575620
H	4.042121	1.506070	-1.473691
C	5.070282	1.100274	0.345123
N	4.739195	0.425356	1.480863
O	5.848421	2.052676	0.319555
H	4.110426	-0.371142	1.499543
H	5.192909	0.712795	2.336843
C	-2.199751	2.206347	-0.471120
H	-2.662789	2.098298	0.517263
H	-3.013978	2.394401	-1.182136
C	-1.260927	3.416866	-0.465191
H	-0.829029	3.596069	-1.457499
H	-0.405076	3.262814	0.200887
C	-1.982553	4.676075	-0.030053
O	-3.136735	4.741780	0.350055
O	-1.175257	5.763076	-0.107072
H	-1.700616	6.523735	0.199506

-1516.8071698

E **Hairpin/-gauche**

H	5.506011	3.681620	1.498741
C	4.830351	4.096101	0.743706
C	3.772793	3.057270	0.414171
N	2.485312	3.423971	0.607279
O	4.079109	1.931057	-0.008293
H	4.411387	5.037206	1.112420
H	5.422124	4.293014	-0.156041
H	2.219907	4.350912	0.927265
C	1.386367	2.529394	0.317460
H	1.406625	2.205896	-0.730610
H	1.441599	1.618047	0.923133
C	0.067154	3.253753	0.602684
N	-1.032018	2.487744	0.409610
O	0.036865	4.432422	0.973442
H	-0.918994	1.515558	0.102590
C	-2.359504	3.025484	0.611600
H	-2.586751	3.141566	1.681959
H	-2.425669	4.027800	0.166901
C	-3.393562	2.124313	-0.062905
N	-4.586889	2.004332	0.599735
O	-3.170293	1.527890	-1.116186
H	-4.735840	2.558742	1.432881
C	-5.729759	1.337915	-0.019608
H	-6.620667	1.534435	0.584561
H	-5.908722	1.730701	-1.025596
C	-5.612207	-0.188605	-0.192981
N	-4.614187	-0.801587	0.509225
O	-6.404930	-0.774010	-0.922544
H	-4.007291	-0.220872	1.072450
C	-4.264161	-2.199354	0.325685
H	-4.197445	-2.719651	1.286251
H	-5.062918	-2.663657	-0.261480
C	-2.928295	-2.445931	-0.398382
N	-2.505322	-1.461736	-1.243429
O	-2.321710	-3.503552	-0.231517
H	-2.964128	-0.555175	-1.248264
C	-1.226849	-1.573490	-1.908476
H	-1.067952	-2.614082	-2.206162
H	-1.239706	-0.949085	-2.809867
C	-0.056609	-1.087023	-1.034424
N	1.124760	-1.695921	-1.260026
O	-0.201796	-0.170294	-0.208139
H	1.213863	-2.404311	-1.984226
C	2.393941	-1.271960	-0.672561
H	2.275571	-0.247910	-0.310587
C	3.447565	-1.338083	-1.794701
N	4.420946	-0.401525	-1.718978
O	3.377153	-2.227368	-2.651658
H	4.322433	0.361168	-1.048510
C	5.529060	-0.356314	-2.661131
H	5.503323	-1.263467	-3.268148
H	5.450983	0.515112	-3.323894
H	6.483935	-0.304255	-2.125301
C	2.836909	-2.185271	0.492614
H	3.814545	-1.842520	0.849501
H	2.978897	-3.204661	0.117118
C	1.837801	-2.192638	1.653085
H	0.855438	-2.560511	1.332779
H	1.663450	-1.182453	2.043986
C	2.296635	-3.062716	2.804779
O	3.307773	-3.735850	2.842611
O	1.419516	-3.012658	3.845744
H	1.778488	-3.604284	4.530874

-1764.1875469

E **Hairpin/trans**

H	5.373150	3.798446	0.280576
C	4.586771	4.051114	-0.437867
C	3.529960	2.961658	-0.406216
N	2.269474	3.348544	-0.111515
O	3.815436	1.774009	-0.637431
H	4.190321	5.043745	-0.204469
H	5.042661	4.070037	-1.433062
H	2.017049	4.318264	0.057391
C	1.172414	2.406396	-0.067474
H	1.059499	1.888236	-1.027517
H	1.341358	1.630365	0.687562
C	-0.120896	3.161319	0.254568
N	-1.203915	2.357206	0.365221
O	-0.140559	4.389199	0.392898
H	-1.094625	1.351220	0.198036
C	-2.523202	2.895109	0.602069
H	-2.713920	3.044642	1.676110
H	-2.600649	3.881954	0.127163
C	-3.584761	1.970424	0.002827
N	-4.783930	1.952704	0.667527
O	-3.379324	1.271227	-0.988056
H	-4.919299	2.593665	1.438852
C	-5.949911	1.286539	0.092476
H	-6.836265	1.571988	0.667357
H	-6.103163	1.599372	-0.945307
C	-5.900353	-0.252424	0.051182
N	-4.942126	-0.847955	0.820588
O	-6.710895	-0.861369	-0.638532
H	-4.322189	-0.250396	1.350949
C	-4.678542	-2.275362	0.783096
H	-4.654091	-2.699624	1.792027
H	-5.500348	-2.748180	0.236524
C	-3.361110	-2.693215	0.105865
N	-2.782908	-1.776244	-0.723371
O	-2.905616	-3.817953	0.306516
H	-3.174155	-0.843352	-0.817802
C	-1.532171	-2.062213	-1.386457
H	-1.396038	-3.147603	-1.402089
H	-1.571650	-1.702418	-2.423054
C	-0.322868	-1.382684	-0.720701
N	0.869876	-1.949901	-0.993151
O	-0.438852	-0.353461	-0.036345
H	0.938496	-2.803802	-1.540390
C	2.149680	-1.386775	-0.571179
H	2.050526	-0.300000	-0.525251
C	3.178215	-1.784666	-1.645670
N	4.125567	-0.851851	-1.904618
O	3.108670	-2.894796	-2.186942
H	4.051868	0.061080	-1.455687
C	5.200794	-1.078460	-2.859272
H	5.161881	-2.121227	-3.180580
H	5.090212	-0.432491	-3.739521
H	6.174241	-0.876407	-2.397239
C	2.546381	-1.915299	0.827245
H	2.630084	-3.007315	0.785375
H	1.730303	-1.682074	1.518151
C	3.849373	-1.307069	1.359054
H	3.851790	-0.213785	1.244326
H	4.727499	-1.662764	0.809542
C	4.065385	-1.598241	2.830599
O	5.328704	-1.271400	3.221062
O	3.245289	-2.047593	3.606694
H	5.377403	-1.468886	4.173472

-1764.187482

E^- Helix/-gauche

H	-4.564071	-1.819032	2.711941
C	-4.902308	-1.664645	1.685482
C	-3.770770	-2.006329	0.732263
N	-4.055195	-1.784561	-0.593980
O	-2.679702	-2.455425	1.110645
H	-5.773948	-2.299369	1.486714
H	-5.195703	-0.619266	1.529738
H	-4.744407	-1.048653	-0.748736
C	-2.976862	-1.879664	-1.570955
H	-2.517787	-2.870133	-1.522190
H	-3.397740	-1.746497	-2.574207
C	-1.834364	-0.864857	-1.373323
N	-2.181194	0.241739	-0.704024
O	-0.689170	-1.122587	-1.799137
H	-3.204548	0.467241	-0.452669
C	-1.244118	1.287439	-0.342001
H	-0.772582	1.720529	-1.234605
C	-0.067664	0.763319	0.498216
N	-0.286718	-0.370627	1.218101
O	1.021599	1.356218	0.509317
H	-1.161050	-0.880379	1.105833
C	0.799578	-1.020650	1.924454
H	1.240957	-0.352209	2.670444
H	0.400558	-1.898245	2.443188
C	1.976084	-1.476326	1.044188
N	1.736488	-1.622225	-0.279609
O	3.084222	-1.682338	1.563649
H	0.816162	-1.410955	-0.678303
C	2.814731	-1.918081	-1.204111
H	3.366769	-2.809668	-0.890215
H	2.380987	-2.112291	-2.189684
C	3.884163	-0.820709	-1.368868
N	3.591860	0.402446	-0.844727
O	4.935966	-1.085976	-1.954580
H	2.691794	0.571201	-0.396459
C	4.586611	1.452717	-0.777572
H	5.310466	1.311930	-1.585213
H	4.111560	2.430480	-0.906615
C	5.399956	1.531458	0.529625
N	5.153067	0.573645	1.462378
O	6.223441	2.436684	0.681738
H	4.478049	-0.178493	1.341193
H	5.692455	0.614541	2.315600
C	-2.005181	2.393626	0.426507
H	-2.356836	1.984209	1.382004
H	-1.272926	3.175876	0.658369
C	-3.198385	3.019846	-0.336354
H	-2.949285	3.106777	-1.404729
H	-3.360049	4.042444	0.019419
C	-4.587496	2.306967	-0.245573
O	-4.572970	1.017591	-0.118955
O	-5.594742	3.024884	-0.320135

-1516.3056826

E^- Helix/trans

H	-5.492558	-1.242789	1.726085
C	-5.396083	-2.279970	1.386071
C	-4.043813	-2.443925	0.713448
N	-4.062245	-2.576375	-0.653283
O	-2.985133	-2.419343	1.344562
H	-5.427764	-2.923959	2.269298
H	-6.241293	-2.514098	0.730599
H	-4.953185	-2.634323	-1.126465
C	-2.834593	-2.795294	-1.409573
H	-2.287181	-3.657722	-1.015873
H	-3.095069	-3.001643	-2.452889
C	-1.828728	-1.627956	-1.382747
N	-2.332640	-0.426456	-1.049405
O	-0.640030	-1.854420	-1.645656
H	-3.328090	-0.367572	-0.872836
C	-1.578232	0.816991	-0.816610
H	-1.171843	1.193240	-1.761464
C	-0.365884	0.576085	0.092930
N	-0.541636	-0.308497	1.120315
O	0.705595	1.163802	-0.087437
H	-1.402516	-0.842520	1.191262
C	0.537605	-0.596525	2.044963
H	0.894030	0.320610	2.525204
H	0.162142	-1.263926	2.827433
C	1.790239	-1.244890	1.432744
N	1.660099	-1.801356	0.204681
O	2.854912	-1.234207	2.066958
H	0.781577	-1.718838	-0.306776
C	2.814424	-2.301848	-0.517940
H	3.373278	-3.017524	0.092830
H	2.464510	-2.818365	-1.416852
C	3.852295	-1.249169	-0.954897
N	3.486212	0.057710	-0.844341
O	4.948204	-1.626317	-1.374007
H	2.549326	0.308914	-0.531294
C	4.443383	1.126444	-1.051733
H	5.187380	0.809734	-1.787564
H	3.931326	2.013565	-1.437436
C	5.229729	1.579994	0.194704
N	4.933914	0.954586	1.366144
O	6.067592	2.477389	0.092796
H	4.262767	0.195531	1.449519
H	5.452685	1.242118	2.183956
C	-2.582786	1.826504	-0.220412
H	-2.876739	1.475328	0.779738
H	-3.498283	1.824744	-0.832343
C	-2.174180	3.297390	-0.162298
H	-1.620168	3.572318	-1.071441
H	-1.522517	3.529100	0.686368
C	-3.455631	4.227893	-0.102498
O	-4.531509	3.697822	-0.508927
O	-3.243907	5.389081	0.319867

-1516.2541666

E^{-} Hairpin/-gauche

H	5.400918	1.864055	1.779077
C	5.608869	2.236672	0.770225
C	4.503624	1.747832	-0.157619
N	3.721049	2.721187	-0.698416
O	4.362977	0.541826	-0.397760
H	5.697663	3.326997	0.803350
H	6.559105	1.803025	0.443689
H	3.733169	3.634866	-0.253654
C	2.466812	2.441211	-1.384959
H	2.478432	2.881347	-2.391556
H	2.327416	1.364511	-1.489578
C	1.326183	3.095606	-0.584362
N	0.116804	2.510682	-0.705852
O	1.552529	4.096917	0.111463
H	0.039804	1.645466	-1.243987
C	-0.995595	2.942287	0.124259
H	-0.808552	2.704875	1.179261
H	-1.095141	4.032952	0.059157
C	-2.325453	2.329039	-0.317706
N	-3.219082	2.210231	0.710519
O	-2.574092	2.051727	-1.495961
H	-2.801241	1.914821	1.606175
C	-4.565285	1.727486	0.439519
H	-5.187174	1.928389	1.319890
H	-4.987144	2.271362	-0.409032
C	-4.712226	0.234074	0.092792
N	-3.731623	-0.572923	0.578603
O	-5.674625	-0.154511	-0.577379
H	-3.053479	-0.206437	1.289715
C	-3.712513	-1.988618	0.278610
H	-3.501089	-2.574208	1.178564
H	-4.692646	-2.283271	-0.113865
C	-2.646893	-2.394149	-0.752524
N	-2.291085	-1.433723	-1.654783
O	-2.161413	-3.531342	-0.760509
H	-2.622555	-0.483373	-1.510462
C	-1.149842	-1.629137	-2.540318
H	-1.110660	-2.685585	-2.821923
H	-1.264130	-1.003105	-3.428235
C	0.137679	-1.195702	-1.819440
N	0.615600	-2.080428	-0.916595
O	0.630081	-0.072289	-2.025743
H	0.095557	-2.944532	-0.775432
C	1.580792	-1.713147	0.125856
H	2.028343	-0.765709	-0.183358
C	2.659702	-2.800506	0.211039
N	3.936719	-2.324064	0.194802
O	2.365365	-3.995228	0.314411
H	4.092556	-1.332934	0.017603
C	5.087252	-3.199997	0.312172
H	4.723789	-4.209325	0.519324
H	5.673982	-3.218621	-0.616722
H	5.743328	-2.877493	1.131306
C	0.867640	-1.531766	1.489717
H	1.613853	-1.347615	2.271052
H	0.362389	-2.468613	1.754470
C	-0.125677	-0.365590	1.446554
H	-0.841921	-0.505864	0.631407
H	0.426453	0.555332	1.210191
C	-0.912239	-0.072399	2.752744
O	-0.375909	-0.322841	3.842782
O	-2.078250	0.458588	2.559481

-1763.6662613

E^{-} Hairpin/trans

H	5.899945	3.307379	-0.757433
C	5.103710	3.543997	-1.471280
C	3.988286	2.525657	-1.294238
N	2.811470	3.003614	-0.823606
O	4.158308	1.328333	-1.562666
H	4.775552	4.575916	-1.310723
H	5.517355	3.443689	-2.479371
H	2.678836	3.975720	-0.563194
C	1.705815	2.123262	-0.516643
H	1.355456	1.600752	-1.415875
H	2.014531	1.359715	0.206778
C	0.555822	2.937017	0.076882
N	-0.510815	2.174886	0.422904
O	0.630382	4.161217	0.230299
H	-0.503813	1.173858	0.205759
C	-1.708060	2.744005	0.984057
H	-1.842614	2.424641	2.028733
H	-1.590523	3.834469	0.979549
C	-2.951126	2.386810	0.157561
N	-4.099653	2.205221	0.906147
O	-2.945113	2.266527	-1.059784
H	-4.068925	2.416029	1.895101
C	-5.391924	2.051224	0.255226
H	-6.179635	2.469952	0.890127
H	-5.390262	2.598623	-0.691973
C	-5.815982	0.612875	-0.094049
N	-4.997238	-0.380919	0.357996
O	-6.844385	0.422027	-0.739302
H	-4.226314	-0.112654	0.957529
C	-5.293066	-1.788754	0.147952
H	-5.731855	-2.250419	1.041208
H	-6.039795	-1.849956	-0.649630
C	-4.093949	-2.670517	-0.229276
N	-2.971758	-2.028595	-0.643623
O	-4.192886	-3.895501	-0.142541
H	-2.921858	-1.015258	-0.666206
C	-1.721681	-2.723155	-0.866882
H	-1.698038	-3.625794	-0.247037
H	-1.620621	-3.045762	-1.913511
C	-0.551535	-1.786631	-0.522911
N	0.660927	-2.346632	-0.579232
O	-0.768158	-0.588825	-0.254563
H	0.781436	-3.334220	-0.794909
C	1.917207	-1.652200	-0.270570
H	1.834068	-0.629865	-0.646704
C	3.001659	-2.402725	-1.056319
N	4.019664	-1.636469	-1.511326
O	2.904822	-3.626078	-1.242131
H	3.997870	-0.629101	-1.354899
C	5.182247	-2.198112	-2.176165
H	5.045813	-3.279245	-2.248955
H	5.295940	-1.779484	-3.184507
H	6.095388	-1.987657	-1.605330
C	2.175659	-1.635717	1.258532
H	2.255271	-2.671270	1.608020
H	1.285026	-1.223264	1.744125
C	3.405850	-0.853895	1.725165
H	3.349501	0.203575	1.425441
H	4.326067	-1.245558	1.275241
C	3.600485	-0.884591	3.294233
O	4.662242	-0.335991	3.685750
O	2.677480	-1.433441	3.954960

-1763.6242014

F Helix/-gauche

H	4.336878	-3.309200	-2.307964
C	4.430390	-3.678637	-1.283017
C	3.299726	-3.106734	-0.447127
N	3.653161	-2.578309	0.772197
O	2.129997	-3.100950	-0.837258
H	4.319679	-4.768236	-1.315188
H	5.424721	-3.435519	-0.895363
H	4.603356	-2.717219	1.090198
C	2.637305	-2.256192	1.771817
H	2.019404	-3.129106	2.008513
H	3.141153	-1.934904	2.689729
C	1.650539	-1.155764	1.361868
N	2.076875	-0.269777	0.429230
O	0.528406	-1.116456	1.873742
H	3.033148	-0.331089	0.101576
C	1.217465	0.793937	-0.075923
H	0.869394	1.408719	0.761462
C	-0.076686	0.272914	-0.731691
N	-0.077914	-1.003860	-1.194612
O	-1.049974	1.027832	-0.823328
H	0.720387	-1.614660	-1.033360
C	-1.293405	-1.584051	-1.743606
H	-1.664667	-0.986751	-2.581671
H	-1.064546	-2.588057	-2.113971
C	-2.477058	-1.691737	-0.765404
N	-2.197797	-1.609201	0.560361
O	-3.619370	-1.840628	-1.211816
H	-1.245069	-1.446741	0.878038
C	-3.264757	-1.584246	1.547544
H	-3.951316	-2.421402	1.390209
H	-2.824176	-1.687364	2.543522
C	-4.155937	-0.326006	1.556974
N	-3.741570	0.727191	0.795688
O	-5.181477	-0.326905	2.235654
H	-2.870113	0.669428	0.275932
C	-4.581436	1.889991	0.581291
H	-5.312418	1.944887	1.392923
H	-3.981768	2.805589	0.597579
C	-5.377412	1.912501	-0.738379
N	-5.350396	0.777004	-1.489849
O	-5.995357	2.927379	-1.057283
H	-4.856081	-0.067014	-1.218445
H	-5.901648	0.771879	-2.336764
C	1.984538	1.681477	-1.082613
C	3.236226	2.308155	-0.498351
H	2.242077	1.080832	-1.964169
H	1.282442	2.454327	-1.412906
C	4.510270	1.923813	-0.949466
H	4.598528	1.191209	-1.751945
C	5.670631	2.493767	-0.402573
H	6.648986	2.192150	-0.771892
C	5.568607	3.458805	0.607077
H	6.465281	3.906930	1.030243
C	4.300229	3.853524	1.061219
H	4.212416	4.610909	1.837613
C	3.146183	3.283150	0.512337
H	2.167153	3.604898	0.865472

-1519.9488645

F Helix/trans

H	-5.954326	-0.505816	2.346365
C	-6.155501	-1.204399	1.529566
C	-4.883464	-1.406537	0.726860
N	-5.010551	-1.339425	-0.641858
O	-3.790928	-1.606260	1.262008
H	-6.435993	-2.163578	1.978966
H	-6.994599	-0.836213	0.930913
H	-5.946228	-1.294636	-1.024151
C	-3.939622	-1.809085	-1.519538
H	-3.659027	-2.841652	-1.285291
H	-4.299385	-1.780904	-2.553470
C	-2.631559	-1.008674	-1.454964
N	-2.711909	0.261106	-0.985741
O	-1.578113	-1.533169	-1.822902
H	-3.613721	0.590682	-0.664919
C	-1.529628	1.093033	-0.755923
H	-1.000600	1.245659	-1.701922
C	-0.495151	0.417691	0.168942
N	-0.970448	-0.456931	1.098877
O	0.701430	0.691327	0.057212
H	-1.945904	-0.746695	1.090894
C	-0.051823	-1.165325	1.976155
H	0.553685	-0.459048	2.552322
H	-0.635980	-1.765734	2.680438
C	0.963907	-2.095422	1.289248
N	0.718180	-2.454078	0.003833
O	1.955324	-2.473508	1.922636
H	-0.080161	-2.068296	-0.493670
C	1.693068	-3.220232	-0.756117
H	1.983482	-4.120803	-0.207220
H	1.236373	-3.529128	-1.701024
C	3.017417	-2.502385	-1.086847
N	3.066077	-1.160834	-0.848085
O	3.952963	-3.158291	-1.542200
H	2.244545	-0.673438	-0.500256
C	4.304193	-0.412715	-0.953192
H	4.987957	-0.961179	-1.607177
H	4.120515	0.571426	-1.395573
C	5.056586	-0.164934	0.368899
N	4.577536	-0.798764	1.475233
O	6.038812	0.575894	0.379325
H	3.783481	-1.431115	1.463114
H	5.092844	-0.674857	2.335602
C	-2.000779	2.460612	-0.197358
C	-0.913630	3.512441	-0.103836
H	-2.455775	2.297105	0.788222
H	-2.794030	2.819680	-0.867802
C	-0.494069	4.200416	-1.255382
H	-0.954212	3.975309	-2.217521
C	0.511174	5.172054	-1.181593
H	0.828409	5.693926	-2.082282
C	1.107556	5.473865	0.051333
H	1.890976	6.226636	0.110562
C	0.690539	4.798231	1.205533
H	1.150163	5.024846	2.165375
C	-0.314726	3.825031	1.126064
H	-0.627521	3.300384	2.027973

-1519.9434707

F Hairpin/-gauche

H	5.871347	3.182219	1.223481
C	5.277355	3.572548	0.390831
C	4.131011	2.613909	0.117670
N	2.883378	3.126399	0.200353
O	4.337988	1.422568	-0.166323
H	4.944608	4.587091	0.629989
H	5.926062	3.604617	-0.490475
H	2.703268	4.100903	0.424080
C	1.707985	2.318810	-0.047138
H	1.758256	1.842794	-1.032561
H	1.618001	1.507346	0.684817
C	0.465612	3.210805	0.036717
N	-0.689914	2.565898	-0.242755
O	0.538099	4.406741	0.343949
H	-0.659655	1.575214	-0.505664
C	-1.963011	3.251142	-0.184651
H	-2.174543	3.603009	0.835054
H	-1.952872	4.142645	-0.828483
C	-3.064158	2.316238	-0.683314
N	-4.248853	2.380318	0.002477
O	-2.899822	1.540958	-1.625090
H	-4.346841	3.069079	0.736919
C	-5.443309	1.702756	-0.494777
H	-6.308679	2.050905	0.077366
H	-5.614587	1.945110	-1.548569
C	-5.435122	0.163914	-0.428809
N	-4.460605	-0.407693	0.337926
O	-6.290887	-0.466612	-1.040583
H	-3.796221	0.201831	0.796417
C	-4.235760	-1.841552	0.382566
H	-4.185409	-2.201301	1.415013
H	-5.088408	-2.323103	-0.106795
C	-2.948851	-2.326932	-0.308127
N	-2.452972	-1.532631	-1.299589
O	-2.446751	-3.403022	0.019035
H	-2.820827	-0.594673	-1.434661
C	-1.192502	-1.857533	-1.936513
H	-1.112287	-2.944212	-2.027033
H	-1.177741	-1.415545	-2.939738
C	0.003813	-1.276785	-1.163656
N	1.095415	-2.065276	-1.080078
O	-0.048473	-0.137758	-0.678877
H	1.121301	-2.962177	-1.555626
C	2.351236	-1.655127	-0.463142
H	2.320493	-0.573687	-0.322060
C	3.490955	-2.057797	-1.414974
N	4.530981	-1.191367	-1.452624
O	3.424875	-3.122716	-2.040569
H	4.452241	-0.299448	-0.962689
C	5.722607	-1.442788	-2.248427
H	5.665954	-2.459427	-2.642775
H	5.792872	-0.740642	-3.089090
H	6.623848	-1.341452	-1.632366
C	2.574227	-2.347706	0.912856
H	3.597171	-2.115971	1.232697
C	1.591323	-1.910830	1.979475
H	2.521060	-3.431168	0.753695
C	0.346582	-2.546606	2.129303
H	0.071367	-3.371488	1.475046
C	-0.558787	-2.124940	3.112721
H	-1.513524	-2.637911	3.213169
C	-0.232747	-1.060400	3.964856
H	-0.930386	-0.741056	4.737282
C	1.006760	-0.418772	3.824178
H	1.274279	0.402519	4.486940
C	1.908665	-0.843287	2.839311
H	2.874289	-0.347241	2.742559

-1767.3282305

F Hairpin/trans

H	4.857508	4.270923	-0.415432
C	3.971769	4.409421	-1.043513
C	3.031546	3.237380	-0.821513
N	1.782355	3.537880	-0.396252
O	3.393847	2.068480	-1.019604
H	3.516126	5.378106	-0.816979
H	4.302569	4.401899	-2.087103
H	1.464212	4.492000	-0.254792
C	0.790541	2.511526	-0.163171
H	0.635402	1.901792	-1.061370
H	1.109143	1.823096	0.628943
C	-0.528813	3.178199	0.237800
N	-1.529105	2.300016	0.493901
O	-0.639045	4.406209	0.316669
H	-1.359380	1.299009	0.347093
C	-2.861521	2.752120	0.816501
H	-3.015680	2.819661	1.904883
H	-2.994360	3.763991	0.412671
C	-3.911982	1.826473	0.198285
N	-5.079170	1.716040	0.910328
O	-3.724541	1.202402	-0.845049
H	-5.206041	2.304340	1.723920
C	-6.241617	1.035300	0.345423
H	-7.112309	1.246695	0.973800
H	-6.454062	1.401223	-0.664152
C	-6.126080	-0.494678	0.209004
N	-5.116892	-1.087952	0.912627
O	-6.929955	-1.100356	-0.491180
H	-4.504831	-0.491514	1.453448
C	-4.772898	-2.491053	0.765220
H	-4.698906	-2.985469	1.739072
H	-5.579151	-2.969825	0.200624
C	-3.447807	-2.769463	0.033096
N	-2.987420	-1.779813	-0.785405
O	-2.883742	-3.853140	0.177417
H	-3.448890	-0.875148	-0.816075
C	-1.734327	-1.925836	-1.487624
H	-1.564015	-2.989415	-1.679753
H	-1.796179	-1.403392	-2.450437
C	-0.536812	-1.327719	-0.726613
N	0.661257	-1.853385	-1.052689
O	-0.667033	-0.387598	0.074624
H	0.725856	-2.675130	-1.646626
C	1.934914	-1.386339	-0.506410
H	1.854237	-0.315467	-0.308223
C	2.996271	-1.681553	-1.580362
N	3.830866	-0.661745	-1.872413
O	3.031612	-2.805976	-2.099607
H	3.673667	0.253057	-1.449194
C	4.914157	-0.794250	-2.834298
H	4.932012	-1.824551	-3.195103
H	4.764195	-0.118690	-3.685544
H	5.875930	-0.556816	-2.364344
C	2.258542	-2.137415	0.815821
H	2.339204	-3.203589	0.576586
C	3.514368	-1.652118	1.509450
H	1.391362	-2.005716	1.472787
C	3.461992	-0.578961	2.416307
H	2.503830	-0.106764	2.632573
C	4.621510	-0.118900	3.054888
H	4.561695	0.710394	3.757550
C	5.855874	-0.731852	2.797247
H	6.757020	-0.381004	3.296769
C	5.919529	-1.804656	1.896467
H	6.872508	-2.290547	1.695037
C	4.757747	-2.258572	1.258353
H	4.813505	-3.092937	0.560389

-1767.3277245

G Helix

H	-7.022157	-1.059381	-0.193498
C	-6.184561	-1.298359	-0.856290
C	-4.948090	-0.458900	-0.594306
N	-5.103888	0.608441	0.260386
C	-4.077924	1.646349	0.349769
C	-2.727951	1.184076	0.913410
N	-2.740573	0.083320	1.711681
C	-1.502143	-0.497670	2.208759
C	-0.525919	-0.995393	1.127342
N	-1.024055	-1.203159	-0.119052
C	-0.131276	-1.548964	-1.213882
C	0.916982	-0.478618	-1.568896
N	0.690922	0.783464	-1.120012
C	1.682734	1.831585	-1.299066
C	2.988902	1.679726	-0.493222
N	3.016893	0.699788	0.454939
C	4.240096	0.344498	1.148867
C	4.997802	-0.878714	0.596866
N	4.542430	-1.405423	-0.573757
H	-6.497191	-1.137777	-1.894064
H	-5.925491	-2.355630	-0.751929
O	-3.854690	-0.718710	-1.101534
H	-6.044420	0.849530	0.545106
H	-3.853341	2.069635	-0.635065
H	-4.451586	2.451570	0.991108
O	-1.701429	1.809617	0.643010
H	-3.615093	-0.410169	1.830056
H	-0.940974	0.238440	2.791578
H	-1.742217	-1.337407	2.868385
O	0.659020	-1.172383	1.422445
H	-1.997149	-0.998138	-0.335666
H	0.442746	-2.449842	-0.977810
H	-0.732375	-1.749291	-2.106127
O	1.911329	-0.797307	-2.228071
H	-0.126259	0.986584	-0.550929
H	1.995082	1.887158	-2.346075
H	1.233562	2.790999	-1.026389
O	3.929898	2.432559	-0.737675
H	2.193390	0.128783	0.623213
H	4.928245	1.192893	1.097052
H	4.035382	0.132994	2.203106
O	5.963899	-1.323615	1.214771
H	3.755051	-1.029571	-1.091982
H	5.056570	-2.185381	-0.959032

-1249.5485782

G Hairpin

H	-6.279868	2.884560	-1.147064
C	-5.683583	3.106032	-0.256051
C	-4.491001	2.166311	-0.222516
N	-3.267404	2.743767	-0.214024
O	-4.636092	0.933609	-0.207039
H	-5.401179	4.163025	-0.265668
H	-6.311059	2.908468	0.618969
H	-3.135522	3.750784	-0.193230
C	-2.054189	1.957535	-0.163259
H	-2.025696	1.338740	0.742894
H	-1.987257	1.271703	-1.014762
C	-0.844113	2.896085	-0.157177
N	0.343017	2.247196	-0.205307
O	-0.970668	4.124112	-0.103332
H	0.352115	1.222357	-0.229545
C	1.597356	2.958984	-0.124171
H	1.905036	3.352781	-1.105208
H	1.480944	3.825251	0.540436
C	2.685099	2.042653	0.441044
N	3.953402	2.294372	-0.017754
O	2.447967	1.130160	1.230066
H	4.101419	3.097680	-0.615044
C	5.112352	1.649131	0.593437
H	6.019429	2.155288	0.249307
H	5.071210	1.731713	1.684288
C	5.285460	0.146512	0.304193
N	4.515434	-0.370166	-0.698120
O	6.095386	-0.501836	0.958189
H	3.878086	0.253471	-1.175161
C	4.476318	-1.787985	-1.007769
H	4.648288	-1.965512	-2.074346
H	5.285919	-2.268235	-0.449400
C	3.165526	-2.512852	-0.655038
N	2.360310	-1.905338	0.264364
O	2.915106	-3.599290	-1.174901
H	2.583832	-0.976014	0.608430
C	1.086734	-2.479223	0.630222
H	1.084441	-3.525649	0.310740
H	0.961061	-2.451734	1.720867
C	-0.106488	-1.729330	0.013703
N	-1.277389	-2.396542	0.035192
O	-0.004564	-0.571069	-0.420169
H	-1.352762	-3.357243	0.361751
C	-2.528084	-1.798394	-0.393080
H	-2.597696	-0.764024	-0.053691
H	-2.604742	-1.786891	-1.489857
C	-3.688300	-2.632366	0.164130
N	-4.871018	-1.973607	0.192841
O	-3.510218	-3.804672	0.513503
H	-4.872067	-0.973428	-0.007317
C	-6.103227	-2.604768	0.640010
H	-5.910660	-3.669225	0.788411
H	-6.449030	-2.172336	1.587600
H	-6.893205	-2.481372	-0.110349

-1496.930

H **Helix/-gauche**

H	4.700410	-2.896438	-2.379259
C	4.804688	-3.302560	-1.369247
C	3.640023	-2.821555	-0.521893
N	3.959680	-2.309169	0.713699
O	2.473958	-2.871882	-0.918403
H	4.748349	-4.393968	-1.446812
H	5.783084	-3.026671	-0.963184
H	4.914288	-2.410409	1.032513
C	2.924882	-2.075119	1.719284
H	2.359755	-2.989515	1.929491
H	3.408293	-1.750554	2.646990
C	1.874192	-1.023463	1.339445
N	2.245390	-0.085329	0.436437
O	0.751556	-1.072496	1.849612
H	3.202307	-0.079605	0.106070
C	1.325718	0.948222	-0.027313
H	0.956896	1.517585	0.831385
C	0.069284	0.376738	-0.710868
N	0.141828	-0.886048	-1.209223
O	-0.946099	1.075028	-0.793682
H	0.972103	-1.455226	-1.060316
C	-1.038278	-1.518333	-1.776088
H	-1.443158	-0.919615	-2.597457
H	-0.753501	-2.497216	-2.174062
C	-2.214463	-1.719974	-0.803638
N	-1.942491	-1.651879	0.524197
O	-3.345556	-1.926082	-1.256774
H	-1.000999	-1.441701	0.848313
C	-3.010870	-1.709541	1.508465
H	-3.645331	-2.583708	1.334558
H	-2.566340	-1.803187	2.503657
C	-3.976228	-0.507257	1.536766
N	-3.619066	0.584758	0.802325
O	-5.005626	-0.584288	2.205577
H	-2.739826	0.591326	0.292255
C	-4.522717	1.702325	0.608295
H	-5.252665	1.705568	1.422657
H	-3.973131	2.648451	0.636309
C	-5.324381	1.700329	-0.708212
N	-5.230122	0.583457	-1.481673
O	-6.005706	2.680654	-1.006148
H	-4.682503	-0.232967	-1.228574
H	-5.782503	0.561069	-2.327508
C	2.039595	1.915560	-0.991607
C	3.212480	2.616961	-0.362093
H	2.368628	1.379212	-1.890807
H	1.289885	2.649454	-1.308309
C	4.462713	2.851598	-0.903893
H	4.899750	2.594659	-1.858541
N	5.144983	3.587386	0.050796
H	6.087131	3.945243	-0.028543
C	4.297252	3.756414	1.112243
H	4.581137	4.299915	2.005315
N	3.128995	3.182362	0.896946

-1513.9302094

H **Helix/trans**

H	-5.928953	-0.010873	2.208042
C	-6.132772	-0.826448	1.508514
C	-4.860075	-1.153280	0.748885
N	-4.974382	-1.249476	-0.619316
O	-3.776226	-1.308497	1.315048
H	-6.416428	-1.703551	2.100550
H	-6.969908	-0.550941	0.859426
H	-5.904772	-1.231536	-1.016055
C	-3.900321	-1.830423	-1.423248
H	-3.625874	-2.825521	-1.056984
H	-4.253645	-1.933163	-2.454624
C	-2.588641	-1.032774	-1.452705
N	-2.667109	0.284425	-1.139868
O	-1.537207	-1.601719	-1.754566
H	-3.565423	0.645011	-0.843354
C	-1.483344	1.130463	-0.983956
H	-0.935017	1.165928	-1.930679
C	-0.461966	0.562775	0.023833
N	-0.940210	-0.204551	1.037907
O	0.738340	0.809069	-0.122338
H	-1.922529	-0.465450	1.078547
C	-0.027740	-0.791298	2.005560
H	0.559793	-0.012994	2.501932
H	-0.614984	-1.313690	2.767111
C	1.005890	-1.780211	1.438540
N	0.760095	-2.307672	0.211334
O	2.009311	-2.054758	2.105524
H	-0.042834	-1.993708	-0.327485
C	1.752716	-3.135076	-0.455081
H	2.075892	-3.947236	0.202522
H	1.299648	-3.578199	-1.346899
C	3.051089	-2.425419	-0.890723
N	3.058184	-1.064071	-0.819250
O	4.003006	-3.104008	-1.274261
H	2.224467	-0.561362	-0.523315
C	4.265639	-0.292182	-1.035749
H	4.989095	-0.923760	-1.559921
H	4.060175	0.584109	-1.658909
C	4.960567	0.231524	0.235641
N	4.539939	-0.291867	1.419844
O	5.856118	1.070897	0.139898
H	3.792504	-0.974323	1.499312
H	5.008305	0.023739	2.257681
C	-1.960066	2.557200	-0.607814
C	-0.852598	3.526039	-0.306555
H	-2.594053	2.496394	0.286023
H	-2.578820	2.924353	-1.436880
C	-0.020696	4.200948	-1.179301
H	0.045552	4.216926	-2.257755
N	0.847484	4.914253	-0.375368
H	1.609081	5.500026	-0.690086
C	0.509260	4.652705	0.924118
H	1.036037	5.089622	1.763378
N	-0.514815	3.822540	0.999607

-1513.9265758

H **Hairpin/-gauche**

H	5.706144	3.275860	1.560556
C	5.133507	3.685557	0.722161
C	4.012173	2.719244	0.381815
N	2.752145	3.199802	0.475859
O	4.248428	1.549796	0.037372
H	4.776945	4.685842	0.985712
H	5.809192	3.757103	-0.136066
H	2.550142	4.159852	0.738606
C	1.599362	2.377196	0.178664
H	1.700001	1.907337	-0.805476
H	1.492706	1.559895	0.902499
C	0.339875	3.248465	0.213720
N	-0.791379	2.589590	-0.133789
O	0.374414	4.439659	0.541312
H	-0.731278	1.604645	-0.409288
C	-2.077861	3.254821	-0.125423
H	-2.303286	3.652632	0.873418
H	-2.080021	4.112411	-0.814306
C	-3.154374	2.274767	-0.589594
N	-4.321050	2.290731	0.127736
O	-2.983264	1.501060	-1.533079
H	-4.428479	2.975852	0.864309
C	-5.492918	1.543343	-0.320889
H	-6.350937	1.834234	0.292947
H	-5.727507	1.777613	-1.364199
C	-5.378479	0.008345	-0.264848
N	-4.362503	-0.494540	0.498605
O	-6.185821	-0.682224	-0.876511
H	-3.737251	0.161029	0.948890
C	-4.019780	-1.904560	0.521102
H	-3.909068	-2.268456	1.546995
H	-4.841679	-2.450771	0.046014
C	-2.713877	-2.251131	-0.212810
N	-2.407990	-1.499822	-1.301456
O	-2.013825	-3.197460	0.170992
H	-2.884181	-0.613413	-1.451550
C	-1.146231	-1.705917	-1.989059
H	-1.023370	-2.768075	-2.221620
H	-1.169132	-1.139058	-2.925728
C	0.034331	-1.183403	-1.153305
N	1.131755	-1.966480	-1.116053
O	-0.048057	-0.099547	-0.559641
H	1.192891	-2.800326	-1.693553
C	2.370655	-1.596364	-0.438240
H	2.312150	-0.538722	-0.176972
C	3.528621	-1.873630	-1.412129
N	4.545758	-0.983587	-1.355313
O	3.492607	-2.876362	-2.136678
H	4.430218	-0.131315	-0.804769
C	5.747395	-1.126231	-2.163795
H	5.749193	-2.122755	-2.610060
H	5.776369	-0.379561	-2.967851
H	6.640717	-1.003788	-1.540823
C	2.586165	-2.436984	0.853571
H	2.593024	-3.497723	0.569664
H	3.584852	-2.205204	1.239693
C	1.602958	-2.175838	1.954818
N	0.281004	-2.589947	1.950880
C	1.764571	-1.538015	3.173757
H	-0.238847	-3.030029	1.193958
C	-0.277687	-2.178034	3.129362
H	-1.311434	-2.383035	3.379483
H	2.670001	-1.088522	3.563884
N	0.590209	-1.541852	3.898135

-1761.3159449

H **Hairpin/trans**

H	5.494763	3.631901	-1.165860
C	4.535418	3.767838	-1.675384
C	3.579989	2.678151	-1.215047
N	2.423284	3.095146	-0.654958
O	3.859859	1.477593	-1.355302
H	4.164362	4.778861	-1.481528
H	4.712352	3.648592	-2.749179
H	2.177679	4.078080	-0.580277
C	1.403872	2.172971	-0.195443
H	1.128724	1.473942	-0.994710
H	1.764651	1.577215	0.654832
C	0.167239	2.978476	0.209006
N	-0.886743	2.211955	0.586367
O	0.161267	4.214617	0.188867
H	-0.822928	1.195736	0.481242
C	-2.170515	2.791718	0.890809
H	-2.379843	2.766842	1.971665
H	-2.145524	3.846218	0.588689
C	-3.301264	2.080901	0.136548
N	-4.537267	2.180150	0.733237
O	-3.132907	1.449059	-0.902320
H	-4.637479	2.790825	1.534151
C	-5.740000	1.759103	0.020758
H	-6.616631	2.167986	0.532762
H	-5.737287	2.140156	-1.005540
C	-5.950409	0.239801	-0.107132
N	-5.154979	-0.545124	0.677754
O	-6.806405	-0.190885	-0.873007
H	-4.489328	-0.084432	1.284348
C	-5.164932	-1.994319	0.608024
H	-5.353124	-2.440698	1.590195
H	-5.984395	-2.283943	-0.057421
C	-3.873631	-2.642251	0.081655
N	-3.046218	-1.842120	-0.651525
O	-3.654275	-3.831601	0.309132
H	-3.248911	-0.852007	-0.754606
C	-1.781886	-2.333600	-1.146150
H	-1.756726	-3.414588	-0.976944
H	-1.696902	-2.155375	-2.227152
C	-0.580714	-1.650586	-0.473104
N	0.611998	-2.243818	-0.692576
O	-0.702223	-0.598875	0.170126
H	0.681827	-3.145144	-1.156775
C	1.877403	-1.688234	-0.218257
H	1.832695	-0.601268	-0.297699
C	2.972142	-2.269203	-1.128284
N	3.912098	-1.392359	-1.541510
O	2.941160	-3.473316	-1.421072
H	3.808177	-0.398048	-1.329833
C	5.024122	-1.790778	-2.390797
H	4.996159	-2.875366	-2.513188
H	4.950925	-1.317099	-3.377688
H	5.978121	-1.498790	-1.936064
C	2.122631	-2.064481	1.268962
H	2.296839	-3.143516	1.336943
H	1.199859	-1.831791	1.812717
C	3.259593	-1.312440	1.895054
C	4.461874	-1.800109	2.366818
N	3.201042	0.059486	2.084228
H	4.874814	-2.798237	2.392356
N	5.144441	-0.697614	2.852468
H	6.062133	-0.699869	3.275696
C	4.342656	0.393325	2.657422
H	4.638063	1.393460	2.950331

-1761.3114864

H+ Helix/-gauche

H	2.701075	-4.451683	-2.333461
C	3.648147	-4.274897	-1.817117
C	3.435224	-3.263380	-0.711222
N	4.397709	-2.286055	-0.574253
O	2.451223	-3.285864	0.034653
H	3.954586	-5.223145	-1.360244
H	4.411830	-3.972581	-2.540106
H	5.237987	-2.384267	-1.130754
C	4.507747	-1.514491	0.661118
H	4.443375	-2.167932	1.539157
H	5.486914	-1.026423	0.685608
C	3.452831	-0.430325	0.903454
N	2.581547	-0.150510	-0.113835
O	3.426720	0.155297	1.987217
H	2.676716	-0.699899	-0.960696
C	1.336509	0.571920	0.115768
H	1.294550	0.738505	1.200315
C	0.066424	-0.243775	-0.246620
N	0.118176	-1.585240	-0.062064
O	-0.945017	0.351042	-0.628439
H	0.996640	-2.052011	0.170430
C	-0.985164	-2.432224	-0.523466
H	-1.143938	-2.313053	-1.599912
H	-0.710818	-3.473207	-0.325889
C	-2.354653	-2.155420	0.118693
N	-2.351698	-1.706805	1.403973
O	-3.383626	-2.354178	-0.524608
H	-1.464631	-1.571885	1.869857
C	-3.592314	-1.399733	2.107358
H	-4.294934	-2.232666	2.009552
H	-3.375729	-1.267559	3.171274
C	-4.362538	-0.153063	1.625767
N	-3.742098	0.627053	0.693187
O	-5.474049	0.072646	2.092650
H	-2.830488	0.356821	0.340506
C	-4.428619	1.704710	0.003723
H	-5.413272	1.823412	0.467192
H	-3.891505	2.653914	0.103709
C	-4.635492	1.477781	-1.505028
N	-4.635052	0.181994	-1.928309
O	-4.808745	2.439201	-2.251101
H	-4.464767	-0.609804	-1.317258
H	-4.829797	0.013750	-2.905856
C	1.239240	1.943464	-0.585423
C	2.296080	2.929007	-0.186541
H	1.269205	1.802049	-1.674273
H	0.248071	2.354720	-0.365754
N	2.439355	4.146497	-0.853554
H	1.892899	4.441034	-1.657763
C	3.385372	4.895440	-0.269022
H	3.703457	5.882953	-0.573574
N	3.859364	4.188022	0.762943
H	4.588598	4.508140	1.392445
C	3.205754	2.965849	0.844914
H	3.423528	2.215778	1.597761

-1514.3056098

H+ Helix/trans

H	5.861457	-2.028007	-1.657696
C	6.351544	-1.408742	-0.902095
C	5.300837	-0.779015	-0.010769
N	5.497606	0.538173	0.331193
O	4.308526	-1.399996	0.387479
H	6.973094	-2.069842	-0.286889
H	7.000472	-0.675428	-1.390547
H	6.376455	0.959966	0.057547
C	4.789123	1.148714	1.455000
H	4.858538	0.521483	2.351013
H	5.262005	2.108221	1.686077
C	3.291042	1.414133	1.270907
N	2.773832	1.263856	0.007436
O	2.607113	1.746544	2.233354
H	3.395401	0.956663	-0.730273
C	1.337618	1.245486	-0.197089
H	0.913892	1.374964	0.806686
C	0.820394	-0.111319	-0.734690
N	1.537729	-1.220995	-0.434612
O	-0.239027	-0.159599	-1.374371
H	2.452984	-1.147154	0.016068
C	1.212353	-2.495050	-1.080188
H	1.280453	-2.410265	-2.169273
H	1.947110	-3.233990	-0.744111
C	-0.194298	-3.053677	-0.811383
N	-0.715191	-2.812997	0.438350
O	-0.778188	-3.700960	-1.668620
H	-0.125707	-2.370272	1.131927
C	-2.019035	-3.319018	0.824069
H	-2.366062	-3.985109	0.027694
H	-1.970574	-3.904370	1.749210
C	-3.091001	-2.249382	1.050339
N	-2.772473	-0.970363	0.695167
O	-4.179989	-2.539003	1.556087
H	-1.942389	-0.804729	0.131885
C	-3.637066	0.137137	1.070401
H	-4.061962	-0.082088	2.056790
H	-3.038837	1.046258	1.145919
C	-4.812798	0.402799	0.109251
N	-5.707874	-0.589596	0.027569
O	-4.929661	1.479483	-0.521491
H	-5.553502	-1.444557	0.566225
H	-6.501732	-0.494276	-0.594617
C	0.852349	2.427721	-1.092599
C	-0.580744	2.817302	-0.860114
H	0.986008	2.178537	-2.149505
H	1.496691	3.285988	-0.871678
N	-0.953957	3.899404	-0.063691
H	-0.316746	4.554557	0.376167
C	-2.297668	3.957137	0.035305
H	-2.858775	4.684460	0.605100
N	-2.802363	2.954776	-0.684309
H	-3.790852	2.528383	-0.640513
C	-1.766285	2.239640	-1.249922
H	-1.926966	1.359848	-1.850961

-1514.3361134

H^+ Hairpin/-gauche

H	5.138678	3.220848	2.482798
C	4.661016	3.806933	1.691584
C	3.616793	2.947298	1.006489
N	2.362909	3.447333	0.940388
O	3.901419	1.833804	0.526505
H	4.250172	4.725858	2.119486
H	5.435343	4.067889	0.962623
H	2.118994	4.361380	1.310590
C	1.295782	2.721281	0.287148
H	1.460623	2.673249	-0.799009
H	1.251353	1.688821	0.643333
C	-0.037207	3.427810	0.544550
N	-1.120406	2.693348	0.180742
O	-0.100585	4.562505	1.019776
H	-0.987716	1.743917	-0.165481
C	-2.465963	3.229547	0.260122
H	-2.717811	3.507160	1.292209
H	-2.556222	4.142961	-0.345293
C	-3.440512	2.189542	-0.289954
N	-4.631207	2.068942	0.368484
O	-3.151198	1.469674	-1.250303
H	-4.850369	2.733836	1.100052
C	-5.702022	1.222950	-0.159571
H	-6.591532	1.355702	0.464028
H	-5.960127	1.505990	-1.184827
C	-5.380556	-0.281773	-0.216340
N	-4.381365	-0.719570	0.619074
O	-6.006462	-1.020426	-0.963202
H	-3.887305	-0.028986	1.168836
C	-3.833930	-2.058658	0.530929
H	-3.664206	-2.481080	1.525797
H	-4.568060	-2.683410	0.009405
C	-2.503073	-2.143172	-0.232304
N	-2.348510	-1.307189	-1.273969
O	-1.635738	-2.978103	0.103745
H	-2.978211	-0.512453	-1.375969
C	-1.107394	-1.262659	-2.020965
H	-0.928186	-2.220275	-2.522215
H	-1.200202	-0.485253	-2.786076
C	0.074547	-0.906047	-1.105003
N	1.230132	-1.560988	-1.378749
O	-0.050649	-0.105890	-0.170174
H	1.327500	-2.082543	-2.248515
C	2.502342	-1.279903	-0.720076
H	2.381336	-0.414517	-0.065017
C	3.543360	-1.027546	-1.834975
N	4.414451	-0.028123	-1.599787
O	3.527508	-1.770813	-2.823351
H	4.255607	0.594793	-0.803161
C	5.482284	0.306096	-2.537193
H	5.591602	-0.510788	-3.252767
H	5.250288	1.226931	-3.085863
H	6.423034	0.446772	-1.995051
C	2.981564	-2.519886	0.080079
H	3.052673	-3.362665	-0.619408
H	3.994235	-2.330882	0.451071
C	2.143332	-2.914995	1.260572
N	0.777667	-3.186823	1.238652
C	2.553101	-3.169865	2.549732
H	0.076587	-3.063435	0.479780
C	0.370495	-3.578065	2.447006
H	-0.643764	-3.843065	2.707992
H	3.532171	-3.096965	2.999033
N	1.436001	-3.580108	3.261890
H	1.417714	-3.846873	4.239595

-1761.7228876

H^+ Hairpin/trans

H	1.041432	4.765046	1.276467
C	0.808208	4.810846	0.207807
C	0.866878	3.413340	-0.369535
N	-0.228113	2.957951	-0.998289
O	1.892190	2.702163	-0.271252
H	-0.164889	5.290561	0.073260
H	1.575488	5.425742	-0.274369
H	-1.079872	3.510547	-1.063932
C	-0.307658	1.639193	-1.598423
H	0.267497	1.592836	-2.532546
H	0.107364	0.880626	-0.929032
C	-1.780121	1.341669	-1.899182
N	-2.042640	0.089507	-2.338904
O	-2.644021	2.220262	-1.761376
H	-1.331316	-0.636529	-2.224820
C	-3.412067	-0.298536	-2.674533
H	-3.872588	0.501955	-3.260631
H	-3.378984	-1.213106	-3.270786
C	-4.214659	-0.607252	-1.400586
N	-4.999354	0.394622	-0.923681
O	-4.093537	-1.694130	-0.822855
H	-4.944675	1.299814	-1.376206
C	-5.777676	0.228218	0.300413
H	-6.400701	1.116712	0.443329
H	-6.436170	-0.640506	0.216877
C	-4.952541	-0.007575	1.581775
N	-3.678693	0.510390	1.577152
O	-5.431833	-0.612332	2.529825
H	-3.372374	1.020036	0.757780
C	-2.710824	0.186809	2.608483
H	-2.197198	1.081101	2.976305
H	-3.257096	-0.256646	3.447985
C	-1.624336	-0.807633	2.167136
N	-1.919089	-1.572543	1.085323
O	-0.545639	-0.884498	2.772621
H	-2.837214	-1.533009	0.643138
C	-1.000713	-2.589293	0.598151
H	-0.655128	-3.219465	1.425913
H	-1.532094	-3.206147	-0.129291
C	0.205854	-1.961790	-0.117447
N	1.263618	-1.701277	0.683253
O	0.204198	-1.713016	-1.340671
H	1.072070	-1.730156	1.685576
C	2.492877	-1.066408	0.206479
H	2.226415	-0.385825	-0.611105
C	3.476531	-2.139528	-0.343283
N	3.087496	-2.678882	-1.524893
O	4.507177	-2.435300	0.265721
H	2.176790	-2.404462	-1.885726
C	3.807061	-3.771835	-2.170451
H	4.753364	-3.916954	-1.646521
H	3.231299	-4.703890	-2.125429
H	4.007611	-3.529792	-3.219880
C	3.128888	-0.286284	1.372601
H	3.435632	-0.997419	2.144405
H	2.372431	0.379890	1.802594
C	4.333472	0.518094	0.984172
C	5.674563	0.226305	1.071536
N	4.246766	1.798692	0.451600
H	6.166470	-0.678760	1.392485
N	6.354683	1.334947	0.596303
H	7.361972	1.429547	0.532895
C	5.470670	2.276513	0.221389
H	5.713348	3.244348	-0.193388
H	3.343690	2.285747	0.214489

-1761.7259372

Helix/-gauche

H	-5.470675	-1.423396	2.419402
C	-5.545840	-2.053289	1.528952
C	-4.265838	-1.926177	0.723412
N	-4.415737	-1.767314	-0.635214
O	-3.150707	-1.950314	1.248912
H	-5.639604	-3.091507	1.866908
H	-6.443804	-1.784422	0.963668
H	-5.345803	-1.871511	-1.019568
C	-3.281531	-1.938361	-1.542058
H	-2.791193	-2.904891	-1.383259
H	-3.652103	-1.911291	-2.572112
C	-2.165685	-0.891460	-1.418385
N	-2.491290	0.285192	-0.832709
O	-1.038783	-1.154073	-1.846759
H	-3.427350	0.379595	-0.459834
C	-1.501303	1.320876	-0.524849
H	-1.015624	1.639292	-1.454182
C	-0.342060	0.777193	0.338081
N	-0.620706	-0.251442	1.183464
O	0.782229	1.278928	0.244593
H	-1.526162	-0.715012	1.156159
C	0.432561	-0.851719	1.987292
H	0.905333	-0.101496	2.628343
H	-0.014672	-1.615351	2.631153
C	1.590959	-1.504359	1.211357
N	1.381904	-1.800046	-0.096454
O	2.654698	-1.731156	1.797777
H	0.513942	-1.531229	-0.553071
C	2.463003	-2.300970	-0.929888
H	2.939302	-3.165893	-0.459086
H	2.046056	-2.621193	-1.889286
C	3.611466	-1.314648	-1.222753
N	3.406874	-0.012855	-0.871852
O	4.640460	-1.735969	-1.748477
H	2.519029	0.273612	-0.467759
C	4.470683	0.970480	-0.936205
H	5.240509	0.602353	-1.620438
H	4.093977	1.922305	-1.323960
C	5.172992	1.289348	0.397752
N	4.867061	0.489887	1.457272
O	5.967377	2.226901	0.458019
H	4.229299	-0.297994	1.402727
H	5.356545	0.666158	2.323567
C	-2.224686	2.537274	0.117588
C	-1.238489	3.552389	0.718295
H	-2.847603	2.144221	0.938805
C	-3.155958	3.205744	-0.922263
H	-2.531974	3.725035	-1.662886
H	-0.640963	3.117963	1.523697
H	-1.783257	4.407699	1.129589
H	-0.541048	3.921300	-0.041921
H	-3.708166	2.437746	-1.480118
C	-4.169357	4.191491	-0.319447
H	-4.829526	4.591159	-1.097682
H	-3.678929	5.043834	0.162051
H	-4.799966	3.701566	0.433748

-1406.8276735

Helix/trans

C	-5.855737	-1.440525	1.466395
C	-4.566733	-1.520696	0.669198
O	-3.463884	-1.628839	1.209864
N	-4.690734	-1.447457	-0.699160
C	-3.574605	-1.805417	-1.573240
C	-2.346020	-0.889879	-1.482748
O	-1.244201	-1.314943	-1.840628
N	-2.547879	0.362706	-1.007317
C	-1.449686	1.305119	-0.787535
C	-0.400868	0.777681	0.214932
N	-0.783120	-0.195471	1.083286
C	0.191186	-0.810955	1.970483
C	1.340470	-1.561830	1.274194
N	1.156920	-1.914512	-0.023519
C	2.230596	-2.522855	-0.792164
C	3.443405	-1.621921	-1.100586
N	3.310767	-0.294125	-0.817969
C	4.436495	0.617267	-0.891870
C	5.135786	0.934212	0.444510
N	4.751080	0.201914	1.526246
C	-2.019022	2.697131	-0.397270
C	-0.956620	3.802959	-0.571856
H	-0.514181	3.704369	-1.572134
O	0.748358	1.232750	0.190705
O	2.372446	-1.809716	1.906814
O	4.457109	-2.129250	-1.577450
O	5.997154	1.811801	0.485921
C	-1.511778	5.226948	-0.414109
C	-2.646398	2.701322	1.009942
H	-2.816849	2.898041	-1.130896
H	-6.725703	-1.163326	0.862788
H	-6.039542	-2.418779	1.924264
H	-5.728094	-0.718036	2.277321
H	-5.623982	-1.484424	-1.088082
H	-3.204183	-2.812088	-1.351501
H	-3.926873	-1.793365	-2.610095
H	-3.480322	0.611374	-0.703127
H	-0.891551	1.406259	-1.724730
H	-1.717526	-0.596205	1.042590
H	0.677739	-0.058701	2.598510
H	-0.330124	-1.517036	2.624221
H	0.307247	-1.647472	-0.514855
H	2.641330	-3.385885	-0.259830
H	1.824396	-2.872445	-1.745845
H	2.434881	0.063649	-0.446478
H	5.193478	0.181497	-1.550100
H	4.126524	1.577895	-1.315302
H	4.049451	-0.531079	1.491227
H	5.231494	0.380022	2.397250
H	-0.137165	3.638440	0.136144
H	-0.734314	5.964379	-0.642719
H	-2.352496	5.408778	-1.096483
H	-1.857345	5.425870	0.606306
H	-3.196204	3.630709	1.185132
H	-3.352761	1.874317	1.160092
H	-1.875231	2.616065	1.783889

-1406.8285936

Hairpin/trans

H	5.434855	3.937467	0.938305
C	4.716646	4.205350	0.156757
C	3.686655	3.094838	0.042009
N	2.396263	3.445986	0.241157
O	4.019821	1.927779	-0.218109
H	4.277062	5.179839	0.389865
H	5.266700	4.273792	-0.787250
H	2.109197	4.403493	0.422586
C	1.319183	2.484408	0.150013
H	1.312482	1.989135	-0.828583
H	1.426338	1.689716	0.897039
C	-0.010567	3.214084	0.363527
N	-1.092605	2.401342	0.340845
O	-0.061720	4.437111	0.537273
H	-0.959842	1.396395	0.180936
C	-2.427945	2.933922	0.485238
H	-2.676225	3.124634	1.540788
H	-2.491635	3.901055	-0.031117
C	-3.447576	1.977739	-0.133712
N	-4.671364	1.950297	0.484019
O	-3.191919	1.263342	-1.102341
H	-4.847992	2.602438	1.237313
C	-5.799684	1.242672	-0.116080
H	-6.713499	1.521511	0.417514
H	-5.920337	1.525103	-1.166779
C	-5.710556	-0.295402	-0.115300
N	-4.768393	-0.844353	0.706843
O	-6.475773	-0.943637	-0.820678
H	-4.183407	-0.214625	1.239728
C	-4.446318	-2.260425	0.702794
H	-4.450569	-2.670257	1.717811
H	-5.222008	-2.772853	0.125026
C	-3.080015	-2.622705	0.092953
N	-2.545134	-1.721569	-0.779809
O	-2.548621	-3.696105	0.375269
H	-2.977976	-0.812897	-0.918692
C	-1.249518	-1.954887	-1.377501
H	-1.108304	-3.032871	-1.497482
H	-1.222888	-1.484555	-2.368205
C	-0.091072	-1.348908	-0.563972
N	1.072762	-2.025027	-0.638661
O	-0.228052	-0.290197	0.070490
H	1.135962	-2.864351	-1.207410
C	2.356037	-1.550106	-0.115269
H	2.235345	-0.496622	0.151245
C	3.361346	-1.721974	-1.271434
N	4.152698	-0.655121	-1.529480
O	3.390844	-2.791582	-1.894100
H	4.041490	0.200996	-0.985210
C	5.172205	-0.677198	-2.567703
H	5.188387	-1.673474	-3.014110
H	4.951471	0.061208	-3.348689
H	6.159055	-0.452547	-2.145449
C	2.803056	-2.355420	1.139042
H	2.884724	-3.406025	0.823009
C	4.197206	-1.892756	1.614099
C	1.747147	-2.258080	2.252383
H	1.999002	-2.919452	3.087413
H	0.756450	-2.550271	1.891831
H	1.676902	-1.233722	2.640666
H	4.154137	-0.824884	1.875584
H	4.907252	-1.976147	0.782998
C	4.753643	-2.689821	2.804681
H	5.780326	-2.378053	3.029995
H	4.773325	-3.764711	2.584587
H	4.158761	-2.545013	3.712746

-1654.2090636

K Helix/-gauche

H	4.364964	-3.686474	-2.604779
C	4.562969	-4.012411	-1.580238
C	3.546665	-3.380609	-0.650735
N	4.029976	-2.862718	0.530937
O	2.346958	-3.313319	-0.929897
H	4.426476	-5.099536	-1.550615
H	5.598734	-3.782295	-1.311889
H	4.990953	-3.076415	0.767854
C	3.119626	-2.582113	1.641726
H	2.525468	-3.464209	1.903772
H	3.716818	-2.306344	2.517621
C	2.100880	-1.460980	1.408387
N	2.409256	-0.509487	0.471418
O	1.070002	-1.423980	2.076802
H	3.179639	-0.702454	-0.157295
C	1.406557	0.482447	0.103221
H	0.936233	0.808445	1.037882
C	0.193334	-0.049947	-0.698648
N	0.177425	-1.348143	-1.074686
O	-0.742242	0.731645	-0.906148
H	0.967912	-1.967780	-0.899718
C	-1.030745	-1.916610	-1.660361
H	-1.332506	-1.359861	-2.551955
H	-0.822225	-2.950558	-1.951459
C	-2.260001	-1.905167	-0.732158
N	-2.023144	-1.824246	0.605480
O	-3.392265	-1.954422	-1.215655
H	-1.070478	-1.772683	0.949257
C	-3.119865	-1.729652	1.558996
H	-3.859278	-2.512466	1.365990
H	-2.725273	-1.877668	2.568332
C	-3.911938	-0.406766	1.553321
N	-3.391344	0.615543	0.811588
O	-4.945816	-0.327176	2.210185
H	-2.544717	0.465821	0.272547
C	-4.133068	1.836621	0.561281
H	-4.932668	1.907986	1.304548
H	-3.488430	2.714958	0.670350
C	-4.792429	1.952538	-0.826527
N	-4.827292	0.823237	-1.587484
O	-5.246413	3.036062	-1.190071
H	-4.482918	-0.079393	-1.276018
H	-5.316375	0.879213	-2.470506
C	2.003011	1.713240	-0.599585
H	1.180690	2.200116	-1.133639
H	2.739669	1.397330	-1.353227
C	2.636586	2.715985	0.378815
H	3.424356	2.224997	0.966387
H	1.869224	3.051836	1.088399
C	3.224199	3.936292	-0.357481
H	2.427219	4.433209	-0.928762
H	3.984860	3.599724	-1.077169
C	3.849271	4.922274	0.626465
H	4.679956	4.483163	1.186106
H	3.117683	5.324014	1.333241
N	4.431763	6.133642	-0.112985
H	3.697227	6.621764	-0.641024
H	5.149323	5.839664	-0.786942
H	4.858888	6.809765	0.532216

-1462.5486921

K Helix/trans

H	-5.922805	-0.010033	2.553027
C	-6.261296	-0.472843	1.622343
C	-5.064607	-0.731336	0.729481
N	-5.215688	-0.426258	-0.604305
O	-3.999530	-1.179685	1.163511
H	-6.712632	-1.437693	1.880782
H	-7.024481	0.155494	1.152868
H	-6.147373	-0.191538	-0.923908
C	-4.284883	-0.961908	-1.597541
H	-4.230903	-2.054772	-1.547106
H	-4.644255	-0.686281	-2.594858
C	-2.837797	-0.475394	-1.474121
N	-2.630804	0.731269	-0.867553
O	-1.917339	-1.152789	-1.930860
H	-3.415359	1.184143	-0.415424
C	-1.270880	1.199761	-0.635218
H	-0.720398	1.073347	-1.574606
C	-0.471281	0.342812	0.372929
N	-1.120933	-0.600449	1.088899
O	0.750834	0.533197	0.473676
H	-2.124405	-0.756809	0.989024
C	-0.356292	-1.514674	1.927931
H	0.244092	-0.965848	2.658526
H	-1.058707	-2.155613	2.469504
C	0.642152	-2.414492	1.174392
N	0.421368	-2.628127	-0.151403
O	1.600420	-2.895041	1.783705
H	-0.364547	-2.185978	-0.618902
C	1.371023	-3.396765	-0.946987
H	1.599501	-4.344802	-0.451663
H	0.918467	-3.621429	-1.917141
C	2.737515	-2.729549	-1.209437
N	2.843375	-1.415867	-0.849643
O	3.641754	-3.381709	-1.721152
H	2.050679	-0.961851	-0.411838
C	4.101538	-0.699263	-0.846948
H	4.810051	-1.251517	-1.472436
H	3.983831	0.301551	-1.275808
C	4.767616	-0.508999	0.529202
N	4.252907	-1.225476	1.565485
O	5.703222	0.283450	0.643371
H	3.503660	-1.903192	1.463060
H	4.736052	-1.166253	2.451654
C	-1.244052	2.695670	-0.244664
H	-1.938313	3.231454	-0.906535
H	-1.608261	2.821991	0.784709
C	0.168960	3.291230	-0.386403
H	0.495953	3.166770	-1.428145
H	0.861610	2.707699	0.225889
C	0.259294	4.781635	-0.010768
H	-0.049196	4.924284	1.034971
H	-0.411987	5.381242	-0.643198
C	1.705170	5.242616	-0.195121
H	2.039758	5.143284	-1.231505
H	2.396036	4.694307	0.451393
N	1.876508	6.724171	0.161001
H	1.612874	6.895073	1.139077
H	1.277244	7.314213	-0.428575
H	2.848720	7.033520	0.037355

-1462.5564246

K Hairpin/-gauche

H	-5.530946	-2.939872	2.713362
C	-5.064362	-3.538344	1.924811
C	-3.972317	-2.716110	1.267256
N	-2.738353	-3.267650	1.223845
O	-4.200483	-1.590280	0.791039
H	-4.700045	-4.477733	2.350688
H	-5.835438	-3.759156	1.179316
H	-2.542476	-4.199714	1.577386
C	-1.630018	-2.589985	0.588032
H	-1.820202	-2.446172	-0.484551
H	-1.477330	-1.592215	1.010932
C	-0.362083	-3.430584	0.758580
N	0.769740	-2.809790	0.339652
O	-0.388346	-4.571246	1.226292
H	0.715308	-1.854855	-0.019695
C	2.048689	-3.494322	0.358621
H	2.323531	-3.781825	1.382317
H	1.998913	-4.423056	-0.227718
C	3.105903	-2.588025	-0.268138
N	4.317093	-2.551564	0.365415
O	2.870089	-1.898897	-1.263652
H	4.479143	-3.187320	1.136231
C	5.458942	-1.852343	-0.222268
H	6.351935	-2.082457	0.366635
H	5.636397	-2.186762	-1.249401
C	5.333440	-0.319719	-0.305492
N	4.352507	0.252290	0.464269
O	6.092369	0.318520	-1.022569
H	3.749496	-0.363323	0.993566
C	3.977646	1.646277	0.331521
H	3.869281	2.119052	1.313089
H	4.785717	2.152716	-0.207937
C	2.662250	1.894356	-0.430711
N	2.352154	0.987022	-1.385886
O	1.978512	2.900496	-0.179498
H	2.858673	0.104241	-1.408486
C	1.100581	1.026375	-2.112845
H	0.959467	2.012541	-2.567395
H	1.149547	0.282151	-2.914735
C	-0.098409	0.695588	-1.198587
N	-1.282055	1.216401	-1.586766
O	0.045159	0.009628	-0.174747
H	-1.356739	1.761602	-2.441610
C	-2.481614	1.247350	-0.745901
H	-2.443396	0.405091	-0.051979
C	-3.692499	1.163220	-1.694262
N	-4.686228	0.340683	-1.298174
O	-3.696448	1.865088	-2.713269
H	-4.526130	-0.285423	-0.506582
C	-5.910745	0.176745	-2.071950
H	-5.964798	0.974961	-2.814607
H	-5.923519	-0.789158	-2.592024
H	-6.782794	0.231272	-1.411446
C	-2.545875	2.591509	0.030542
H	-3.386504	2.541977	0.735050
H	-2.765692	3.392492	-0.687087
C	-1.231616	2.879191	0.766064
H	-0.402659	2.911628	0.055399
H	-1.015121	2.048059	1.450377
C	-1.213567	4.194994	1.560833
H	-1.386779	5.044823	0.884834
H	-2.008704	4.202875	2.321235
C	0.157271	4.318294	2.222289
H	0.970095	4.329240	1.488462
H	0.339182	3.506883	2.932734
N	0.258118	5.614016	3.040545
H	-0.471097	5.645526	3.762413
H	0.124927	6.430522	2.431946
H	1.171410	5.708260	3.499823

-1709.9502022

K Hairpin/trans

H	4.734140	4.239248	1.077193
C	3.932809	4.549491	0.399373
C	2.980935	3.385728	0.192937
N	1.675396	3.621677	0.434110
O	3.390438	2.269595	-0.184784
H	3.442969	5.440571	0.802287
H	4.393276	4.801170	-0.561858
H	1.326490	4.529334	0.732604
C	0.661627	2.609684	0.229518
H	0.631804	2.289378	-0.819281
H	0.866255	1.712084	0.822205
C	-0.702729	3.189187	0.621671
N	-1.723160	2.306610	0.516472
O	-0.825922	4.360039	0.993153
H	-1.529245	1.359445	0.178281
C	-3.092050	2.701859	0.786753
H	-3.255232	2.859820	1.862284
H	-3.312220	3.656160	0.289375
C	-4.040260	1.635003	0.238185
N	-5.126263	1.346897	1.017523
O	-3.820251	1.048129	-0.822440
H	-5.289881	1.904545	1.846026
C	-6.191537	0.467870	0.536177
H	-7.032712	0.522355	1.233677
H	-6.545646	0.790545	-0.447892
C	-5.815089	-1.017389	0.367811
N	-4.664581	-1.425394	0.986272
O	-6.542843	-1.754902	-0.284772
H	-4.123292	-0.729127	1.480664
C	-4.071743	-2.732169	0.759117
H	-3.785548	-3.205947	1.703083
H	-4.830050	-3.357107	0.275834
C	-2.815753	-2.720473	-0.130961
N	-2.756343	-1.735266	-1.072143
O	-1.948459	-3.586740	-0.008932
H	-3.394014	-0.945272	-1.028389
C	-1.585470	-1.602718	-1.904928
H	-1.318726	-2.576754	-2.328250
H	-1.818296	-0.919128	-2.729430
C	-0.382227	-1.019862	-1.144333
N	0.832455	-1.373118	-1.622779
O	-0.519823	-0.238457	-0.189884
H	0.924624	-2.065102	-2.360416
C	2.082361	-0.914583	-1.027596
H	1.928059	0.088358	-0.624269
C	3.143368	-0.904477	-2.142295
N	3.943937	0.188162	-2.164628
O	3.228409	-1.868070	-2.911441
H	3.751844	0.951332	-1.513230
C	4.987451	0.365872	-3.166875
H	5.111408	-0.573735	-3.708822
H	4.715883	1.151218	-3.882836
H	5.934869	0.641721	-2.689902
C	2.499190	-1.869611	0.114677
H	2.622090	-2.874463	-0.309075
H	1.668697	-1.910602	0.829994
C	3.788519	-1.442285	0.831517
H	3.667989	-0.431492	1.244061
H	4.616458	-1.394505	0.113394
C	4.148377	-2.432390	1.955603
H	3.320006	-2.483596	2.677339
H	4.275135	-3.436857	1.526738
C	5.428492	-2.009802	2.668722
H	5.334164	-1.033374	3.152082
H	6.293210	-1.994447	1.999388
N	5.775258	-3.004737	3.785938
H	5.904748	-3.947602	3.398660
H	5.010070	-3.057733	4.469274
H	6.633781	-2.744611	4.286193

-1709.9426748

L Helix/-gauche

H	5.329477	-1.865732	-2.449489
C	5.464931	-2.365199	-1.486097
C	4.241149	-2.115623	-0.624083
N	4.477800	-1.704085	0.666950
O	3.093542	-2.255848	-1.052817
H	5.536112	-3.440943	-1.681211
H	6.398553	-2.026245	-1.026266
H	5.433012	-1.724452	0.999631
C	3.407790	-1.715660	1.663199
H	2.942512	-2.704716	1.733430
H	3.838891	-1.471911	2.639959
C	2.252971	-0.739253	1.402366
N	2.516657	0.329654	0.610221
O	1.150660	-0.950290	1.914459
H	3.434392	0.397577	0.189172
C	1.473711	1.271297	0.207455
H	0.979338	1.648930	1.107727
C	0.327132	0.614285	-0.588910
N	0.570274	-0.582483	-1.184378
O	-0.765975	1.186346	-0.651192
H	1.464252	-1.054477	-1.067053
C	-0.504705	-1.302270	-1.847608
H	-0.965158	-0.684071	-2.624031
H	-0.087227	-2.194897	-2.323488
C	-1.664966	-1.742201	-0.936983
N	-1.437342	-1.750804	0.401426
O	-2.746669	-2.057918	-1.443762
H	-0.540907	-1.447376	0.773384
C	-2.510836	-2.033793	1.340289
H	-3.014213	-2.968673	1.076553
H	-2.081526	-2.147990	2.339994
C	-3.633910	-0.980642	1.428084
N	-3.413643	0.209133	0.798109
O	-4.659315	-1.253812	2.049469
H	-2.532087	0.378510	0.321579
C	-4.462841	1.200204	0.653549
H	-5.213046	1.028432	1.430569
H	-4.058481	2.208857	0.784930
C	-5.209612	1.200642	-0.694989
N	-4.927875	0.180690	-1.552075
O	-6.013712	2.098256	-0.942698
H	-4.281445	-0.572716	-1.339521
H	-5.444630	0.153028	-2.420065
C	2.072900	2.441884	-0.591659
H	1.232440	3.044671	-0.954541
H	2.577304	2.037716	-1.482523
C	3.049570	3.349426	0.187158
H	3.863037	2.727180	0.592033
C	3.679723	4.373176	-0.773238
H	4.401526	5.011216	-0.250074
H	2.911560	5.026485	-1.206367
H	4.204246	3.881763	-1.602252
C	2.369165	4.060370	1.369684
H	1.988017	3.355325	2.116780
H	1.525138	4.670524	1.023932
H	3.076525	4.725506	1.878468

-1406.8310419

L Helix/trans

C	-5.919607	-1.473863	1.498248
C	-4.635097	-1.532569	0.692027
N	-4.767486	-1.428897	-0.673442
C	-3.656027	-1.762342	-1.562872
C	-2.429322	-0.845740	-1.458783
N	-2.630681	0.393929	-0.949287
C	-1.531852	1.327691	-0.694693
C	-2.122839	2.644571	-0.147573
H	-2.632413	2.426304	0.802856
O	-3.528837	-1.650370	1.223753
O	-1.328773	-1.257738	-1.834390
C	-0.457987	0.742674	0.244593
O	0.703024	1.162155	0.182638
N	-0.846292	-0.220989	1.121428
C	0.129113	-0.864907	1.987208
C	1.250204	-1.641404	1.273700
O	2.277789	-1.926040	1.898118
N	1.050219	-1.974889	-0.026411
C	2.104993	-2.600347	-0.807766
C	3.341468	-1.727987	-1.104716
O	4.343249	-2.256480	-1.583717
N	3.241357	-0.399866	-0.810524
C	4.389416	0.483947	-0.875224
C	5.093730	0.773245	0.464829
O	5.974006	1.631353	0.515472
N	4.691448	0.039669	1.539374
C	-1.127527	3.803624	0.069968
H	-2.894865	2.978221	-0.857552
H	-6.795015	-1.191857	0.904846
H	-6.094452	-2.460831	1.940616
H	-5.791422	-0.764280	2.320452
H	-5.702928	-1.457102	-1.057737
H	-3.281279	-2.772933	-1.367732
H	-4.014938	-1.726642	-2.596900
H	-3.559881	0.630337	-0.624734
H	-1.003762	1.513284	-1.634461
H	-1.786141	-0.610016	1.085076
H	0.643007	-0.126458	2.609958
H	-0.397416	-1.560555	2.647865
H	0.209739	-1.672261	-0.512461
H	2.492815	-3.482853	-0.290522
H	1.686833	-2.922970	-1.765790
H	2.372633	-0.023319	-0.440429
H	5.136801	0.034611	-1.535346
H	4.104386	1.455164	-1.291958
H	3.976383	-0.679818	1.495829
H	5.176593	0.197054	2.411761
C	-1.861590	4.986205	0.725291
H	-0.345150	3.460942	0.757327
C	-0.443112	4.239177	-1.237091
H	-1.167405	5.810306	0.924913
H	-2.655546	5.372449	0.071753
H	-2.321231	4.698701	1.679332
H	0.200313	5.108400	-1.059255
H	0.190354	3.445760	-1.645573
H	-1.183413	4.522791	-1.997953

-1406.8304702

L Hairpin/-gauche

H	5.727203	3.572784	1.281583
C	5.103655	3.887065	0.438423
C	4.017658	2.847181	0.222248
N	2.740452	3.283686	0.305826
O	4.295226	1.661472	-0.016409
H	4.709771	4.888745	0.635127
H	5.742053	3.918412	-0.450298
H	2.502289	4.254009	0.489654
C	1.613580	2.394701	0.124617
H	1.613774	1.952598	-0.879866
H	1.649602	1.559306	0.832239
C	0.321545	3.191716	0.328255
N	-0.804843	2.443371	0.267483
O	0.334855	4.412192	0.525067
H	-0.730567	1.432833	0.105244
C	-2.108176	3.053109	0.403788
H	-2.352983	3.252168	1.458534
H	-2.109716	4.025475	-0.106584
C	-3.178456	2.164658	-0.229913
N	-4.403783	2.201145	0.383944
O	-2.961358	1.449115	-1.207176
H	-4.543253	2.850001	1.147767
C	-5.570405	1.568195	-0.226019
H	-6.467918	1.898326	0.305892
H	-5.668722	1.866225	-1.274741
C	-5.576418	0.027514	-0.238126
N	-4.665356	-0.585620	0.573657
O	-6.383566	-0.565853	-0.945069
H	-4.037794	0.002446	1.105708
C	-4.424818	-2.017738	0.548695
H	-4.448888	-2.440951	1.557923
H	-5.230428	-2.476826	-0.032966
C	-3.082269	-2.446027	-0.071078
N	-2.505596	-1.565861	-0.938727
O	-2.605649	-3.547886	0.198780
H	-2.886550	-0.631789	-1.059651
C	-1.218269	-1.852703	-1.529632
H	-1.134531	-2.932104	-1.685552
H	-1.152151	-1.351015	-2.502810
C	-0.038762	-1.344372	-0.679863
N	1.092008	-2.070005	-0.783109
O	-0.132043	-0.315257	0.010751
H	1.130292	-2.862533	-1.418174
C	2.387938	-1.681761	-0.227299
H	2.312795	-0.643628	0.101434
C	3.418662	-1.831301	-1.362517
N	4.386148	-0.885515	-1.396079
O	3.339332	-2.794096	-2.136131
H	4.304874	-0.060553	-0.800863
C	5.465572	-0.916858	-2.371605
H	5.440836	-1.880719	-2.883895
H	5.351692	-0.117986	-3.115761
H	6.433626	-0.793187	-1.872486
C	2.811063	-2.592581	0.949681
H	3.819290	-2.287250	1.263811
H	2.898642	-3.615745	0.560599
C	1.872137	-2.586351	2.172139
H	0.859119	-2.828861	1.822613
C	2.304260	-3.683283	3.160606
C	1.821105	-1.214791	2.867793
H	1.627326	-3.725629	4.022366
H	3.317039	-3.494650	3.541555
H	2.304223	-4.672211	2.686403
H	1.194392	-1.261085	3.766753
H	1.394954	-0.449061	2.211670
H	2.824517	-0.891672	3.176156

-1654.2109685

L Hairpin/trans

H	5.437090	3.972870	0.380832
C	4.661593	4.145686	-0.372322
C	3.641808	3.023644	-0.286407
N	2.364343	3.386448	-0.034577
O	3.971519	1.836102	-0.437186
H	4.225938	5.137719	-0.220052
H	5.142077	4.109508	-1.355289
H	2.079800	4.355917	0.072285
C	1.296368	2.414831	0.060275
H	1.209328	1.831601	-0.864457
H	1.481665	1.695679	0.866398
C	-0.019815	3.152975	0.323539
N	-1.086899	2.331395	0.468288
O	-0.070208	4.385767	0.394442
H	-0.953777	1.320291	0.351553
C	-2.417441	2.856341	0.664579
H	-2.655610	2.969316	1.733878
H	-2.469416	3.858669	0.220405
C	-3.457133	1.958350	-0.009349
N	-4.679812	1.919578	0.611023
O	-3.217949	1.296050	-1.018025
H	-4.841757	2.533395	1.399072
C	-5.824248	1.269935	-0.022944
H	-6.731350	1.542192	0.525333
H	-5.938520	1.608271	-1.057706
C	-5.770560	-0.267859	-0.100086
N	-4.839037	-0.878262	0.690414
O	-6.551924	-0.862289	-0.834561
H	-4.237949	-0.288539	1.250428
C	-4.546342	-2.298482	0.611062
H	-4.566293	-2.763610	1.601864
H	-5.328460	-2.762261	0.001849
C	-3.183447	-2.655151	-0.009461
N	-2.613771	-1.707582	-0.808134
O	-2.684920	-3.759808	0.201885
H	-3.032317	-0.786566	-0.900974
C	-1.319098	-1.927714	-1.409250
H	-1.185925	-3.002001	-1.567551
H	-1.284095	-1.424514	-2.383561
C	-0.154970	-1.366016	-0.571457
N	1.029863	-1.973954	-0.772622
O	-0.297922	-0.385963	0.178956
H	1.095604	-2.805148	-1.353062
C	2.292712	-1.551167	-0.162850
H	2.229216	-0.478967	0.031494
C	3.388619	-1.865923	-1.196920
N	4.304429	-0.889035	-1.396271
O	3.390658	-2.964713	-1.766311
H	4.159543	0.027636	-0.973362
C	5.412812	-1.046066	-2.326549
H	5.479917	-2.097913	-2.611581
H	5.260495	-0.444769	-3.232289
H	6.351158	-0.734109	-1.854129
C	2.536736	-2.326712	1.153754
H	1.661207	-2.165280	1.797249
H	2.561862	-3.394854	0.902444
C	3.814915	-1.955565	1.931418
H	4.673072	-2.050095	1.251020
C	4.023127	-2.951340	3.086124
C	3.782750	-0.510546	2.461884
H	4.941151	-2.724573	3.641777
H	4.098431	-3.981292	2.717144
H	3.186031	-2.911119	3.795880
H	4.682463	-0.295600	3.051461
H	2.912958	-0.357945	3.115385
H	3.738302	0.229467	1.655581

-1654.2110651

M **Helix/-gauche**

H	4.690731	-2.867266	-2.449480
C	4.720091	-3.420698	-1.506743
C	3.545896	-2.992874	-0.646512
N	3.829406	-2.680454	0.662985
O	2.398449	-2.910419	-1.091017
H	4.603172	-4.483498	-1.745921
H	5.691257	-3.270804	-1.024827
H	4.761324	-2.879139	1.003093
C	2.758533	-2.544163	1.648613
H	2.123798	-3.436497	1.670612
H	3.210130	-2.423386	2.638982
C	1.801017	-1.366457	1.423565
N	2.265359	-0.329694	0.680303
O	0.672104	-1.393923	1.918914
H	3.175618	-0.424224	0.247544
C	1.408202	0.792319	0.302380
H	0.974951	1.223514	1.211760
C	0.176471	0.378910	-0.532368
N	0.217399	-0.815138	-1.177674
O	-0.796366	1.137904	-0.578984
H	1.011843	-1.442692	-1.072526
C	-0.954535	-1.300091	-1.889070
H	-1.283730	-0.571603	-2.635865
H	-0.691198	-2.227447	-2.406838
C	-2.191062	-1.572699	-1.013556
N	-1.989801	-1.695662	0.324067
O	-3.302621	-1.661377	-1.544343
H	-1.060336	-1.572695	0.716849
C	-3.109699	-1.836503	1.240482
H	-3.769999	-2.646173	0.915894
H	-2.722418	-2.089793	2.231721
C	-4.023506	-0.604269	1.395845
N	-3.588965	0.559158	0.831673
O	-5.082877	-0.721412	2.009021
H	-2.691991	0.590748	0.355494
C	-4.437051	1.733690	0.757891
H	-5.209533	1.652142	1.527683
H	-3.855197	2.640607	0.950344
C	-5.164386	1.956824	-0.582366
N	-5.078777	0.954168	-1.500144
O	-5.782963	3.003288	-0.770427
H	-4.580727	0.084607	-1.339546
H	-5.582789	1.078455	-2.367137
C	2.220761	1.867591	-0.440069
H	1.514769	2.583149	-0.871750
H	2.758123	1.401914	-1.278970
C	3.205254	2.608748	0.477285
H	3.823898	1.905255	1.048246
H	2.651055	3.229076	1.191560
S	4.298101	3.680174	-0.536463
C	5.214624	4.525666	0.799515
H	5.922491	5.206301	0.317672
H	5.773953	3.811520	1.412997
H	4.539374	5.110033	1.432702

-1765.6937809

M **Helix/trans**

H	-5.655925	-1.229369	2.306408
C	-5.744640	-2.003478	1.539051
C	-4.455661	-2.057047	0.740377
N	-4.586477	-2.063121	-0.629312
O	-3.346579	-2.077688	1.279218
H	-5.872131	-2.962535	2.053191
H	-6.630013	-1.810203	0.925333
H	-5.517003	-2.168346	-1.012306
C	-3.453398	-2.403096	-1.488013
H	-3.027352	-3.375166	-1.217281
H	-3.805866	-2.462051	-2.523203
C	-2.278673	-1.416661	-1.445898
N	-2.555505	-0.151609	-1.041685
O	-1.151618	-1.793401	-1.775379
H	-3.500632	0.058508	-0.746098
C	-1.511411	0.854953	-0.854306
H	-0.973477	0.992265	-1.798712
C	-0.422938	0.417470	0.147745
N	-0.760137	-0.506364	1.084162
O	0.705126	0.917751	0.074526
H	-1.672750	-0.957331	1.069013
C	0.242603	-1.013334	2.008508
H	0.686321	-0.197111	2.586312
H	-0.244309	-1.701030	2.706809
C	1.431567	-1.747976	1.363048
N	1.268056	-2.204285	0.094779
O	2.474795	-1.893492	2.008298
H	0.409475	-2.011216	-0.414829
C	2.374976	-2.807542	-0.630210
H	2.832044	-3.603915	-0.035554
H	1.989893	-3.249871	-1.553688
C	3.536249	-1.866389	-1.009610
N	3.327860	-0.530677	-0.828887
O	4.577452	-2.351151	-1.449011
H	2.432672	-0.196665	-0.482079
C	4.396571	0.437936	-0.979372
H	5.192807	-0.016462	-1.576051
H	4.040102	1.328812	-1.506247
C	5.041924	0.933686	0.329210
N	4.706467	0.265635	1.467638
O	5.821453	1.885114	0.300904
H	4.072047	-0.526366	1.489397
H	5.155598	0.558789	2.324073
C	-2.173666	2.185689	-0.436800
H	-2.978638	2.397915	-1.154083
H	-2.638297	2.068232	0.551877
C	-1.206154	3.375763	-0.413340
H	-0.749251	3.510761	-1.400634
H	-0.403666	3.211344	0.310349
S	-2.139588	4.892429	0.039014
C	-0.757282	6.087481	0.075335
H	-1.182391	7.058759	0.344403
H	-0.280374	6.169260	-0.906499
H	-0.012749	5.805267	0.826220

-1765.6941755

M **Hairpin/-gauche**

H	5.607120	3.602600	1.487921
C	4.907775	4.046313	0.772041
C	3.839652	3.020329	0.436374
N	2.560215	3.374325	0.695326
O	4.131094	1.915070	-0.047080
H	4.501326	4.971448	1.191654
H	5.470197	4.279270	-0.137820
H	2.306203	4.289418	1.056082
C	1.450580	2.492754	0.406010
H	1.449341	2.194244	-0.649466
H	1.510700	1.567239	0.990029
C	0.142252	3.219340	0.732414
N	-0.966932	2.469619	0.532140
O	0.128572	4.386310	1.139489
H	-0.868693	1.508493	0.187712
C	-2.285971	3.016634	0.757842
H	-2.526475	3.059839	1.831048
H	-2.324162	4.049814	0.386966
C	-3.331115	2.188635	0.010281
N	-4.547987	2.085057	0.632763
O	-3.098785	1.632370	-1.062420
H	-4.702596	2.610566	1.483507
C	-5.695987	1.495555	-0.051544
H	-6.597401	1.700778	0.533882
H	-5.827111	1.940440	-1.043155
C	-5.633940	-0.025020	-0.291580
N	-4.681152	-0.708692	0.408295
O	-6.428017	-0.544622	-1.067947
H	-4.070978	-0.178802	1.016124
C	-4.387450	-2.111840	0.176386
H	-4.376059	-2.674251	1.115381
H	-5.186815	-2.515136	-0.453349
C	-3.043198	-2.395557	-0.518256
N	-2.526707	-1.386875	-1.277783
O	-2.514854	-3.500818	-0.402923
H	-2.942312	-0.459999	-1.256223
C	-1.247150	-1.540610	-1.931128
H	-1.116631	-2.591640	-2.204612
H	-1.235893	-0.936655	-2.846889
C	-0.065744	-1.069989	-1.062826
N	1.112747	-1.669255	-1.329948
O	-0.197656	-0.175354	-0.211474
H	1.187833	-2.352162	-2.079979
C	2.395413	-1.252412	-0.769100
H	2.283143	-0.240270	-0.374043
C	3.416321	-1.283098	-1.923340
N	4.393799	-0.351186	-1.845847
O	3.319983	-2.144967	-2.805725
H	4.318456	0.388606	-1.147042
C	5.473547	-0.276267	-2.818534
H	5.422975	-1.158775	-3.459388
H	5.381853	0.621104	-3.443782
H	6.444630	-0.250885	-2.310493
C	2.882956	-2.194404	0.358882
H	3.866763	-1.840093	0.691834
H	3.022022	-3.200988	-0.054000
C	1.919806	-2.249381	1.550473
H	0.951193	-2.654311	1.239175
H	1.756482	-1.245978	1.960963
S	2.620515	-3.337101	2.855317
C	1.183401	-3.413692	3.981416
H	0.314411	-3.851406	3.479278
H	0.926983	-2.421690	4.368349
H	1.467783	-4.055808	4.820408

-2013.0747057

M **Hairpin/trans**

H	5.225394	3.994269	0.133855
C	4.399434	4.222794	-0.547348
C	3.386103	3.093621	-0.481256
N	2.127822	3.431898	-0.122305
O	3.703655	1.921494	-0.742539
H	3.977023	5.197816	-0.286710
H	4.810367	4.267383	-1.561089
H	1.850231	4.390366	0.069513
C	1.065869	2.452936	-0.038681
H	0.936468	1.929720	-0.993738
H	1.287788	1.685057	0.711492
C	-0.238656	3.166751	0.329733
N	-1.296587	2.332558	0.462420
O	-0.287810	4.391820	0.484802
H	-1.167974	1.332449	0.274456
C	-2.620649	2.834712	0.745654
H	-2.793969	2.935175	1.828420
H	-2.721655	3.839374	0.314802
C	-3.679895	1.919612	0.127794
N	-4.865969	1.857886	0.813504
O	-3.483854	1.264090	-0.894323
H	-4.995500	2.466690	1.611430
C	-6.033195	1.196685	0.235453
H	-6.912258	1.446866	0.837337
H	-6.211009	1.545784	-0.786732
C	-5.961249	-0.338697	0.135707
N	-4.981067	-0.946441	0.867122
O	-6.773598	-0.934993	-0.562836
H	-4.360687	-0.358085	1.407199
C	-4.691509	-2.365889	0.768762
H	-4.643674	-2.829572	1.759300
H	-5.512489	-2.832074	0.215182
C	-3.376261	-2.728025	0.055960
N	-2.842719	-1.773060	-0.759836
O	-2.884545	-3.844080	0.215967
H	-3.256360	-0.846557	-0.812170
C	-1.597093	-2.002918	-1.453239
H	-1.445177	-3.083269	-1.534946
H	-1.656220	-1.583010	-2.465802
C	-0.387452	-1.346770	-0.764065
N	0.807647	-1.883346	-1.083594
O	-0.506885	-0.359791	-0.020362
H	0.876940	-2.709291	-1.672168
C	2.088307	-1.336409	-0.642630
H	1.981647	-0.255445	-0.527729
C	3.105656	-1.663557	-1.750620
N	4.022861	-0.696997	-1.990957
O	3.051388	-2.752685	-2.334811
H	3.930623	0.200972	-1.515546
C	5.078884	-0.856625	-2.979748
H	5.061695	-1.887335	-3.339529
H	4.927520	-0.181434	-3.831580
H	6.057698	-0.642956	-2.534518
C	2.506057	-1.951390	0.716521
H	2.596487	-3.037901	0.602395
H	1.687801	-1.759631	1.421214
C	3.812999	-1.370459	1.271703
H	3.776247	-0.274175	1.277969
H	4.666041	-1.679200	0.658018
S	4.067643	-1.977347	2.987087
C	5.716205	-1.260801	3.315164
H	5.695851	-0.168094	3.244470
H	6.466158	-1.661185	2.624921
H	5.990659	-1.542912	4.336036

-2013.0748083

N$_{\mathbf{Helix/-gauche}}$

H	5.319114	-1.283336	-2.396625
C	5.465457	-1.948821	-1.540553
C	4.241122	-1.871251	-0.647089
N	4.466859	-1.554387	0.666845
O	3.100224	-2.067399	-1.077680
H	5.548202	-2.969864	-1.927379
H	6.396341	-1.681330	-1.031219
H	5.421103	-1.458013	0.985743
C	3.396781	-1.615105	1.655401
H	2.970482	-2.621906	1.719313
H	3.811032	-1.356468	2.635861
C	2.209539	-0.685720	1.370893
N	2.473795	0.424638	0.642520
O	1.083204	-0.979358	1.785033
H	3.434973	0.710759	0.460315
C	1.425432	1.365811	0.280776
H	0.946460	1.769854	1.181117
C	0.270476	0.725574	-0.518695
N	0.542078	-0.417369	-1.195709
O	-0.831544	1.284379	-0.533505
H	1.445754	-0.878331	-1.093242
C	-0.515580	-1.119119	-1.906167
H	-0.975304	-0.471249	-2.658456
H	-0.076992	-1.980854	-2.418579
C	-1.680381	-1.620581	-1.033606
N	-1.454511	-1.734697	0.299500
O	-2.761971	-1.891921	-1.566221
H	-0.557296	-1.464338	0.697704
C	-2.528376	-2.099436	1.209365
H	-3.029378	-3.009549	0.866227
H	-2.099756	-2.297723	2.196115
C	-3.653108	-1.059892	1.386577
N	-3.435728	0.176678	0.852941
O	-4.676078	-1.382798	1.987845
H	-2.556984	0.381537	0.385329
C	-4.483836	1.176847	0.792989
H	-5.245540	0.923775	1.536068
H	-4.086651	2.168029	1.034152
C	-5.206833	1.313852	-0.561112
N	-4.938069	0.362640	-1.497613
O	-5.985331	2.249443	-0.742186
H	-4.304215	-0.416054	-1.346133
H	-5.438439	0.424304	-2.373413
C	2.004851	2.523097	-0.566048
H	1.195049	3.229771	-0.766589
H	2.355218	2.125702	-1.526015
C	3.210623	3.189499	0.093419
O	4.255572	2.563152	0.302086
N	3.068135	4.501833	0.428101
H	2.211890	5.009171	0.260082
H	3.847417	4.978256	0.863662

-1457.6155548

N$_{\mathbf{Helix/trans}}$

H	-5.741976	-0.846344	2.406745
C	-5.900721	-1.483441	1.532276
C	-4.643452	-1.479965	0.684071
N	-4.816191	-1.271723	-0.664430
O	-3.520110	-1.638592	1.169469
H	-6.069258	-2.503500	1.895014
H	-6.791519	-1.148068	0.992204
H	-5.762518	-1.267177	-1.022611
C	-3.732301	-1.536852	-1.608636
H	-3.369044	-2.566401	-1.520016
H	-4.113701	-1.396498	-2.625660
C	-2.491608	-0.650012	-1.444223
N	-2.674056	0.554928	-0.846316
O	-1.395825	-1.044505	-1.849515
H	-3.600511	0.791163	-0.513940
C	-1.558791	1.457223	-0.562481
H	-1.033719	1.669942	-1.499970
C	-0.496508	0.806592	0.348692
N	-0.875703	-0.228559	1.129661
O	0.664771	1.249662	0.344710
H	-1.815924	-0.616769	1.062823
C	0.098049	-0.929178	1.954243
H	0.620916	-0.229484	2.612488
H	-0.432732	-1.655158	2.577330
C	1.204392	-1.675547	1.187223
N	0.997526	-1.913840	-0.133149
O	2.226806	-2.018957	1.788158
H	0.153491	-1.582788	-0.594052
C	2.028352	-2.533777	-0.951018
H	2.386079	-3.455838	-0.483357
H	1.597353	-2.789314	-1.923430
C	3.292139	-1.688968	-1.205809
N	3.233212	-0.374326	-0.842815
O	4.275428	-2.221154	-1.715497
H	2.379333	-0.002935	-0.438673
C	4.405702	0.479478	-0.865305
H	5.153720	0.015061	-1.514431
H	4.162860	1.464284	-1.277238
C	5.081939	0.728739	0.496813
N	4.670860	-0.050775	1.535693
O	5.949388	1.594908	0.594746
H	3.969622	-0.779573	1.450617
H	5.142255	0.075088	2.420776
C	-2.118237	2.769945	0.035482
H	-3.076181	2.994709	-0.445294
H	-2.321373	2.647919	1.107409
C	-1.309833	4.073268	-0.130703
O	-1.910536	5.145403	-0.116825
N	0.036573	3.959402	-0.287708
H	0.520177	3.073677	-0.131027
H	0.563009	4.823090	-0.317984

-1457.610916

N Hairpin/-gauche

H	5.791536	3.244453	1.190353
C	5.186752	3.557405	0.332756
C	4.049214	2.566027	0.155187
N	2.797943	3.057395	0.289134
O	4.270134	1.370368	-0.097898
H	4.844249	4.584165	0.493325
H	5.826852	3.523131	-0.554425
H	2.617113	4.041986	0.458043
C	1.624154	2.218472	0.155204
H	1.703207	1.597189	-0.742511
H	1.522100	1.536072	1.009695
C	0.381767	3.106767	0.054465
N	-0.755455	2.427825	-0.236501
O	0.427650	4.329195	0.232192
H	-0.706640	1.419291	-0.404867
C	-2.025317	3.112025	-0.368852
H	-2.270666	3.656146	0.553233
H	-1.987267	3.859236	-1.175438
C	-3.109753	2.094260	-0.722289
N	-4.282024	2.208176	-0.020411
O	-2.942310	1.210348	-1.563155
H	-4.388197	2.979935	0.624815
C	-5.456126	1.418716	-0.380255
H	-6.323870	1.810179	0.159235
H	-5.661024	1.497357	-1.452863
C	-5.374089	-0.091925	-0.090776
N	-4.343342	-0.498390	0.708854
O	-6.216335	-0.846286	-0.565242
H	-3.688654	0.203827	1.027438
C	-4.034643	-1.896634	0.947493
H	-3.936895	-2.104861	2.017511
H	-4.869750	-2.486505	0.554715
C	-2.734004	-2.381792	0.284364
N	-2.429895	-1.836394	-0.925846
O	-2.043515	-3.251662	0.825397
H	-2.882945	-0.970317	-1.206422
C	-1.162005	-2.156170	-1.555445
H	-1.023901	-3.241359	-1.570720
H	-1.184659	-1.788434	-2.586976
C	0.004903	-1.464199	-0.831940
N	1.148628	-2.168486	-0.715013
O	-0.121521	-0.313485	-0.393636
H	1.247637	-3.098875	-1.112238
C	2.348890	-1.638877	-0.074453
H	2.333530	-0.549876	-0.136879
C	3.560179	-2.213642	-0.829384
N	4.628536	-1.388079	-0.886451
O	3.509244	-3.360187	-1.293081
H	4.525422	-0.424678	-0.560941
C	5.885205	-1.785030	-1.502824
H	5.845083	-2.855272	-1.715527
H	6.053969	-1.243785	-2.442566
H	6.722601	-1.579463	-0.826050
C	2.433249	-2.057916	1.416681
H	2.346215	-3.147838	1.486708
H	3.410821	-1.758672	1.806347
C	1.387889	-1.350096	2.283698
N	0.260831	-2.074225	2.573349
O	1.576695	-0.215616	2.722693
H	-0.039951	-2.841914	1.982582
H	-0.480404	-1.561600	3.034678

-1704.9335555

N Hairpin/trans

H	5.801725	3.591211	-0.468360
C	4.922506	3.744029	-1.102796
C	3.899113	2.668521	-0.778633
N	2.709791	3.096769	-0.302551
O	4.155611	1.465914	-0.946648
H	4.544466	4.759696	-0.951831
H	5.240109	3.625990	-2.143528
H	2.484650	4.082504	-0.206870
C	1.642329	2.183629	0.050089
H	1.428106	1.504265	-0.784254
H	1.921790	1.566608	0.914795
C	0.383687	2.996514	0.361733
N	-0.704443	2.234657	0.641597
O	0.387001	4.232006	0.353940
H	-0.638065	1.218594	0.543896
C	-2.004305	2.821873	0.849581
H	-2.278923	2.833481	1.916089
H	-1.962814	3.866301	0.515657
C	-3.085402	2.082054	0.052619
N	-4.352927	2.183292	0.576657
O	-2.853296	1.425532	-0.959096
H	-4.501641	2.808661	1.358483
C	-5.512236	1.733495	-0.188539
H	-6.418744	2.137702	0.272548
H	-5.463646	2.098983	-1.219363
C	-5.695505	0.209564	-0.303000
N	-4.933842	-0.552867	0.536000
O	-6.502930	-0.244512	-1.106950
H	-4.302783	-0.074857	1.165700
C	-4.911102	-2.002484	0.478747
H	-5.122550	-2.444071	1.458179
H	-5.700801	-2.315428	-0.211739
C	-3.588223	-2.624913	0.001743
N	-2.763938	-1.816487	-0.726817
O	-3.341322	-3.803032	0.257242
H	-2.981671	-0.830374	-0.839967
C	-1.470179	-2.281228	-1.166622
H	-1.445410	-3.368650	-1.045165
H	-1.323833	-2.051211	-2.230778
C	-0.314959	-1.625158	-0.394269
N	0.892217	-2.214757	-0.548334
O	-0.473493	-0.594431	0.273141
H	0.995511	-3.108586	-1.020322
C	2.113231	-1.662526	0.028988
H	2.079903	-0.575401	-0.049190
C	3.287454	-2.248980	-0.774582
N	4.267037	-1.375614	-1.087660
O	3.282387	-3.455355	-1.059232
H	4.134073	-0.375940	-0.911374
C	5.454632	-1.777033	-1.827774
H	5.474029	-2.866639	-1.894409
H	5.443404	-1.358864	-2.842015
H	6.356806	-1.422929	-1.316384
C	2.235594	-2.037232	1.527832
H	2.412873	-3.113697	1.624187
H	1.282278	-1.794090	2.008244
C	3.300710	-1.200075	2.232918
O	3.229628	0.026885	2.284346
N	4.317310	-1.901779	2.823541
H	5.074933	-1.378235	3.242196
H	4.435042	-2.891878	2.666387

-1704.991028

P **Helix**

H	-5.430730	-1.297602	2.462325
C	-5.525187	-1.827639	1.510243
C	-4.362139	-1.438762	0.613670
N	-4.687295	-1.042810	-0.654484
O	-3.195000	-1.469280	1.014110
H	-5.452242	-2.899727	1.722560
H	-6.506566	-1.618792	1.072786
H	-5.657816	-1.106876	-0.930908
C	-3.700426	-0.852783	-1.716619
H	-3.326938	-1.813831	-2.085557
H	-4.198714	-0.339931	-2.548702
C	-2.434128	-0.063956	-1.345987
N	-2.547003	1.104252	-0.663439
O	-1.337121	-0.505659	-1.709902
C	-1.330512	1.873462	-0.335964
H	-0.814798	2.158783	-1.259926
C	-0.275064	1.131041	0.492968
N	-0.679664	0.064318	1.229134
O	0.888864	1.546783	0.484970
H	-1.609540	-0.331111	1.098985
C	0.295506	-0.723722	1.965418
H	0.851611	-0.093844	2.665719
H	-0.236039	-1.488756	2.539738
C	1.366444	-1.423831	1.109642
N	1.107126	-1.559919	-0.215831
O	2.407440	-1.821622	1.643746
H	0.251934	-1.178002	-0.613837
C	2.109715	-2.094678	-1.121809
H	2.496677	-3.046688	-0.746292
H	1.643397	-2.276854	-2.094549
C	3.354639	-1.215485	-1.356198
N	3.308497	0.057848	-0.869675
O	4.316022	-1.690860	-1.958348
H	2.472010	0.395751	-0.401010
C	4.477394	0.916344	-0.868344
H	5.172747	0.559339	-1.633270
H	4.197472	1.945946	-1.112824
C	5.264542	0.980166	0.455324
N	4.888341	0.110973	1.433715
O	6.182102	1.790664	0.577960
H	4.141185	-0.567911	1.327524
H	5.422928	0.122615	2.291127
C	-1.874790	3.116376	0.400998
H	-1.941320	2.914145	1.476410
H	-1.223188	3.980799	0.257308
C	-3.281972	3.296452	-0.193709
H	-3.942868	3.893798	0.441751
H	-3.220517	3.779306	-1.175981
C	-3.779217	1.849949	-0.348583
H	-4.222943	1.487916	0.588068
H	-4.523032	1.741062	-1.143976

-1366.2925575

P **Hairpin**

H	1.838798	2.865631	4.133101
C	1.751169	3.652123	3.376907
C	1.809578	3.011443	2.000563
N	0.746797	3.236956	1.186322
O	2.776402	2.317802	1.650795
H	0.832834	4.221028	3.552064
H	2.613998	4.316011	3.491185
H	-0.026621	3.828472	1.468309
C	0.688527	2.688526	-0.153519
H	1.274853	3.297776	-0.858363
H	1.110166	1.680957	-0.170325
C	-0.750125	2.669767	-0.659355
N	-0.954761	1.786987	-1.667072
O	-1.617793	3.440132	-0.216391
H	-0.319950	0.980966	-1.720310
C	-2.232119	1.706118	-2.359298
H	-2.631551	2.718572	-2.476217
H	-2.073637	1.259220	-3.343554
C	-3.243569	0.816293	-1.621176
N	-3.836314	1.388902	-0.533433
O	-3.473254	-0.344406	-1.975435
H	-3.537508	2.326801	-0.275253
C	-4.928227	0.723604	0.166760
H	-5.320658	1.406284	0.927602
H	-5.741453	0.482697	-0.525148
C	-4.581097	-0.607713	0.856805
N	-3.268125	-0.773196	1.198121
O	-5.459618	-1.429401	1.094100
H	-2.621312	-0.030115	0.966803
C	-2.744736	-2.022923	1.716658
H	-2.400816	-1.927048	2.752629
H	-3.563441	-2.750506	1.701471
C	-1.550710	-2.573272	0.924395
N	-1.480315	-2.236951	-0.393427
O	-0.705869	-3.276333	1.485371
H	-2.210340	-1.673084	-0.822804
C	-0.295547	-2.564923	-1.177728
H	-0.027821	-3.612138	-1.026180
H	-0.531698	-2.409407	-2.234188
C	0.854466	-1.602014	-0.829606
N	1.967844	-2.072548	-0.245457
O	0.712654	-0.384528	-1.074412
C	3.036479	-1.140231	0.163036
H	2.627831	-0.432322	0.895495
C	3.640812	-0.371187	-1.027604
N	3.931215	0.928769	-0.749526
O	3.907748	-0.938234	-2.087491
H	3.571730	1.350363	0.107169
C	4.574168	1.792829	-1.725448
H	5.043100	1.165372	-2.486623
H	3.851848	2.454824	-2.223474
H	5.337119	2.410909	-1.238926
C	4.096425	-2.060733	0.809229
H	4.816178	-2.366751	0.042343
H	4.635786	-1.549597	1.611464
C	2.240258	-3.460239	0.183395
H	1.327804	-3.928520	0.555591
H	2.652234	-4.035113	-0.656569
C	3.287522	-3.276872	1.290382
H	3.903272	-4.171350	1.427326
H	2.783053	-3.056187	2.238013

-1613.6749162

Q Helix/-gauche

H	-5.256540	-0.963656	1.813977
C	-5.097799	-2.017073	1.554475
C	-3.841562	-2.117054	0.709532
N	-4.021023	-1.998441	-0.642060
O	-2.715603	-2.249467	1.201926
H	-4.950634	-2.585873	2.474811
H	-5.988687	-2.381876	1.032551
H	-4.954902	-1.850152	-0.998204
C	-2.903307	-2.073604	-1.570687
H	-2.373657	-3.025829	-1.464291
H	-3.288101	-2.015824	-2.594199
C	-1.829692	-0.988959	-1.394443
N	-2.201211	0.130299	-0.732102
O	-0.689317	-1.180338	-1.833694
H	-3.163159	0.253735	-0.406105
C	-1.239486	1.166926	-0.389653
H	-0.763484	1.548496	-1.301486
C	-0.058259	0.654380	0.462568
N	-0.251872	-0.476122	1.186571
O	1.003030	1.288715	0.465402
H	-1.125691	-0.996812	1.117388
C	0.845224	-1.062884	1.939470
H	1.243553	-0.351584	2.669368
H	0.466971	-1.934687	2.482165
C	2.057527	-1.506857	1.101090
N	1.857088	-1.697322	-0.227695
O	3.150845	-1.668763	1.654104
H	0.948756	-1.506313	-0.645760
C	2.967623	-2.005972	-1.113419
H	3.540519	-2.854752	-0.728278
H	2.567911	-2.281182	-2.093835
C	4.001200	-0.882075	-1.327255
N	3.673177	0.352264	-0.849013
O	5.054421	-1.140859	-1.907622
H	2.774499	0.503512	-0.398173
C	4.628814	1.442382	-0.830396
H	5.409983	1.231908	-1.566577
H	4.143904	2.384586	-1.104900
C	5.338168	1.692935	0.514561
N	5.153427	0.758580	1.487391
O	6.034474	2.697734	0.656576
H	4.580721	-0.071519	1.370331
H	5.640856	0.899742	2.361183
C	-1.916421	2.332065	0.357722
H	-2.306525	1.975506	1.318338
H	-1.124652	3.053637	0.579265
C	-3.038366	3.053929	-0.420642
H	-2.844220	3.006526	-1.501306
H	-3.042928	4.118082	-0.157265
C	-4.438347	2.482307	-0.186455
O	-4.653602	1.289786	0.068573
N	-5.459928	3.377101	-0.292368
H	-5.298719	4.356664	-0.474378
H	-6.409259	3.046002	-0.180625

-1496.9399048

Q Helix/trans

H	-5.591325	-1.208976	2.311671
C	-5.678507	-1.998468	1.559761
C	-4.395642	-2.053305	0.751493
N	-4.535727	-2.051830	-0.616637
O	-3.282730	-2.080290	1.282847
H	-5.791445	-2.948770	2.092933
H	-6.570979	-1.824550	0.950661
H	-5.468821	-2.147607	-0.995569
C	-3.406907	-2.377262	-1.485935
H	-2.975785	-3.350989	-1.229676
H	-3.765245	-2.424390	-2.519736
C	-2.236362	-1.386062	-1.435944
N	-2.520185	-0.125776	-1.024343
O	-1.106177	-1.756037	-1.764203
H	-3.469550	0.084262	-0.742302
C	-1.485091	0.889026	-0.835422
H	-0.948201	1.031255	-1.780155
C	-0.394132	0.458346	0.165739
N	-0.716731	-0.482987	1.088791
O	0.725168	0.982573	0.106017
H	-1.623298	-0.946304	1.068040
C	0.292877	-0.986946	2.006966
H	0.720289	-0.173592	2.600865
H	-0.182742	-1.696557	2.691018
C	1.496743	-1.685943	1.349519
N	1.338362	-2.133444	0.077465
O	2.546695	-1.811723	1.988178
H	0.471171	-1.960039	-0.425244
C	2.456766	-2.701501	-0.658335
H	2.935406	-3.493729	-0.075173
H	2.078902	-3.141838	-1.585748
C	3.593309	-1.727785	-1.030436
N	3.350205	-0.399709	-0.836745
O	4.646036	-2.180921	-1.476070
H	2.447571	-0.092868	-0.483847
C	4.390923	0.599565	-0.979852
H	5.211953	0.160086	-1.554024
H	4.020228	1.472544	-1.526649
C	4.991638	1.131163	0.335590
N	4.689109	0.437879	1.468038
O	5.712795	2.128140	0.317998
H	4.092789	-0.383494	1.482394
H	5.110151	0.754598	2.330300
C	-2.155500	2.210542	-0.413695
H	-2.617018	2.090568	0.575043
H	-2.966997	2.432711	-1.116056
C	-1.198872	3.406833	-0.394360
H	-0.717454	3.517058	-1.375771
H	-0.388084	3.256745	0.325293
C	-1.949210	4.707435	-0.095835
O	-3.168029	4.818937	-0.205141
N	-1.149511	5.750195	0.292630
H	-0.146523	5.659645	0.363312
H	-1.575894	6.654667	0.441472

-1496.9316784

Q Hairpin/-gauche

H	5.412284	3.516366	2.151659
C	4.884967	3.989475	1.317063
C	3.830024	3.026494	0.800813
N	2.550055	3.465397	0.821484
O	4.131019	1.896939	0.384898
H	4.471513	4.946839	1.648416
H	5.617141	4.168313	0.523090
H	2.293372	4.393531	1.143955
C	1.457679	2.639394	0.355839
H	1.600470	2.352098	-0.693248
H	1.394264	1.704111	0.922162
C	0.145678	3.416343	0.499196
N	-0.952993	2.713556	0.132060
O	0.113575	4.579583	0.915335
H	-0.844542	1.746974	-0.193125
C	-2.273019	3.307106	0.205545
H	-2.499768	3.625704	1.232207
H	-2.330993	4.207382	-0.423470
C	-3.306461	2.305767	-0.307475
N	-4.449881	2.197845	0.438263
O	-3.118471	1.618692	-1.312806
H	-4.574557	2.819444	1.226658
C	-5.588701	1.412812	-0.031364
H	-6.445589	1.613294	0.619011
H	-5.863262	1.699358	-1.051584
C	-5.384520	-0.113589	-0.078075
N	-4.312789	-0.600832	0.616451
O	-6.172726	-0.811193	-0.706066
H	-3.708528	0.064121	1.080910
C	-3.876140	-1.982154	0.523143
H	-3.688582	-2.403966	1.515296
H	-4.683142	-2.551861	0.050401
C	-2.588913	-2.188784	-0.293046
N	-2.370253	-1.322752	-1.315657
O	-1.827309	-3.126798	-0.029211
H	-2.910229	-0.462533	-1.367700
C	-1.120346	-1.362606	-2.052137
H	-0.939274	-2.377093	-2.420510
H	-1.206752	-0.686560	-2.909213
C	0.053314	-0.885235	-1.177086
N	1.212046	-1.547878	-1.332653
O	-0.094622	0.061563	-0.385471
H	1.283896	-2.312729	-1.996706
C	2.416005	-1.318985	-0.531805
H	2.332204	-0.334044	-0.067280
C	3.618159	-1.388861	-1.487687
N	4.587039	-0.470930	-1.262100
O	3.656611	-2.268920	-2.356098
H	4.409469	0.291949	-0.608497
C	5.817088	-0.438748	-2.038968
H	5.900857	-1.374986	-2.594581
H	5.817322	0.395289	-2.752976
H	6.680880	-0.330352	-1.373527
C	2.550844	-2.419946	0.547826
H	3.520912	-2.324235	1.047085
H	2.555669	-3.389251	0.034762
C	1.414749	-2.370632	1.592928
H	0.473124	-2.087731	1.117434
H	1.646012	-1.618028	2.355028
C	1.258956	-3.717065	2.298535
O	2.110327	-4.158877	3.069360
N	0.110134	-4.394253	1.987208
H	-0.563428	-4.046162	1.311005
H	-0.007660	-5.318949	2.378957

-1744.314385

Q Hairpin/trans

H	5.401086	3.827509	0.159408
C	4.602816	4.057147	-0.553806
C	3.555871	2.959409	-0.483294
N	2.302252	3.340737	-0.156172
O	3.845520	1.772559	-0.713394
H	4.201075	5.051123	-0.335683
H	5.044535	4.058283	-1.555451
H	2.050284	4.310068	0.016030
C	1.211693	2.394186	-0.064098
H	1.091916	1.840220	-1.002381
H	1.393477	1.649673	0.719928
C	-0.081429	3.155119	0.246233
N	-1.168349	2.355875	0.349838
O	-0.096495	4.383230	0.384704
H	-1.063865	1.350803	0.175611
C	-2.484916	2.897148	0.593123
H	-2.670192	3.045068	1.668355
H	-2.562200	3.885089	0.120360
C	-3.552097	1.977196	-0.003486
N	-4.745157	1.954972	0.672775
O	-3.357759	1.286432	-1.002330
H	-4.871360	2.587809	1.452282
C	-5.918400	1.299220	0.101180
H	-6.799175	1.584284	0.684783
H	-6.077736	1.621437	-0.932847
C	-5.878404	-0.239455	0.046721
N	-4.914357	-0.847293	0.798782
O	-6.701321	-0.837797	-0.637916
H	-4.284856	-0.258503	1.327833
C	-4.664802	-2.276792	0.750359
H	-4.636550	-2.707717	1.756394
H	-5.495404	-2.738062	0.207218
C	-3.356471	-2.703101	0.061155
N	-2.776162	-1.787470	-0.768009
O	-2.909959	-3.833166	0.252891
H	-3.155876	-0.848467	-0.848865
C	-1.524261	-2.075865	-1.428827
H	-1.388851	-3.161282	-1.441129
H	-1.561468	-1.718402	-2.466357
C	-0.319970	-1.390898	-0.760441
N	0.875478	-1.965968	-1.003718
O	-0.442402	-0.348070	-0.099627
H	0.949471	-2.837844	-1.520451
C	2.146957	-1.401014	-0.557396
H	2.054560	-0.312820	-0.543523
C	3.205079	-1.833574	-1.587160
N	4.148895	-0.901674	-1.864216
O	3.161138	-2.966557	-2.082477
H	4.058513	0.028047	-1.453983
C	5.243482	-1.154694	-2.789059
H	5.218102	-2.208863	-3.072562
H	5.145315	-0.542448	-3.694548
H	6.207221	-0.927481	-2.317928
C	2.494515	-1.889534	0.867808
H	2.541728	-2.985273	0.865387
H	1.678032	-1.595018	1.534450
C	3.804296	-1.303397	1.417230
H	3.841081	-0.223427	1.217862
H	4.676670	-1.744570	0.921485
C	3.896900	-1.466533	2.936148
O	2.940705	-1.282685	3.683562
N	5.143485	-1.788929	3.415019
H	5.902037	-2.040945	2.799697
H	5.249268	-1.929021	4.410903

-1744.309958

R **Helix/-gauche**

H	-0.278379	5.421199	-2.518254
C	-1.169147	5.717644	-1.958067
C	-1.360833	4.774332	-0.787616
N	-2.643573	4.327152	-0.564177
O	-0.424870	4.407689	-0.070350
H	-0.987966	6.723572	-1.562254
H	-2.031011	5.756103	-2.631395
H	-3.380489	4.747358	-1.116334
C	-3.027691	3.726608	0.712180
H	-2.636803	4.315657	1.549976
H	-4.119118	3.731357	0.787536
C	-2.555449	2.292804	0.989461
N	-1.891144	1.641583	-0.014897
O	-2.775156	1.787409	2.087353
H	-1.743919	2.142875	-0.882248
C	-1.109279	0.439130	0.251166
H	-1.093593	0.347658	1.345797
C	0.365463	0.568191	-0.197496
N	0.913957	1.811799	-0.163812
O	1.001873	-0.437743	-0.524340
H	0.352558	2.630962	0.071926
C	2.272572	2.037654	-0.651972
H	2.365501	1.729695	-1.698605
H	2.478140	3.110969	-0.592839
C	3.402201	1.294571	0.079514
N	3.153209	0.875166	1.349159
O	4.471965	1.098552	-0.497468
H	2.250290	1.075674	1.756399
C	4.142200	0.124161	2.111689
H	5.108643	0.635945	2.082760
H	3.822094	0.070777	3.156409
C	4.424078	-1.313918	1.631119
N	3.585419	-1.815422	0.677984
O	5.364215	-1.930288	2.122846
H	2.839044	-1.233928	0.312194
C	3.853747	-3.070713	0.001374
H	4.571662	-3.635788	0.603155
H	2.941125	-3.668891	-0.088254
C	4.441467	-2.958951	-1.418827
N	4.876297	-1.728070	-1.809926
O	4.488324	-3.958763	-2.132162
H	4.876401	-0.911971	-1.206785
H	5.328593	-1.666672	-2.712101
C	-1.721454	-0.854150	-0.316190
H	-1.030213	-1.671455	-0.089183
H	-1.770954	-0.782699	-1.411489
C	-3.115033	-1.141318	0.257623
H	-3.790785	-0.306212	0.033876
H	-3.054439	-1.226359	1.350741
C	-3.694814	-2.432741	-0.326454
H	-3.032816	-3.276021	-0.085586
H	-3.783294	-2.352988	-1.417365
N	-5.035100	-2.681088	0.244960
H	-5.332618	-2.063488	0.991894
C	-5.816855	-3.721063	-0.050196
N	-5.385557	-4.680292	-0.888159
H	-4.429331	-4.695669	-1.214362
H	-6.009670	-5.399572	-1.227105
N	-7.058330	-3.813191	0.471137
H	-7.446654	-3.066237	1.030675
H	-7.586113	-4.673614	0.414055

-1572.1137305

R **Helix/trans**

H	5.554789	-1.521550	3.001607
C	6.160519	-1.530227	2.091683
C	5.339717	-0.986212	0.939863
N	5.431525	-1.661760	-0.252540
O	4.614199	0.007752	1.058407
H	7.010541	-0.857967	2.255816
H	6.542997	-2.539751	1.912563
H	6.129189	-2.392451	-0.318728
C	4.984885	-1.047748	-1.503322
H	5.468168	-0.078239	-1.664857
H	5.266591	-1.706540	-2.331486
C	3.480239	-0.778025	-1.622364
N	2.648125	-1.524105	-0.828580
O	3.067719	0.055603	-2.421988
H	3.061785	-2.134993	-0.135287
C	1.221852	-1.244444	-0.792042
H	0.957130	-0.889699	-1.795150
C	0.839444	-0.080150	0.153404
N	1.816018	0.631419	0.754799
O	-0.361118	0.205914	0.317258
H	2.800685	0.363889	0.681394
C	1.461731	1.674005	1.718045
H	0.967182	1.247422	2.596316
H	2.388067	2.160386	2.040816
C	0.490880	2.741255	1.182085
N	0.715822	3.189312	-0.085384
O	-0.426809	3.169799	1.885122
H	1.467702	2.778742	-0.625836
C	-0.148306	4.193938	-0.695768
H	-0.236305	5.068301	-0.044393
H	0.297873	4.519748	-1.640101
C	-1.593393	3.741637	-0.985423
N	-1.811145	2.392740	-0.912917
O	-2.457368	4.563383	-1.267455
H	-1.062326	1.782356	-0.598281
C	-3.130781	1.810524	-0.968202
H	-3.849063	2.615107	-1.168762
H	-3.220144	1.067098	-1.768161
C	-3.529922	1.111197	0.335858
N	-3.131228	1.680784	1.482225
O	-4.218818	0.064470	0.299035
H	-2.424795	2.415746	1.502402
H	-3.356571	1.216892	2.352991
C	0.388396	-2.512512	-0.467570
H	0.868089	-3.367428	-0.959342
H	0.419658	-2.699555	0.614743
C	-1.077284	-2.368243	-0.931597
H	-1.142554	-2.500898	-2.017965
H	-1.374988	-1.342893	-0.708361
C	-2.071180	-3.309305	-0.225115
H	-1.726697	-3.540093	0.792478
H	-2.163340	-4.256535	-0.772314
N	-3.379915	-2.638476	-0.155618
H	-3.388274	-1.612172	-0.174443
C	-4.525418	-3.176550	0.270285
N	-4.663965	-4.516493	0.393540
H	-3.977475	-5.142178	-0.000660
H	-5.534376	-4.916518	0.713026
N	-5.538652	-2.359926	0.601634
H	-5.363555	-1.349842	0.590744
H	-6.476799	-2.711060	0.727179

-1572.1526123

R **Hairpin/-gauche**

H	6.379077	-1.526424	-2.787629
C	6.069086	-2.236798	-2.014826
C	4.776222	-1.747489	-1.391849
N	3.726177	-2.600159	-1.416516
O	4.681429	-0.616542	-0.883237
H	5.985311	-3.234751	-2.454727
H	6.848781	-2.248217	-1.246202
H	3.797067	-3.545108	-1.782466
C	2.458936	-2.246473	-0.817326
H	2.583636	-2.012860	0.248603
H	2.032498	-1.351566	-1.283667
C	1.485904	-3.419619	-0.957825
N	0.228058	-3.139590	-0.528755
O	1.830589	-4.512367	-1.410646
H	0.019988	-2.211462	-0.158826
C	-0.801345	-4.159412	-0.500116
H	-1.054651	-4.495527	-1.514864
H	-0.450160	-5.046019	0.047240
C	-2.028522	-3.604022	0.218057
N	-3.242218	-3.966068	-0.294674
O	-1.932404	-2.838882	1.182423
H	-3.267521	-4.648951	-1.041904
C	-4.485123	-3.644424	0.405950
H	-5.315876	-4.133349	-0.111960
H	-4.463849	-4.014384	1.436014
C	-4.815020	-2.145188	0.514844
N	-4.147599	-1.311228	-0.349047
O	-5.636505	-1.751096	1.330746
H	-3.470352	-1.718738	-0.980004
C	-4.224944	0.128234	-0.237492
H	-4.296077	0.591974	-1.226895
H	-5.134251	0.371503	0.324036
C	-3.043925	0.800511	0.484363
N	-2.331964	0.029567	1.320500
O	-2.837605	2.021758	0.291876
H	-2.495961	-0.978322	1.308998
C	-1.151420	0.468718	2.039153
H	-1.300276	1.490396	2.401978
H	-1.013279	-0.185374	2.906765
C	0.112309	0.408727	1.148861
N	1.171809	1.119242	1.592719
O	0.107965	-0.222250	0.081872
H	1.146111	1.608713	2.485111
C	2.317227	1.477637	0.751307
H	2.516362	0.650056	0.069284
C	3.495012	1.725870	1.710577
N	4.697797	1.304647	1.270831
O	3.278633	2.326284	2.771476
H	4.734096	0.698569	0.448733
C	5.920541	1.509081	2.038440
H	5.724203	2.244316	2.821017
H	6.253144	0.575336	2.508457
H	6.717338	1.876115	1.382852
C	2.054405	2.794175	-0.076596
H	2.477762	3.649052	0.464399
H	2.583066	2.710789	-1.032466
C	0.552947	3.001011	-0.282535
H	0.078606	3.063193	0.701466
H	0.143727	2.114289	-0.778083
C	0.046995	4.218112	-1.061572
H	0.338709	5.159491	-0.576299
H	0.410636	4.223398	-2.097929
N	-1.418247	4.053732	-1.036041
H	-1.775726	3.260691	-0.484987
C	-2.349712	4.831811	-1.565663
N	-3.644094	4.447567	-1.457684
N	-2.037521	5.973733	-2.214941
H	-4.384032	5.124833	-1.581080
H	-3.833837	3.634525	-0.871728
H	-2.732185	6.463453	-2.760831
H	-1.071722	6.242257	-2.337744

-1819.526349

R **Hairpin/trans**

H	-4.142204	4.546856	-1.196898
C	-3.261645	4.876511	-0.636699
C	-2.387316	3.673250	-0.334868
N	-1.095482	3.756279	-0.709626
O	-2.846875	2.662697	0.236335
H	-2.742924	5.654379	-1.204162
H	-3.612682	5.301738	0.309523
H	-0.709488	4.575199	-1.173854
C	-0.144706	2.697869	-0.445391
H	-0.022044	2.538263	0.633039
H	-0.480783	1.744367	-0.865461
C	1.207774	3.087248	-1.055472
N	2.163776	2.139700	-0.918746
O	1.375189	4.176694	-1.611449
H	1.939307	1.275848	-0.416608
C	3.521026	2.364049	-1.378038
H	3.580267	2.328843	-2.475126
H	3.858464	3.364134	-1.074229
C	4.444232	1.322172	-0.744396
N	5.420769	0.819100	-1.558598
O	4.294706	0.936331	0.416024
H	5.536485	1.217926	-2.481523
C	6.464686	-0.060511	-1.033808
H	7.231620	-0.191634	-1.803078
H	6.939827	0.383637	-0.153568
C	6.002607	-1.461253	-0.586044
N	4.770485	-1.862435	-1.025847
O	6.737109	-2.144381	0.116198
H	4.233530	-1.211494	-1.582766
C	4.112065	-3.059974	-0.532663
H	3.687370	-3.643791	-1.354937
H	4.870786	-3.670879	-0.032469
C	2.968368	-2.799470	0.464505
N	3.083240	-1.675426	1.228643
O	2.036122	-3.596585	0.576945
H	3.763000	-0.959444	0.988008
C	2.025404	-1.310816	2.139021
H	1.732200	-2.181590	2.734744
H	2.398904	-0.536254	2.818714
C	0.793548	-0.740180	1.416295
N	-0.380678	-0.880627	2.073297
O	0.879146	-0.148252	0.329001
H	-0.443579	-1.424417	2.928893
C	-1.646850	-0.396509	1.538209
H	-1.458801	0.512249	0.962955
C	-2.569382	-0.107021	2.735573
N	-3.293447	1.034501	2.637842
O	-2.625081	-0.909346	3.673065
H	-3.125429	1.654102	1.843238
C	-4.197365	1.472716	3.694524
H	-4.368020	0.636964	4.376084
H	-3.766324	2.305283	4.264225
H	-5.152354	1.799229	3.268012
C	-2.267077	-1.466877	0.610165
H	-2.441805	-2.373545	1.203458
H	-1.520555	-1.708293	-0.156168
C	-3.573815	-1.012379	-0.056125
H	-3.395829	-0.102326	-0.644503
H	-4.318514	-0.766378	0.711373
C	-4.131960	-2.115754	-0.957944
H	-3.409990	-2.368565	-1.744876
H	-4.332406	-3.012940	-0.356463
N	-5.388901	-1.660358	-1.590177
H	-5.735324	-0.749666	-1.310168
C	-6.130342	-2.378548	-2.432550
N	-7.211853	-1.834516	-3.031303
N	-5.801602	-3.653116	-2.711141
H	-7.890260	-2.413288	-3.507311
H	-7.443893	-0.859577	-2.900305
H	-6.260751	-4.161638	-3.454039
H	-5.039978	-4.109427	-2.228873

-1819.5073707

S Helix/-gauche

H	-5.748459	-0.252958	2.381079
C	-5.927471	-0.948109	1.555683
C	-4.674467	-1.032389	0.703640
N	-4.843436	-0.841592	-0.647600
O	-3.560901	-1.242642	1.190277
H	-6.115260	-1.934886	1.992477
H	-6.814409	-0.630022	0.998991
H	-5.788740	-0.796137	-1.004526
C	-3.768658	-1.148018	-1.587899
H	-3.446288	-2.191348	-1.502027
H	-4.139437	-0.988236	-2.606250
C	-2.495229	-0.312122	-1.404046
N	-2.644783	0.906160	-0.828971
O	-1.405094	-0.766284	-1.761654
H	-3.577365	1.265006	-0.660165
C	-1.515757	1.779984	-0.547603
H	-0.999972	2.046420	-1.478841
C	-0.432690	1.130799	0.334659
N	-0.824171	0.124462	1.157704
O	0.723184	1.562900	0.286869
H	-1.770175	-0.249544	1.104275
C	0.151625	-0.576525	1.977640
H	0.683597	0.122544	2.629800
H	-0.377167	-1.297374	2.608653
C	1.250682	-1.330128	1.207319
N	1.023836	-1.590539	-0.104928
O	2.285197	-1.658202	1.797640
H	0.170638	-1.267367	-0.555298
C	2.052018	-2.196501	-0.935278
H	2.434168	-3.109053	-0.468110
H	1.610332	-2.466697	-1.898921
C	3.297605	-1.332096	-1.215823
N	3.229164	-0.018835	-0.852819
O	4.278515	-1.852564	-1.743871
H	2.377107	0.351875	-0.441584
C	4.392528	0.846214	-0.897840
H	5.114866	0.420055	-1.599817
H	4.116429	1.843771	-1.253971
C	5.134565	1.048997	0.437819
N	4.739175	0.270579	1.482961
O	6.036067	1.882954	0.510490
H	4.007366	-0.430214	1.419487
H	5.248140	0.371084	2.350190
C	-2.028727	3.055164	0.152193
H	-1.183852	3.739385	0.295950
H	-2.434455	2.797700	1.137214
O	-3.105864	3.661858	-0.557866
H	-2.750533	4.088579	-1.350897

-1364.1053518

S Helix/trans

H	-5.915664	0.051211	2.284547
C	-6.054532	-0.705632	1.507626
C	-4.761281	-0.858264	0.728433
N	-4.875288	-0.876702	-0.643918
O	-3.663850	-0.946592	1.282469
H	-6.268579	-1.655890	2.009495
H	-6.911191	-0.436105	0.881910
H	-5.807206	-0.927966	-1.034763
C	-3.764042	-1.324857	-1.482341
H	-3.414888	-2.318248	-1.180489
H	-4.112676	-1.387553	-2.518520
C	-2.515428	-0.432653	-1.460179
N	-2.689811	0.855431	-1.067071
O	-1.425944	-0.900579	-1.794368
H	-3.613648	1.122183	-0.748705
C	-1.568932	1.767453	-0.846031
H	-1.040942	1.939774	-1.790317
C	-0.491132	1.207092	0.104473
N	-0.912903	0.366541	1.089534
O	0.687429	1.531956	-0.045293
H	-1.870279	0.022468	1.107164
C	0.047330	-0.225456	2.007579
H	0.618688	0.554469	2.519988
H	-0.498843	-0.798373	2.763302
C	1.103681	-1.153320	1.381926
N	0.872453	-1.610240	0.124660
O	2.112377	-1.442068	2.034380
H	0.061743	-1.288238	-0.396747
C	1.880596	-2.379407	-0.586762
H	2.220737	-3.219883	0.025118
H	1.435182	-2.781414	-1.501559
C	3.163672	-1.621315	-0.982674
N	3.148567	-0.266065	-0.831635
O	4.125906	-2.259024	-1.407082
H	2.307366	0.202409	-0.505565
C	4.348318	0.531332	-1.000075
H	5.050951	-0.025364	-1.626527
H	4.114054	1.475286	-1.502168
C	5.101149	0.898894	0.293676
N	4.663467	0.316298	1.444201
O	6.047167	1.683764	0.243191
H	3.896435	-0.347347	1.483536
H	5.176344	0.524089	2.289742
C	-2.155739	3.104918	-0.328740
H	-2.668429	2.919878	0.625480
H	-2.908881	3.453473	-1.053862
O	-1.168275	4.078873	-0.074540
H	-1.016092	4.600941	-0.872982

-1364.0959299

S **Hairpin/-gauche**

H	4.728832	3.456929	2.276778
C	4.300506	3.841357	1.345369
C	3.422431	2.763443	0.732441
N	2.117084	3.076625	0.560354
O	3.885523	1.657752	0.409573
H	3.762552	4.770993	1.554542
H	5.129517	4.049748	0.661625
H	1.735904	3.980524	0.820396
C	1.179463	2.136646	-0.019587
H	1.585964	1.707745	-0.939909
H	0.984623	1.295419	0.660120
C	-0.142334	2.849221	-0.313762
N	-0.993242	2.113220	-1.078207
O	-0.390239	3.977541	0.125618
H	-0.689047	1.187685	-1.399852
C	-2.308198	2.595030	-1.462992
H	-2.487399	3.538356	-0.939414
H	-2.359086	2.777159	-2.544648
C	-3.353592	1.523068	-1.140734
N	-4.209180	1.797235	-0.108464
O	-3.381932	0.446472	-1.741924
H	-4.179213	2.714614	0.316683
C	-5.306200	0.892344	0.221299
H	-5.945136	1.371480	0.969683
H	-5.916070	0.683129	-0.663366
C	-4.895879	-0.485811	0.769360
N	-3.615074	-0.588559	1.234448
O	-5.708821	-1.403460	0.785572
H	-3.018608	0.226615	1.174061
C	-3.036569	-1.845676	1.665722
H	-2.605839	-1.764022	2.668658
H	-3.844766	-2.584499	1.696454
C	-1.917992	-2.378438	0.757887
N	-2.007248	-2.104393	-0.567812
O	-0.994458	-3.047124	1.246314
H	-2.681868	-1.417673	-0.900249
C	-0.934560	-2.499661	-1.477980
H	-0.696282	-3.555791	-1.319467
H	-1.284760	-2.353090	-2.502943
C	0.287853	-1.595421	-1.247155
N	1.376383	-2.179119	-0.704417
O	0.215820	-0.385134	-1.510018
H	1.362995	-3.176317	-0.524336
C	2.447681	-1.416631	-0.080936
H	2.341122	-0.381465	-0.401633
C	3.820199	-1.970449	-0.483667
N	4.811850	-1.043229	-0.436518
O	3.970469	-3.163853	-0.761036
H	4.567681	-0.084154	-0.191997
C	6.204124	-1.376310	-0.690482
H	6.270806	-2.451761	-0.868503
H	6.581506	-0.845773	-1.573800
H	6.831720	-1.114171	0.170277
C	2.309269	-1.474472	1.467612
H	2.542933	-2.493639	1.812467
O	1.029890	-1.057707	1.914554
H	3.043815	-0.788033	1.901180
H	0.406875	-1.797808	1.787914

-1611.4878863

S **Hairpin/trans**

H	6.132845	3.274228	0.320631
C	5.371502	3.444219	-0.447595
C	4.254353	2.433420	-0.256305
N	3.021955	2.936217	-0.019649
O	4.470045	1.212754	-0.313732
H	5.029462	4.481883	-0.390374
H	5.839849	3.271110	-1.421730
H	2.830846	3.934016	-0.008511
C	1.867089	2.086547	0.173864
H	1.735958	1.405833	-0.676253
H	1.979197	1.464514	1.070459
C	0.620535	2.967934	0.304407
N	-0.525055	2.269429	0.494574
O	0.687786	4.199615	0.236334
H	-0.495036	1.245678	0.478414
C	-1.809242	2.925501	0.542897
H	-2.133941	3.107623	1.579340
H	-1.716301	3.906654	0.060222
C	-2.871286	2.101834	-0.192473
N	-4.157638	2.298184	0.246893
O	-2.605348	1.304997	-1.089319
H	-4.328463	3.024410	0.930799
C	-5.290913	1.758599	-0.500000
H	-6.210229	2.222256	-0.129080
H	-5.200111	1.989904	-1.566153
C	-5.481794	0.232609	-0.427484
N	-4.777667	-0.417209	0.545756
O	-6.246693	-0.318349	-1.211833
H	-4.177726	0.135314	1.143731
C	-4.758176	-1.863000	0.669353
H	-4.997052	-2.177627	1.690489
H	-5.530612	-2.259968	0.003218
C	-3.427902	-2.545093	0.306559
N	-2.563871	-1.824755	-0.466819
O	-3.210476	-3.693948	0.689115
H	-2.769155	-0.859272	-0.707950
C	-1.276429	-2.361869	-0.836903
H	-1.283431	-3.432884	-0.612190
H	-1.109226	-2.238721	-1.915770
C	-0.108908	-1.671859	-0.113637
N	1.072358	-2.325114	-0.182628
O	-0.233784	-0.572326	0.444537
H	1.164175	-3.223508	-0.650636
C	2.317419	-1.751569	0.302993
H	2.302958	-0.669473	0.159037
C	3.462310	-2.391843	-0.494299
N	4.595932	-1.643348	-0.557370
O	3.344690	-3.531687	-0.956727
H	4.517154	-0.635057	-0.400155
C	5.761865	-2.119424	-1.294505
H	5.892500	-3.189580	-1.117262
H	5.650463	-1.964279	-2.375746
H	6.647727	-1.579004	-0.948705
C	2.503314	-2.022090	1.822017
H	2.632074	-3.104004	1.982074
O	3.563771	-1.267464	2.385882
H	1.591590	-1.700855	2.334476
H	4.380028	-1.495255	1.914948

-1611.4850511

T Helix/-gauche

H	-5.706117	-0.390487	2.320600
C	-5.851021	-1.156376	1.552970
C	-4.585655	-1.263116	0.721762
N	-4.741351	-1.156213	-0.639363
O	-3.474259	-1.419295	1.233773
H	-6.008730	-2.110668	2.066731
H	-6.742127	-0.915674	0.964951
H	-5.682282	-1.145865	-1.009784
C	-3.645652	-1.477134	-1.549463
H	-3.289249	-2.502146	-1.400302
H	-4.009542	-1.389797	-2.578970
C	-2.402086	-0.589522	-1.401872
N	-2.596994	0.646081	-0.885007
O	-1.296249	-1.026055	-1.734922
H	-3.539680	0.992023	-0.742702
C	-1.508727	1.578270	-0.628889
H	-1.006821	1.851382	-1.566110
C	-0.403663	0.978206	0.263818
N	-0.779679	0.012478	1.141497
O	0.753162	1.405321	0.183685
H	-1.720862	-0.376419	1.110649
C	0.206399	-0.634640	1.993015
H	0.723385	0.101273	2.616238
H	-0.312680	-1.335964	2.653478
C	1.323295	-1.403104	1.264228
N	1.105554	-1.740311	-0.031478
O	2.362697	-1.676936	1.873935
H	0.251674	-1.451114	-0.503827
C	2.149922	-2.367606	-0.824973
H	2.549681	-3.244941	-0.307891
H	1.717873	-2.698847	-1.773851
C	3.377853	-1.492709	-1.148051
N	3.277438	-0.163439	-0.858576
O	4.373312	-2.019348	-1.641923
H	2.413923	0.209639	-0.473699
C	4.420007	0.725319	-0.945651
H	5.163663	0.269338	-1.605522
H	4.127550	1.689145	-1.374352
C	5.133422	1.037422	0.384333
N	4.753875	0.307030	1.469238
O	6.001355	1.908864	0.418113
H	4.047886	-0.422156	1.440172
H	5.245723	0.479306	2.335049
C	-2.101865	2.852695	0.044098
C	-1.125310	4.025775	0.029710
H	-2.365946	2.598115	1.078045
O	-3.357207	3.193811	-0.561669
H	-3.172575	3.679432	-1.379858
H	-0.169442	3.747240	0.481264
H	-1.551686	4.871324	0.578550
H	-0.919509	4.344692	-1.001186

-1403.4307344

T Hairpin/-gauche

H	5.151134	3.491016	1.908060
C	4.661565	3.866427	1.003661
C	3.650449	2.834456	0.533781
N	2.363758	3.245320	0.451625
O	3.993179	1.677715	0.243163
H	4.215940	4.843362	1.214499
H	5.431918	3.979822	0.234176
H	2.073849	4.189205	0.687611
C	1.311894	2.352439	0.015046
H	1.565643	1.887220	-0.942413
H	1.168257	1.527864	0.724371
C	0.001852	3.135809	-0.106265
N	-1.021626	2.412043	-0.626967
O	-0.093244	4.312126	0.261580
H	-0.839288	1.454051	-0.944885
C	-2.343669	2.985504	-0.803781
H	-2.515552	3.728909	-0.018593
H	-2.437279	3.499937	-1.772163
C	-3.377971	1.859600	-0.791377
N	-4.362416	1.948126	0.155195
O	-3.308771	0.907295	-1.571458
H	-4.403337	2.776283	0.734468
C	-5.494415	1.024705	0.157489
H	-6.236718	1.381080	0.878288
H	-5.967855	0.991633	-0.829052
C	-5.175697	-0.441619	0.502950
N	-3.964654	-0.676122	1.090152
O	-6.002146	-1.313566	0.257944
H	-3.345873	0.110922	1.236251
C	-3.454469	-2.013717	1.329261
H	-3.089320	-2.120681	2.355223
H	-4.283524	-2.713734	1.181103
C	-2.293437	-2.427400	0.410860
N	-2.276973	-1.899977	-0.840412
O	-1.440900	-3.230161	0.814053
H	-2.875219	-1.107115	-1.062612
C	-1.136255	-2.142659	-1.715748
H	-0.949935	-3.218833	-1.782721
H	-1.377739	-1.752945	-2.708570
C	0.088664	-1.384697	-1.177828
N	1.160868	-2.128453	-0.842440
O	0.036783	-0.154416	-1.034847
H	1.141082	-3.129912	-0.998029
C	2.274844	-1.622722	-0.044111
H	2.163408	-0.539374	0.020783
C	3.591914	-2.018384	-0.723938
N	4.517035	-1.029823	-0.799456
O	3.749631	-3.173804	-1.135657
H	4.289554	-0.105735	-0.432569
C	5.842049	-1.247006	-1.358521
H	5.914832	-2.290577	-1.671743
H	6.010399	-0.599285	-2.227853
H	6.619511	-1.038704	-0.613019
C	2.184782	-2.224537	1.393624
H	2.315013	-3.315024	1.299115
O	0.927302	-1.919076	1.982669
C	3.270121	-1.669917	2.314702
H	0.235065	-2.477607	1.584014
H	3.146235	-2.097802	3.314356
H	3.189492	-0.579632	2.392051
H	4.270352	-1.918740	1.947585

-1650.811744

T Hairpin/trans

H	5.952455	3.600715	0.342139
C	5.177430	3.772065	-0.411973
C	4.106305	2.706067	-0.258580
N	2.856454	3.144955	0.011686
O	4.374175	1.500201	-0.375550
H	4.791860	4.790394	-0.305315
H	5.643619	3.663123	-1.396351
H	2.625327	4.132371	0.074042
C	1.738522	2.241454	0.178331
H	1.618457	1.601664	-0.704823
H	1.891794	1.579015	1.039245
C	0.462637	3.067714	0.373114
N	-0.655608	2.320838	0.540754
O	0.485170	4.303098	0.369838
H	-0.593547	1.300306	0.477370
C	-1.958527	2.931351	0.649533
H	-2.266696	3.046638	1.700563
H	-1.905208	3.939739	0.220065
C	-3.012209	2.117872	-0.108881
N	-4.293862	2.253994	0.366735
O	-2.744832	1.379298	-1.053718
H	-4.468557	2.937227	1.092690
C	-5.428929	1.728867	-0.387573
H	-6.350965	2.150917	0.024059
H	-5.363673	2.018664	-1.441233
C	-5.585606	0.197368	-0.393576
N	-4.847364	-0.487363	0.529083
O	-6.355064	-0.328019	-1.190950
H	-4.245484	0.045831	1.142567
C	-4.792613	-1.936872	0.573458
H	-5.005231	-2.311968	1.579871
H	-5.567624	-2.315845	-0.100147
C	-3.452646	-2.564224	0.152396
N	-2.623590	-1.782372	-0.598915
O	-3.197359	-3.724573	0.472079
H	-2.856209	-0.810103	-0.780579
C	-1.324532	-2.259399	-1.008610
H	-1.297018	-3.340895	-0.844157
H	-1.171447	-2.070762	-2.080077
C	-0.172455	-1.574655	-0.256317
N	1.026277	-2.189151	-0.361946
O	-0.327369	-0.510026	0.359513
H	1.145599	-3.042048	-0.903375
C	2.259116	-1.590660	0.125987
H	2.189201	-0.504940	0.033618
C	3.406027	-2.124371	-0.744208
N	4.514757	-1.336990	-0.767414
O	3.314185	-3.220431	-1.308117
H	4.408938	-0.345755	-0.534988
C	5.672238	-1.711459	-1.573196
H	5.856384	-2.783730	-1.471850
H	5.515602	-1.492881	-2.637739
H	6.545063	-1.153878	-1.221787
C	2.491642	-1.882345	1.644824
H	1.561990	-1.592242	2.146889
O	3.476668	-0.997528	2.172359
C	2.803081	-3.347857	1.958013
H	4.321868	-1.200220	1.741571
H	2.885830	-3.476169	3.042007
H	3.745592	-3.665820	1.496986
H	2.011167	-4.008386	1.588773

-1650.8092206

V **Helix/-gauche**

H	-5.741328	-0.627111	2.402626
C	-5.864578	-1.306854	1.554970
C	-4.582348	-1.321401	0.743075
N	-4.722194	-1.185478	-0.618931
O	-3.472027	-1.432675	1.267639
H	-6.027293	-2.311624	1.960515
H	-6.744997	-1.013006	0.974856
H	-5.659117	-1.218097	-0.999337
C	-3.611349	-1.481200	-1.522636
H	-3.214016	-2.486492	-1.345386
H	-3.980233	-1.439299	-2.552828
C	-2.401681	-0.541644	-1.420062
N	-2.614761	0.674629	-0.863269
O	-1.304386	-0.919273	-1.839009
H	-3.538083	0.868270	-0.497524
C	-1.529518	1.618019	-0.583793
H	-1.020066	1.867280	-1.521605
C	-0.425007	0.989511	0.294277
N	-0.797133	0.008514	1.159800
O	0.739998	1.386835	0.195793
H	-1.741633	-0.370109	1.142102
C	0.195507	-0.668104	1.980019
H	0.730904	0.048413	2.610405
H	-0.320258	-1.377265	2.634769
C	1.293828	-1.434754	1.221952
N	1.067181	-1.730162	-0.082699
O	2.329101	-1.747520	1.819823
H	0.226264	-1.397498	-0.547886
C	2.103230	-2.340324	-0.900453
H	2.491252	-3.241760	-0.417209
H	1.666154	-2.629282	-1.860798
C	3.342682	-1.470050	-1.191158
N	3.256667	-0.149407	-0.861737
O	4.333889	-1.993505	-1.697107
H	2.394925	0.224886	-0.473153
C	4.409746	0.727982	-0.922735
H	5.142680	0.291833	-1.607467
H	4.124222	1.711919	-1.308096
C	5.139102	0.976115	0.412142
N	4.746680	0.219560	1.474473
O	6.028637	1.824131	0.470102
H	4.027440	-0.495186	1.422719
H	5.249918	0.347429	2.341415
C	-2.121477	2.912089	0.035312
C	-1.032299	3.898337	0.488204
H	-2.699162	2.612061	0.924449
C	-3.073718	3.604404	-0.959802
H	-2.524752	3.932163	-1.851102
H	-0.387008	3.479649	1.264140
H	-1.502217	4.805850	0.884001
H	-0.388761	4.185442	-0.351163
H	-3.893457	2.958970	-1.296500
H	-3.520864	4.492244	-0.499214

-1367.5097018

V **Helix/trans**

C	-5.960255	-0.926033	1.497657
C	-4.675075	-1.092488	0.707790
O	-3.576804	-1.215486	1.254454
N	-4.796891	-1.083680	-0.662825
C	-3.696012	-1.530621	-1.514997
C	-2.432992	-0.659778	-1.472209
O	-1.348389	-1.146025	-1.803343
N	-2.585945	0.624953	-1.068647
C	-1.450485	1.532440	-0.899358
C	-0.425027	1.022457	0.135588
N	-0.847779	0.117644	1.057673
C	0.099302	-0.482108	1.983904
C	1.217012	-1.321559	1.339657
N	1.023519	-1.740936	0.063197
C	2.072062	-2.441379	-0.660558
C	3.324455	-1.615028	-1.015811
N	3.250167	-0.268090	-0.813378
C	4.415988	0.586119	-0.934714
C	5.128361	0.946052	0.383808
N	4.706455	0.298641	1.505183
C	-1.963440	2.965547	-0.590736
H	-2.745576	3.159563	-1.341144
C	-0.860517	4.017646	-0.791169
O	0.741229	1.429945	0.090586
O	2.234257	-1.577476	1.992491
O	4.315978	-2.194114	-1.456024
O	6.031843	1.781121	0.377231
C	-2.597453	3.088945	0.807599
H	-6.814440	-0.626928	0.882046
H	-6.192077	-1.879990	1.984331
H	-5.800302	-0.185923	2.286528
H	-5.731123	-1.105525	-1.050598
H	-3.364344	-2.537359	-1.238971
H	-4.048230	-1.560454	-2.551505
H	-3.506712	0.925503	-0.775904
H	-0.887559	1.558743	-1.838797
H	-1.796137	-0.250098	1.034169
H	0.615716	0.286619	2.566547
H	-0.451308	-1.124775	2.677807
H	0.188653	-1.466020	-0.448636
H	2.442518	-3.287030	-0.073628
H	1.654382	-2.830859	-1.593600
H	2.390126	0.148566	-0.467505
H	5.152999	0.079866	-1.564504
H	4.150111	1.534098	-1.413035
H	3.970967	-0.401149	1.510079
H	5.194953	0.502877	2.365904
H	-1.265805	5.021184	-0.617428
H	-0.025952	3.855652	-0.103283
H	-0.459956	3.985021	-1.810790
H	-3.045651	4.081123	0.929464
H	-3.387685	2.348182	0.988319
H	-1.844708	2.964383	1.593833

-1367.5101625

V Hairpin/-gauche/trans

H	5.757920	3.585673	1.040216
C	5.098416	3.828489	0.200876
C	4.008182	2.774012	0.118261
N	2.733931	3.215422	0.216287
O	4.280216	1.571957	-0.028782
H	4.709260	4.842954	0.329850
H	5.698876	3.785821	-0.713484
H	2.499121	4.199329	0.310912
C	1.604256	2.314491	0.143767
H	1.608280	1.748957	-0.795975
H	1.630963	1.572902	0.950134
C	0.314231	3.134649	0.242651
N	-0.814628	2.388182	0.222461
O	0.331827	4.367700	0.331274
H	-0.738481	1.368214	0.137664
C	-2.118706	3.008512	0.274376
H	-2.391470	3.291588	1.302791
H	-2.104195	3.936795	-0.312329
C	-3.173361	2.071291	-0.314168
N	-4.413154	2.151545	0.265694
O	-2.932446	1.282259	-1.227053
H	-4.573097	2.859491	0.970716
C	-5.562319	1.466082	-0.320078
H	-6.472990	1.826827	0.167860
H	-5.640800	1.684623	-1.389839
C	-5.556365	-0.071149	-0.217207
N	-4.668815	-0.612786	0.668140
O	-6.335649	-0.724049	-0.902631
H	-4.064541	0.019105	1.176429
C	-4.422472	-2.040773	0.764955
H	-4.477369	-2.382128	1.803523
H	-5.207562	-2.549348	0.196460
C	-3.059613	-2.509589	0.223937
N	-2.464773	-1.707153	-0.704760
O	-2.582842	-3.578086	0.605317
H	-2.845217	-0.789243	-0.917076
C	-1.162256	-2.040599	-1.236220
H	-1.075424	-3.129249	-1.297604
H	-1.072265	-1.623200	-2.246538
C	-0.005392	-1.456953	-0.403917
N	1.119736	-2.198548	-0.391915
O	-0.108172	-0.359956	0.169422
H	1.158706	-3.072888	-0.907593
C	2.398083	-1.776812	0.184826
H	2.338640	-0.702656	0.379903
C	3.463787	-2.093960	-0.882060
N	4.361364	-1.111837	-1.127686
O	3.449048	-3.196891	-1.444362
H	4.270201	-0.213525	-0.652390
C	5.447087	-1.273172	-2.083183
H	5.412623	-2.291663	-2.475072
H	5.344066	-0.565860	-2.915616
H	6.416474	-1.102730	-1.599572
C	2.704534	-2.528196	1.510296
H	2.725022	-3.599431	1.265244
C	4.084500	-2.135470	2.066287
C	1.605269	-2.286140	2.557172
H	4.269847	-2.655992	3.012923
H	1.814456	-2.867169	3.463150
H	0.618421	-2.584020	2.189792
H	1.555883	-1.227022	2.840071
H	4.141081	-1.056493	2.261223
H	4.894918	-2.395162	1.377767

-1614.891193

W **Helix/-gauche**

H	-3.220861	4.671334	-1.940650
C	-3.288496	4.904154	-0.874193
C	-2.322099	4.013761	-0.113227
N	-2.830238	3.372388	0.991615
O	-1.151643	3.860207	-0.468541
H	-2.973594	5.945510	-0.745371
H	-4.327095	4.804721	-0.543110
H	-3.757275	3.630188	1.303354
C	-1.946118	2.710673	1.948477
H	-1.206081	3.407508	2.356608
H	-2.552717	2.331119	2.777842
C	-1.134771	1.540695	1.378047
N	-1.655494	0.901696	0.303540
O	-0.057849	1.234089	1.897554
H	-2.587390	1.145450	-0.012094
C	-0.972585	-0.208781	-0.346155
H	-0.807183	-1.012631	0.379114
C	0.434110	0.157419	-0.853099
N	0.685893	1.466912	-1.115155
O	1.267442	-0.738338	-1.023772
H	-0.001176	2.181249	-0.883457
C	2.014905	1.890150	-1.526301
H	2.326700	1.362171	-2.432432
H	1.987683	2.961594	-1.747448
C	3.138732	1.640754	-0.504306
N	2.770095	1.413751	0.781858
O	4.314587	1.649203	-0.884272
H	1.785742	1.373535	1.037055
C	3.754535	1.052602	1.788754
H	4.579417	1.771325	1.794210
H	3.275316	1.076624	2.771897
C	4.420641	-0.328487	1.625291
N	3.878265	-1.165783	0.695150
O	5.392000	-0.609789	2.325237
H	3.057526	-0.879741	0.168212
C	4.520920	-2.412077	0.325443
H	5.186699	-2.713517	1.139046
H	3.775984	-3.201106	0.181478
C	5.373636	-2.376667	-0.957857
N	5.579503	-1.155105	-1.523418
O	5.829283	-3.424976	-1.413118
H	5.218679	-0.287444	-1.139075
H	6.169051	-1.120877	-2.343414
C	-1.817303	-0.739196	-1.532155
C	-3.221510	-1.122714	-1.156973
H	-1.844084	0.026808	-2.317822
H	-1.275154	-1.598641	-1.942207
C	-4.378992	-0.524632	-1.613083
H	-4.498287	0.284344	-2.323538
N	-5.485373	-1.144040	-1.052226
H	-6.446920	-0.935791	-1.276481
C	-5.059281	-2.171468	-0.226011
C	-5.800719	-3.083687	0.541670
C	-3.635047	-2.185526	-0.265265
C	-2.940061	-3.156633	0.488154
H	-1.853651	-3.208531	0.467997
C	-3.670512	-4.066379	1.252089
H	-3.148664	-4.822412	1.834460
C	-5.086064	-4.028720	1.280210
H	-5.626655	-4.753623	1.885078
H	-6.888632	-3.062512	0.559432

-1651.5400051

W **Helix/trans**

H	-5.958146	0.363382	2.022190
C	-6.215316	-0.576459	1.525884
C	-4.974072	-1.137489	0.856275
N	-5.123732	-1.564760	-0.443234
O	-3.884867	-1.195257	1.430979
H	-6.537193	-1.280182	2.301599
H	-7.046263	-0.408999	0.833493
H	-6.062281	-1.603558	-0.819011
C	-4.097938	-2.382853	-1.088852
H	-3.865832	-3.270580	-0.490597
H	-4.479147	-2.717470	-2.059322
C	-2.748354	-1.690021	-1.326914
N	-2.751026	-0.334646	-1.343591
O	-1.735240	-2.373727	-1.489388
H	-3.625250	0.134556	-1.142157
C	-1.524462	0.462832	-1.402214
H	-1.003894	0.266270	-2.344628
C	-0.503745	0.077243	-0.312201
N	-0.998060	-0.375170	0.873181
O	0.703131	0.195018	-0.533561
H	-1.989553	-0.577637	0.979931
C	-0.091986	-0.775057	1.938225
H	0.568284	0.054179	2.210298
H	-0.684784	-1.042043	2.818687
C	0.846345	-1.953175	1.622948
N	0.530421	-2.740444	0.562903
O	1.842151	-2.136939	2.331190
H	-0.259604	-2.506252	-0.032646
C	1.434923	-3.787386	0.115794
H	1.702603	-4.443773	0.948916
H	0.926085	-4.389395	-0.642900
C	2.778004	-3.319161	-0.480138
N	2.898872	-1.988038	-0.748674
O	3.662109	-4.149680	-0.684903
H	2.117031	-1.359125	-0.582365
C	4.162651	-1.402953	-1.151980
H	4.794163	-2.191725	-1.570713
H	4.004049	-0.643438	-1.924088
C	4.978116	-0.723529	-0.034441
N	4.527229	-0.889648	1.239163
O	5.984677	-0.077146	-0.324047
H	3.702434	-1.430972	1.478341
H	5.072916	-0.472740	1.980393
C	-1.928089	1.961211	-1.337987
C	-0.783187	2.931695	-1.267676
H	-2.576320	2.105523	-0.462420
H	-2.541757	2.164479	-2.225725
C	-0.029861	3.402637	-2.322562
H	-0.110458	3.182585	-3.379788
N	0.952770	4.255491	-1.853397
H	1.646410	4.715353	-2.424366
C	0.851106	4.357915	-0.476984
C	1.612739	5.101691	0.437703
C	-0.236923	3.532056	-0.072368
C	-0.563237	3.457573	1.298445
H	-1.383684	2.830444	1.643451
C	0.187887	4.195601	2.213025
H	-0.050852	4.146214	3.273408
C	1.266413	5.008859	1.786870
H	1.838173	5.567631	2.524742
H	2.445780	5.722263	0.113542

-1651.5341456

W Hairpin/-gauche

H	5.556687	3.097614	0.601995
C	5.025371	3.374536	-0.314287
C	3.904606	2.376043	-0.547824
N	2.651244	2.879273	-0.606035
O	4.136303	1.162929	-0.677800
H	4.673301	4.406962	-0.228061
H	5.737201	3.304239	-1.143011
H	2.454641	3.870687	-0.507909
C	1.500369	2.030497	-0.831636
H	1.653482	1.391502	-1.706538
H	1.327313	1.355796	0.016835
C	0.260850	2.907442	-1.029252
N	-0.850221	2.216344	-1.384071
O	0.291329	4.131351	-0.856091
H	-0.780875	1.206955	-1.548707
C	-2.121021	2.886450	-1.584349
H	-2.251392	3.660398	-0.819656
H	-2.164877	3.388677	-2.563170
C	-3.239810	1.845861	-1.553858
N	-4.308371	2.134059	-0.747860
O	-3.173436	0.796801	-2.196483
H	-4.334221	3.029470	-0.278029
C	-5.511244	1.305666	-0.763744
H	-6.296740	1.815249	-0.197430
H	-5.869583	1.164784	-1.788536
C	-5.368037	-0.114535	-0.184864
N	-4.241222	-0.359614	0.546777
O	-6.251436	-0.940187	-0.388630
H	-3.568937	0.389243	0.647961
C	-3.887279	-1.682183	1.029792
H	-3.637337	-1.660473	2.095018
H	-4.762412	-2.326123	0.893705
C	-2.691077	-2.334417	0.314724
N	-2.497130	-1.992242	-0.989766
O	-1.986690	-3.154178	0.910213
H	-2.974121	-1.183072	-1.379808
C	-1.340834	-2.501523	-1.710245
H	-1.234157	-3.570921	-1.507187
H	-1.506662	-2.351511	-2.781796
C	-0.074413	-1.723511	-1.318348
N	0.968172	-2.454138	-0.868674
O	-0.053259	-0.488149	-1.403742
H	0.925554	-3.466947	-0.892405
C	2.207453	-1.869368	-0.378271
H	2.162827	-0.796365	-0.564496
C	3.395073	-2.512391	-1.121422
N	4.426328	-1.666750	-1.361054
O	3.371424	-3.714137	-1.407392
H	4.323159	-0.679787	-1.122108
C	5.662856	-2.107348	-1.988403
H	5.617996	-3.191248	-2.113057
H	5.792935	-1.641355	-2.973360
H	6.525958	-1.849638	-1.362984
C	2.387547	-2.121761	1.137156
H	2.467635	-3.195481	1.333617
H	3.320657	-1.675552	1.481107
C	1.247220	-1.512587	1.897789
C	0.050211	-2.167474	2.211304
C	1.221601	-0.112105	2.434441
H	-0.092047	-3.114376	1.922965
N	-0.758951	-1.156183	2.967972
H	-1.733850	-1.411643	3.327433
C	2.061360	0.533787	2.284351
C	-0.023420	0.012572	3.062436
H	2.919395	0.180669	1.751449
C	2.037449	1.840903	2.785226
H	2.877209	2.486795	2.635136
C	0.914787	2.302926	3.482481
H	0.896512	3.301936	3.865293
C	-0.183964	1.457834	3.678860
H	-1.041999	1.810951	4.211762

-1898.6769765

W Hairpin/trans

H	4.976453	3.476177	-2.018735
C	4.061965	3.493029	-2.620559
C	3.088916	2.475385	-2.049995
N	1.905855	2.957271	-1.606152
O	3.372159	1.269192	-1.997013
H	3.661817	4.511292	-2.637378
H	4.329476	3.192445	-3.638450
H	1.667456	3.943808	-1.642071
C	0.903739	2.098993	-1.012926
H	0.590855	1.314636	-1.712900
H	1.298244	1.596895	-0.121108
C	-0.309059	2.950195	-0.627618
N	-1.320621	2.241974	-0.064487
O	-0.332270	4.170929	-0.815440
H	-1.248106	1.220337	-0.019136
C	-2.571986	2.869484	0.283647
H	-2.634458	3.078923	1.363061
H	-2.625903	3.835117	-0.234770
C	-3.762442	2.005141	-0.142369
N	-4.893754	2.166335	0.618487
O	-3.714662	1.205693	-1.075295
H	-4.901284	2.892805	1.323076
C	-6.164066	1.575639	0.205363
H	-6.966129	2.005309	0.813513
H	-6.375442	1.801214	-0.844763
C	-6.262705	0.043244	0.321747
N	-5.308768	-0.564659	1.087042
O	-7.174900	-0.548435	-0.245304
H	-4.596466	0.019264	1.504943
C	-5.169479	-2.007226	1.173170
H	-5.119148	-2.339418	2.215073
H	-6.059683	-2.451557	0.716986
C	-3.931624	-2.592172	0.470143
N	-3.368705	-1.823954	-0.506900
O	-3.524824	-3.711320	0.779293
H	-3.700568	-0.879203	-0.679146
C	-2.180412	-2.261653	-1.199362
H	-2.137191	-3.353997	-1.153401
H	-2.238318	-1.960277	-2.253088
C	-0.885244	-1.657064	-0.628024
N	0.238300	-2.341113	-0.924573
O	-0.876964	-0.585156	0.000126
H	0.196479	-3.253239	-1.370005
C	1.578292	-1.885914	-0.557175
H	1.582513	-0.794347	-0.568868
C	2.533358	-2.466992	-1.613636
N	3.428030	-1.603008	-2.139493
O	2.443217	-3.666387	-1.912214
H	3.361567	-0.607826	-1.922297
C	4.402809	-2.013363	-3.138583
H	4.433022	-3.104443	-3.169212
H	4.131784	-1.640199	-4.134761
H	5.393502	-1.624043	-2.878973
C	1.960712	-2.388404	0.866148
H	2.137401	-3.468078	0.808110
H	1.083947	-2.233272	1.505864
C	3.143157	-1.679156	1.459740
C	4.454058	-2.103544	1.486820
C	3.120257	-0.388531	2.110674
H	4.884729	-3.020127	1.103792
N	5.248051	-1.150791	2.108760
H	6.239892	-1.236075	2.272268
C	2.092125	0.531516	2.408376
C	4.456207	-0.087703	2.506347
H	1.062422	0.317782	2.125496
C	2.417735	1.710127	3.082034
H	1.636932	2.428739	3.323617
C	3.753016	1.990615	3.462675
H	3.975137	2.917767	3.987198
C	4.789512	1.096586	3.182112
H	5.813258	1.313683	3.481544

-1898.9192042

Y **Helix/-gauche**

H	-3.756910	3.835121	-2.270592
C	-3.825540	4.187295	-1.237619
C	-2.753639	3.501395	-0.410055
N	-3.159522	2.976656	0.794460
O	-1.585763	3.405225	-0.793839
H	-3.621952	5.263868	-1.243436
H	-4.839647	4.021073	-0.860771
H	-4.097304	3.185844	1.110780
C	-2.181545	2.540708	1.788327
H	-1.488682	3.348569	2.047206
H	-2.716273	2.244402	2.697152
C	-1.295562	1.366380	1.353119
N	-1.796338	0.545621	0.398512
O	-0.182890	1.215754	1.865358
H	-2.745752	0.694690	0.078782
C	-1.038198	-0.579314	-0.135362
H	-0.770814	-1.261541	0.679350
C	0.311785	-0.164605	-0.751703
N	0.434373	1.114565	-1.192055
O	1.215741	-1.001386	-0.842291
H	-0.306982	1.792114	-1.026334
C	1.703401	1.590801	-1.718946
H	2.027883	0.976285	-2.564013
H	1.570539	2.617401	-2.074046
C	2.881867	1.575244	-0.728566
N	2.582011	1.500928	0.593149
O	4.037783	1.624770	-1.162113
H	1.615310	1.415541	0.898897
C	3.631215	1.364957	1.590177
H	4.395270	2.135122	1.448544
H	3.192021	1.499269	2.583038
C	4.399409	0.028293	1.594262
N	3.898906	-0.971098	0.812501
O	5.410405	-0.076472	2.286626
H	3.045096	-0.824544	0.281064
C	4.629182	-2.204486	0.592367
H	5.340050	-2.338596	1.412593
H	3.946231	-3.059942	0.587504
C	5.438358	-2.284075	-0.716860
N	5.524441	-1.142525	-1.454721
O	5.966276	-3.347152	-1.040559
H	5.104443	-0.260180	-1.179943
H	6.085769	-1.178182	-2.294240
C	-1.871310	-1.341275	-1.193240
C	-3.201583	-1.843667	-0.667847
H	-2.034980	-0.683379	-2.056090
H	-1.247682	-2.172757	-1.538002
C	-4.413826	-1.277744	-1.100718
H	-4.400525	-0.489606	-1.853716
C	-5.648102	-1.722290	-0.611974
H	-6.583446	-1.290400	-0.958905
C	-5.685879	-2.758787	0.332562
C	-4.485906	-3.343152	0.773687
H	-4.508899	-4.157457	1.497954
C	-3.262258	-2.885780	0.273408
H	-2.343096	-3.357328	0.618252
O	-6.915679	-3.161358	0.782607
H	-6.804971	-3.898846	1.399609

-1595.1908866

Y **Helix/trans**

H	-6.042564	0.461834	2.394934
C	-6.395558	-0.142200	1.554502
C	-5.201613	-0.583273	0.727799
N	-5.306758	-0.412410	-0.633699
O	-4.183865	-1.054686	1.239633
H	-6.876088	-1.034729	1.970405
H	-7.136943	0.424429	0.982416
H	-6.207275	-0.137898	-1.004318
C	-4.364627	-1.059308	-1.545681
H	-4.324894	-2.140467	-1.374561
H	-4.702645	-0.889610	-2.573409
C	-2.911160	-0.577182	-1.442928
N	-2.710341	0.653096	-0.908805
O	-1.998352	-1.304772	-1.840361
H	-3.518731	1.161731	-0.573223
C	-1.374081	1.194540	-0.657216
H	-0.825912	1.266701	-1.601914
C	-0.517627	0.268990	0.230338
N	-1.177699	-0.534407	1.109987
O	0.712099	0.291105	0.138382
H	-2.192419	-0.607543	1.087468
C	-0.442784	-1.472125	1.943972
H	0.285427	-0.946730	2.569477
H	-1.152544	-1.980884	2.603578
C	0.371938	-2.547120	1.202149
N	0.074199	-2.773379	-0.102183
O	1.260369	-3.152500	1.811556
H	-0.628680	-2.209772	-0.573970
C	0.890666	-3.667918	-0.907849
H	0.988436	-4.641576	-0.418976
H	0.397367	-3.818753	-1.872621
C	2.336434	-3.206045	-1.184606
N	2.640143	-1.915599	-0.866581
O	3.135062	-4.005744	-1.669942
H	1.922554	-1.296992	-0.497114
C	4.000368	-1.415200	-0.919017
H	4.582294	-2.057985	-1.585778
H	4.019535	-0.397171	-1.320585
C	4.751766	-1.369695	0.425637
N	4.145338	-1.971422	1.486495
O	5.845389	-0.809665	0.493899
H	3.254309	-2.454286	1.426313
H	4.654830	-1.988445	2.358963
C	-1.524088	2.610873	-0.037299
C	-0.248987	3.428003	-0.014810
H	-1.933588	2.507207	0.976151
H	-2.276238	3.140449	-0.638226
C	0.116608	4.204582	-1.129013
H	-0.530558	4.228590	-2.005556
C	1.297367	4.950816	-1.143878
H	1.580165	5.547769	-2.007166
C	2.142555	4.931294	-0.022564
C	1.789368	4.172161	1.103971
H	2.441515	4.153618	1.976961
C	0.603148	3.429518	1.099101
H	0.351294	2.835996	1.976147
O	3.289948	5.677317	-0.083499
H	3.813818	5.520646	0.715137

-1595.1856957

Y Hairpin/-gauche

H	5.860648	3.177274	1.023991
C	5.301668	3.518514	0.146764
C	4.161098	2.549274	-0.113927
N	2.915854	3.074362	-0.131781
O	4.370830	1.339373	-0.301490
H	4.966063	4.547083	0.310098
H	5.984607	3.495051	-0.708676
H	2.733508	4.061068	0.026881
C	1.746626	2.257547	-0.380140
H	1.830469	1.729214	-1.335937
H	1.627548	1.487118	0.390595
C	0.503529	3.152030	-0.384453
N	-0.639150	2.495409	-0.687713
O	0.563911	4.358577	-0.117305
H	-0.597557	1.496138	-0.913459
C	-1.917581	3.174147	-0.695589
H	-2.128270	3.620208	0.286135
H	-1.919314	3.998930	-1.423468
C	-3.009148	2.184135	-1.101179
N	-4.190596	2.290959	-0.415717
O	-2.838648	1.333461	-1.974200
H	-4.293369	3.035411	0.261349
C	-5.377077	1.554597	-0.843362
H	-6.243696	1.935644	-0.294486
H	-5.560570	1.704778	-1.912150
C	-5.342876	0.027149	-0.648681
N	-4.358650	-0.462070	0.160680
O	-6.188796	-0.666483	-1.203640
H	-3.708845	0.193608	0.574382
C	-4.122108	-1.883254	0.338964
H	-4.070491	-2.144272	1.400533
H	-4.969949	-2.415535	-0.104193
C	-2.830097	-2.418858	-0.302846
N	-2.349539	-1.726838	-1.375324
O	-2.309127	-3.446835	0.132941
H	-2.728521	-0.810976	-1.601279
C	-1.083549	-2.099248	-1.977717
H	-0.987989	-3.187944	-1.950308
H	-1.076085	-1.765647	-3.021802
C	0.098041	-1.416208	-1.269618
N	1.173586	-2.189970	-1.015708
O	0.043930	-0.213936	-0.975979
H	1.203991	-3.152984	-1.335912
C	2.398492	-1.698639	-0.396516
H	2.381737	-0.608654	-0.434260
C	3.588331	-2.254687	-1.195668
N	4.650582	-1.417361	-1.277135
O	3.541043	-3.396987	-1.666750
H	4.559066	-0.467709	-0.915371
C	5.892866	-1.798630	-1.930689
H	5.832097	-2.856499	-2.194537
H	6.054786	-1.214135	-2.845378
H	6.745432	-1.640730	-1.259415
C	2.523353	-2.160390	1.085805
H	3.534097	-1.902644	1.423482
C	1.498974	-1.533820	2.008201
H	2.446726	-3.254071	1.101958
C	0.215638	-2.087899	2.169858
H	-0.061520	-2.995309	1.637065
C	-0.734945	-1.491883	3.004143
H	-1.718904	-1.936174	3.133509
C	-0.413698	-0.317184	3.700686
C	0.862915	0.248320	3.561486
H	1.124157	1.154386	4.109282
C	1.804280	-0.363189	2.721767
H	2.794337	0.081445	2.626356
O	-1.384593	0.235637	4.504489
H	-1.005605	0.991095	4.975647

-1842.570198

Y Hairpin/trans

H	-4.629367	4.253104	0.778746
C	-3.715403	4.388124	1.365973
C	-2.786101	3.218618	1.091413
N	-1.566826	3.521695	0.588450
O	-3.131764	2.049202	1.314901
H	-3.271839	5.358766	1.124388
H	-3.996873	4.372997	2.423770
H	-1.260363	4.476627	0.427964
C	-0.588684	2.498160	0.293890
H	-0.380371	1.884928	1.178864
H	-0.951605	1.812683	-0.481688
C	0.704407	3.168701	-0.179463
N	1.688972	2.292873	-0.497689
O	0.808392	4.397215	-0.258238
H	1.530326	1.291379	-0.342835
C	3.002047	2.747464	-0.887749
H	3.105316	2.801042	-1.982930
H	3.146311	3.765380	-0.503661
C	4.086129	1.835420	-0.308015
N	5.228631	1.740942	-1.061558
O	3.944578	1.207646	0.740155
H	5.320273	2.333364	-1.876944
C	6.418742	1.075254	-0.538004
H	7.264417	1.298536	-1.195783
H	6.661221	1.442920	0.464153
C	6.328028	-0.456124	-0.399937
N	5.303086	-1.062059	-1.069181
O	7.163665	-1.051968	0.270943
H	4.665906	-0.473691	-1.589452
C	4.986540	-2.470860	-0.916053
H	4.885040	-2.962254	-1.888987
H	5.820196	-2.939447	-0.383666
C	3.694439	-2.775429	-0.137022
N	3.240191	-1.793003	0.693569
O	3.151047	-3.872425	-0.258642
H	3.685610	-0.879929	0.709771
C	2.020553	-1.966883	1.446466
H	1.866534	-3.037152	1.613522
H	2.121539	-1.473547	2.421498
C	0.786449	-1.355455	0.758203
N	-0.393865	-1.884937	1.138964
O	0.876054	-0.403219	-0.033954
H	-0.428751	-2.713522	1.725683
C	-1.693787	-1.402287	0.673835
H	-1.617173	-0.328709	0.489124
C	-2.693674	-1.708865	1.801910
N	-3.500511	-0.688351	2.162040
O	-2.709217	-2.842631	2.301927
H	-3.364881	0.231898	1.742830
C	-4.523933	-0.830044	3.185901
H	-4.522168	-1.864290	3.535801
H	-4.321183	-0.164070	4.033724
H	-5.512153	-0.585539	2.778280
C	-2.100691	-2.127232	-0.640139
H	-2.179410	-3.196553	-0.414275
C	-3.386796	-1.616246	-1.254108
H	-1.269657	-1.995753	-1.342455
C	-3.378138	-0.532899	-2.151627
H	-2.429333	-0.071153	-2.423508
C	-4.557768	-0.037997	-2.715620
H	-4.546711	0.796550	-3.412379
C	-5.785217	-0.633788	-2.386711
C	-5.817477	-1.718842	-1.497277
H	-6.767552	-2.190406	-1.244447
C	-4.625127	-2.198745	-0.940131
H	-4.662007	-3.040541	-0.250385
O	-6.917944	-0.117757	-2.968216
H	-7.684745	-0.622487	-2.662314

-1842.5695364

Atp Helix

H	5.476031	2.459475	-1.022258
C	5.602046	1.623427	-1.716095
C	4.365666	0.747689	-1.660370
N	4.576361	-0.601082	-1.486801
O	3.224670	1.209202	-1.744454
H	5.682152	2.042911	-2.724927
H	6.526739	1.088371	-1.479025
H	5.527837	-0.941481	-1.537019
C	3.496120	-1.561150	-1.700954
H	3.050909	-1.446065	-2.695135
H	3.907547	-2.573280	-1.626956
C	2.326592	-1.458962	-0.715152
N	2.589759	-0.863256	0.479696
O	1.219155	-1.905453	-1.013633
H	3.501767	-0.445194	0.617240
C	1.522728	-0.595382	1.429397
H	1.023734	-1.526701	1.715189
C	0.381127	0.310737	0.886611
N	0.688735	1.125414	-0.150822
O	-0.737795	0.225452	1.390730
H	1.612229	1.108576	-0.580884
C	-0.347780	1.931156	-0.779924
H	-0.835915	2.575752	-0.042930
H	0.120306	2.569192	-1.535360
C	-1.487101	1.146845	-1.455990
N	-1.270270	-0.166625	-1.723041
O	-2.542208	1.729335	-1.722717
H	-0.400289	-0.611526	-1.445795
C	-2.327879	-1.005024	-2.265921
H	-2.765040	-0.540236	-3.154501
H	-1.896625	-1.965987	-2.561186
C	-3.519264	-1.293007	-1.330357
N	-3.379287	-0.926528	-0.023805
O	-4.523311	-1.829547	-1.793889
H	-2.510722	-0.508977	0.296121
C	-4.490529	-0.983365	0.907650
H	-5.221968	-1.704463	0.532313
H	-4.149262	-1.322260	1.890856
C	-5.246964	0.339622	1.138693
N	-4.902023	1.391002	0.343979
O	-6.112245	0.398482	2.010407
H	-4.209974	1.334761	-0.396050
H	-5.424044	2.248141	0.462941
C	2.150872	-0.010826	2.700119
F	1.236396	0.301747	3.619029
F	2.866034	1.110991	2.416313
F	3.017762	-0.893162	3.250783

-1586.6774131

Atp Hairpin

H	5.646535	3.663650	0.730068
C	4.917883	3.910195	-0.048907
C	3.819945	2.862297	-0.035132
N	2.561926	3.304355	0.188348
O	4.067749	1.659096	-0.216717
H	4.546601	4.926283	0.114489
H	5.435885	3.865449	-1.012254
H	2.335595	4.288978	0.293833
C	1.434399	2.398956	0.205280
H	1.407155	1.794470	-0.708752
H	1.497325	1.697144	1.045814
C	0.138854	3.208052	0.314071
N	-0.976669	2.440212	0.324036
O	0.141492	4.441234	0.383934
H	-0.881311	1.427791	0.208704
C	-2.298591	3.021600	0.344283
H	-2.616382	3.273750	1.367661
H	-2.292124	3.961004	-0.224532
C	-3.299166	2.058008	-0.297103
N	-4.577397	2.131195	0.193141
O	-2.982635	1.253092	-1.171646
H	-4.797281	2.855695	0.864698
C	-5.673843	1.429742	-0.470153
H	-6.620811	1.781752	-0.049535
H	-5.679297	1.643020	-1.543923
C	-5.662210	-0.106144	-0.359152
N	-4.832608	-0.639781	0.585672
O	-6.390474	-0.767126	-1.091289
H	-4.276962	-0.004571	1.142694
C	-4.628870	-2.069261	0.732942
H	-4.752818	-2.381155	1.775081
H	-5.394395	-2.574552	0.135995
C	-3.258348	-2.604513	0.283084
N	-2.522181	-1.798782	-0.539036
O	-2.896366	-3.721608	0.647723
H	-2.850408	-0.868926	-0.784996
C	-1.220819	-2.217086	-1.004818
H	-1.124248	-3.289025	-0.808637
H	-1.134470	-2.053699	-2.087980
C	-0.068000	-1.445437	-0.345179
N	1.130236	-2.085523	-0.370301
O	-0.211693	-0.311761	0.123035
H	1.240760	-2.979820	-0.843105
C	2.378501	-1.453823	-0.001270
H	2.264661	-0.367813	-0.002975
C	3.459984	-1.906774	-1.016947
N	4.458289	-1.020799	-1.205709
O	3.343999	-3.002729	-1.572267
H	4.394282	-0.099661	-0.769198
C	5.577519	-1.293835	-2.095828
H	5.505920	-2.328007	-2.437648
H	5.556427	-0.628011	-2.967525
H	6.526701	-1.151034	-1.566860
C	2.778792	-1.842776	1.435745
F	1.831945	-1.460413	2.320246
F	3.933485	-1.241003	1.796099
F	2.947761	-3.172544	1.575673

-1834.0618969

Atb Helix/-gauche

H	5.049734	-2.182096	-2.487286
C	5.173945	-2.683059	-1.523239
C	3.975976	-2.370932	-0.646365
N	4.249825	-1.976676	0.642079
O	2.816554	-2.449594	-1.060730
H	5.191136	-3.761994	-1.713218
H	6.128569	-2.386671	-1.077582
H	5.207338	-2.037928	0.962855
C	3.192654	-1.921977	1.648852
H	2.688274	-2.888768	1.752105
H	3.642707	-1.666302	2.614097
C	2.078886	-0.908080	1.363477
N	2.399075	0.145409	0.568052
O	0.956529	-1.071480	1.846368
H	3.350346	0.232535	0.234207
C	1.397733	1.137632	0.198125
H	0.945530	1.550895	1.105915
C	0.201885	0.547617	-0.587943
N	0.382653	-0.650200	-1.196939
O	-0.852408	1.186900	-0.634342
H	1.250738	-1.170900	-1.081569
C	-0.730403	-1.306422	-1.866011
H	-1.163462	-0.651970	-2.628184
H	-0.357386	-2.208280	-2.360632
C	-1.906702	-1.708353	-0.957828
N	-1.676610	-1.748283	0.379425
O	-3.001744	-1.965588	-1.467432
H	-0.763446	-1.504274	0.754226
C	-2.757030	-2.013918	1.316272
H	-3.292541	-2.925918	1.036156
H	-2.328845	-2.162173	2.311975
C	-3.842539	-0.924773	1.429273
N	-3.579315	0.271439	0.827996
O	-4.876864	-1.175758	2.044197
H	-2.692896	0.416812	0.354339
C	-4.588974	1.306971	0.713743
H	-5.345232	1.140130	1.486010
H	-4.146469	2.294795	0.876473
C	-5.334405	1.377542	-0.633487
N	-5.103609	0.366031	-1.516138
O	-6.093005	2.319204	-0.858145
H	-4.498948	-0.425660	-1.321550
H	-5.625154	0.383066	-2.381603
C	1.995810	2.282469	-0.635441
H	1.185359	2.974801	-0.879835
H	2.419126	1.912897	-1.576459
C	3.081923	3.079698	0.066492
F	3.413573	4.175205	-0.646119
F	4.229340	2.349482	0.215378
F	2.715569	3.486092	1.298894

-1626.0082757

Atb Helix/trans

C	-5.829838	-1.817744	1.502778
C	-4.539480	-1.799900	0.705111
N	-4.669548	-1.733885	-0.664097
C	-3.536481	-2.027664	-1.540442
C	-2.364304	-1.041512	-1.460157
N	-2.636270	0.195649	-0.967764
C	-1.579994	1.176927	-0.717902
C	-2.261674	2.448872	-0.174985
H	-2.682329	2.275251	0.822100
O	-3.430318	-1.826024	1.243099
O	-1.244453	-1.388028	-1.837239
C	-0.460260	0.640360	0.208380
O	0.684206	1.078335	0.089225
N	-0.809634	-0.304341	1.121613
C	0.199089	-0.887948	1.994240
C	1.330898	-1.666352	1.299495
O	2.362076	-1.915875	1.931569
N	1.135645	-2.041967	0.009886
C	2.199866	-2.675023	-0.753152
C	3.420514	-1.792812	-1.084900
O	4.427721	-2.321854	-1.551162
N	3.301744	-0.457702	-0.833398
C	4.436074	0.440986	-0.937312
C	5.176132	0.749951	0.379106
O	6.071273	1.593249	0.387881
N	4.782840	0.054371	1.482416
C	-1.387414	3.693861	-0.082704
H	-3.082673	2.715809	-0.850583
H	-6.714318	-1.575605	0.905289
H	-5.956368	-2.818380	1.931183
H	-5.745028	-1.113364	2.334725
H	-5.598924	-1.837285	-1.050941
H	-3.105271	-3.009703	-1.317534
H	-3.891074	-2.040212	-2.576427
H	-3.571312	0.366796	-0.619839
H	-1.070269	1.408717	-1.658136
H	-1.735387	-0.727687	1.108327
H	0.700739	-0.108489	2.575623
H	-0.297431	-1.567462	2.693566
H	0.295770	-1.758397	-0.486565
H	2.603839	-3.531381	-0.205466
H	1.785195	-3.039007	-1.697772
H	2.428207	-0.082226	-0.475283
H	5.171157	-0.000912	-1.615699
H	4.122962	1.404385	-1.351745
H	4.066346	-0.664236	1.469615
H	5.292764	0.223285	2.338400
F	-2.180311	4.773576	0.146742
F	-0.716221	3.928844	-1.229268
F	-0.496006	3.640335	0.921886

-1626.0001958

Atb **Hairpin/-gauche**

H	5.827614	3.472754	0.635258
C	5.094862	3.784013	-0.116245
C	3.976718	2.757650	-0.158618
N	2.731716	3.208736	0.106798
O	4.203533	1.564992	-0.422781
H	4.745059	4.793526	0.119381
H	5.600804	3.794415	-1.086956
H	2.529784	4.187769	0.288587
C	1.580851	2.331475	0.090004
H	1.513852	1.793604	-0.862806
H	1.646136	1.572814	0.878299
C	0.314473	3.168833	0.291658
N	-0.825857	2.439851	0.276460
O	0.360162	4.393462	0.451975
H	-0.769703	1.432811	0.098257
C	-2.123159	3.062587	0.397455
H	-2.386167	3.252985	1.449458
H	-2.106470	4.039119	-0.104728
C	-3.187918	2.184470	-0.262236
N	-4.437436	2.262013	0.297659
O	-2.946451	1.440705	-1.211644
H	-4.596397	2.937678	1.034085
C	-5.591065	1.657186	-0.363588
H	-6.502813	2.012123	0.126681
H	-5.634731	1.953153	-1.416666
C	-5.639649	0.117937	-0.370896
N	-4.791459	-0.519266	0.489200
O	-6.427761	-0.454918	-1.115442
H	-4.183003	0.049328	1.062879
C	-4.634748	-1.962232	0.511096
H	-4.726415	-2.354522	1.529140
H	-5.442558	-2.390515	-0.090283
C	-3.303582	-2.501804	-0.040799
N	-2.577032	-1.654893	-0.827883
O	-2.965022	-3.655864	0.216629
H	-2.880169	-0.696647	-0.978439
C	-1.301213	-2.063876	-1.368934
H	-1.244359	-3.154462	-1.308626
H	-1.230201	-1.768834	-2.424676
C	-0.109122	-1.417371	-0.645654
N	1.043075	-2.128404	-0.690896
O	-0.193310	-0.307078	-0.107172
H	1.093607	-2.994060	-1.221813
C	2.336114	-1.603095	-0.274160
H	2.253251	-0.520519	-0.164158
C	3.354237	-1.971552	-1.376388
N	4.333540	-1.062661	-1.574855
O	3.245620	-3.053653	-1.964765
H	4.275557	-0.153880	-1.110405
C	5.406897	-1.278244	-2.535122
H	5.367967	-2.315387	-2.873863
H	5.297439	-0.617722	-3.404483
H	6.378529	-1.082915	-2.067453
C	2.843426	-2.218100	1.052198
H	3.895977	-1.949949	1.192165
H	2.775198	-3.309808	1.012732
C	2.117857	-1.749252	2.300582
F	2.711528	-2.259465	3.408485
F	0.821859	-2.123166	2.340327
F	2.149096	-0.396634	2.428582

-1873.3886525

Atb **Hairpin/trans**

H	5.340843	3.943830	0.007463
C	4.516678	4.119219	-0.691654
C	3.495859	3.007642	-0.525898
N	2.252067	3.380640	-0.147207
O	3.792403	1.819591	-0.724678
H	4.102018	5.116179	-0.514868
H	4.927193	4.072184	-1.705222
H	1.985356	4.352484	-0.021029
C	1.191310	2.414592	0.036078
H	1.077227	1.787485	-0.855841
H	1.409280	1.738617	0.872146
C	-0.120358	3.157659	0.306297
N	-1.186313	2.336056	0.471666
O	-0.171573	4.390080	0.365781
H	-1.056628	1.327544	0.346927
C	-2.519041	2.857002	0.664429
H	-2.760767	2.969555	1.732838
H	-2.572827	3.858661	0.218922
C	-3.552462	1.953269	-0.012762
N	-4.775394	1.904111	0.605837
O	-3.306635	1.293698	-1.021911
H	-4.942843	2.515343	1.394798
C	-5.914729	1.247804	-0.030676
H	-6.823644	1.509432	0.519729
H	-6.033088	1.590487	-1.063568
C	-5.848240	-0.288993	-0.115506
N	-4.910246	-0.895810	0.670562
O	-6.624843	-0.886887	-0.852003
H	-4.313778	-0.304396	1.233712
C	-4.609746	-2.313951	0.587017
H	-4.615091	-2.779741	1.577561
H	-5.395520	-2.782127	-0.014338
C	-3.251747	-2.659114	-0.049591
N	-2.716552	-1.720414	-0.883494
O	-2.725461	-3.748152	0.174763
H	-3.137300	-0.798914	-0.964653
C	-1.422871	-1.929583	-1.488373
H	-1.277323	-3.002493	-1.646460
H	-1.392775	-1.426736	-2.462949
C	-0.266205	-1.357370	-0.650433
N	0.940876	-1.920964	-0.876568
O	-0.421969	-0.405401	0.129121
H	1.035991	-2.762160	-1.438766
C	2.172984	-1.455192	-0.242628
H	2.123911	-0.369477	-0.141394
C	3.318843	-1.884759	-1.181987
N	4.215999	-0.923758	-1.479692
O	3.338365	-3.047750	-1.604952
H	4.069771	0.025489	-1.132869
C	5.379266	-1.181461	-2.315393
H	5.414205	-2.248788	-2.541916
H	5.318822	-0.618397	-3.255072
H	6.296354	-0.890688	-1.790337
C	2.301805	-2.101382	1.158176
H	1.349518	-1.983229	1.683817
H	2.517382	-3.169703	1.058040
C	3.363746	-1.488581	2.050009
F	3.325688	-2.040773	3.286732
F	3.183760	-0.151048	2.209778
F	4.619200	-1.662810	1.577269

-1873.3887548

Hfl Helix/-gauche

H	-3.542135	4.040072	-2.354453
C	-3.567932	4.490950	-1.358899
C	-2.491137	3.855688	-0.500860
N	-2.855370	3.505149	0.777415
O	-1.348576	3.648151	-0.918745
H	-3.336278	5.556044	-1.472458
H	-4.572922	4.391248	-0.937340
H	-3.770548	3.785994	1.104702
C	-1.845918	3.158886	1.774884
H	-1.099599	3.953855	1.878148
H	-2.341432	3.029872	2.742975
C	-1.046560	1.882350	1.484009
N	-1.626228	0.969994	0.656968
O	0.060211	1.717802	1.999343
H	-2.497356	1.218987	0.203967
C	-0.901114	-0.220033	0.225149
H	-0.528925	-0.742395	1.113592
C	0.380153	0.076409	-0.588820
N	0.518481	1.314405	-1.124285
O	1.225574	-0.815349	-0.705957
H	-0.181427	2.038388	-0.970225
C	1.756964	1.687348	-1.790829
H	1.985701	0.991442	-2.603288
H	1.635455	2.686914	-2.218998
C	3.008538	1.695871	-0.894343
N	2.810809	1.724881	0.449490
O	4.127221	1.668521	-1.415392
H	1.869117	1.716213	0.830931
C	3.931077	1.623988	1.371742
H	4.700982	2.359109	1.119434
H	3.573832	1.838303	2.383217
C	4.662297	0.267104	1.410091
N	4.070678	-0.771286	0.752193
O	5.725631	0.181775	2.020963
H	3.180763	-0.635569	0.281929
C	4.744499	-2.043832	0.573569
H	5.515622	-2.138673	1.343235
H	4.038754	-2.872013	0.692352
C	5.440980	-2.253647	-0.785165
N	5.499412	-1.176411	-1.617084
O	5.907747	-3.356751	-1.063681
H	5.140087	-0.258834	-1.375311
H	5.993914	-1.295306	-2.490343
C	-1.809767	-1.167801	-0.573592
H	-1.187649	-1.996520	-0.917605
H	-2.175485	-0.662083	-1.473369
C	-3.009026	-1.709919	0.254252
H	-2.999986	-1.284878	1.263800
C	-4.347143	-1.281737	-0.358501
C	-2.905202	-3.229977	0.448986
F	-5.405375	-1.642393	0.384883
F	-4.530851	-1.755148	-1.603078
F	-4.381991	0.084974	-0.447615
F	-3.896488	-3.714920	1.221263
F	-1.736822	-3.518642	1.064306
F	-2.927547	-3.901691	-0.718654

-2002.4544813

Hfl Helix/trans

C	-4.461994	-4.289722	1.518620
C	-3.275492	-3.862210	0.676036
N	-3.490957	-3.767839	-0.679796
C	-2.367194	-3.666184	-1.609459
C	-1.523704	-2.392074	-1.479641
N	-2.129844	-1.307092	-0.928697
C	-1.396174	-0.071056	-0.676365
C	-2.381488	0.986970	-0.134180
H	-2.729483	0.666034	0.853597
O	-2.177085	-3.595169	1.169661
O	-0.355837	-2.384399	-1.871815
C	-0.196958	-0.251929	0.278728
O	0.708660	0.592610	0.267961
N	-0.194211	-1.330160	1.098409
C	0.948712	-1.596285	1.959013
C	2.272235	-1.889684	1.230518
O	3.334657	-1.807974	1.854045
N	2.201398	-2.221709	-0.084294
C	3.409121	-2.418713	-0.870810
C	4.263845	-1.162484	-1.132338
O	5.387725	-1.295441	-1.611741
N	3.705052	0.039723	-0.809311
C	4.471937	1.271017	-0.840323
C	5.014662	1.762265	0.516010
O	5.533060	2.874587	0.594612
N	4.885307	0.912483	1.573047
C	-1.778226	2.408900	-0.016368
H	-3.259486	1.021134	-0.788943
H	-5.406825	-4.299504	0.966343
H	-4.266673	-5.296780	1.903738
H	-4.547792	-3.620647	2.379253
H	-4.369606	-4.116214	-1.041219
H	-1.664421	-4.496043	-1.478279
H	-2.756223	-3.707862	-2.632418
H	-3.074941	-1.412211	-0.581354
H	-0.953894	0.275424	-1.614301
H	-0.923931	-2.038338	1.036145
H	1.155419	-0.737558	2.604299
H	0.712853	-2.454087	2.595923
H	1.305356	-2.244365	-0.563448
H	4.079627	-3.124456	-0.371648
H	3.130872	-2.840559	-1.841011
H	2.761743	0.075048	-0.434961
H	5.336186	1.121975	-1.493696
H	3.871665	2.089497	-1.250163
H	4.487256	-0.018435	1.503008
H	5.281265	1.210886	2.453714
C	-2.344114	3.120682	1.219449
H	-0.695089	2.341624	0.136397
C	-1.993160	3.214995	-1.304581
F	-1.906782	4.384489	1.339281
F	-1.965900	2.452746	2.336296
F	-3.697562	3.153547	1.221113
F	-1.333020	4.383550	-1.296057
F	-3.299764	3.481034	-1.534170
F	-1.545036	2.507629	-2.372476

-2002.4548917

Hfl Hairpin/-gauche

H	-4.881921	4.658147	-0.957981
C	-4.059511	4.999018	-0.320816
C	-3.124238	3.829713	-0.069159
N	-1.843303	3.982908	-0.472875
O	-3.521533	2.788479	0.477583
H	-3.565935	5.851975	-0.796070
H	-4.490844	5.316989	0.633725
H	-1.501714	4.838058	-0.901697
C	-0.856752	2.939612	-0.296047
H	-0.721541	2.701106	0.766898
H	-1.167458	2.015044	-0.793083
C	0.478230	3.414802	-0.875620
N	1.450498	2.470114	-0.850489
O	0.626845	4.558798	-1.316312
H	1.245154	1.556419	-0.435707
C	2.795986	2.761820	-1.284566
H	2.945841	2.503693	-2.344509
H	2.965612	3.842189	-1.192884
C	3.816003	2.019379	-0.418159
N	4.981623	1.680148	-1.055482
O	3.604765	1.719531	0.756389
H	5.130037	2.011235	-2.000284
C	6.119399	1.149476	-0.308258
H	6.996267	1.141452	-0.963053
H	6.346492	1.780776	0.556651
C	5.945689	-0.272780	0.257909
N	4.925526	-1.010084	-0.272986
O	6.714421	-0.678134	1.122474
H	4.347440	-0.574345	-0.979146
C	4.524625	-2.300185	0.259131
H	4.430382	-3.045317	-0.537042
H	5.309624	-2.631755	0.946196
C	3.188871	-2.306476	1.023522
N	2.782073	-1.112597	1.545984
O	2.566097	-3.357069	1.168531
H	3.279068	-0.252910	1.329753
C	1.526829	-1.013117	2.250845
H	1.332473	-1.960672	2.762475
H	1.597455	-0.218254	3.003824
C	0.341749	-0.658964	1.336161
N	-0.865410	-1.075115	1.778008
O	0.486654	-0.003023	0.291071
H	-0.946553	-1.608907	2.640262
C	-2.145206	-0.654333	1.211368
H	-1.992569	0.271993	0.656015
C	-3.104006	-0.443552	2.401240
N	-3.949803	0.602332	2.282942
O	-3.075208	-1.241243	3.346524
H	-3.794448	1.293712	1.546763
C	-4.947718	0.904757	3.299805
H	-5.073998	0.028413	3.938553
H	-4.636787	1.752619	3.923726
H	-5.902340	1.152178	2.823200
C	-2.753332	-1.740969	0.292056
H	-3.770614	-1.449605	0.010538
H	-2.838311	-2.658595	0.881432
C	-1.916651	-2.028015	-0.979276
H	-0.862962	-1.793255	-0.800478
C	-1.963586	-3.522638	-1.327535
C	-2.349870	-1.151457	-2.161088
F	-1.321169	-3.806664	-2.478991
F	-3.227419	-3.986697	-1.444493
F	-1.365469	-4.235354	-0.347699
F	-1.469090	-1.192646	-3.180604
F	-2.442796	0.147856	-1.776419
F	-3.558688	-1.497784	-2.652122

-2249.8383288

Hfl Hairpin/trans

H	-4.012203	4.946798	-0.095043
C	-3.141059	5.123758	0.544078
C	-2.299870	3.860474	0.578373
N	-1.030131	3.967185	0.130456
O	-2.762856	2.786133	0.996110
H	-2.594681	5.995866	0.172799
H	-3.508219	5.330321	1.554382
H	-0.634600	4.845551	-0.192399
C	-0.129077	2.835436	0.103614
H	0.019604	2.425945	1.110847
H	-0.530334	2.024670	-0.512703
C	1.221367	3.289462	-0.457856
N	2.113549	2.280829	-0.608598
O	1.446686	4.471577	-0.736434
H	1.844981	1.334151	-0.322473
C	3.463771	2.526334	-1.056532
H	3.549493	2.447745	-2.151594
H	3.742755	3.553601	-0.788976
C	4.435609	1.553055	-0.385442
N	5.531298	1.214458	-1.136976
O	4.243914	1.081378	0.734611
H	5.673976	1.682427	-2.022876
C	6.635912	0.456933	-0.553654
H	7.481433	0.477671	-1.248121
H	6.960075	0.906321	0.390356
C	6.340573	-1.018248	-0.222233
N	5.223292	-1.553590	-0.797614
O	7.105151	-1.637153	0.509508
H	4.658307	-0.951661	-1.381846
C	4.720102	-2.873758	-0.462153
H	4.510969	-3.458559	-1.363478
H	5.501022	-3.389650	0.105425
C	3.430250	-2.891163	0.377437
N	3.164055	-1.767656	1.105314
O	2.721481	-3.896335	0.394916
H	3.730889	-0.931439	0.995842
C	1.955423	-1.669982	1.888114
H	1.678042	-2.670958	2.232167
H	2.142742	-1.039032	2.765937
C	0.784786	-1.033103	1.117392
N	-0.443869	-1.375235	1.564261
O	0.962295	-0.221918	0.196491
H	-0.562596	-2.067576	2.298551
C	-1.683397	-0.798759	1.054411
H	-1.470956	0.203606	0.679524
C	-2.668425	-0.763368	2.239984
N	-3.429563	0.352348	2.332729
O	-2.741929	-1.736427	2.998022
H	-3.169493	1.182083	1.794667
C	-4.403397	0.514236	3.405847
H	-4.840488	-0.457639	3.646365
H	-3.939592	0.916124	4.316342
H	-5.189180	1.200336	3.076667
C	-2.240219	-1.688685	-0.086915
H	-1.444247	-1.850315	-0.819762
H	-2.483352	-2.663160	0.346386
C	-3.496517	-1.118816	-0.793112
H	-4.040590	-0.444382	-0.122632
C	-4.482907	-2.247792	-1.126562
C	-3.130391	-0.287982	-2.029542
F	-5.563587	-1.810013	-1.804515
F	-4.931977	-2.807845	0.019847
F	-3.916149	-3.230630	-1.857765
F	-4.184447	0.389684	-2.524871
F	-2.621470	-1.038786	-3.028512
F	-2.187626	0.634985	-1.709589

-2249.8371

Pff Helix/-gauche

H	-2.563580	4.769043	-2.181537
C	-2.621331	5.086542	-1.136758
C	-1.709549	4.207203	-0.302716
N	-2.246627	3.692227	0.852368
O	-0.550472	3.950169	-0.640074
H	-2.250368	6.116144	-1.084320
H	-3.664517	5.068205	-0.806586
H	-3.165933	4.008511	1.131686
C	-1.397244	3.066323	1.862162
H	-0.598814	3.743115	2.184868
H	-2.012727	2.826076	2.735553
C	-0.689207	1.779720	1.419013
N	-1.247974	1.095221	0.386339
O	0.333469	1.410081	1.999547
H	-2.049016	1.498982	-0.083830
C	-0.597586	-0.083651	-0.173899
H	-0.399003	-0.794827	0.634704
C	0.797093	0.196704	-0.779997
N	1.108481	1.483353	-1.075642
O	1.568292	-0.749805	-0.962135
H	0.462633	2.242883	-0.866669
C	2.445886	1.826170	-1.533406
H	2.710816	1.251205	-2.425663
H	2.464539	2.889065	-1.792521
C	3.576749	1.557052	-0.524336
N	3.222310	1.376228	0.774111
O	4.743996	1.505153	-0.923553
H	2.244238	1.402412	1.049276
C	4.210137	1.003977	1.774713
H	5.060998	1.691168	1.747364
H	3.749516	1.071868	2.764613
C	4.821615	-0.405132	1.636906
N	4.232807	-1.246674	0.739040
O	5.793753	-0.702678	2.328043
H	3.415622	-0.943189	0.217810
C	4.821367	-2.526827	0.393403
H	5.491780	-2.829155	1.202819
H	4.045038	-3.291221	0.288083
C	5.648392	-2.560834	-0.906680
N	5.892894	-1.364347	-1.510177
O	6.050301	-3.638794	-1.341685
H	5.582897	-0.471954	-1.139184
H	6.471622	-1.376717	-2.338536
C	-1.486918	-0.762596	-1.239125
C	-2.801899	-1.274019	-0.691157
H	-1.680729	-0.060694	-2.057429
H	-0.910966	-1.597324	-1.645684
C	-3.958251	-0.487275	-0.685802
C	-5.181756	-0.941402	-0.190837
C	-5.269280	-2.238553	0.324374
C	-4.134476	-3.056166	0.338504
C	-2.924931	-2.566506	-0.164848
F	-3.903145	0.777728	-1.171575
F	-6.259968	-0.147563	-0.201972
F	-6.429534	-2.691470	0.804574
F	-4.213554	-4.295500	0.830754
F	-1.854339	-3.374643	-0.139038

-2016.2378699

Pff Helix/trans

H	5.711641	-2.337186	2.389807
C	6.269971	-1.901480	1.556809
C	5.332572	-1.043244	0.728225
N	5.403073	-1.206157	-0.636790
O	4.537035	-0.251666	1.239116
H	7.044609	-1.254172	1.983109
H	6.752635	-2.697808	0.981673
H	6.156006	-1.774542	-1.002854
C	4.785752	-0.235628	-1.539386
H	5.145524	0.779469	-1.339495
H	5.055831	-0.496066	-2.568281
C	3.256235	-0.148428	-1.464355
N	2.601707	-1.237069	-0.982260
O	2.681187	0.875014	-1.838222
H	3.156278	-2.012569	-0.641748
C	1.157645	-1.233147	-0.766094
H	0.653656	-1.016607	-1.712986
C	0.680185	-0.121926	0.193100
N	1.578235	0.369845	1.084016
O	-0.488256	0.271820	0.121888
H	2.553214	0.077593	1.060558
C	1.208737	1.465407	1.966891
H	0.327608	1.203068	2.559851
H	2.038901	1.654557	2.654224
C	0.853606	2.791142	1.270343
N	1.249919	2.943681	-0.019550
O	0.228537	3.653283	1.895479
H	1.719381	2.185168	-0.506573
C	0.859174	4.114889	-0.788065
H	1.108137	5.030083	-0.242992
H	1.414932	4.117664	-1.730336
C	-0.639813	4.238608	-1.127276
N	-1.418939	3.147279	-0.876717
O	-1.058878	5.293510	-1.599494
H	-1.001806	2.299439	-0.503488
C	-2.864324	3.199640	-0.988132
H	-3.131239	4.035357	-1.641201
H	-3.249565	2.278643	-1.436985
C	-3.635211	3.398973	0.331476
N	-2.894051	3.690891	1.436414
O	-4.860589	3.293417	0.340243
H	-1.884810	3.797215	1.426298
H	-3.396784	3.861791	2.296250
C	0.734682	-2.638195	-0.267472
C	-0.758739	-2.809696	-0.122330
H	1.217115	-2.841013	0.694637
H	1.105073	-3.370197	-0.994896
C	-1.572115	-3.031049	-1.238633
C	-2.954554	-3.192336	-1.136734
C	-3.558692	-3.135463	0.123899
C	-2.774702	-2.918115	1.260966
C	-1.394000	-2.757765	1.121949
F	-1.006844	-3.093388	-2.460992
F	-3.699198	-3.404180	-2.227738
F	-4.879011	-3.292314	0.240390
F	-3.347462	-2.865298	2.468198
F	-0.663783	-2.546524	2.233082

-2016.2344373

Pff Hairpin/-gauche

H	6.048836	2.806183	2.135930
C	5.528311	3.457476	1.426487
C	4.361016	2.690462	0.830211
N	3.133887	3.228546	1.004499
O	4.533235	1.624729	0.216740
H	5.225122	4.376279	1.937284
H	6.233337	3.704975	0.626383
H	2.986878	4.115394	1.477747
C	1.939474	2.596531	0.486773
H	2.018734	2.435669	-0.595354
H	1.780255	1.612625	0.941463
C	0.737236	3.498769	0.779897
N	-0.447348	2.991604	0.364013
O	0.862092	4.590434	1.345742
H	-0.470194	2.073995	-0.091640
C	-1.677375	3.729978	0.553026
H	-1.967434	3.757010	1.613790
H	-1.538886	4.773924	0.239597
C	-2.788163	3.112245	-0.295303
N	-4.030396	3.104288	0.282045
O	-2.585499	2.633063	-1.411176
H	-4.151305	3.553699	1.180361
C	-5.212403	2.705990	-0.477681
H	-6.105596	2.964263	0.099196
H	-5.257439	3.240836	-1.431602
C	-5.306593	1.211829	-0.843417
N	-4.463820	0.373989	-0.170470
O	-6.115433	0.846666	-1.689324
H	-3.825713	0.784694	0.498008
C	-4.296320	-1.026433	-0.516787
H	-4.358991	-1.663198	0.370885
H	-5.111930	-1.297693	-1.194980
C	-2.961574	-1.362777	-1.205553
N	-2.401803	-0.375157	-1.962317
O	-2.471757	-2.487294	-1.096363
H	-2.732727	0.582001	-1.877568
C	-1.112718	-0.587392	-2.581990
H	-1.091388	-1.576969	-3.048591
H	-0.968485	0.170730	-3.360520
C	0.051008	-0.449285	-1.584524
N	1.138847	-1.203069	-1.852824
O	-0.008116	0.337322	-0.626554
H	1.162741	-1.815566	-2.663177
C	2.380217	-1.131309	-1.094540
H	2.385817	-0.194005	-0.536473
C	3.542799	-1.193124	-2.104004
N	4.616861	-0.436219	-1.787365
O	3.453406	-1.936323	-3.088550
H	4.549603	0.233678	-1.018809
C	5.820319	-0.421366	-2.606322
H	5.772043	-1.253350	-3.311693
H	5.904477	0.515646	-3.171694
H	6.708449	-0.528862	-1.973652
C	2.527518	-2.326298	-0.106607
H	3.435072	-2.164291	0.484728
C	1.335529	-2.484723	0.809071
H	2.662108	-3.242326	-0.688189
C	0.379107	-3.486664	0.606652
C	-0.743967	-3.627559	1.428170
C	-0.933289	-2.734152	2.484394
C	0.002682	-1.721346	2.716525
C	1.116931	-1.617408	1.885195
F	0.529643	-4.359318	-0.403672
F	-1.629954	-4.602057	1.211956
F	-2.012021	-2.840595	3.272400
F	-0.180931	-0.858370	3.728676
F	2.004503	-0.631067	2.132119

-2263.6201249

Pff Hairpin/trans

H	-4.171537	4.008681	0.695848
C	-3.393980	4.106286	1.460803
C	-2.380994	2.992438	1.266671
N	-1.119097	3.368195	0.965441
O	-2.704653	1.796763	1.373183
H	-2.953540	5.105708	1.396564
H	-3.872266	3.982596	2.437350
H	-0.840925	4.342519	0.891624
C	-0.066995	2.402524	0.731666
H	0.112638	1.791237	1.625544
H	-0.335713	1.715534	-0.076849
C	1.221166	3.146707	0.370667
N	2.243832	2.327727	0.025829
O	1.294025	4.379453	0.403918
H	2.096471	1.313618	0.035950
C	3.554269	2.850107	-0.283797
H	3.633877	3.145404	-1.341558
H	3.727687	3.756551	0.310736
C	4.631647	1.818826	0.056717
N	5.740891	1.849013	-0.748441
O	4.510797	1.000976	0.968038
H	5.814837	2.581293	-1.442920
C	6.928768	1.064031	-0.421830
H	7.755845	1.396744	-1.056372
H	7.219326	1.216883	0.622446
C	6.798205	-0.461874	-0.589432
N	5.717212	-0.900568	-1.299717
O	7.651214	-1.196592	-0.103875
H	5.070012	-0.207072	-1.650215
C	5.363637	-2.304630	-1.409503
H	5.182248	-2.586865	-2.451473
H	6.213367	-2.888369	-1.041718
C	4.114265	-2.732149	-0.618719
N	3.757093	-1.934849	0.429830
O	3.518848	-3.764503	-0.923519
H	4.225694	-1.047424	0.588952
C	2.572779	-2.222696	1.202795
H	2.396559	-3.302405	1.183571
H	2.733671	-1.915092	2.243739
C	1.324110	-1.477151	0.698289
N	0.146911	-2.030093	1.062341
O	1.399398	-0.418979	0.055210
H	0.106420	-2.902500	1.583653
C	-1.149280	-1.419379	0.802672
H	-1.030446	-0.334863	0.780475
C	-2.075018	-1.852267	1.955694
N	-2.986059	-0.928904	2.336315
O	-1.958406	-2.984209	2.439965
H	-2.920683	0.016299	1.955683
C	-3.961440	-1.192705	3.384904
H	-4.009922	-2.270381	3.552905
H	-3.678326	-0.703021	4.325757
H	-4.947514	-0.825443	3.080685
C	-1.700377	-1.896946	-0.575635
H	-1.863953	-2.977159	-0.529581
C	-2.975803	-1.200562	-0.992749
H	-0.920676	-1.697950	-1.317092
C	-2.954513	0.072095	-1.574737
F	-1.772797	0.683448	-1.790820
C	-4.117109	0.751985	-1.940625
F	-4.050632	1.978919	-2.476876
C	-5.358951	0.143837	-1.739697
F	-6.483386	0.781857	-2.084513
C	-5.419716	-1.132386	-1.173165
F	-6.607751	-1.720132	-0.978353
C	-4.236673	-1.781470	-0.809064
F	-4.335035	-3.003997	-0.260527

-2263.6198492

$Ser(P)^{-1}$ Helix

H	-4.225263	-2.185773	2.727389
C	-4.410998	-1.793755	1.724341
C	-3.261090	-2.200511	0.826988
N	-3.546472	-2.263942	-0.518251
O	-2.129990	-2.463123	1.262521
H	-5.369079	-2.165015	1.345560
H	-4.476889	-0.696292	1.777049
H	-4.284611	-1.627971	-0.820486
C	-2.461933	-2.520806	-1.459091
H	-1.965700	-3.459380	-1.201021
H	-2.888187	-2.619808	-2.464355
C	-1.350951	-1.452537	-1.490214
N	-1.774614	-0.203212	-1.229302
O	-0.169922	-1.767042	-1.723459
H	-2.796836	-0.007774	-1.126490
C	-0.888515	0.932084	-1.054405
H	-0.295550	1.116382	-1.960592
C	0.175830	0.725760	0.039233
N	-0.075022	-0.197601	0.996643
O	1.230723	1.378792	-0.003801
H	-0.925874	-0.755048	0.971003
C	0.941857	-0.557592	1.964572
H	1.265853	0.314143	2.541386
H	0.515507	-1.291379	2.655715
C	2.225881	-1.150074	1.360727
N	2.135030	-1.665717	0.111416
O	3.281230	-1.129067	2.012211
H	1.251457	-1.639186	-0.403022
C	3.314593	-2.127773	-0.594660
H	3.869031	-2.856819	0.004818
H	2.995338	-2.617620	-1.519513
C	4.347607	-1.046913	-0.968666
N	3.956691	0.250507	-0.826201
O	5.460074	-1.391045	-1.373341
H	3.013714	0.474628	-0.510001
C	4.897702	1.342677	-0.965918
H	5.703437	1.035759	-1.638609
H	4.399862	2.216124	-1.399060
C	5.571600	1.825563	0.333925
N	5.272921	1.139503	1.468682
O	6.337381	2.791504	0.300128
H	4.637184	0.345544	1.502401
H	5.712341	1.451201	2.323309
C	-1.741649	2.193185	-0.784510
H	-2.310472	2.430670	-1.693797
H	-1.065078	3.028588	-0.570924
O	-2.601743	2.024786	0.335756
P	-4.232483	1.683921	0.116440
O	-4.819644	1.513354	1.487795
O	-4.343473	0.586063	-0.970860
O	-4.756072	3.081592	-0.629226
H	-5.120810	2.811096	-1.484010

-1931.3929305

Ser(P)$^{-2}$ Helix

H	4.434226	2.964411	2.448799
C	4.711842	2.250443	1.663780
C	4.066215	2.694093	0.349265
N	3.212899	1.793158	-0.180356
O	4.324281	3.803251	-0.154064
H	5.801023	2.292433	1.547741
H	4.410513	1.239307	1.954834
H	3.115601	0.868234	0.272700
C	2.521715	2.011041	-1.450781
H	2.121563	3.028173	-1.480240
H	3.224961	1.891936	-2.288960
C	1.369448	0.994348	-1.568597
N	1.780964	-0.258820	-1.633632
O	0.170661	1.431161	-1.523056
H	3.360405	-0.870452	-1.377286
C	0.779833	-1.315184	-1.523920
H	0.185385	-1.413876	-2.447893
C	-0.276944	-1.114322	-0.424739
N	0.182849	-0.664598	0.762386
O	-1.486196	-1.373219	-0.625185
H	1.215923	-0.630541	0.932531
C	-0.725540	-0.436769	1.859384
H	-1.276291	-1.344532	2.139180
H	-0.144726	-0.114643	2.731242
C	-1.811092	0.616994	1.594823
N	-1.600417	1.467289	0.569977
O	-2.836166	0.644096	2.309350
H	-0.799259	1.344869	-0.081897
C	-2.647120	2.360189	0.121461
H	-3.003504	3.001173	0.934718
H	-2.231955	2.996176	-0.667001
C	-3.912218	1.682288	-0.438379
N	-3.804591	0.361181	-0.742170
O	-4.949174	2.341449	-0.587650
H	-2.908650	-0.134141	-0.645881
C	-4.965733	-0.422410	-1.094656
H	-5.694902	0.207314	-1.613314
H	-4.672271	-1.237970	-1.763907
C	-5.720150	-1.076320	0.080243
N	-5.218570	-0.857092	1.321247
O	-6.724917	-1.764765	-0.139999
H	-4.388502	-0.292313	1.514272
H	-5.702625	-1.292027	2.093670
C	1.493335	-2.678330	-1.313274
H	2.215215	-2.799665	-2.135796
H	0.740504	-3.476026	-1.387269
O	2.131703	-2.867706	-0.056738
P	3.487776	-1.966822	0.449103
O	2.906888	-0.735047	1.206240
O	4.096181	-1.448978	-0.985526
O	4.430145	-2.906455	1.153302

-1930.7631176

Ser(P)$^{-2}$ Hairpin

H	5.655223	3.802288	0.005158
C	5.012794	3.776280	-0.881662
C	3.959869	2.683504	-0.686560
N	2.683437	3.098868	-0.751719
O	4.312734	1.510182	-0.490645
H	4.582531	4.769926	-1.045587
H	5.639648	3.507547	-1.738845
H	2.436243	4.081536	-0.870931
C	1.502391	2.269219	-0.562012
H	1.413020	1.512378	-1.350455
H	1.521615	1.730920	0.393133
C	0.294271	3.224032	-0.599636
N	-0.901486	2.644073	-0.446447
O	0.478437	4.452110	-0.762385
H	-0.950582	1.594648	-0.275661
C	-2.096559	3.476999	-0.421370
H	-2.006887	4.233860	0.372696
H	-2.205193	4.021585	-1.364862
C	-3.416054	2.719767	-0.212278
N	-3.421890	1.743317	0.723122
O	-4.414577	3.074581	-0.862482
H	-2.592073	1.495697	1.323102
C	-4.621703	0.972196	0.974821
H	-4.701227	0.783207	2.052278
H	-5.498212	1.539860	0.652556
C	-4.741974	-0.414733	0.313579
N	-3.642337	-0.941450	-0.277705
O	-5.832368	-1.005446	0.384253
H	-2.729293	-0.473241	-0.163930
C	-3.706653	-2.332596	-0.714988
H	-3.928826	-2.984589	0.141845
H	-4.543648	-2.455696	-1.409363
C	-2.489117	-2.964545	-1.401044
N	-1.289108	-2.351329	-1.332255
O	-2.664543	-4.059883	-1.966392
H	-1.151561	-1.450202	-0.822121
C	-0.112764	-3.010853	-1.883724
H	-0.175381	-4.087150	-1.675720
H	-0.058189	-2.895209	-2.972923
C	1.203975	-2.449489	-1.330605
N	1.270146	-2.263967	0.010199
O	2.145285	-2.204890	-2.100953
H	0.422476	-2.349145	0.600219
C	2.318704	-1.413633	0.554835
H	2.455366	-0.558259	-0.116268
C	3.680128	-2.121115	0.680015
N	4.728888	-1.292801	0.379533
O	3.823748	-3.280046	1.079037
H	4.530053	-0.365290	0.009340
C	6.103123	-1.732200	0.462230
H	6.105805	-2.735608	0.897247
H	6.574999	-1.778268	-0.530824
H	6.701381	-1.061229	1.096028
C	1.904189	-0.863410	1.943145
H	1.555039	-1.695352	2.568305
O	0.926309	0.161660	1.874472
H	2.793393	-0.415218	2.407247
P	-0.718301	-0.275130	1.604423
O	-0.932482	0.003607	0.041353
O	-1.531089	0.749505	2.416107
O	-0.790514	-1.778324	1.913939

-2178.1477187

AcK Helix/-gauche

H	2.974274	4.105981	2.429499
C	2.797748	4.712072	1.536506
C	1.734021	4.041604	0.687478
N	2.017536	3.905586	-0.651259
O	0.675827	3.623510	1.164195
H	2.413104	5.682685	1.868663
H	3.743579	4.867066	1.008196
H	2.846294	4.363034	-1.008123
C	0.972543	3.536978	-1.604113
H	0.115711	4.215588	-1.534486
H	1.382977	3.609041	-2.616843
C	0.392768	2.126522	-1.430878
N	1.167244	1.221630	-0.781968
O	-0.726773	1.866268	-1.878968
H	2.048096	1.538306	-0.396752
C	0.687654	-0.120277	-0.458655
H	0.353253	-0.605250	-1.382654
C	-0.564936	-0.125105	0.441519
N	-0.828004	0.989768	1.170788
O	-1.282361	-1.130774	0.466545
H	-0.247630	1.821655	1.084574
C	-2.049765	1.079963	1.953893
H	-2.116400	0.253661	2.667819
H	-2.036280	2.018738	2.516121
C	-3.356902	1.023605	1.143175
N	-3.273860	1.279446	-0.187989
O	-4.415463	0.747880	1.716943
H	-2.372031	1.459288	-0.622085
C	-4.434805	1.132883	-1.050975
H	-5.284423	1.691671	-0.647501
H	-4.194009	1.543312	-2.036002
C	-4.954688	-0.304238	-1.256101
N	-4.164681	-1.315982	-0.794923
O	-6.037125	-0.472628	-1.815015
H	-3.268007	-1.108232	-0.363608
C	-4.623333	-2.691572	-0.771311
H	-5.443612	-2.795013	-1.487343
H	-3.819157	-3.371313	-1.070423
C	-5.146307	-3.203609	0.584735
N	-5.308345	-2.275698	1.568245
O	-5.398153	-4.399538	0.726580
H	-5.111350	-1.287213	1.448228
H	-5.684797	-2.598001	2.448879
C	1.810506	-0.955810	0.179226
H	1.357266	-1.881180	0.550253
H	2.203424	-0.422184	1.057558
C	2.956376	-1.292149	-0.788504
H	3.337888	-0.375440	-1.260181
H	2.562869	-1.916208	-1.603583
C	4.120446	-2.007700	-0.088994
H	3.761804	-2.930273	0.388419
H	4.521883	-1.367041	0.705600
C	5.267262	-2.335597	-1.059902
H	5.574263	-1.421418	-1.579364
H	4.935462	-3.059089	-1.814811
N	6.449738	-2.873909	-0.396042
C	7.377186	-2.055490	0.194095
H	6.538337	-3.873944	-0.283831
O	7.285511	-0.827647	0.161384
C	8.539631	-2.761380	0.879326
H	8.571354	-2.448656	1.927795
H	9.472170	-2.432290	0.409850
H	8.482153	-3.853939	0.831732

-1614.8872862

AcK Helix/trans

H	1.170408	5.646822	2.332264
C	0.556474	6.140756	1.573716
C	-0.171423	5.084018	0.763427
N	-0.084668	5.196963	-0.604616
O	-0.794002	4.160973	1.293837
H	-0.189512	6.746745	2.099427
H	1.185732	6.799828	0.967484
H	0.335516	6.036143	-0.982394
C	-0.963290	4.420741	-1.477883
H	-2.016698	4.582292	-1.225135
H	-0.806502	4.748939	-2.510827
C	-0.761774	2.900073	-1.430390
N	0.444529	2.453939	-1.000928
O	-1.679586	2.152156	-1.776467
H	1.129049	3.137637	-0.703428
C	0.733491	1.032085	-0.813860
H	0.595173	0.509391	-1.766739
C	-0.251105	0.347014	0.155620
N	-0.861837	1.127875	1.085382
O	-0.443724	-0.871360	0.074857
H	-0.747090	2.139177	1.073700
C	-1.845502	0.559080	1.993228
H	-1.409855	-0.257896	2.576267
H	-2.168877	1.339787	2.688690
C	-3.099087	-0.040605	1.331403
N	-3.372820	0.334929	0.055948
O	-3.799357	-0.831986	1.971691
H	-2.735738	0.947133	-0.447916
C	-4.464937	-0.279158	-0.682523
H	-5.392987	-0.227250	-0.105747
H	-4.611884	0.272779	-1.615588
C	-4.292207	-1.769636	-1.040109
N	-3.055790	-2.308972	-0.840014
O	-5.256393	-2.393236	-1.480340
H	-2.295821	-1.728292	-0.495573
C	-2.812532	-3.732561	-0.969803
H	-3.611356	-4.165808	-1.578639
H	-1.859028	-3.917148	-1.474781
C	-2.770577	-4.529323	0.348511
N	-3.171359	-3.873326	1.472855
O	-2.392807	-5.700312	0.340545
H	-3.496641	-2.911690	1.478379
H	-3.188955	-4.402514	2.333515
C	2.192752	0.866648	-0.338220
H	2.826252	1.517961	-0.957689
H	2.277282	1.226942	0.697198
C	2.724345	-0.571569	-0.439423
H	2.739815	-0.868609	-1.497479
H	2.034745	-1.258113	0.064065
C	4.134247	-0.707094	0.152724
H	4.104759	-0.504624	1.232946
H	4.811095	0.028943	-0.298528
C	4.724542	-2.106492	-0.089099
H	4.802979	-2.283866	-1.167150
H	4.065016	-2.875089	0.332231
N	6.053458	-2.282796	0.489380
C	7.184469	-1.825308	-0.133152
H	6.138710	-2.720644	1.395476
O	7.151068	-1.245646	-1.218704
C	8.493390	-2.092218	0.600096
H	9.003397	-1.138208	0.767028
H	9.135535	-2.699770	-0.045610
H	8.365643	-2.604702	1.559565

-1614.8854751

AcK Hairpin/-gauche

H	4.336679	-4.372607	-2.772982
C	3.408872	-4.880495	-2.490458
C	2.564755	-3.925890	-1.664039
N	1.339936	-3.627072	-2.152871
O	2.983842	-3.445637	-0.598992
H	2.902243	-5.232493	-3.394111
H	3.678789	-5.740375	-1.869121
H	0.976769	-4.035376	-3.009223
C	0.437902	-2.733398	-1.459981
H	0.242255	-3.084406	-0.439423
H	0.863903	-1.728110	-1.365405
C	-0.880024	-2.659002	-2.236957
N	-1.791992	-1.819152	-1.693523
O	-1.069449	-3.317680	-3.265589
H	-1.555982	-1.306814	-0.836769
C	-3.096104	-1.648869	-2.295293
H	-3.036137	-1.059805	-3.222764
H	-3.508433	-2.628556	-2.573320
C	-4.046821	-0.987486	-1.298275
N	-4.945018	-0.106412	-1.841504
O	-4.002522	-1.208137	-0.088153
H	-4.972180	-0.000629	-2.847369
C	-6.036050	0.445764	-1.042716
H	-6.742676	0.947093	-1.711087
H	-6.572827	-0.350792	-0.517616
C	-5.634027	1.454329	0.050214
N	-4.365941	1.955536	-0.025817
O	-6.450107	1.766199	0.910692
H	-3.768870	1.619618	-0.769774
C	-3.781778	2.781502	1.016117
H	-3.333838	3.688326	0.597691
H	-4.590926	3.078600	1.690907
C	-2.684676	2.097988	1.852346
N	-2.741964	0.737712	1.931612
O	-1.842738	2.778781	2.438287
H	-3.368794	0.213852	1.327191
C	-1.712550	0.001895	2.631299
H	-1.410114	0.567278	3.517580
H	-2.122783	-0.962639	2.953666
C	-0.489239	-0.295796	1.744770
N	0.688036	-0.376069	2.395602
O	-0.607712	-0.483498	0.522726
H	0.729428	-0.267495	3.405354
C	1.945344	-0.793678	1.778621
H	1.709102	-1.303296	0.841666
C	2.631858	-1.749832	2.770515
N	3.329264	-2.760872	2.202514
O	2.550840	-1.535857	3.986921
H	3.239877	-2.923135	1.198899
C	4.082718	-3.722693	2.992605
H	4.145975	-3.354262	4.018504
H	3.590539	-4.703942	3.000578
H	5.092959	-3.841861	2.584498
C	2.880639	0.404514	1.490375
H	3.814297	0.007577	1.070058
H	3.137892	0.881601	2.445620
C	2.277381	1.440276	0.531832
H	1.343790	1.830187	0.958031
H	2.007045	0.951531	-0.414349
C	3.233705	2.607782	0.248828
H	3.539881	3.071319	1.198349
H	4.147231	2.241651	-0.237621
C	2.587484	3.671662	-0.653496
H	1.671030	4.051303	-0.182197
H	2.316558	3.230724	-1.617209
N	3.468879	4.797347	-0.944358
H	3.651827	5.466872	-0.209672
C	4.179325	4.900289	-2.112275
O	4.078086	4.086726	-3.030154
C	5.102985	6.109321	-2.210508
H	6.125845	5.754197	-2.371873
H	5.082779	6.755540	-1.326278
H	4.816351	6.695923	-3.089084

-1862.2667855

AcK Hairpin/trans

H	-4.236267	4.505957	-1.271329
C	-3.269681	4.920177	-0.966680
C	-2.373851	3.780511	-0.513933
N	-1.219451	3.613412	-1.197166
O	-2.692268	3.041103	0.431497
H	-2.848403	5.495931	-1.796128
H	-3.448445	5.586993	-0.117199
H	-0.937474	4.227192	-1.956106
C	-0.278632	2.565539	-0.866682
H	0.047492	2.643330	0.177668
H	-0.730412	1.573270	-0.980364
C	0.938948	2.682982	-1.788715
N	1.868633	1.718890	-1.589264
O	1.035727	3.585347	-2.627373
H	1.717275	1.025386	-0.848736
C	3.105552	1.702888	-2.334596
H	2.992329	1.180616	-3.297408
H	3.396267	2.735430	-2.568481
C	4.216113	1.050091	-1.509575
N	5.157165	0.368423	-2.238119
O	4.259197	1.105680	-0.281477
H	5.108836	0.409054	-3.248072
C	6.381592	-0.121995	-1.610519
H	7.076883	-0.436407	-2.394903
H	6.863068	0.669375	-1.027151
C	6.216382	-1.303546	-0.636243
N	5.025572	-1.969702	-0.690700
O	7.140110	-1.598374	0.114459
H	4.327939	-1.638162	-1.343507
C	4.658515	-3.004647	0.259267
H	4.301531	-3.903765	-0.253099
H	5.559108	-3.268883	0.822362
C	3.563298	-2.616680	1.269102
N	3.378952	-1.281951	1.482827
O	2.935668	-3.496494	1.857100
H	3.869448	-0.595177	0.916936
C	2.344865	-0.811717	2.374508
H	2.148301	-1.590391	3.117412
H	2.695192	0.087465	2.897089
C	1.043107	-0.432428	1.645171
N	-0.064932	-0.451278	2.411580
O	1.036061	-0.077640	0.454894
H	-0.038031	-0.785856	3.370856
C	-1.384410	-0.026792	1.945502
H	-1.249625	0.762658	1.202403
C	-2.125562	0.513262	3.180945
N	-2.876391	1.618473	2.964990
O	-2.017413	-0.071188	4.266797
H	-2.836646	2.072719	2.052122
C	-3.666293	2.240124	4.017081
H	-3.601899	1.616167	4.910784
H	-3.285559	3.241574	4.254037
H	-4.715684	2.329698	3.711521
C	-2.145296	-1.208137	1.299316
H	-2.276185	-1.992105	2.056602
H	-1.496133	-1.618799	0.515689
C	-3.503254	-0.827236	0.691604
H	-3.374096	0.019673	0.002583
H	-4.180814	-0.479274	1.483038
C	-4.150563	-2.000079	-0.058906
H	-3.494681	-2.330433	-0.874463
H	-4.269178	-2.854842	0.623060
C	-5.516981	-1.626071	-0.656088
H	-5.398987	-0.795966	-1.358974
H	-6.200765	-1.302428	0.139843
N	-6.145417	-2.718115	-1.392607
H	-6.600972	-3.453287	-0.869721
C	-5.970490	-2.889946	-2.741722
O	-5.340193	-2.094722	-3.437850
C	-6.627544	-4.131648	-3.333724
H	-5.851521	-4.750746	-3.795273
H	-7.313121	-3.817984	-4.127173
H	-7.177692	-4.732632	-2.601740

-1862.2667117

M(O_2) Helix/-gauche

H	-4.433018	-1.872519	2.208541
C	-4.359089	-2.800881	1.632003
C	-3.144258	-2.714239	0.728551
N	-3.390683	-2.592159	-0.611354
O	-1.989707	-2.727261	1.172038
H	-4.210518	-3.622616	2.338137
H	-5.295504	-2.950938	1.086332
H	-4.347096	-2.504778	-0.925653
C	-2.307596	-2.530799	-1.581413
H	-1.683638	-3.428305	-1.523639
H	-2.736786	-2.482540	-2.587719
C	-1.340052	-1.350175	-1.406313
N	-1.824031	-0.266772	-0.749903
O	-0.184743	-1.427168	-1.836927
H	-2.808513	-0.243805	-0.481356
C	-0.968923	0.865276	-0.425568
H	-0.522242	1.265821	-1.344189
C	0.253529	0.490169	0.444392
N	0.181698	-0.652391	1.168887
O	1.232952	1.243973	0.451790
H	-0.622532	-1.274786	1.089619
C	1.333244	-1.109638	1.930687
H	1.657814	-0.344034	2.641619
H	1.045426	-2.002084	2.494552
C	2.582720	-1.447717	1.097407
N	2.404280	-1.654844	-0.232241
O	3.684239	-1.508524	1.653719
H	1.481643	-1.558164	-0.650452
C	3.538968	-1.868162	-1.115197
H	4.185143	-2.660055	-0.724746
H	3.167164	-2.184054	-2.094304
C	4.468368	-0.657739	-1.334754
N	4.035089	0.544719	-0.857403
O	5.538427	-0.823897	-1.917525
H	3.128720	0.615260	-0.403454
C	4.895371	1.711957	-0.836551
H	5.693544	1.566232	-1.570019
H	4.334421	2.609976	-1.114385
C	5.576562	2.022455	0.510638
N	5.464701	1.078108	1.485088
O	6.186245	3.081752	0.652660
H	4.967326	0.201116	1.366268
H	5.936806	1.259889	2.359860
C	-1.744985	1.986526	0.290354
H	-1.019783	2.773929	0.512546
H	-2.122128	1.635621	1.259213
C	-2.896674	2.610418	-0.529360
H	-2.806585	2.414696	-1.603133
H	-2.960422	3.690060	-0.362082
S	-4.521607	1.957246	0.008444
C	-5.724562	2.807048	-1.044912
H	-6.702057	2.418958	-0.747033
H	-5.523337	2.570988	-2.092474
H	-5.669830	3.881191	-0.853528
O	-4.746985	2.361267	1.411792
O	-4.578104	0.503030	-0.336913

-1916.1266175

M(O_2) Helix/trans

H	-4.858161	-2.882219	2.340699
C	-4.771079	-3.658042	1.574819
C	-3.524230	-3.399831	0.750850
N	-3.675424	-3.434946	-0.616013
O	-2.431113	-3.153659	1.267172
H	-4.651452	-4.617124	2.090783
H	-5.688756	-3.687710	0.979293
H	-4.560223	-3.757921	-0.985477
C	-2.508703	-3.483926	-1.494972
H	-1.856250	-4.327030	-1.243744
H	-2.853724	-3.614418	-2.526135
C	-1.607091	-2.243752	-1.452633
N	-2.179781	-1.083856	-1.039761
O	-0.423933	-2.331725	-1.787866
H	-3.145868	-1.106203	-0.737753
C	-1.402716	0.139478	-0.861977
H	-0.904226	0.385048	-1.806296
C	-0.248872	-0.008819	0.151384
N	-0.330549	-1.004588	1.066739
O	0.702120	0.781701	0.096261
H	-1.097091	-1.675471	1.045439
C	0.767752	-1.246698	1.990254
H	0.979996	-0.353946	2.585883
H	0.476317	-2.051328	2.672178
C	2.108127	-1.630385	1.337280
N	2.069665	-2.094192	0.061083
O	3.153614	-1.501788	1.981557
H	1.188360	-2.138941	-0.444115
C	3.297600	-2.369672	-0.668346
H	3.950487	-3.024661	-0.084047
H	3.044972	-2.883873	-1.600299
C	4.164264	-1.145598	-1.027097
N	3.600620	0.081508	-0.833029
O	5.299387	-1.324383	-1.464089
H	2.648628	0.154715	-0.484940
C	4.364896	1.306230	-0.967970
H	5.268245	1.085264	-1.544085
H	3.791288	2.065111	-1.509763
C	4.818174	1.961782	0.350602
N	4.687470	1.214106	1.481447
O	5.277357	3.103154	0.336610
H	4.317957	0.268772	1.491632
H	5.023600	1.619022	2.344169
C	-2.361551	1.281665	-0.469841
H	-3.162748	1.328556	-1.218064
H	-2.821358	1.064392	0.502420
C	-1.673552	2.650357	-0.407280
H	-1.234997	2.929521	-1.370437
H	-0.905303	2.693936	0.367905
S	-2.940474	3.906801	-0.010802
C	-2.002954	5.455873	0.079990
H	-2.737010	6.230092	0.317881
H	-1.540985	5.656075	-0.889606
H	-1.257423	5.381536	0.875046
O	-3.469799	3.603789	1.340711
O	-3.869018	3.991884	-1.164027

-1916.1204485

$M(O_2)$ Hairpin/-gauche

H	-4.785576	4.149383	-2.400386
C	-4.161018	4.654665	-1.656678
C	-3.230954	3.631876	-1.028575
N	-1.903807	3.865435	-1.151838
O	-3.668951	2.632624	-0.437194
H	-3.631283	5.484072	-2.134810
H	-4.825284	5.049553	-0.881348
H	-1.532784	4.695036	-1.605240
C	-0.920133	2.966701	-0.588958
H	-1.064873	2.853603	0.492899
H	-1.004024	1.961757	-1.016371
C	0.479752	3.525391	-0.858783
N	1.486496	2.725087	-0.431889
O	0.654479	4.614088	-1.416382
H	1.263452	1.826358	0.008345
C	2.869382	3.127435	-0.597899
H	3.110636	3.262296	-1.661209
H	3.050809	4.095293	-0.108882
C	3.782315	2.089553	0.052095
N	4.885039	1.731311	-0.676353
O	3.533924	1.585619	1.148417
H	5.067346	2.210096	-1.548672
C	5.921820	0.873096	-0.108713
H	6.787476	0.876930	-0.777907
H	6.245454	1.250810	0.866387
C	5.524306	-0.595786	0.135161
N	4.375017	-1.017996	-0.471992
O	6.236498	-1.307014	0.834572
H	3.848037	-0.339181	-1.005129
C	3.754950	-2.299407	-0.183916
H	3.498487	-2.830261	-1.105762
H	4.483991	-2.899186	0.371030
C	2.461846	-2.203893	0.645392
N	2.408572	-1.201899	1.565815
O	1.556078	-3.028283	0.490664
H	3.069618	-0.432274	1.506193
C	1.189802	-0.969259	2.313130
H	0.881425	-1.889831	2.819034
H	1.391129	-0.204291	3.070816
C	0.054903	-0.462116	1.404638
N	-1.187157	-0.850663	1.749235
O	0.294412	0.264965	0.425331
H	-1.348175	-1.430610	2.568131
C	-2.386001	-0.523268	0.983005
H	-2.173388	0.360360	0.377762
C	-3.512094	-0.249042	1.997534
N	-4.380390	0.724223	1.639619
O	-3.585076	-0.931972	3.025432
H	-4.156041	1.320406	0.842514
C	-5.535177	1.068410	2.456116
H	-5.680568	0.282685	3.200156
H	-5.385362	2.023130	2.976913
H	-6.430374	1.147478	1.829232
C	-2.809560	-1.705863	0.071991
H	-3.675186	-1.396195	-0.524537
H	-3.122611	-2.545066	0.703134
C	-1.666956	-2.163393	-0.840672
H	-0.772500	-2.415458	-0.263965
H	-1.414776	-1.421441	-1.604190
S	-2.124758	-3.690607	-1.734458
C	-0.520046	-4.309310	-2.317906
H	0.145093	-4.444851	-1.461304
H	-0.106201	-3.598416	-3.037521
H	-0.732042	-5.263869	-2.806360
O	-2.940615	-3.322952	-2.917540
O	-2.652625	-4.663556	-0.747996

-2163.5008965

$M(O_2)$ Hairpin/trans

H	5.331063	3.348444	-1.606591
C	4.358317	3.453472	-2.097811
C	3.380751	2.478104	-1.464892
N	2.261378	3.006639	-0.933307
O	3.613900	1.253222	-1.448244
H	4.032271	4.495605	-2.031413
H	4.489757	3.184277	-3.150903
H	2.066864	4.003753	-0.963236
C	1.219546	2.197194	-0.328818
H	0.999353	1.328225	-0.953361
H	1.522813	1.826142	0.660550
C	-0.038432	3.053263	-0.174880
N	-1.156175	2.335014	0.091621
O	-0.001988	4.284889	-0.271644
H	-1.115545	1.314877	0.025462
C	-2.437704	2.976018	0.273480
H	-2.607976	3.244012	1.327715
H	-2.450334	3.912982	-0.298599
C	-3.567745	2.073659	-0.226852
N	-4.759546	2.215199	0.440139
O	-3.427324	1.264120	-1.141001
H	-4.839594	2.944543	1.137038
C	-5.978604	1.595541	-0.071764
H	-6.837782	2.017843	0.458458
H	-6.102135	1.805091	-1.139180
C	-6.066398	0.063867	0.056950
N	-5.155641	-0.527080	0.885493
O	-6.936143	-0.543502	-0.558545
H	-4.483808	0.067409	1.352529
C	-5.046490	-1.966974	1.030763
H	-5.046950	-2.259177	2.085928
H	-5.925420	-2.413474	0.555621
C	-3.799359	-2.613651	0.403814
N	-3.126107	-1.872281	-0.523868
O	-3.480433	-3.755809	0.730997
H	-3.403928	-0.915083	-0.721576
C	-1.929617	-2.381384	-1.152363
H	-1.851426	-3.445246	-0.908437
H	-2.002020	-2.284159	-2.244594
C	-0.663266	-1.629268	-0.717861
N	0.501390	-2.274246	-0.955337
O	-0.707223	-0.486314	-0.243317
H	0.527110	-3.234499	-1.287551
C	1.801585	-1.656732	-0.724549
H	1.749182	-0.609197	-1.029153
C	2.817695	-2.405135	-1.603908
N	3.822466	-1.634762	-2.090909
O	2.691938	-3.617394	-1.808560
H	3.791234	-0.627953	-1.931387
C	4.887092	-2.180139	-2.921125
H	4.799749	-3.268436	-2.922005
H	4.807250	-1.818669	-3.954159
H	5.868742	-1.894554	-2.525411
C	2.170003	-1.704085	0.780022
H	2.203793	-2.742833	1.121245
H	1.352888	-1.203545	1.310326
C	3.499837	-0.993665	1.089834
H	3.682079	-0.154484	0.410835
H	4.355222	-1.675705	1.074824
S	3.418891	-0.253082	2.753913
C	5.067532	0.470875	2.986487
H	5.257241	1.201266	2.196415
H	5.814229	-0.326686	2.987490
H	5.037818	0.963627	3.961689
O	3.243885	-1.324037	3.761243
O	2.442540	0.867298	2.694427

-2163.5010081

C(SMe) Helix/-gauche

H	4.674903	-3.152772	-2.290545
C	4.826346	-3.436409	-1.245404
C	3.687683	-2.876959	-0.412940
N	4.043558	-2.229226	0.747210
O	2.507536	-2.979720	-0.755578
H	4.788824	-4.530179	-1.192702
H	5.813838	-3.099549	-0.914803
H	5.010582	-2.280618	1.039708
C	3.039988	-1.891896	1.753588
H	2.487301	-2.779349	2.080502
H	3.549024	-1.467493	2.625692
C	1.969968	-0.893762	1.292321
N	2.307840	-0.067245	0.271581
O	0.865128	-0.879802	1.840787
H	3.263413	-0.070772	-0.066286
C	1.365590	0.882441	-0.303532
H	1.018606	1.587186	0.460458
C	0.076680	0.216141	-0.836710
N	0.155150	-1.091611	-1.194879
O	-0.951262	0.890523	-0.941047
H	1.001661	-1.629823	-1.018376
C	-1.030128	-1.799570	-1.653218
H	-1.470121	-1.297863	-2.520341
H	-0.737315	-2.809682	-1.955541
C	-2.173391	-1.919364	-0.629883
N	-1.867673	-1.699888	0.674365
O	-3.309718	-2.199861	-1.024342
H	-0.924261	-1.432569	0.943678
C	-2.906253	-1.679991	1.691851
H	-3.523336	-2.580958	1.625688
H	-2.432263	-1.663416	2.677612
C	-3.899824	-0.502320	1.628662
N	-3.595508	0.512554	0.769824
O	-4.903166	-0.529606	2.338953
H	-2.734265	0.482655	0.231601
C	-4.534250	1.582042	0.488592
H	-5.248547	1.642400	1.314558
H	-4.013089	2.541786	0.415276
C	-5.358955	1.432410	-0.804931
N	-5.258868	0.243500	-1.461975
O	-6.062772	2.365930	-1.187226
H	-4.694505	-0.534565	-1.136266
H	-5.827792	0.124938	-2.288693
C	2.010321	1.666248	-1.458068
H	1.297971	2.410069	-1.820279
H	2.259316	0.995098	-2.291240
S	3.614710	2.494011	-1.073545
S	3.103563	3.977027	0.307587
C	3.408983	3.188073	1.939328
H	3.215869	3.972573	2.679705
H	4.449297	2.864711	2.023366
H	2.728491	2.352779	2.119365

-2124.5684771

C(SMe) Helix/trans

H	-5.277206	-2.201656	2.382078
C	-5.268099	-2.956735	1.591358
C	-3.998215	-2.802994	0.775272
N	-4.144843	-2.836251	-0.592547
O	-2.893169	-2.642500	1.298511
H	-5.250557	-3.939669	2.075385
H	-6.181685	-2.873723	0.994338
H	-5.052200	-3.089437	-0.961879
C	-2.984788	-3.003175	-1.466544
H	-2.413080	-3.899227	-1.201973
H	-3.338408	-3.116742	-2.496770
C	-1.971182	-1.851674	-1.442895
N	-2.429211	-0.638884	-1.036602
O	-0.805807	-2.053896	-1.788347
H	-3.387140	-0.572555	-0.716408
C	-1.532902	0.505719	-0.877011
H	-1.031473	0.703128	-1.830711
C	-0.383213	0.249203	0.121185
N	-0.567919	-0.712117	1.062486
O	0.646760	0.923423	0.033881
H	-1.397959	-1.301535	1.053385
C	0.504123	-1.048885	1.986056
H	0.831528	-0.162392	2.537365
H	0.126701	-1.779795	2.707668
C	1.781068	-1.623345	1.347322
N	1.691722	-2.080610	0.072232
O	2.827497	-1.640034	2.003161
H	0.816578	-2.009686	-0.439774
C	2.876071	-2.522767	-0.646876
H	3.425355	-3.264841	-0.060053
H	2.562390	-2.992905	-1.583553
C	3.910691	-1.432671	-0.992344
N	3.526604	-0.137316	-0.806837
O	5.015023	-1.772597	-1.412783
H	2.588598	0.074740	-0.479163
C	4.465663	0.961450	-0.933690
H	5.292580	0.641389	-1.573733
H	3.982642	1.824199	-1.403042
C	5.091205	1.469778	0.380171
N	4.816459	0.754859	1.506703
O	5.797190	2.476837	0.366690
H	4.258880	-0.093013	1.513005
H	5.258828	1.057585	2.363342
C	-2.369105	1.734635	-0.473635
H	-2.789283	1.596959	0.529528
H	-3.186363	1.858941	-1.190744
S	-1.362392	3.290692	-0.524386
S	-2.870115	4.737101	-0.505066
C	-3.070386	5.106989	1.284551
H	-3.790417	5.931191	1.342029
H	-2.119097	5.427095	1.715124
H	-3.464314	4.246472	1.831293

-2124.5617978

C(SMe) Hairpin/-gauche

H	5.651328	3.720649	0.693050
C	4.841558	4.122526	0.075499
C	3.766379	3.059734	-0.067605
N	2.533560	3.383944	0.379744
O	4.015193	1.951065	-0.569576
H	4.474117	5.050330	0.524084
H	5.256613	4.339305	-0.914012
H	2.310477	4.300487	0.756963
C	1.420873	2.463186	0.289434
H	1.298151	2.101574	-0.737998
H	1.580282	1.580790	0.922010
C	0.141054	3.181051	0.727126
N	-0.966755	2.407319	0.643881
O	0.149856	4.354537	1.114748
H	-0.883053	1.459728	0.265058
C	-2.278391	2.923706	0.954889
H	-2.497326	2.856415	2.031947
H	-2.319780	3.988652	0.690404
C	-3.344107	2.170574	0.155316
N	-4.569035	2.073506	0.767478
O	-3.125776	1.664291	-0.943230
H	-4.715142	2.568869	1.637741
C	-5.732265	1.585542	0.031561
H	-6.633570	1.802958	0.612941
H	-5.821271	2.092090	-0.934872
C	-5.746641	0.080668	-0.294032
N	-4.840857	-0.692233	0.374764
O	-6.558452	-0.352101	-1.105039
H	-4.222117	-0.235992	1.031881
C	-4.663879	-2.106973	0.103793
H	-4.717730	-2.696859	1.024643
H	-5.484868	-2.421588	-0.548031
C	-3.343773	-2.499725	-0.581988
N	-2.667494	-1.503876	-1.225817
O	-2.972928	-3.672513	-0.552894
H	-2.990064	-0.541811	-1.170537
C	-1.400904	-1.766065	-1.870248
H	-1.286443	-2.850679	-1.953831
H	-1.395403	-1.338517	-2.882339
C	-0.211999	-1.151114	-1.116083
N	0.989347	-1.716562	-1.370326
O	-0.347703	-0.174748	-0.367381
H	1.083810	-2.485145	-2.030100
C	2.254511	-1.153742	-0.916483
H	2.128331	-0.082052	-0.750743
C	3.285466	-1.402222	-2.040895
N	4.268310	-0.477609	-2.114637
O	3.181921	-2.401520	-2.761895
H	4.204029	0.359940	-1.533213
C	5.356380	-0.574476	-3.076584
H	5.291410	-1.542368	-3.577453
H	5.287584	0.220326	-3.829921
H	6.324451	-0.492352	-2.568727
C	2.787934	-1.809217	0.377044
H	2.831846	-2.895265	0.251256
S	1.713755	-1.402176	1.833503
H	3.791040	-1.429232	0.587472
S	2.787531	-2.264475	3.404206
C	2.114133	-3.973073	3.475964
H	2.606737	-4.455697	4.327796
H	2.346853	-4.530063	2.564982
H	1.034739	-3.953863	3.642481

-2371.9477732

C(SMe) Hairpin/trans

H	4.956930	4.403933	0.161998
C	4.071405	4.643666	-0.435644
C	3.136215	3.447392	-0.417128
N	1.906854	3.650824	0.109402
O	3.487629	2.342986	-0.858549
H	3.612057	5.557070	-0.045940
H	4.403117	4.815476	-1.464464
H	1.593263	4.560601	0.434363
C	0.921398	2.594223	0.177463
H	0.765811	2.141198	-0.808857
H	1.246960	1.789375	0.848273
C	-0.402201	3.179138	0.679463
N	-1.401659	2.267832	0.768345
O	-0.517622	4.374321	0.969504
H	-1.228566	1.308460	0.452779
C	-2.740361	2.651616	1.148070
H	-2.899547	2.552242	2.233255
H	-2.885660	3.711261	0.901541
C	-3.776332	1.815395	0.392182
N	-4.952678	1.604345	1.066257
O	-3.572383	1.340846	-0.723901
H	-5.093154	2.074169	1.951487
C	-6.104611	1.007254	0.395711
H	-6.986216	1.133031	1.031735
H	-6.300842	1.506532	-0.558583
C	-5.987772	-0.490508	0.056049
N	-4.977806	-1.172505	0.672747
O	-6.791197	-0.997344	-0.719354
H	-4.368654	-0.655756	1.292947
C	-4.645847	-2.548454	0.349938
H	-4.574015	-3.161536	1.254040
H	-5.457493	-2.946349	-0.267279
C	-3.326202	-2.747917	-0.416267
N	-2.850148	-1.666404	-1.099416
O	-2.780903	-3.850683	-0.417304
H	-3.293575	-0.757261	-1.001836
C	-1.597679	-1.737662	-1.813583
H	-1.403576	-2.784660	-2.064909
H	-1.672354	-1.164659	-2.746818
C	-0.416381	-1.154149	-1.019111
N	0.804628	-1.569992	-1.420338
O	-0.575346	-0.316294	-0.118862
H	0.919134	-2.302974	-2.115599
C	2.049831	-1.047675	-0.865512
H	1.913878	0.008656	-0.624118
C	3.126659	-1.237220	-1.950910
N	4.009659	-0.221811	-2.064376
O	3.138537	-2.278973	-2.618628
H	3.840191	0.647272	-1.555249
C	5.128357	-0.268437	-2.994229
H	5.222591	-1.287339	-3.374857
H	4.968204	0.411915	-3.840420
H	6.054079	0.018649	-2.483357
C	2.408806	-1.825330	0.422654
H	2.617729	-2.869660	0.172906
S	3.874824	-1.073883	1.272499
H	1.563393	-1.771290	1.112272
S	3.741426	-1.901145	3.184121
C	4.680940	-3.472698	3.025290
H	4.685369	-3.923279	4.024416
H	5.708231	-3.275166	2.710607
H	4.193706	-4.156174	2.325537

-2371.94695

Abu Helix/-gauche

H	5.568091	-1.549098	-2.419499
C	5.706981	-2.033008	-1.448701
C	4.484462	-1.772950	-0.588076
N	4.722637	-1.340568	0.696053
O	3.336324	-1.922861	-1.012146
H	5.780333	-3.111524	-1.627173
H	6.640797	-1.684758	-0.996307
H	5.678489	-1.355113	1.027367
C	3.655114	-1.341503	1.695207
H	3.195087	-2.331537	1.783320
H	4.087631	-1.078980	2.666482
C	2.495254	-0.374505	1.421494
N	2.755065	0.687587	0.618224
O	1.393178	-0.584588	1.934005
H	3.671092	0.751142	0.192702
C	1.706383	1.619299	0.208453
H	1.211966	2.003634	1.107653
C	0.565011	0.950486	-0.586448
N	0.815122	-0.249500	-1.171594
O	-0.529572	1.518844	-0.657494
H	1.709084	-0.718766	-1.043516
C	-0.255095	-0.977961	-1.833592
H	-0.714139	-0.367587	-2.616939
H	0.167200	-1.873330	-2.300034
C	-1.417577	-1.412598	-0.922689
N	-1.188092	-1.419331	0.415896
O	-2.500082	-1.726371	-1.428001
H	-0.292196	-1.111961	0.785738
C	-2.261755	-1.693820	1.357015
H	-2.776463	-2.621335	1.089360
H	-1.831086	-1.818892	2.354806
C	-3.371965	-0.628223	1.455523
N	-3.147739	0.555794	0.816474
O	-4.391844	-0.887869	2.091675
H	-2.271311	0.714457	0.326786
C	-4.185421	1.561124	0.687789
H	-4.926841	1.398490	1.475110
H	-3.765741	2.564180	0.813893
C	-4.950146	1.572278	-0.650318
N	-4.696652	0.545988	-1.508981
O	-5.743042	2.482011	-0.889193
H	-4.056164	-0.213994	-1.303942
H	-5.222633	0.525772	-2.371564
C	2.297660	2.796751	-0.585738
H	1.453658	3.382737	-0.962879
H	2.830652	2.409442	-1.466371
C	3.222281	3.698429	0.246059
H	4.092758	3.157686	0.636933
H	3.595145	4.529265	-0.362706
H	2.685396	4.124171	1.101630

-1328.1916308

Abu Helix/trans

C	-6.016713	-1.106641	1.467148
C	-4.730826	-1.156675	0.662662
N	-4.861700	-1.045849	-0.702652
C	-3.748358	-1.374344	-1.591674
C	-2.523431	-0.455808	-1.482739
N	-2.729180	0.783221	-0.972162
C	-1.634650	1.718149	-0.706552
C	-2.223956	3.028177	-0.141650
H	-2.690933	2.817826	0.831172
O	-3.624976	-1.275154	1.194853
O	-1.421140	-0.864506	-1.855905
C	-0.556594	1.128004	0.225834
O	0.602851	1.549998	0.165871
N	-0.943837	0.160818	1.100026
C	0.033073	-0.480002	1.966015
C	1.154467	-1.257437	1.254337
O	2.180215	-1.544160	1.880712
N	0.956624	-1.590103	-0.046532
C	2.011546	-2.218627	-0.825287
C	3.248915	-1.348564	-1.124554
O	4.249855	-1.879450	-1.602722
N	3.150954	-0.019811	-0.832783
C	4.300755	0.861582	-0.900017
C	5.008122	1.150082	0.438555
O	5.893465	2.003050	0.486042
N	4.602710	0.421756	1.515641
C	-1.205169	4.167346	0.002020
H	-3.026385	3.347725	-0.821587
H	-6.889082	-0.808753	0.877014
H	-6.198160	-2.101159	1.889663
H	-5.886887	-0.414010	2.303363
H	-5.796631	-1.075850	-1.088139
H	-3.371502	-2.384527	-1.398666
H	-4.105906	-1.336348	-2.626073
H	-3.660855	1.016661	-0.652506
H	-1.104413	1.928306	-1.641825
H	-1.882498	-0.230791	1.062969
H	0.546347	0.260796	2.586519
H	-0.491841	-1.174662	2.629047
H	0.118391	-1.284300	-0.534326
H	2.398431	-3.099314	-0.304279
H	1.594095	-2.544631	-1.782491
H	2.282764	0.358296	-0.463444
H	5.045972	0.409198	-1.560437
H	4.017213	1.832833	-1.317607
H	3.885390	-0.295484	1.474248
H	5.090919	0.577515	2.386608
H	-1.699081	5.063014	0.394769
H	-0.389014	3.896730	0.676632
H	-0.760574	4.421020	-0.966770

-1328.1908037

Nva Helix/-gauche

H	-5.528012	-1.218821	2.490527
C	-5.685847	-1.783553	1.567595
C	-4.441851	-1.677927	0.704900
N	-4.644415	-1.383291	-0.623565
O	-3.307191	-1.831724	1.162414
H	-5.825626	-2.834323	1.844981
H	-6.592562	-1.425956	1.069554
H	-5.596075	-1.390723	-0.966944
C	-3.566792	-1.547365	-1.598001
H	-3.152699	-2.560766	-1.564073
H	-3.975250	-1.379596	-2.600188
C	-2.365492	-0.609698	-1.417539
N	-2.581284	0.544605	-0.738669
O	-1.270627	-0.925936	-1.889605
H	-3.497402	0.698667	-0.337350
C	-1.493461	1.466634	-0.416405
H	-0.980207	1.738900	-1.345435
C	-0.386564	0.829904	0.449760
N	-0.697723	-0.285918	1.159275
O	0.735235	1.347074	0.466668
H	-1.615118	-0.719055	1.078789
C	0.330817	-0.990309	1.907437
H	0.811700	-0.324314	2.630116
H	-0.138196	-1.811296	2.458458
C	1.479360	-1.572894	1.064484
N	1.266527	-1.707611	-0.269699
O	2.538897	-1.885059	1.617887
H	0.390622	-1.400276	-0.684749
C	2.337192	-2.130596	-1.158122
H	2.795873	-3.054001	-0.792300
H	1.914599	-2.328631	-2.147513
C	3.506201	-1.140724	-1.336069
N	3.334328	0.113125	-0.827486
O	4.522844	-1.517883	-1.916177
H	2.458044	0.366567	-0.379061
C	4.424929	1.067855	-0.771846
H	5.171453	0.786504	-1.519866
H	4.066150	2.074774	-1.007469
C	5.162777	1.170762	0.577570
N	4.832157	0.253286	1.528118
O	6.003436	2.053456	0.744006
H	4.153268	-0.487714	1.384741
H	5.340901	0.290355	2.400464
C	-2.034475	2.740309	0.255281
H	-1.167825	3.316353	0.597169
H	-2.604028	2.464690	1.155945
C	-2.895745	3.615427	-0.670115
H	-3.756246	3.043882	-1.045299
C	-3.395768	4.888192	0.028847
H	-3.992898	5.504840	-0.652431
H	-2.556585	5.498597	0.383089
H	-4.020953	4.647500	0.897854
H	-2.302514	3.888954	-1.553029

-1367.5114123

Nva Helix/trans

C	-5.957126	-1.145807	1.530229
C	-4.670568	-1.272633	0.735250
N	-4.796961	-1.257610	-0.634910
C	-3.686243	-1.664176	-1.494363
C	-2.450069	-0.755578	-1.448459
N	-2.640951	0.518898	-1.027978
C	-1.535188	1.457688	-0.829960
C	-2.106917	2.816021	-0.372486
H	-2.554488	2.700913	0.625775
O	-3.567714	-1.368957	1.278033
O	-1.352482	-1.203713	-1.789246
C	-0.468485	0.926000	0.149501
O	0.696023	1.329965	0.065565
N	-0.869238	0.026621	1.087472
C	0.098301	-0.564111	1.998515
C	1.212121	-1.400332	1.343466
O	2.236268	-1.651111	1.987600
N	1.008382	-1.823710	0.070067
C	2.055477	-2.516722	-0.663032
C	3.298920	-1.681476	-1.028989
O	4.292612	-2.253958	-1.473090
N	3.214364	-0.334657	-0.830803
C	4.371393	0.529434	-0.964627
C	5.089859	0.901668	0.347126
O	5.984378	1.746236	0.331051
N	4.683071	0.253920	1.473936
C	-1.085379	3.963798	-0.354887
H	-2.923086	3.080973	-1.061134
H	-6.825698	-0.887429	0.916363
H	-6.148813	-2.101405	2.030729
H	-5.821600	-0.389684	2.308605
H	-5.731350	-1.302483	-1.020244
H	-3.322956	-2.662427	-1.227342
H	-4.041008	-1.696863	-2.529896
H	-3.570495	0.787070	-0.730266
H	-0.999160	1.585639	-1.776734
H	-1.812394	-0.355534	1.074742
H	0.618463	0.211520	2.568665
H	-0.435898	-1.206431	2.705441
H	0.173169	-1.543745	-0.438165
H	2.436756	-3.359620	-0.079224
H	1.632265	-2.909082	-1.592399
H	2.351819	0.076717	-0.484374
H	5.108521	0.027184	-1.597533
H	4.093381	1.472804	-1.445135
H	3.956900	-0.455411	1.485965
H	5.177156	0.464711	2.329871
C	-1.719097	5.290250	0.088417
H	-0.250455	3.708487	0.305316
H	-0.655337	4.075808	-1.358963
H	-0.977287	6.096466	0.085178
H	-2.538393	5.588479	-0.578383
H	-2.125346	5.217266	1.105143

-1367.510551

Nle Helix/-gauche

H	5.122716	-2.243341	-2.456154
C	5.213576	-2.766150	-1.500167
C	3.993965	-2.459188	-0.650761
N	4.234309	-2.130278	0.663294
O	2.848634	-2.487541	-1.106592
H	5.224910	-3.841063	-1.712638
H	6.158250	-2.491463	-1.020296
H	5.178224	-2.240284	1.010407
C	3.146781	-2.107680	1.640076
H	2.604934	-3.059485	1.651836
H	3.575503	-1.947706	2.635043
C	2.076852	-1.031864	1.410692
N	2.437362	0.048504	0.674654
O	0.952126	-1.179551	1.895337
H	3.366727	0.065447	0.274617
C	1.477469	1.085941	0.301952
H	1.016317	1.481395	1.213852
C	0.286521	0.550706	-0.519463
N	0.447825	-0.623566	-1.183312
O	-0.763614	1.200566	-0.548939
H	1.304228	-1.165261	-1.088712
C	-0.670075	-1.220008	-1.896907
H	-1.068855	-0.526949	-2.643679
H	-0.315628	-2.115896	-2.415938
C	-1.875790	-1.615483	-1.025640
N	-1.663859	-1.737196	0.310115
O	-2.974512	-1.800552	-1.558858
H	-0.750720	-1.530200	0.706402
C	-2.767321	-1.988911	1.222774
H	-3.347583	-2.855587	0.892492
H	-2.359421	-2.209537	2.213589
C	-3.793820	-0.849400	1.381871
N	-3.463942	0.355853	0.835705
O	-4.843779	-1.072272	1.982133
H	-2.567112	0.479729	0.373654
C	-4.413757	1.450035	0.771589
H	-5.181246	1.289328	1.534113
H	-3.916853	2.402660	0.980407
C	-5.149203	1.626105	-0.571166
N	-4.981122	0.640217	-1.495419
O	-5.849224	2.621095	-0.754482
H	-4.408836	-0.183991	-1.342371
H	-5.487178	0.732284	-2.365194
C	2.173469	2.230529	-0.453811
H	1.385361	2.896878	-0.820874
H	2.680276	1.823823	-1.342010
C	3.164471	3.035986	0.401820
H	3.962155	2.383552	0.786924
H	2.638837	3.428413	1.283760
C	3.800496	4.200474	-0.373634
H	3.005788	4.860456	-0.747612
H	4.316253	3.807901	-1.261862
C	4.787403	5.016883	0.473509
H	5.611700	4.390661	0.837844
H	4.290455	5.453469	1.348440
H	5.223517	5.838223	-0.106496

-1406.8312458

Nle Helix/trans

H	-5.789027	-0.750789	2.288756
C	-5.900656	-1.527163	1.526571
C	-4.609730	-1.631894	0.735404
N	-4.732854	-1.628887	-0.635071
O	-3.506420	-1.699754	1.281596
H	-6.063751	-2.477066	2.047457
H	-6.775926	-1.307971	0.907042
H	-5.664866	-1.696937	-1.022707
C	-3.610456	-2.014043	-1.489099
H	-3.223921	-3.001471	-1.214588
H	-3.961556	-2.062064	-2.525279
C	-2.396809	-1.075421	-1.445962
N	-2.619703	0.196367	-1.033595
O	-1.287805	-1.498851	-1.781458
H	-3.556414	0.443714	-0.740341
C	-1.538543	1.164120	-0.839234
H	-1.003504	1.299448	-1.785532
C	-0.461264	0.665599	0.146052
N	-0.841385	-0.237952	1.088590
O	0.693204	1.097408	0.061988
H	-1.774814	-0.643220	1.076259
C	0.139226	-0.799935	2.003996
H	0.637807	-0.008916	2.572262
H	-0.379669	-1.453367	2.712146
C	1.275779	-1.609184	1.353802
N	1.082713	-2.046971	0.083581
O	2.306929	-1.827133	1.998706
H	0.240434	-1.792191	-0.426356
C	2.148119	-2.715460	-0.645889
H	2.553329	-3.543318	-0.056719
H	1.735101	-3.125894	-1.572039
C	3.367299	-1.848166	-1.019150
N	3.244867	-0.502953	-0.830470
O	4.376257	-2.395553	-1.460533
H	2.371796	-0.113546	-0.484882
C	4.376293	0.393058	-0.972502
H	5.126452	-0.091434	-1.603941
H	4.069968	1.324924	-1.458245
C	5.086094	0.794590	0.335295
N	4.698279	0.144726	1.467564
O	5.956818	1.663514	0.311731
H	3.994089	-0.586262	1.485134
H	5.188519	0.374169	2.320908
C	-2.146525	2.510038	-0.392072
H	-2.969172	2.746407	-1.083354
H	-2.591846	2.389030	0.606443
C	-1.157411	3.685301	-0.382306
H	-0.734317	3.808609	-1.389059
H	-0.311799	3.456537	0.275500
C	-1.820272	4.998926	0.059880
H	-2.236363	4.873959	1.070130
H	-2.672188	5.222124	-0.599326
C	-0.845575	6.185763	0.053248
H	-0.438021	6.356495	-0.950693
H	0.000841	6.005285	0.726771
H	-1.339893	7.109611	0.375625

-1406.8302459

Tle **Helix**

H	-5.623551	-0.871994	2.368135
C	-5.737964	-1.551424	1.519073
C	-4.452713	-1.553507	0.711826
N	-4.589350	-1.428324	-0.651568
O	-3.342746	-1.645609	1.240781
H	-5.893782	-2.558198	1.922429
H	-6.618614	-1.264308	0.935901
H	-5.524140	-1.477368	-1.035449
C	-3.470169	-1.714078	-1.548146
H	-3.057333	-2.711142	-1.360295
H	-3.835438	-1.687233	-2.580132
C	-2.277337	-0.753140	-1.449369
N	-2.513291	0.463265	-0.903570
O	-1.172313	-1.115451	-1.862962
H	-3.443173	0.647658	-0.552325
C	-1.445833	1.435855	-0.664273
H	-0.914224	1.599172	-1.608542
C	-0.356473	0.871893	0.278562
N	-0.710825	-0.131716	1.124790
O	0.794988	1.317744	0.226792
H	-1.643119	-0.538042	1.093636
C	0.288204	-0.774231	1.964011
H	0.787662	-0.041673	2.605231
H	-0.215107	-1.503063	2.606765
C	1.424453	-1.499034	1.220506
N	1.223257	-1.801045	-0.086911
O	2.464331	-1.772894	1.829296
H	0.371327	-1.507880	-0.558939
C	2.287663	-2.376169	-0.893254
H	2.702804	-3.262496	-0.404470
H	1.870200	-2.682338	-1.856974
C	3.498345	-1.463594	-1.174442
N	3.365685	-0.147125	-0.843223
O	4.510021	-1.951841	-1.674947
H	2.490669	0.195891	-0.455959
C	4.489754	0.767935	-0.892664
H	5.241558	0.358031	-1.573080
H	4.175108	1.743237	-1.277347
C	5.200549	1.035681	0.448414
N	4.823676	0.265182	1.506242
O	6.062461	1.911165	0.514784
H	4.126734	-0.470907	1.448339
H	5.314974	0.408045	2.377684
C	-2.033754	2.809154	-0.196680
C	-0.902232	3.855363	-0.130485
C	-2.698523	2.693365	1.191568
C	-3.071903	3.290454	-1.235409
H	-2.642546	3.315398	-2.244068
H	-0.125471	3.576830	0.584984
H	-1.319074	4.824881	0.167069
H	-0.418742	3.979750	-1.106342
H	-3.970497	2.662673	-1.270633
H	-3.398799	4.306362	-0.986625
H	-3.512155	1.955869	1.215213
H	-3.131770	3.658289	1.477714
H	-1.973922	2.410857	1.962373

-1406.8283522

Tle **Hairpin**

H	5.532059	3.838413	1.112776
C	4.874684	4.082666	0.272108
C	3.820220	2.996444	0.148651
N	2.531785	3.391183	0.256654
O	4.132855	1.809125	-0.036759
H	4.451598	5.080243	0.423804
H	5.486649	4.083797	-0.635516
H	2.263632	4.363504	0.378975
C	1.432894	2.455716	0.153167
H	1.454013	1.924474	-0.806370
H	1.485934	1.688029	0.933212
C	0.115912	3.227695	0.279374
N	-0.985884	2.442491	0.250200
O	0.091510	4.458523	0.393652
H	-0.875925	1.427724	0.146408
C	-2.311063	3.014644	0.314517
H	-2.592678	3.269155	1.347978
H	-2.330775	3.953178	-0.255461
C	-3.331804	2.050031	-0.289656
N	-4.580263	2.093936	0.275695
O	-3.057956	1.271388	-1.201873
H	-4.768376	2.795804	0.979910
C	-5.703537	1.383405	-0.330163
H	-6.629784	1.718518	0.146575
H	-5.772296	1.606069	-1.399771
C	-5.661548	-0.153578	-0.235480
N	-4.769264	-0.679291	0.654554
O	-6.419057	-0.820661	-0.931655
H	-4.186530	-0.036620	1.174286
C	-4.498035	-2.102539	0.751772
H	-4.558793	-2.446933	1.789078
H	-5.267715	-2.624225	0.174257
C	-3.122805	-2.550503	0.224805
N	-2.516619	-1.724891	-0.676172
O	-2.647791	-3.623949	0.593841
H	-2.907691	-0.811185	-0.887262
C	-1.208406	-2.042598	-1.202596
H	-1.091989	-3.130050	-1.210230
H	-1.135356	-1.674951	-2.233921
C	-0.061397	-1.386972	-0.411746
N	1.098844	-2.075805	-0.417454
O	-0.203375	-0.287728	0.147201
H	1.175343	-2.922472	-0.974277
C	2.382517	-1.548555	0.048809
H	2.257924	-0.474361	0.207361
C	3.378382	-1.796281	-1.103984
N	4.250916	-0.789702	-1.344655
O	3.334570	-2.854999	-1.745655
H	4.180301	0.075417	-0.808574
C	5.271873	-0.875601	-2.377718
H	5.232421	-1.873121	-2.819736
H	5.098294	-0.130290	-3.164240
H	6.268048	-0.706301	-1.951491
C	2.837741	-2.184079	1.410331
C	1.817470	-1.786156	2.496690
C	4.221480	-1.622423	1.797255
C	2.910333	-3.721409	1.321321
H	2.101177	-2.233507	3.457084
H	0.808975	-2.133604	2.250858
H	1.776268	-0.698175	2.629730
H	4.486042	-1.955809	2.807777
H	4.229675	-0.525326	1.791916
H	5.007400	-1.966750	1.116824
H	3.262084	-4.129100	2.276725
H	3.592965	-4.051853	0.532688
H	1.924977	-4.158165	1.122096

-1654.20956

Dpr Helix/-gauche

H	-3.502700	-3.732026	1.304000
C	-4.468437	-3.230062	1.206707
C	-4.294057	-1.942056	0.433430
N	-5.021564	-0.857388	0.878119
O	-3.537663	-1.834328	-0.538834
H	-5.125402	-3.889956	0.627379
H	-4.909803	-3.083546	2.197060
H	-5.681884	-1.017113	1.629480
C	-5.267773	0.303524	0.032137
H	-5.595047	0.000790	-0.971884
H	-6.073262	0.902111	0.467713
C	-4.089378	1.236072	-0.222674
N	-2.877328	0.947620	0.309618
O	-4.259467	2.240240	-0.945178
H	-2.824572	0.107576	0.874601
C	-1.635199	1.386474	-0.354627
H	-1.809253	1.360561	-1.439807
C	-0.488895	0.398577	-0.009693
N	-0.745611	-0.911625	-0.252340
O	0.573774	0.830470	0.435495
H	-1.664008	-1.209635	-0.587915
C	0.198455	-1.950016	0.177448
H	0.357905	-1.900117	1.259192
H	-0.243236	-2.922778	-0.059102
C	1.610514	-1.904327	-0.434269
N	1.766456	-1.248272	-1.614666
O	2.535227	-2.442190	0.170104
H	0.952619	-0.864603	-2.074089
C	3.080704	-1.112619	-2.237260
H	3.573559	-2.087482	-2.292817
H	2.954350	-0.741176	-3.258224
C	4.079399	-0.184132	-1.514900
N	3.584473	0.574277	-0.491845
O	5.245282	-0.167027	-1.891317
H	2.610183	0.481322	-0.227858
C	4.453981	1.327680	0.395970
H	5.423759	1.443197	-0.096814
H	4.050206	2.327910	0.582962
C	4.717846	0.697990	1.777607
N	4.322395	-0.597124	1.953577
O	5.247194	1.373332	2.654787
H	3.966551	-1.181303	1.204693
H	4.591987	-1.038300	2.823017
C	-1.189299	2.800838	0.050272
H	-0.281912	3.079343	-0.486288
H	-0.977100	2.845484	1.119199
N	-2.284490	3.811547	-0.269219
H	-2.526581	4.372887	0.551539
H	-1.989061	4.461778	-1.003147
H	-3.169549	3.280198	-0.616945

-1344.5969391

Dpr Helix/trans

H	4.822654	-1.730124	-2.921967
C	5.496658	-1.613423	-2.069562
C	4.716606	-1.110328	-0.875625
N	5.320627	-0.151699	-0.099065
O	3.581320	-1.523590	-0.601949
H	5.893293	-2.606229	-1.825865
H	6.332729	-0.963178	-2.343815
H	6.287519	0.068420	-0.306977
C	4.864425	0.112775	1.265263
H	4.865378	-0.798922	1.872794
H	5.555203	0.823896	1.730751
C	3.456639	0.692055	1.409483
N	2.914656	1.331035	0.304898
O	2.855399	0.615876	2.470723
H	3.400764	1.251485	-0.580693
C	1.524512	1.717829	0.340722
H	1.260019	1.755988	1.406921
C	0.545697	0.698768	-0.291942
N	0.964491	-0.539481	-0.535155
O	-0.638241	1.077227	-0.528419
H	1.947312	-0.804498	-0.380283
C	0.082473	-1.541695	-1.145135
H	-0.280295	-1.188108	-2.114522
H	0.675380	-2.446007	-1.309152
C	-1.182312	-1.915346	-0.350603
N	-1.181347	-1.680675	0.990249
O	-2.147652	-2.374774	-0.955125
H	-0.327665	-1.386843	1.445841
C	-2.370309	-1.944861	1.797918
H	-2.745618	-2.954051	1.604630
H	-2.101076	-1.882388	2.856068
C	-3.566987	-1.001941	1.559279
N	-3.333382	0.099472	0.779021
O	-4.644525	-1.264943	2.076606
H	-2.424284	0.206787	0.344096
C	-4.412034	0.948787	0.304587
H	-5.288121	0.760521	0.932746
H	-4.156352	2.009101	0.406949
C	-4.851756	0.744611	-1.158646
N	-4.371388	-0.359175	-1.802186
O	-5.575330	1.582705	-1.688007
H	-3.862949	-1.100115	-1.331824
H	-4.754719	-0.549575	-2.719194
C	1.340995	3.123536	-0.282347
H	1.636832	3.126125	-1.335990
N	-0.117343	3.518000	-0.239525
H	-0.351895	4.221715	-0.945093
H	-0.386519	3.884531	0.678521
H	-0.631978	2.556746	-0.417912
H	1.944794	3.857769	0.255338

-1344.6036906

Dab Helix/-gauche

H	3.769512	-3.655379	-2.061019
C	4.673651	-3.274908	-1.578860
C	4.296675	-2.201001	-0.580468
N	5.093557	-1.079333	-0.553878
O	3.320587	-2.304271	0.171705
H	5.128266	-4.107773	-1.029691
H	5.380841	-2.931846	-2.340041
H	5.925658	-1.090769	-1.130510
C	5.093588	-0.160643	0.582219
H	5.131527	-0.715032	1.528258
H	5.992005	0.461137	0.533380
C	3.895611	0.781071	0.736088
N	2.879321	0.693084	-0.179557
O	3.854911	1.570611	1.678908
H	2.960324	-0.000185	-0.912674
C	1.557063	1.209370	0.153256
H	1.531084	1.303374	1.247803
C	0.418231	0.227872	-0.229858
N	0.687913	-1.094718	-0.097664
O	-0.676538	0.668949	-0.589455
H	1.614485	-1.422461	0.183584
C	-0.292736	-2.098742	-0.515082
H	-0.542374	-1.975111	-1.573596
H	0.161012	-3.085928	-0.384549
C	-1.646519	-2.091542	0.215239
N	-1.691448	-1.514261	1.446100
O	-2.629530	-2.585270	-0.334636
H	-0.837144	-1.151357	1.845262
C	-2.939667	-1.414488	2.194371
H	-3.435597	-2.388813	2.232647
H	-2.716712	-1.111100	3.221393
C	-3.991780	-0.437436	1.631033
N	-3.583850	0.383814	0.617935
O	-5.118121	-0.434796	2.114995
H	-2.640216	0.298620	0.256551
C	-4.520032	1.200461	-0.133256
H	-5.438852	1.290768	0.453722
H	-4.121373	2.207776	-0.292275
C	-4.920462	0.667853	-1.523224
N	-4.562265	-0.613791	-1.820267
O	-5.520618	1.406914	-2.299235
H	-4.123930	-1.243596	-1.156574
H	-4.900874	-0.991253	-2.695322
C	1.260933	2.599465	-0.447123
H	0.184233	2.778762	-0.346244
H	1.496193	2.584707	-1.519739
C	2.064675	3.684094	0.265479
H	1.810865	3.763284	1.325468
N	1.746746	5.050647	-0.355336
H	2.259050	5.808232	0.112266
H	0.742034	5.256623	-0.286786
H	1.994802	5.066459	-1.351940
H	3.144447	3.547103	0.182162

-1383.9084556

Dab Helix/trans

H	4.672190	-2.303120	-2.794375
C	5.415088	-2.055727	-2.031747
C	4.721659	-1.432468	-0.838772
N	5.338639	-0.345227	-0.265315
O	3.649124	-1.860464	-0.396445
H	5.877651	-2.995056	-1.706795
H	6.191058	-1.416899	-2.464267
H	6.264565	-0.111035	-0.601927
C	4.999894	0.090178	1.089091
H	5.030100	-0.748665	1.793615
H	5.744789	0.822571	1.416139
C	3.622972	0.729390	1.290584
N	2.921397	1.100523	0.162848
O	3.190752	0.906444	2.422665
H	3.303784	0.845878	-0.739688
C	1.525144	1.472938	0.278877
H	1.345852	1.528281	1.361325
C	0.536010	0.418310	-0.274630
N	0.974274	-0.850701	-0.407610
O	-0.622356	0.767992	-0.555551
H	1.948266	-1.103764	-0.218561
C	0.097749	-1.879688	-0.967560
H	-0.235578	-1.596749	-1.970776
H	0.676868	-2.804559	-1.049272
C	-1.195167	-2.184951	-0.191560
N	-1.230548	-1.856696	1.129203
O	-2.150585	-2.683235	-0.785157
H	-0.396497	-1.487153	1.564926
C	-2.436800	-2.052770	1.926837
H	-2.815605	-3.070941	1.797330
H	-2.190434	-1.918825	2.984121
C	-3.623465	-1.123941	1.599131
N	-3.362257	-0.085435	0.747586
O	-4.714292	-1.339021	2.113364
H	-2.441602	-0.016560	0.329732
C	-4.413641	0.744833	0.192994
H	-5.306353	0.619978	0.813809
H	-4.136661	1.804623	0.220975
C	-4.829031	0.443502	-1.260116
N	-4.357476	-0.709852	-1.810227
O	-5.531436	1.253510	-1.861247
H	-3.828277	-1.403542	-1.291751
H	-4.702999	-0.953389	-2.729215
C	1.238777	2.867370	-0.342876
H	2.085575	3.516706	-0.085582
H	1.186546	2.783187	-1.436853
C	-0.063826	3.467121	0.193824
H	-0.025586	3.630564	1.274831
H	-0.913725	2.833239	-0.061390
N	-0.326136	4.840467	-0.431976
H	-0.432085	4.757074	-1.450668
H	0.442998	5.496227	-0.248685
H	-1.191913	5.253972	-0.064061

-1383.9121221

Orn Helix/-gauche

H	3.325092	-4.137639	-2.155995
C	4.247934	-3.880005	-1.629537
C	3.951193	-2.820014	-0.588913
N	4.837481	-1.769463	-0.511423
O	2.962962	-2.873444	0.150809
H	4.592185	-4.782281	-1.111064
H	5.011905	-3.578738	-2.352642
H	5.677323	-1.832119	-1.073020
C	4.885817	-0.894619	0.658470
H	4.841723	-1.483821	1.582387
H	5.838839	-0.357877	0.657575
C	3.772225	0.149242	0.811113
N	2.812776	0.202224	-0.162271
O	3.760421	0.882493	1.798876
H	2.895361	-0.438856	-0.941234
C	1.536630	0.868629	0.073938
H	1.484965	1.029183	1.159416
C	0.322258	-0.025990	-0.283852
N	0.480672	-1.366050	-0.127874
O	-0.739633	0.490211	-0.642835
H	1.378221	-1.766111	0.150420
C	-0.585327	-2.289927	-0.513319
H	-0.838245	-2.167915	-1.571401
H	-0.216566	-3.309772	-0.366330
C	-1.923261	-2.152997	0.232388
N	-1.899404	-1.549685	1.451500
O	-2.954177	-2.574282	-0.290835
H	-1.011716	-1.239573	1.821534
C	-3.119661	-1.328724	2.218348
H	-3.696119	-2.256112	2.285526
H	-2.852446	-1.025672	3.234922
C	-4.096701	-0.276972	1.653541
N	-3.640644	0.485810	0.616975
O	-5.209731	-0.169561	2.157708
H	-2.711861	0.318127	0.245761
C	-4.517615	1.365870	-0.133460
H	-5.413705	1.546294	0.467591
H	-4.035694	2.330885	-0.321283
C	-4.989816	0.842518	-1.504042
N	-4.749045	-0.470503	-1.779539
O	-5.545039	1.613557	-2.282965
H	-4.335171	-1.119195	-1.118166
H	-5.126442	-0.833216	-2.644694
C	1.412019	2.240485	-0.616048
H	0.351288	2.510358	-0.626917
H	1.735245	2.153153	-1.661219
C	2.235793	3.314832	0.116035
H	3.290905	3.013883	0.160190
H	1.883542	3.393950	1.153763
C	2.102169	4.666368	-0.578721
H	1.072416	5.035080	-0.585728
H	2.480752	4.651413	-1.604748
N	2.928208	5.725336	0.162288
H	2.861497	6.649458	-0.281342
H	3.918732	5.454508	0.192907
H	2.607297	5.814962	1.134496

-1423.2289131

Orn Helix/trans

H	-5.820145	-0.334874	2.603618
C	-6.139728	-0.769179	1.652773
C	-4.946028	-0.880188	0.727406
N	-5.145786	-0.511743	-0.583575
O	-3.839675	-1.267207	1.116816
H	-6.508024	-1.780351	1.861388
H	-6.959590	-0.178522	1.232766
H	-6.099114	-0.332078	-0.874307
C	-4.200483	-0.922845	-1.621285
H	-4.079382	-2.011183	-1.646219
H	-4.591606	-0.603177	-2.593309
C	-2.785042	-0.356329	-1.483086
N	-2.645524	0.837902	-0.826746
O	-1.827350	-0.950561	-1.975234
H	-3.444240	1.208527	-0.326427
C	-1.309814	1.362482	-0.587192
H	-0.756083	1.257791	-1.527606
C	-0.466938	0.542614	0.417298
N	-1.042846	-0.476120	1.085857
O	0.733619	0.836569	0.551671
H	-2.029429	-0.714032	0.966721
C	-0.205781	-1.357699	1.892224
H	0.344548	-0.790025	2.647053
H	-0.854140	-2.075185	2.404320
C	0.867562	-2.139837	1.109542
N	0.673816	-2.319562	-0.226544
O	1.863525	-2.553997	1.705495
H	-0.153769	-1.945801	-0.680896
C	1.695754	-2.966379	-1.042955
H	2.005536	-3.909651	-0.584049
H	1.271606	-3.191719	-2.025670
C	2.996877	-2.166806	-1.266122
N	2.974198	-0.864261	-0.852613
O	3.960275	-2.708040	-1.796645
H	2.142304	-0.517970	-0.391583
C	4.151950	-0.025166	-0.802745
H	4.918796	-0.476192	-1.441003
H	3.939223	0.975311	-1.194917
C	4.783985	0.175429	0.587765
N	4.337702	-0.630668	1.588841
O	5.623289	1.062949	0.744615
H	3.674544	-1.386792	1.447149
H	4.810092	-0.562241	2.480449
C	-1.346983	2.860619	-0.201399
H	-2.037714	3.370192	-0.885901
H	-1.743168	2.978077	0.816909
C	0.054244	3.498433	-0.302122
H	0.412921	3.383511	-1.335640
H	0.740718	2.933963	0.337426
C	0.069655	4.977389	0.068989
H	-0.220624	5.155646	1.108558
H	-0.545386	5.601050	-0.586869
N	1.504226	5.500334	-0.067016
H	1.836873	5.399962	-1.034061
H	2.141246	4.952860	0.527635
H	1.588673	6.488788	0.198767

-1423.2369686

Agp Helix/-gauche

H	-2.574233	-4.643887	1.724834
C	-3.601394	-4.363331	1.478232
C	-3.588818	-3.164662	0.554156
N	-4.521360	-2.183411	0.811789
O	-2.786797	-3.045453	-0.379086
H	-4.051615	-5.205458	0.939512
H	-4.168411	-4.195334	2.398751
H	-5.200034	-2.367895	1.540212
C	-4.885985	-1.182958	-0.185732
H	-5.028191	-1.649056	-1.169535
H	-5.837406	-0.725325	0.099327
C	-3.890649	-0.051292	-0.441545
N	-2.696643	-0.075811	0.205761
O	-4.181960	0.838248	-1.257633
H	-2.547672	-0.832589	0.861901
C	-1.511872	0.597561	-0.343543
H	-1.613665	0.591785	-1.437597
C	-0.232348	-0.185676	0.039104
N	-0.275284	-1.530615	-0.145241
O	0.755744	0.419325	0.458091
H	-1.132392	-1.991831	-0.456759
C	0.832328	-2.379317	0.301700
H	1.001807	-2.258386	1.376399
H	0.553379	-3.420855	0.114916
C	2.204478	-2.127709	-0.347044
N	2.222857	-1.505990	-1.556284
O	3.221064	-2.478414	0.248938
H	1.345362	-1.272720	-1.999596
C	3.478733	-1.181635	-2.224934
H	4.126002	-2.062810	-2.259981
H	3.266423	-0.880197	-3.254735
C	4.327924	-0.071200	-1.572981
N	3.740347	0.638563	-0.564164
O	5.464778	0.123802	-1.987734
H	2.801218	0.399301	-0.266157
C	4.493747	1.564290	0.262882
H	5.417492	1.816767	-0.266054
H	3.933471	2.490969	0.424122
C	4.897161	1.049901	1.658646
N	4.730052	-0.282697	1.897817
O	5.330447	1.843382	2.489579
H	4.438388	-0.948152	1.189852
H	5.081572	-0.635079	2.778248
C	-1.356273	2.058026	0.114955
H	-0.472628	2.478688	-0.377960
H	-1.195081	2.087978	1.199277
N	-2.570164	2.797428	-0.249036
H	-3.290769	2.250994	-0.758648
C	-2.769828	4.085709	0.000036
N	-1.820103	4.840000	0.588024
H	-2.011502	5.788730	0.878696
H	-0.922301	4.448991	0.836349
N	-3.955856	4.654902	-0.317315
H	-4.053013	5.660749	-0.341913
H	-4.652541	4.114682	-0.813741

-1493.4806654

Agp Helix/trans

H	-5.683170	-0.491707	2.749120
C	-6.052757	-0.911995	1.810372
C	-4.911910	-1.009561	0.821085
N	-5.190328	-0.665140	-0.480812
O	-3.775557	-1.367788	1.152207
H	-6.410875	-1.926249	2.022795
H	-6.893386	-0.316241	1.441986
H	-6.160779	-0.499281	-0.719334
C	-4.302764	-1.079549	-1.567217
H	-4.191319	-2.168567	-1.603725
H	-4.740427	-0.752044	-2.516648
C	-2.879194	-0.523878	-1.500724
N	-2.702758	0.690974	-0.876016
O	-1.945308	-1.128975	-2.017353
H	-3.460991	1.051006	-0.308624
C	-1.351230	1.181320	-0.700864
H	-0.820065	0.998426	-1.642160
C	-0.495342	0.414398	0.333979
N	-1.031170	-0.591531	1.031783
O	0.710990	0.744203	0.440578
H	-2.020166	-0.844884	0.937751
C	-0.173799	-1.431386	1.867100
H	0.360337	-0.825152	2.603110
H	-0.810195	-2.142935	2.401219
C	0.920202	-2.214576	1.113631
N	0.742144	-2.444811	-0.217139
O	1.920223	-2.579328	1.731061
H	-0.105150	-2.140873	-0.684810
C	1.790988	-3.093671	-1.000794
H	2.119658	-4.011768	-0.505640
H	1.383317	-3.364294	-1.978944
C	3.072261	-2.265752	-1.237668
N	3.008367	-0.950502	-0.869899
O	4.055429	-2.799488	-1.736004
H	2.160548	-0.614618	-0.431940
C	4.160418	-0.073932	-0.832959
H	4.949230	-0.523427	-1.444997
H	3.926011	0.904412	-1.266337
C	4.764827	0.191907	0.559066
N	4.328137	-0.593748	1.582640
O	5.565975	1.115039	0.700328
H	3.715580	-1.392807	1.452205
H	4.797521	-0.487632	2.472518
C	-1.347440	2.708730	-0.446845
H	-1.937995	2.955830	0.448169
N	0.042685	3.144578	-0.278074
H	-1.811109	3.190114	-1.317355
H	0.667603	2.341737	-0.042302
C	0.450640	4.387717	-0.043265
N	-0.380065	5.441118	-0.199372
H	-0.062191	6.384057	-0.022347
H	-1.268293	5.332175	-0.666702
N	1.713672	4.603507	0.375819
H	2.119131	5.529444	0.372405
H	2.338952	3.826220	0.552155

-1493.484844

Agb Helix/-gauche

H	1.851066	-5.156179	-2.117011
C	2.841453	-5.112282	-1.656367
C	2.866386	-4.002145	-0.626070
N	3.980957	-3.194340	-0.621050
O	1.938526	-3.810484	0.167342
H	3.004331	-6.064274	-1.137905
H	3.604013	-5.002571	-2.433462
H	4.745280	-3.458272	-1.230053
C	4.308726	-2.346454	0.523110
H	4.194655	-2.902570	1.461513
H	5.356853	-2.043083	0.447203
C	3.476969	-1.074440	0.723605
N	2.496778	-0.802483	-0.189657
O	3.695108	-0.353002	1.697106
H	2.378192	-1.444482	-0.963198
C	1.422054	0.133716	0.122611
H	1.436959	0.252306	1.214981
C	0.025280	-0.433864	-0.235461
N	-0.144199	-1.773438	-0.085599
O	-0.878432	0.326248	-0.592516
H	0.632625	-2.378529	0.186493
C	-1.397094	-2.413124	-0.486326
H	-1.602536	-2.230508	-1.546025
H	-1.285310	-3.492108	-0.341532
C	-2.671684	-1.962475	0.246713
N	-2.519765	-1.386610	1.469493
O	-3.766321	-2.124553	-0.291336
H	-1.588345	-1.296873	1.850913
C	-3.662893	-0.884805	2.222832
H	-4.443324	-1.649508	2.276812
H	-3.345002	-0.657361	3.244524
C	-4.354633	0.370515	1.653118
N	-3.716778	1.006971	0.626954
O	-5.417477	0.736818	2.143540
H	-2.848442	0.626547	0.266425
C	-4.349624	2.072937	-0.128202
H	-5.185142	2.458465	0.463458
H	-3.650138	2.896849	-0.303130
C	-4.914335	1.681401	-1.507682
N	-4.988462	0.349551	-1.788343
O	-5.259279	2.564585	-2.289059
H	-4.752646	-0.380247	-1.124078
H	-5.432788	0.089267	-2.658612
C	1.603218	1.525636	-0.507854
H	0.656492	2.065538	-0.402036
H	1.808464	1.418675	-1.580364
C	2.737531	2.293197	0.174638
H	2.509041	2.412749	1.241898
N	2.885686	3.622628	-0.452295
H	2.161944	3.914848	-1.099331
H	3.682750	1.747413	0.085577
C	3.790542	4.530493	-0.080052
N	4.645462	4.261199	0.922454
H	5.415813	4.880245	1.134046
H	4.568538	3.405405	1.455485
N	3.871906	5.717279	-0.717026
H	3.281618	5.925108	-1.511047
H	4.435570	6.471116	-0.348368

-1532.7943726

Agb Helix/trans

H	-5.922733	0.489091	2.603102
C	-6.325813	0.091140	1.668242
C	-5.181665	-0.277833	0.747296
N	-5.321763	0.064420	-0.578264
O	-4.162441	-0.846215	1.151853
H	-6.878969	-0.823244	1.912393
H	-7.020859	0.812287	1.227389
H	-6.227599	0.405810	-0.875859
C	-4.478366	-0.559421	-1.597776
H	-4.561063	-1.651369	-1.574311
H	-4.812112	-0.215530	-2.582834
C	-2.981304	-0.260118	-1.485395
N	-2.617548	0.916409	-0.883336
O	-2.154535	-1.042876	-1.949225
H	-3.329280	1.444033	-0.392694
C	-1.206579	1.186348	-0.655044
H	-0.683064	0.936505	-1.585733
C	-0.527830	0.262233	0.382501
N	-1.272047	-0.610451	1.088902
O	0.707641	0.334504	0.502160
H	-2.287041	-0.669839	0.984109
C	-0.591428	-1.599312	1.918478
H	0.062052	-1.111825	2.646383
H	-1.348632	-2.171187	2.463585
C	0.316390	-2.589914	1.160950
N	0.082359	-2.787455	-0.166751
O	1.230633	-3.147974	1.770332
H	-0.670364	-2.293181	-0.635880
C	0.973473	-3.632805	-0.956510
H	1.121656	-4.594264	-0.456705
H	0.508315	-3.824678	-1.927720
C	2.391486	-3.079625	-1.215846
N	2.592040	-1.776730	-0.856999
O	3.244244	-3.799679	-1.721958
H	1.829276	-1.280156	-0.416376
C	3.888395	-1.135965	-0.838796
H	4.570505	-1.728382	-1.457655
H	3.836764	-0.131407	-1.272773
C	4.551108	-0.983949	0.543348
N	3.987795	-1.666944	1.575695
O	5.518862	-0.231287	0.665951
H	3.208671	-2.309231	1.465743
H	4.466579	-1.634647	2.465945
C	-0.964777	2.683783	-0.348046
H	-1.477117	3.272493	-1.120311
H	-1.402855	2.944826	0.625833
C	0.534103	3.017942	-0.346699
H	0.966079	2.780937	-1.327212
H	1.030177	2.398421	0.402859
N	0.770377	4.446388	-0.054105
H	-0.023970	5.066317	0.047590
C	1.994737	4.981760	0.002764
N	3.080531	4.191869	-0.025000
H	4.012421	4.582768	-0.063596
H	3.004969	3.187178	0.073509
N	2.152443	6.320107	0.082375
H	1.371535	6.949481	-0.039829
H	3.050532	6.728145	0.303725

-1532.8006417

Agh Helix/-gauche

H	-0.238956	5.869948	-2.551146
C	-0.207965	6.224393	-1.517563
C	0.193799	5.079131	-0.609643
N	-0.505476	4.956317	0.570730
O	1.089451	4.283860	-0.903990
H	0.566266	6.998055	-1.458909
H	-1.170448	6.671661	-1.250214
H	-1.125778	5.716436	0.820608
C	0.027289	4.143665	1.664521
H	1.045628	4.448816	1.928410
H	-0.607180	4.289056	2.545446
C	0.110850	2.635978	1.399208
N	-0.726235	2.115146	0.451494
O	0.891148	1.944316	2.052016
H	-1.221825	2.762970	-0.148892
C	-0.587866	0.720275	0.048635
H	-0.463102	0.138928	0.969228
C	0.704987	0.378426	-0.730275
N	1.542056	1.381957	-1.079354
O	0.947267	-0.814618	-0.947098
H	1.312318	2.358812	-0.901017
C	2.851148	1.070019	-1.638250
H	2.757387	0.455084	-2.537730
H	3.346205	2.007542	-1.909047
C	3.777299	0.280604	-0.694092
N	3.510031	0.356277	0.637982
O	4.701709	-0.388022	-1.160718
H	2.723577	0.906004	0.967899
C	4.280672	-0.413900	1.603558
H	5.352037	-0.268472	1.436868
H	4.042854	-0.058696	2.610388
C	4.066509	-1.940762	1.581324
N	3.037546	-2.403896	0.811834
O	4.806383	-2.657332	2.249777
H	2.480702	-1.751301	0.269496
C	2.853614	-3.818135	0.547509
H	3.412604	-4.381206	1.300690
H	1.797772	-4.096542	0.628573
C	3.327117	-4.312522	-0.833039
N	4.081318	-3.449968	-1.569194
O	3.011439	-5.439298	-1.211746
H	4.364379	-2.529897	-1.247197
H	4.441515	-3.790298	-2.450264
C	-1.820933	0.191301	-0.702916
H	-1.494250	-0.686328	-1.269908
H	-2.163644	0.938970	-1.433880
C	-2.973859	-0.211522	0.230330
H	-3.274549	0.644429	0.850810
H	-2.615738	-0.991159	0.915871
C	-4.188446	-0.734994	-0.553181
H	-3.885566	-1.603693	-1.154241
H	-4.541933	0.038983	-1.248754
C	-5.341180	-1.138181	0.372208
H	-5.674027	-0.277287	0.965441
H	-5.002635	-1.930329	1.054373
N	-6.479350	-1.632595	-0.431074
H	-6.318714	-1.751179	-1.424939
C	-7.642610	-2.064228	0.061282
N	-7.836565	-2.130987	1.390204
H	-8.749302	-2.329060	1.777049
H	-7.071934	-1.993068	2.036003
N	-8.646130	-2.417205	-0.768321
H	-8.573281	-2.277705	-1.766993
H	-9.452495	-2.923334	-0.427760

-1611.4347845

Agh Helix/trans

H	4.664337	-4.242417	2.538251
C	5.235260	-4.192478	1.607497
C	4.652361	-3.108668	0.722872
N	4.530520	-3.408455	-0.614889
O	4.291578	-2.014980	1.166710
H	6.262496	-3.910831	1.866266
H	5.252409	-5.177449	1.130708
H	4.964575	-4.263032	-0.940972
C	4.323931	-2.344709	-1.597241
H	5.109379	-1.583912	-1.534432
H	4.358062	-2.785758	-2.599300
C	3.001933	-1.580845	-1.468285
N	1.956197	-2.236146	-0.886032
O	2.908742	-0.431306	-1.900060
H	2.126582	-3.141730	-0.466650
C	0.697124	-1.540633	-0.650712
H	0.434969	-1.022200	-1.580427
C	0.798901	-0.395143	0.381651
N	1.932724	-0.254926	1.104885
O	-0.154764	0.390188	0.495075
H	2.722668	-0.892879	1.005346
C	2.083898	0.923279	1.948466
H	1.265839	0.995046	2.670128
H	3.025010	0.835909	2.500365
C	2.083816	2.271017	1.199916
N	2.404052	2.260995	-0.123340
O	1.783428	3.300023	1.810447
H	2.612601	1.385217	-0.594662
C	2.338551	3.486498	-0.911039
H	2.880255	4.290817	-0.404991
H	2.819678	3.310062	-1.877480
C	0.928502	4.050800	-1.186381
N	-0.115696	3.240594	-0.843492
O	0.806177	5.162605	-1.690545
H	0.084598	2.344257	-0.417733
C	-1.496286	3.673190	-0.853719
H	-1.568815	4.571273	-1.475648
H	-2.143222	2.906329	-1.292797
C	-2.103794	4.024963	0.517385
N	-1.239647	4.131133	1.561454
O	-3.323878	4.168839	0.619070
H	-0.233179	4.026108	1.471336
H	-1.620126	4.426694	2.450236
C	-0.442464	-2.528631	-0.306135
H	-0.390974	-3.363937	-1.017939
H	-0.286387	-2.945366	0.699335
C	-1.820840	-1.850740	-0.394796
H	-1.942966	-1.458002	-1.414484
H	-1.827770	-0.980928	0.266331
C	-3.019561	-2.754172	-0.068995
H	-2.939030	-3.140446	0.956577
H	-3.061608	-3.615492	-0.750494
C	-4.300067	-1.921090	-0.206231
H	-4.383340	-1.529047	-1.227403
H	-4.243893	-1.073522	0.490250
N	-5.505980	-2.719843	0.091811
H	-5.370647	-3.671544	0.411722
C	-6.754049	-2.246766	0.049511
N	-6.978460	-0.940124	-0.170253
H	-7.912353	-0.589106	-0.333692
H	-6.223365	-0.267635	-0.151657
N	-7.805003	-3.078130	0.212661
H	-7.682687	-4.081245	0.221160
H	-8.737612	-2.719092	0.364948

-1611.4431252

Taza Helix

H	-5.703297	-0.411657	2.317322
C	-5.848190	-1.169213	1.541581
C	-4.577403	-1.277062	0.717619
N	-4.727630	-1.181187	-0.643457
O	-3.469255	-1.428937	1.238288
H	-6.015985	-2.127163	2.045373
H	-6.733925	-0.917922	0.949708
H	-5.666360	-1.162034	-1.018443
C	-3.626896	-1.493615	-1.549801
H	-3.265423	-2.516785	-1.400167
H	-3.988527	-1.408071	-2.580270
C	-2.388178	-0.599508	-1.397944
N	-2.589781	0.635161	-0.887785
O	-1.278952	-1.037582	-1.722259
H	-3.527529	1.011540	-0.766965
C	-1.509875	1.576601	-0.633830
H	-1.008167	1.852271	-1.570775
C	-0.400867	0.981299	0.257690
N	-0.776472	0.022034	1.143577
O	0.758067	1.401378	0.170692
H	-1.717388	-0.367386	1.113436
C	0.209988	-0.622407	1.996193
H	0.727569	0.115189	2.617051
H	-0.308906	-1.321571	2.659189
C	1.327093	-1.393660	1.270759
N	1.108230	-1.739546	-0.022107
O	2.367676	-1.662533	1.881404
H	0.254614	-1.451592	-0.496648
C	2.153288	-2.368377	-0.813148
H	2.555296	-3.242549	-0.292437
H	1.721226	-2.704632	-1.760275
C	3.379288	-1.492486	-1.140852
N	3.275229	-0.161892	-0.859430
O	4.376624	-2.019484	-1.630976
H	2.410153	0.211923	-0.478221
C	4.415503	0.729068	-0.951260
H	5.158925	0.273850	-1.611921
H	4.119387	1.691307	-1.381025
C	5.131584	1.045613	0.376250
N	4.752241	0.320844	1.464866
O	6.001236	1.915744	0.405105
H	4.044580	-0.407062	1.440228
H	5.245512	0.496335	2.329195
C	-2.107436	2.850277	0.038265
C	-1.109034	4.015998	0.018457
H	-2.335819	2.588409	1.080319
N	-3.409406	3.157420	-0.594564
H	-3.250946	3.586590	-1.507541
H	-0.148894	3.732779	0.458886
H	-1.513196	4.868957	0.576094
H	-0.918692	4.340675	-1.012661
H	-3.897446	3.854901	-0.035052

-1383.5566421

Taza Hairpin/-gauche

H	5.455132	3.466339	1.687091
C	4.959430	3.803576	0.771179
C	3.896487	2.786951	0.391220
N	2.629150	3.249781	0.290000
O	4.181823	1.596486	0.188739
H	4.558848	4.809829	0.927168
H	5.715018	3.834881	-0.020374
H	2.389279	4.225776	0.436183
C	1.525981	2.383712	-0.066172
H	1.644439	1.990613	-1.084961
H	1.471198	1.513705	0.594959
C	0.221721	3.181865	0.013178
N	-0.895093	2.429917	-0.132243
O	0.213601	4.405986	0.185405
H	-0.807893	1.410877	-0.217039
C	-2.207167	3.041852	-0.100999
H	-2.421247	3.469216	0.889343
H	-2.254671	3.873802	-0.817564
C	-3.265904	2.011714	-0.491789
N	-4.430935	2.063784	0.226846
O	-3.083623	1.174883	-1.376607
H	-4.546670	2.800465	0.910448
C	-5.590061	1.259177	-0.150996
H	-6.459226	1.605790	0.416122
H	-5.811274	1.377710	-1.216474
C	-5.461832	-0.260742	0.071148
N	-4.424548	-0.669129	0.860076
O	-6.276907	-1.016579	-0.446160
H	-3.796680	0.039588	1.215599
C	-4.042057	-2.063541	1.006182
H	-3.924190	-2.333760	2.059960
H	-4.846844	-2.671041	0.579488
C	-2.720593	-2.429916	0.307808
N	-2.431180	-1.744117	-0.834573
O	-1.998350	-3.321782	0.756952
H	-2.935940	-0.890587	-1.056449
C	-1.150734	-1.944722	-1.480940
H	-0.997115	-3.010501	-1.678420
H	-1.159945	-1.408688	-2.436200
C	0.007043	-1.383005	-0.637396
N	1.141426	-2.105536	-0.648346
O	-0.116432	-0.309735	-0.020329
H	1.194951	-2.973223	-1.172930
C	2.359145	-1.737007	0.068298
H	2.295517	-0.672884	0.309350
C	3.531795	-2.033508	-0.878579
N	4.475030	-1.065739	-0.967087
O	3.563992	-3.113040	-1.484149
H	4.327220	-0.176815	-0.488882
C	5.664026	-1.213808	-1.792496
H	5.668672	-2.221926	-2.211841
H	5.665527	-0.485976	-2.613745
H	6.570976	-1.065593	-1.194040
C	2.486844	-2.556065	1.401390
H	2.643441	-3.604175	1.113670
N	1.290119	-2.512419	2.242664
C	3.698897	-2.082895	2.213848
H	0.482730	-2.906330	1.759973
H	3.779613	-2.682796	3.125026
H	3.586187	-1.030362	2.506869
H	4.630199	-2.174328	1.646696
H	1.054019	-1.546965	2.469501

-1630.9336257

Taza Hairpin/trans

H	5.682570	3.620563	0.991279
C	5.039218	3.801257	0.124144
C	3.938056	2.756421	0.109526
N	2.667957	3.213444	0.155710
O	4.197715	1.540529	0.061944
H	4.660156	4.826768	0.164114
H	5.652948	3.678268	-0.773994
H	2.440481	4.203518	0.182397
C	1.532072	2.317258	0.137407
H	1.527387	1.706057	-0.773301
H	1.559845	1.619431	0.981629
C	0.243518	3.142924	0.196887
N	-0.881324	2.391368	0.234295
O	0.262245	4.378599	0.206074
H	-0.800102	1.369368	0.196286
C	-2.192028	2.999280	0.240328
H	-2.490834	3.307099	1.254241
H	-2.172073	3.910371	-0.372251
C	-3.223419	2.032434	-0.343925
N	-4.482384	2.132176	0.189863
O	-2.948111	1.206386	-1.212655
H	-4.668352	2.869702	0.857238
C	-5.609833	1.422861	-0.409424
H	-6.537545	1.805044	0.027367
H	-5.646887	1.595643	-1.489757
C	-5.614866	-0.108281	-0.241034
N	-4.756247	-0.617740	0.690664
O	-6.379166	-0.785003	-0.920351
H	-4.165755	0.030156	1.194908
C	-4.541074	-2.043043	0.866350
H	-4.628063	-2.329159	1.919448
H	-5.323316	-2.565459	0.306893
C	-3.179592	-2.574734	0.383298
N	-2.509904	-1.802845	-0.520798
O	-2.771903	-3.661547	0.790618
H	-2.858314	-0.882449	-0.772812
C	-1.209954	-2.196045	-1.011656
H	-1.110867	-3.278614	-0.889206
H	-1.133416	-1.959470	-2.080956
C	-0.049270	-1.474058	-0.304334
N	1.143913	-2.095051	-0.392879
O	-0.200540	-0.375028	0.254748
H	1.244631	-2.975387	-0.892252
C	2.399182	-1.505694	0.058036
H	2.323348	-0.419820	-0.036093
C	3.497810	-2.030444	-0.878192
N	4.570143	-1.208705	-1.012120
O	3.386481	-3.134249	-1.426757
H	4.509175	-0.267123	-0.626895
C	5.703966	-1.545973	-1.860685
H	5.642354	-2.606271	-2.113750
H	5.694640	-0.963049	-2.791002
H	6.645604	-1.350797	-1.334879
C	2.681957	-1.815022	1.575053
H	1.779995	-1.467281	2.096416
N	3.818180	-1.075814	2.130224
C	2.855394	-3.310507	1.858622
H	4.689822	-1.450009	1.757804
H	2.964395	-3.462694	2.936940
H	3.746961	-3.710326	1.360698
H	1.991845	-3.886181	1.511586
H	3.786425	-0.095031	1.851822

-1630.9336385

Gcch Helix

H	-5.828360	0.186103	2.322393
C	-6.000995	-0.609060	1.591501
C	-4.741509	-0.802877	0.768627
N	-4.899750	-0.816230	-0.597948
O	-3.628406	-0.925243	1.285522
H	-6.193608	-1.531760	2.149724
H	-6.882701	-0.367038	0.990016
H	-5.842583	-0.831023	-0.964373
C	-3.820005	-1.269459	-1.471610
H	-3.478733	-2.273005	-1.195485
H	-4.194680	-1.307353	-2.499916
C	-2.560210	-0.394983	-1.462719
N	-2.715274	0.895898	-1.070440
O	-1.475315	-0.869220	-1.804367
H	-3.623326	1.216100	-0.759631
C	-1.562167	1.783287	-0.917654
H	-1.014970	1.839713	-1.866261
C	-2.015591	3.127743	-0.536863
C	-0.496504	1.267432	0.099011
N	-0.925452	0.402459	1.049833
O	0.667843	1.646847	-0.014161
H	-1.884525	0.060432	1.057007
C	0.023112	-0.185326	1.983377
H	0.570699	0.595561	2.519561
H	-0.531993	-0.778429	2.716371
C	1.103851	-1.086076	1.359460
N	0.892492	-1.531157	0.094153
O	2.111746	-1.362265	2.017353
H	0.066795	-1.240724	-0.422095
C	1.912788	-2.290601	-0.611199
H	2.251373	-3.133222	-0.001244
H	1.479267	-2.689237	-1.533089
C	3.195403	-1.522692	-0.989062
N	3.171010	-0.168001	-0.831267
O	4.165241	-2.152265	-1.406919
H	2.325651	0.290847	-0.504913
C	4.367568	0.638284	-0.983368
H	5.078097	0.090509	-1.608656
H	4.131526	1.584366	-1.480530
C	5.108095	1.000163	0.319091
N	4.661844	0.410295	1.462972
O	6.051666	1.788158	0.281248
H	3.901381	-0.261172	1.491229
H	5.170160	0.611463	2.312911
C	-2.387651	4.236610	-0.216659
H	-2.692874	5.222231	0.064867

-1325.6874694

Gcch Hairpin

H	6.108141	3.128567	0.803425
C	5.426935	3.377578	-0.016597
C	4.263365	2.402374	0.006040
N	3.028906	2.936223	0.145998
O	4.440798	1.177971	-0.095913
H	5.116658	4.422470	0.078217
H	5.976839	3.247939	-0.954261
H	2.864731	3.937806	0.188197
C	1.841619	2.110086	0.164659
H	1.780504	1.494293	-0.741120
H	1.848503	1.420555	1.016922
C	0.604042	3.009507	0.237659
N	-0.560280	2.320707	0.293051
O	0.691095	4.241982	0.237927
H	-0.534027	1.297907	0.247611
C	-1.840015	2.989008	0.276413
H	-2.155924	3.289362	1.287552
H	-1.759347	3.910178	-0.315683
C	-2.897046	2.079989	-0.355737
N	-4.175135	2.263404	0.108414
O	-2.628172	1.226668	-1.198798
H	-4.352375	3.024711	0.751121
C	-5.308547	1.623192	-0.553919
H	-6.234010	2.073393	-0.181952
H	-5.265537	1.781204	-1.636340
C	-5.430555	0.099414	-0.368457
N	-4.654013	-0.455737	0.608223
O	-6.208815	-0.532233	-1.075003
H	-4.050948	0.157744	1.139718
C	-4.578265	-1.887785	0.831426
H	-4.758583	-2.135346	1.882658
H	-5.367389	-2.355098	0.234195
C	-3.245941	-2.558068	0.454064
N	-2.427585	-1.866040	-0.392405
O	-2.987426	-3.677226	0.893911
H	-2.668208	-0.924701	-0.689614
C	-1.144361	-2.401983	-0.782560
H	-1.118591	-3.454644	-0.485458
H	-1.024876	-2.349214	-1.873466
C	0.035827	-1.638339	-0.161241
N	1.215260	-2.298224	-0.178320
O	-0.076942	-0.482466	0.266207
H	1.304386	-3.253842	-0.515681
C	2.466801	-1.690118	0.250802
H	2.457712	-0.626708	-0.003430
C	2.692513	-1.808283	1.706513
C	3.612473	-2.402976	-0.522690
N	4.721142	-1.649968	-0.673498
O	3.453988	-3.557291	-0.927098
H	4.692330	-0.670401	-0.387192
C	5.921823	-2.156645	-1.320848
H	5.785214	-3.221110	-1.521028
H	6.106434	-1.637472	-2.269677
H	6.793063	-2.019758	-0.669744
C	2.892258	-1.894884	2.900050
H	3.063473	-1.982028	3.951460

-1573.0686706

Gcn Helix

H	5.696836	2.488684	-0.769929
C	5.826632	1.647464	-1.456737
C	4.602130	0.757166	-1.382001
N	4.830843	-0.589114	-1.211071
O	3.454177	1.204367	-1.450211
H	5.892623	2.058264	-2.470230
H	6.759873	1.125890	-1.223305
H	5.784981	-0.919233	-1.278187
C	3.757975	-1.559588	-1.413613
H	3.308314	-1.458358	-2.407326
H	4.176909	-2.567916	-1.329432
C	2.591026	-1.453709	-0.426109
N	2.858775	-0.867520	0.774170
O	1.478536	-1.889330	-0.720438
H	3.775971	-0.472850	0.939897
C	1.782167	-0.615549	1.724512
H	1.274662	-1.553768	1.977834
C	2.354478	-0.045448	2.955861
C	0.632346	0.306309	1.196846
N	0.933593	1.116689	0.156625
O	-0.470701	0.230652	1.731095
H	1.849001	1.088384	-0.290732
C	-0.105068	1.936144	-0.452222
H	-0.585307	2.568838	0.299982
H	0.359331	2.585806	-1.199901
C	-1.249843	1.164778	-1.133237
N	-1.043886	-0.150186	-1.404641
O	-2.299988	1.756403	-1.397469
H	-0.173146	-0.600353	-1.139481
C	-2.105808	-0.975408	-1.960006
H	-2.537458	-0.496738	-2.843922
H	-1.680789	-1.935853	-2.265792
C	-3.301647	-1.266182	-1.031272
N	-3.164793	-0.912088	0.279236
O	-4.306369	-1.793540	-1.503098
H	-2.296129	-0.499177	0.603110
C	-4.280392	-0.971408	1.205853
H	-5.014291	-1.683230	0.817994
H	-3.946151	-1.324211	2.186534
C	-5.029556	0.353753	1.447929
N	-4.674985	1.411763	0.665962
O	-5.897725	0.408209	2.316628
H	-3.983316	1.358303	-0.074330
H	-5.193627	2.270144	0.790913
N	2.834973	0.413793	3.912495

-1341.7838058

Gcn Hairpin

H	6.062999	3.061757	0.795201
C	5.400914	3.317670	-0.038420
C	4.214143	2.372776	-0.021351
N	2.992740	2.930826	0.127958
O	4.359593	1.143243	-0.134767
H	5.114597	4.370514	0.041413
H	5.961379	3.164258	-0.966054
H	2.848508	3.934899	0.181017
C	1.794655	2.121460	0.156982
H	1.725069	1.500001	-0.743949
H	1.796585	1.439895	1.015596
C	0.563597	3.029305	0.223614
N	-0.601718	2.340861	0.276312
O	0.655031	4.260539	0.220943
H	-0.572607	1.320185	0.219946
C	-1.886018	3.000142	0.242189
H	-2.207074	3.318928	1.245717
H	-1.813532	3.908399	-0.370740
C	-2.928873	2.062862	-0.372995
N	-4.211186	2.236096	0.080571
O	-2.641501	1.194033	-1.194399
H	-4.403909	3.007818	0.706296
C	-5.330707	1.563294	-0.573422
H	-6.265271	1.998270	-0.206099
H	-5.291914	1.709118	-1.657708
C	-5.421302	0.039689	-0.369965
N	-4.642529	-0.488807	0.620039
O	-6.180147	-0.616372	-1.075156
H	-4.059525	0.141700	1.154007
C	-4.553247	-1.915909	0.868217
H	-4.725486	-2.145636	1.924787
H	-5.341650	-2.400977	0.284356
C	-3.219435	-2.584650	0.495071
N	-2.400803	-1.896362	-0.356143
O	-2.956269	-3.699256	0.942452
H	-2.646295	-0.960737	-0.667718
C	-1.120112	-2.439258	-0.742829
H	-1.081371	-3.476675	-0.396354
H	-1.014496	-2.439220	-1.836848
C	0.059622	-1.637751	-0.176350
N	1.255861	-2.277319	-0.196106
O	-0.054573	-0.472122	0.215747
H	1.365683	-3.246486	-0.487193
C	2.489472	-1.617510	0.181213
H	2.494480	-0.581248	-0.168277
C	2.677110	-1.568979	1.652353
C	3.669821	-2.397923	-0.472944
N	4.796126	-1.670427	-0.596922
O	3.506448	-3.570477	-0.812958
H	4.763086	-0.674502	-0.369497
C	6.027507	-2.230861	-1.136138
H	5.892744	-3.305926	-1.268703
H	6.271064	-1.780462	-2.106218
H	6.858791	-2.052595	-0.444829
N	2.831024	-1.519742	2.806518

-1589.1690918

Gcno Helix

H	-5.732997	-0.363301	2.350750
C	-5.862488	-1.154092	1.606456
C	-4.601216	-1.257519	0.771490
N	-4.770047	-1.280419	-0.594045
O	-3.476309	-1.302029	1.276198
H	-5.994721	-2.096772	2.148888
H	-6.761718	-0.956083	1.015034
H	-5.713123	-1.360845	-0.951812
C	-3.672180	-1.669745	-1.475838
H	-3.272520	-2.653473	-1.206566
H	-4.049975	-1.724460	-2.502278
C	-2.465318	-0.725063	-1.470739
N	-2.692727	0.557664	-1.079099
O	-1.354493	-1.130189	-1.814886
H	-3.612996	0.822470	-0.752207
C	-1.581864	1.495214	-0.929156
H	-1.033902	1.567576	-1.876825
C	-2.096367	2.824833	-0.568030
C	-0.490708	1.048895	0.101720
N	-0.872123	0.158567	1.046891
O	0.643260	1.509612	-0.006992
H	-1.807363	-0.246193	1.046225
C	0.106647	-0.372445	1.985334
H	0.608436	0.440560	2.518294
H	-0.416764	-0.991574	2.719896
C	1.235146	-1.216446	1.365822
N	1.051188	-1.673495	0.100039
O	2.252822	-1.439144	2.027501
H	0.211395	-1.432032	-0.417775
C	2.105249	-2.395503	-0.595798
H	2.478911	-3.215197	0.024821
H	1.691142	-2.823822	-1.513222
C	3.353538	-1.577134	-0.982001
N	3.274467	-0.223654	-0.829064
O	4.346092	-2.168138	-1.401449
H	2.413468	0.198309	-0.496117
C	4.436691	0.630981	-0.987622
H	5.166314	0.110782	-1.614349
H	4.160535	1.564936	-1.487053
C	5.166791	1.027176	0.310832
N	4.745261	0.428482	1.459873
O	6.079244	1.849884	0.265293
H	4.015871	-0.275925	1.493125
H	5.249625	0.651942	2.306684
N	-2.198078	3.950191	-0.247900
O	-2.386706	5.105861	0.079684

-1416.9496948

Gcno Hairpin

H	5.888626	3.237417	0.683531
C	5.209075	3.510166	-0.130426
C	4.047533	2.534264	-0.139459
N	2.813314	3.054436	0.040081
O	4.223726	1.314041	-0.299732
H	4.896588	4.550845	-0.003318
H	5.761169	3.410571	-1.070371
H	2.644185	4.051505	0.135746
C	1.636002	2.214751	0.045112
H	1.562333	1.643035	-0.888184
H	1.671719	1.485242	0.862229
C	0.385437	3.086527	0.189024
N	-0.760935	2.367717	0.247322
O	0.447199	4.318693	0.237240
H	-0.709858	1.350157	0.154935
C	-2.060085	2.997347	0.288453
H	-2.344840	3.276309	1.314518
H	-2.034092	3.926368	-0.296508
C	-3.107137	2.057011	-0.314341
N	-4.368899	2.174536	0.208610
O	-2.838267	1.232379	-1.186356
H	-4.549085	2.912484	0.877312
C	-5.503320	1.499570	-0.417253
H	-6.427877	1.888776	0.020111
H	-5.527763	1.697316	-1.493703
C	-5.540461	-0.034080	-0.282955
N	-4.703312	-0.582830	0.646534
O	-6.310886	-0.680129	-0.984667
H	-4.112824	0.040359	1.180913
C	-4.553198	-2.016457	0.816665
H	-4.663212	-2.304047	1.867292
H	-5.353050	-2.500851	0.247967
C	-3.216736	-2.613938	0.344156
N	-2.477264	-1.858355	-0.521839
O	-2.883755	-3.733509	0.728237
H	-2.772888	-0.918511	-0.770937
C	-1.198096	-2.327522	-0.998793
H	-1.120676	-3.391892	-0.756638
H	-1.132686	-2.217052	-2.089977
C	-0.016614	-1.553084	-0.396743
N	1.188248	-2.165721	-0.497355
O	-0.141861	-0.424423	0.091729
H	1.303228	-3.111191	-0.856055
C	2.422743	-1.517324	-0.091280
H	2.398076	-0.459669	-0.369821
C	2.656439	-1.582703	1.367790
C	3.591443	-2.224923	-0.843759
N	4.693974	-1.460650	-0.970945
O	3.445974	-3.380060	-1.246760
H	4.646436	-0.482134	-0.680638
C	5.913997	-1.953992	-1.594597
H	5.804817	-3.025140	-1.774444
H	6.097716	-1.450198	-2.551657
H	6.772254	-1.783004	-0.935097
N	2.882739	-1.599076	2.516151
O	3.110933	-1.620838	3.718222

-1664.3349568

Model Structures 1-5: Cartesian coordinates and total energies (in hartrees, B3LYP/6-31G*).

1

H	9.391462	3.095456	0.459235
C	9.685227	2.365952	-0.299435
C	8.534750	1.407305	-0.534872
N	8.858281	0.077991	-0.634471
O	7.369758	1.790538	-0.625512
H	9.879558	2.917754	-1.226011
H	10.609222	1.869414	0.011377
H	9.838238	-0.169676	-0.687667
C	7.895984	-0.893632	-1.161913
H	7.491884	-0.522648	-2.110563
C	8.578022	-2.247020	-1.390047
H	7.846292	-2.960910	-1.775746
H	8.991081	-2.645862	-0.456660
H	9.386738	-2.155709	-2.123652
C	6.658478	-1.067947	-0.267254
N	6.799192	-0.777936	1.048636
O	5.605264	-1.477991	-0.755031
H	7.683052	-0.402644	1.365570
C	5.678645	-0.861769	1.979222
H	5.260664	-1.872161	1.929742
C	6.148353	-0.574036	3.408452
H	5.295725	-0.638098	4.088410
H	6.575637	0.432622	3.490003
H	6.900973	-1.304337	3.723993
C	4.497788	0.056530	1.606151
N	4.744969	1.116993	0.803315
O	3.378327	-0.201629	2.056197
H	5.670163	1.265299	0.406048
C	3.656474	1.983670	0.361154
H	3.113030	2.336865	1.243251
C	4.204206	3.184261	-0.417771
H	3.370126	3.810207	-0.744049
H	4.769243	2.861250	-1.298151
H	4.868430	3.781818	0.214349
C	2.586693	1.246453	-0.466923
N	2.929978	0.076488	-1.055404
O	1.459578	1.739504	-0.568101
H	3.855731	-0.321879	-0.917692
C	1.936684	-0.706112	-1.782778
H	1.481056	-0.070460	-2.548657
C	2.590724	-1.922067	-2.447361
H	1.825708	-2.499883	-2.971827
H	3.073339	-2.565523	-1.704496
H	3.349984	-1.603545	-3.168612
C	0.749245	-1.148405	-0.906171
N	0.933253	-1.203079	0.434180
O	-0.319893	-1.447308	-1.446354
H	1.808752	-0.894948	0.851830
C	-0.172511	-1.552224	1.318899
H	-0.602950	-2.500003	0.980161
C	0.316630	-1.698822	2.763524
H	-0.533032	-1.940984	3.406653
H	0.779180	-0.772870	3.120364
H	1.057457	-2.501293	2.837876
C	-1.351411	-0.561997	1.247408
N	-1.110878	0.679653	0.764239
O	-2.466882	-0.925538	1.631460
H	-0.192623	0.928150	0.402129
C	-2.203529	1.630679	0.594089
H	-2.744727	1.710880	1.542154
C	-1.664678	3.007827	0.193190
H	-2.504193	3.693073	0.052145
H	-1.090245	2.951916	-0.737047
H	-1.009494	3.405295	0.974891
C	-3.275630	1.159675	-0.407857
N	-2.933432	0.202874	-1.303675
O	-4.400975	1.664881	-0.368944
H	-2.013612	-0.231638	-1.275615
C	-3.928549	-0.337400	-2.222706
H	-4.412503	0.497542	-2.739211
C	-3.268042	-1.262874	-3.249538
H	-4.037147	-1.668305	-3.911773
H	-2.748870	-2.091871	-2.757791
H	-2.536693	-0.711625	-3.849427
C	-5.091853	-1.064101	-1.515840
N	-4.885757	-1.501333	-0.250756
O	-6.153278	-1.229806	-2.123727
H	-4.017701	-1.283779	0.233565
C	-5.958520	-2.128693	0.513587
H	-6.463232	-2.839848	-0.148290
C	-5.393669	-2.871456	1.727606
H	-6.223237	-3.290489	2.301470
H	-4.821320	-2.195789	2.372078
H	-4.730569	-3.682545	1.408941
C	-7.086753	-1.162022	0.948231
N	-6.886683	0.163024	0.723176
O	-8.102188	-1.629745	1.457041
H	-6.006875	0.489245	0.332531
C	-7.924926	1.158652	0.940938
H	-8.713397	0.651793	1.507065
C	-7.394137	2.352353	1.742200
H	-8.169967	3.117811	1.811504
H	-6.516619	2.787055	1.249715
H	-7.102965	2.033564	2.748352
C	-8.594784	1.643382	-0.366996
N	-8.317604	0.936390	-1.491368
O	-9.358885	2.604107	-0.350057
H	-7.669535	0.157176	-1.533363
H	-8.770528	1.234610	-2.343106

-2435.2005621

2

H	12.178375	2.728939	0.364163
C	12.349970	2.001170	-0.433230
C	11.124440	1.123058	-0.575218
N	11.340765	-0.227893	-0.650974
O	9.982940	1.585168	-0.611612
H	12.487478	2.564222	-1.362968
H	13.266060	1.441669	-0.223332
H	12.292787	-0.554149	-0.760910
C	10.270146	-1.133425	-1.075730
H	9.857770	-0.786654	-2.029823
C	10.812012	-2.558304	-1.240319
H	10.002933	-3.223489	-1.552262
H	11.228885	-2.937736	-0.300866
H	11.591322	-2.588794	-2.009662
C	9.072980	-1.125070	-0.114073
N	9.325409	-0.879749	1.191508
O	7.939643	-1.364844	-0.540315
H	10.273378	-0.658336	1.467063
C	8.251959	-0.829673	2.180232
H	7.721886	-1.786623	2.170367
C	8.826115	-0.576052	3.577615
H	8.012193	-0.549735	4.306339
H	9.355222	0.382886	3.621758
H	9.516905	-1.376434	3.863565
C	7.173451	0.219343	1.840137
N	7.571162	1.349193	1.208412
O	6.009968	0.018059	2.195274
H	8.507351	1.414009	0.815619
C	6.593965	2.371456	0.835221
H	5.969515	2.576761	1.707957
C	7.302946	3.654848	0.388211
H	6.558758	4.397438	0.087568
H	7.972871	3.466057	-0.456590
H	7.891397	4.070358	1.212715
C	5.607528	1.879317	-0.243708
N	6.114730	1.075445	-1.212223
O	4.432323	2.248000	-0.224167
H	7.046972	0.695128	-1.099539
C	5.246672	0.474031	-2.221082
H	4.627930	1.264540	-2.651985
C	6.082322	-0.192301	-3.319261
H	5.420380	-0.646861	-4.061867
H	6.728549	-0.972577	-2.903617
H	6.707154	0.551834	-3.824427
C	4.238772	-0.515569	-1.601077
N	4.708033	-1.336325	-0.629709
O	3.071065	-0.541495	-1.993571
H	5.688271	-1.302330	-0.369148
C	3.814391	-2.253631	0.073401

H	3.291977	-2.874075	-0.660368
C	4.611731	-3.139160	1.036344
H	3.935839	-3.834364	1.542313
H	5.117859	-2.532373	1.794840
H	5.360352	-3.723225	0.489844
C	2.689963	-1.500427	0.816558
N	3.052314	-0.388961	1.498133
O	1.532923	-1.925616	0.786036
H	4.031819	-0.124813	1.558633
C	2.061830	0.413348	2.210342
H	1.498104	-0.232546	2.890141
C	2.754580	1.529424	3.000066
H	2.007039	2.112556	3.545475
H	3.299020	2.200733	2.327649
H	3.461119	1.106487	3.721795
C	0.999338	0.994131	1.255306
N	1.446768	1.508569	0.086979
O	-0.190380	0.997045	1.581944
H	2.443647	1.535928	-0.110141
C	0.518459	2.058800	-0.896763
H	-0.113505	2.808605	-0.411633
C	1.292820	2.696067	-2.055606
H	0.589258	3.118959	-2.778493
H	1.912302	1.949936	-2.564109
H	1.941871	3.497790	-1.688559
C	-0.468823	0.993459	-1.417413
N	0.045752	-0.229908	-1.678670
O	-1.654927	1.280326	-1.599400
H	1.040267	-0.400131	-1.556550
C	-0.807225	-1.322700	-2.133030
H	-1.379854	-0.990492	-3.004013
C	0.045590	-2.542502	-2.498166
H	-0.601184	-3.351078	-2.851074
H	0.607690	-2.895848	-1.627710
H	0.754897	-2.289038	-3.292912
C	-1.877663	-1.692310	-1.084649
N	-1.468158	-1.753114	0.203456
O	-3.033824	-1.936772	-1.438928
H	-0.495065	-1.573279	0.436546
C	-2.410533	-2.046255	1.279137
H	-2.941942	-2.973349	1.044528
C	-1.664599	-2.191313	2.609831
H	-2.376502	-2.429304	3.405615
H	-1.147742	-1.261155	2.867302
H	-0.924917	-2.996495	2.548516
C	-3.520219	-0.978474	1.383956
N	-3.128889	0.311379	1.256972
O	-4.689248	-1.310181	1.592245
H	-2.145506	0.538530	1.135054
C	-4.104826	1.393930	1.295348
H	-4.697128	1.314111	2.212160
C	-3.393296	2.750356	1.245510
H	-4.132958	3.555201	1.288817
H	-2.817484	2.850228	0.319776
H	-2.710172	2.855397	2.094975
C	-5.141036	1.275063	0.159583
N	-4.671178	0.940375	-1.063321
O	-6.331748	1.507885	0.381685
H	-3.673567	0.804003	-1.205532
C	-5.578604	0.781508	-2.194905
H	-6.231788	1.657755	-2.249808
C	-4.778482	0.634884	-3.493875
H	-5.466844	0.530951	-4.337567
H	-4.137062	-0.251912	-3.452446
H	-4.147513	1.514585	-3.658775
C	-6.547580	-0.408380	-2.025122
N	-6.057607	-1.512621	-1.416936
O	-7.697258	-0.339467	-2.465129
H	-5.078224	-1.554310	-1.149373
C	-6.899240	-2.683958	-1.204878
H	-7.418384	-2.920162	-2.138520
C	-6.041170	-3.874653	-0.762506
H	-6.682449	-4.748540	-0.617790
H	-5.532462	-3.650146	0.181430
H	-5.283868	-4.107938	-1.518407
C	-8.034965	-2.449593	-0.183827
N	-7.773731	-1.556015	0.810907
O	-9.074116	-3.094593	-0.270869
H	-6.838610	-1.169240	0.890687
C	-8.738126	-1.294266	1.867989
H	-9.408887	-2.155710	1.911481
C	-8.027149	-1.115215	3.216927
H	-8.765916	-0.905108	3.995330
H	-7.316051	-0.281611	3.175317
H	-7.472772	-2.021834	3.479967
C	-9.668396	-0.085857	1.618804
N	-9.215580	0.876142	0.765105
O	-10.739163	-0.020368	2.217904
H	-8.237792	0.855692	0.487201
C	-9.916856	2.148221	0.637659
H	-10.945806	1.947049	0.953975
C	-9.321116	3.226955	1.554730
H	-9.836227	4.176397	1.391471
H	-8.254483	3.361635	1.343571
H	-9.433982	2.924759	2.600881
C	-10.005852	2.666305	-0.810092
N	-9.659841	1.804818	-1.797747
O	-10.412943	3.804878	-1.032469
H	-9.212174	0.907914	-1.651260
H	-9.687627	2.159679	-2.742898

-3671.8674824

3

H	8.392294	-2.219067	-0.715631
C	8.608350	-1.197183	-0.389072
C	7.344759	-0.593784	0.198845
N	6.918651	0.577148	-0.357752
O	6.739681	-1.131247	1.135546
H	9.363304	-1.257376	0.401495
H	9.015060	-0.626310	-1.229716
H	7.409050	0.952949	-1.158893
C	5.772634	1.334319	0.159760
H	5.780017	1.202307	1.248272
C	5.910195	2.812113	-0.198631
H	5.077655	3.382440	0.221810
H	5.883177	2.955581	-1.283892
H	6.847743	3.212443	0.201815
C	4.438522	0.748038	-0.368049
N	4.105081	-0.451053	0.168723
O	3.740527	1.343002	-1.201753
H	4.791420	-0.891934	0.785066
C	2.974403	-1.249830	-0.320134
H	2.892996	-1.068379	-1.400153
C	3.223393	-2.731873	-0.049102
H	2.393054	-3.330946	-0.431939
H	3.298454	-2.924492	1.025485
H	4.148645	-3.054282	-0.538318
C	1.640598	-0.778665	0.314544
N	1.243076	0.458440	-0.073983
O	0.989208	-1.490142	1.091916
H	1.870927	0.984140	-0.685900
C	0.088456	1.140151	0.520479
H	0.035640	0.827848	1.571582
C	0.263405	2.654186	0.426055
H	-0.584460	3.162489	0.893000
H	0.303638	2.977401	-0.618624
H	1.183896	2.959120	0.935287
C	-1.231343	0.685463	-0.153495
N	-1.582555	-0.596781	0.115686
O	-1.908442	1.443030	-0.862163
H	-0.927269	-1.157399	0.664158
C	-2.710428	-1.260856	-0.546773
H	-2.776100	-0.846932	-1.561454
C	-2.474737	-2.768398	-0.604084
H	-3.302134	-3.260494	-1.122398
H	-2.420430	-3.192882	0.403159
H	-1.542874	-2.984238	-1.137807
C	-4.048516	-0.928455	0.163369
N	-4.447162	0.358964	0.022067
O	-4.695119	-1.780983	0.789002
H	-3.820586	0.992485	-0.477363
C	-5.613647	0.910866	0.720971
H	-5.680384	0.393535	1.686260
C	-5.437626	2.412503	0.932578
H	-6.292347	2.817701	1.481303
H	-5.389753	2.935490	-0.027469
H	-4.522570	2.611809	1.501181
C	-6.921307	0.606740	-0.062654
N	-7.300844	-0.701052	0.014765
O	-7.545174	1.478114	-0.668438
H	-6.636850	-1.345536	0.442628
C	-8.441531	-1.223821	-0.718535

H	-9.019243	-0.377718	-1.097041
H	-8.127114	-1.842922	-1.569854
H	-9.077519	-1.829765	-0.061896

-1485.1776449

4

H	-3.437657	-4.239510	-0.058932
N	-4.440524	-4.271273	-0.241173
C	-5.171879	-3.159347	0.004808
O	-6.361475	-3.017989	-0.286861
C	-4.415589	-2.046317	0.744081
N	-4.952216	-0.777391	0.267989
H	-5.926191	-0.803154	-0.015742
C	-4.195558	0.317458	0.065475
O	-2.990787	0.358858	0.357322
C	-4.902869	1.500965	-0.598654
N	-4.355644	2.741058	-0.083341
H	-3.354207	2.797266	0.090947
C	-5.152143	3.838272	0.074215
O	-6.353290	3.829917	-0.189624
C	-4.454743	5.074680	0.621036
C	-5.006179	-5.440134	-0.889125
C	-4.582548	-2.200134	2.265662
C	-4.773219	1.397321	-2.132703
H	-6.084394	-5.293708	-0.967700
H	-4.593656	-5.577951	-1.896799
H	-4.804066	-6.344003	-0.302914
H	-3.355011	-2.084297	0.496159
H	-5.966248	1.498682	-0.340692
H	-4.881409	5.310606	1.602198
H	-3.373541	4.950386	0.714867
H	-4.675862	5.920059	-0.038153
H	-4.075802	-1.376281	2.777441
H	-4.142111	-3.144494	2.602289
H	-5.642696	-2.189302	2.540139
H	-5.290481	2.244515	-2.591459
H	-5.220104	0.469296	-2.507571
H	-3.719433	1.420336	-2.431692
O	-1.627347	-3.603744	0.297038
C	-0.424315	-3.596622	-0.003293
N	0.360271	-2.506891	0.154252
C	-0.152542	-1.255006	0.698338
C	0.543732	-0.080964	0.001250
O	1.749965	-0.138098	-0.294335
N	-0.223016	1.003410	-0.208511
C	0.291136	2.241297	-0.787809
C	-0.261414	3.428240	0.015067
O	-1.460922	3.500199	0.308137
N	0.631101	4.384326	0.330758
C	0.226355	5.593074	1.030204
C	0.245181	-4.826001	-0.589597
C	0.059932	-1.158144	2.221279
C	-0.112319	2.379942	-2.265885
H	0.031148	-5.682394	0.057575
H	1.324491	-4.710667	-0.705464
H	-0.205688	-5.036282	-1.565955
H	1.357285	-2.573582	-0.040857
H	-1.222442	-1.215839	0.493678
H	-0.429164	-2.005280	2.710920
H	-0.375672	-0.232121	2.610808
H	1.127538	-1.173954	2.467902
H	-1.223355	0.961505	0.006160
H	1.378729	2.213494	-0.711215
H	0.303844	3.299564	-2.691810
H	-1.202177	2.416662	-2.362956
H	0.266751	1.527256	-2.837279
H	1.614577	4.262577	0.093286
H	-0.519716	6.150226	0.453334
H	-0.210863	5.355714	2.006108
H	1.108979	6.219790	1.173386
O	3.428045	3.973238	-0.322828
C	4.614210	3.804912	-0.008825
N	5.224516	2.606802	-0.112297
C	4.525715	1.403449	-0.536877
C	5.250045	0.213043	0.103461
O	6.447846	0.276307	0.393203
N	4.480339	-0.887320	0.273428
C	5.020032	-2.129297	0.802411
C	4.468926	-3.288035	-0.036920
O	3.259952	-3.413537	-0.246712
N	5.380123	-4.178853	-0.492867
C	5.000671	-5.336145	-1.291300
C	5.463464	4.952798	0.506665
C	4.492475	1.259476	-2.069469
C	4.654862	-2.337055	2.282522
H	5.463309	5.755159	-0.237578
H	6.496579	4.666827	0.723602
H	5.005929	5.352003	1.417599
H	6.185383	2.465556	0.179552
H	3.498895	1.448255	-0.171078
H	4.028369	2.148260	-2.506334
H	3.901373	0.383834	-2.354008
H	5.506086	1.154508	-2.471336
H	3.477007	-0.831918	0.081661
H	6.107248	-2.056970	0.705990
H	5.092247	-3.265948	2.666539
H	3.568369	-2.393336	2.402236
H	5.036015	-1.500723	2.875362
H	6.360736	-3.976318	-0.363979
H	4.696124	-5.044173	-2.302973
H	5.852811	-6.015917	-1.355243
H	4.163078	-5.852856	-0.816519

-2229.6140496

5

O	-6.220952	2.307697	0.821213
C	-5.211322	2.980375	0.582563
N	-4.373554	2.704387	-0.452376
C	-4.620045	1.569805	-1.340477
C	-4.079735	0.281655	-0.681366
O	-2.965532	-0.184788	-0.964893
N	-4.905944	-0.247799	0.242342
C	-4.566023	-1.414317	1.059351
C	-5.062552	-2.698025	0.350383
O	-6.234271	-3.059408	0.431506
N	-4.116415	-3.363342	-0.350191
C	-4.441784	-4.557712	-1.108296
C	-4.841205	4.196690	1.412657
C	-4.009401	1.812453	-2.716722
C	-5.203285	-1.289220	2.441889
H	-4.874726	3.926049	2.472192
H	-5.595039	4.975310	1.251857
H	-3.854392	4.593302	1.160405
H	-3.463699	3.165170	-0.500194
H	-5.706987	1.475634	-1.425722
H	-4.445225	2.712106	-3.161885
H	-4.206259	0.961808	-3.376216
H	-2.926174	1.941553	-2.651982
H	-5.736752	0.293918	0.478246
H	-3.474749	-1.437384	1.139567
H	-4.878172	-2.114204	3.083982
H	-6.292136	-1.345308	2.356109
H	-4.922342	-0.342532	2.914124
H	-3.175537	-2.990691	-0.441452
H	-5.012295	-5.254902	-0.487311
H	-5.046913	-4.326039	-1.994246
H	-3.506833	-5.024724	-1.424740
O	-1.751758	3.954505	-0.264457
C	-0.543268	3.947831	0.009584
N	0.252474	2.867987	-0.170394
C	-0.247268	1.616174	-0.717063
C	0.432762	0.444602	-0.000025
O	1.579056	0.576793	0.461451
N	-0.260952	-0.707392	0.038440
C	0.294127	-1.930113	0.610907
C	-0.082873	-3.120921	-0.280980
O	-1.223114	-3.249340	-0.738022
N	0.897436	-4.021804	-0.488615
C	0.646209	-5.242436	-1.238236
C	0.128218	5.176706	0.594248
C	-0.004084	1.509489	-2.236001
C	-0.207734	-2.170929	2.045532
H	-0.148747	6.044861	-0.011262
H	1.215553	5.087730	0.641598

H	-0.268321	5.344962	1.602024
H	1.248426	2.939280	0.028677
H	-1.321662	1.578442	-0.535840
H	-0.455155	2.371538	-2.736319
H	-0.458673	0.597031	-2.633579
H	1.068388	1.502131	-2.460893
H	-1.220118	-0.729472	-0.319996
H	1.378513	-1.814515	0.627353
H	0.261490	-3.065757	2.468796
H	-1.293082	-2.313502	2.055462
H	0.040013	-1.312234	2.677169
H	1.820524	-3.869196	-0.085592
H	0.318352	-5.013870	-2.257757
H	1.570744	-5.822008	-1.278191
H	-0.134549	-5.844274	-0.760258
O	3.168581	3.813625	0.150076
C	4.399602	3.773015	0.188043
N	5.119764	2.705788	-0.250647
C	4.507127	1.492258	-0.785895
C	5.095559	0.292423	-0.028046
O	6.275599	-0.047234	-0.196822
N	4.252276	-0.329868	0.823055
C	4.669734	-1.502032	1.595229
C	4.492016	-2.750221	0.703486
O	3.498610	-3.481866	0.785072
N	5.498826	-2.939339	-0.178805
C	5.459993	-3.977339	-1.195707
C	5.208280	4.937106	0.733785
C	4.755825	1.348609	-2.291532
C	3.889481	-1.606813	2.900750
H	4.945506	5.840837	0.175803
H	6.290350	4.785416	0.679282
H	4.924783	5.103726	1.777783
H	6.129130	2.719978	-0.186192
H	3.439629	1.575065	-0.580367
H	4.370247	2.225460	-2.820429
H	4.248739	0.458123	-2.680559
H	5.825956	1.245840	-2.495756
H	3.263358	-0.065807	0.827767
H	5.736343	-1.372790	1.802647
H	4.231875	-2.471332	3.477302
H	2.819956	-1.734232	2.715595
H	4.040198	-0.703241	3.498923
H	6.168555	-2.177750	-0.270061
H	4.932013	-4.843207	-0.792413
H	4.941814	-3.644389	-2.104691
H	6.480836	-4.266783	-1.457772

-2229.6132932

Model Structures 6-8: Cartesian coordinates and total energies (in hartrees, B3LYP/6-31G*).

6a

H	6.310346	-0.389755	-0.191789
N	6.437325	-1.376201	-0.416259
C	5.406123	-2.222309	-0.197230
O	5.380532	-3.408532	-0.534649
C	4.226493	-1.609011	0.572992
N	3.034194	-2.341519	0.163804
H	3.215547	-3.284682	-0.167167
C	1.813294	-1.783630	0.079412
O	1.576198	-0.617993	0.424097
C	0.717571	-2.672386	-0.523799
N	-0.567519	-2.191614	-0.042660
H	-0.641171	-1.204032	0.200962
C	-1.658663	-2.986744	-0.017663
O	-1.666772	-4.159911	-0.402490
C	-2.920405	-2.344704	0.577599
N	-4.056559	-3.060934	0.014676
H	-3.836755	-3.990260	-0.328833
C	-5.277523	-2.514471	-0.148764
O	-5.547276	-1.361655	0.219520
C	-6.323483	-3.394006	-0.810869
C	7.656724	-1.797327	-1.082528
C	4.460167	-1.696450	2.091103
C	0.801157	-2.658140	-2.063926
C	-2.895973	-2.414048	2.115252
H	7.629952	-2.883125	-1.183661
H	7.741074	-1.351261	-2.081599
H	8.535506	-1.508119	-0.495437
H	4.108117	-0.561293	0.295438
H	0.839154	-3.704260	-0.177422
H	-2.973986	-1.297624	0.276009
H	-7.186164	-3.482728	-0.143313
H	-6.668366	-2.906153	-1.728332
H	-5.957948	-4.394903	-1.057089
H	3.588019	-1.300923	2.620209
H	5.337008	-1.105345	2.373629
H	4.620540	-2.735447	2.398010
H	0.004089	-3.285981	-2.470277
H	1.764835	-3.047433	-2.409933
H	0.681154	-1.637473	-2.442394
H	-3.825817	-1.997907	2.513501
H	-2.056991	-1.831499	2.508174
H	-2.796843	-3.450895	2.453981
O	5.547187	1.361582	0.219503
C	5.277495	2.514396	-0.148826
N	4.056560	3.060933	0.014591
C	2.920389	2.344810	0.577616
C	1.658654	2.986858	-0.017651
O	1.666777	4.160014	-0.402510
N	0.567505	2.191735	-0.042655
C	-0.717594	2.672502	-0.523768
C	-1.813290	1.783665	0.079379
O	-1.576136	0.618028	0.424035
N	-3.034216	2.341487	0.163766
C	-4.226480	1.608948	0.572995
C	-5.406153	2.222190	-0.197211
O	-5.380592	3.408401	-0.534673
N	-6.437313	1.376029	-0.416228
C	-7.656697	1.797077	-1.082580
C	6.323524	3.393869	-0.810904
C	2.896016	2.414281	2.115264
C	-0.801175	2.658353	-2.063899
C	-4.460126	1.696420	2.091108
H	7.185742	3.483443	-0.142855
H	5.957753	4.394441	-1.058090
H	6.669204	2.905465	-1.727768
H	3.836808	3.990260	-0.328946
H	2.973914	1.297702	0.276115
H	3.825841	1.998096	2.513509
H	2.057003	1.831827	2.508261
H	2.796979	3.451162	2.453913
H	0.641126	1.204170	0.201032
H	-0.839217	3.704351	-0.177328
H	-1.764869	3.047622	-2.409886
H	-0.681119	1.637715	-2.442431
H	-0.004133	3.286257	-2.470206
H	-3.215598	3.284672	-0.167127
H	-4.108060	0.561231	0.295461
H	-5.336951	1.105309	2.373668
H	-4.620516	2.735424	2.397984
H	-3.587961	1.300928	2.620212
H	-6.310265	0.389576	-0.191802
H	-7.629910	2.882860	-1.183863
H	-7.741031	1.350865	-2.081587
H	-8.535496	1.507964	-0.495466

-1981.0730242

6b

O	4.350566	1.195587	-0.454294
C	4.613565	2.360285	-0.157991
N	3.892377	3.056994	0.766138
C	2.668068	2.525397	1.368954
C	1.453929	2.941728	0.511303
O	0.867920	4.009981	0.715358
N	1.110566	2.081007	-0.474344
C	0.014212	2.400800	-1.391787
C	-1.291493	1.846961	-0.784917
O	-1.699380	0.700571	-1.000439
N	-1.933837	2.724410	0.019802
C	-3.102376	2.364147	0.830194
C	-4.395177	2.606448	0.010258
O	-4.971806	3.691276	0.025402
N	-4.803581	1.540967	-0.715230
C	-5.974321	1.601735	-1.570702
C	5.778717	3.101557	-0.789350
C	2.517304	3.017602	2.806304
C	0.292264	1.853346	-2.788528
C	-3.120420	3.185559	2.116177
H	5.583456	3.221115	-1.860169
H	6.680362	2.490970	-0.687180
H	5.962252	4.086736	-0.350396
H	4.063289	4.048160	0.873864
H	2.773651	1.438469	1.343464
H	3.404574	2.759023	3.392131
H	1.641850	2.557101	3.275039
H	2.366116	4.101012	2.827364
H	1.575861	1.179319	-0.585731
H	-0.053955	3.491404	-1.421838
H	-0.564886	2.031631	-3.445363
H	0.491042	0.781331	-2.748484
H	1.168408	2.353598	-3.213479
H	-1.457149	3.595055	0.232465
H	-3.014751	1.296119	1.056053
H	-3.934768	2.850882	2.765961
H	-3.298593	4.239526	1.884743
H	-2.173826	3.081536	2.656688
H	-4.266426	0.680228	-0.707205
H	-6.778428	2.140030	-1.061483
H	-5.765054	2.121415	-2.514900
H	-6.290814	0.579515	-1.788776
O	4.888476	-3.722021	0.324046
C	4.397801	-2.613421	0.124588
C	3.169080	-2.124607	0.932099
N	4.819669	-1.742984	-0.825334
H	4.400982	-0.819392	-0.870822
C	5.939013	-2.033504	-1.700797
H	6.343459	-3.006591	-1.418310
H	5.622553	-2.067377	-2.750695
H	6.720276	-1.271141	-1.597577
H	3.151021	-1.030561	0.924169
C	3.210727	-2.658118	2.360779
H	2.305870	-2.372988	2.907272
H	4.081535	-2.256575	2.888239
H	3.306317	-3.747754	2.353006
N	1.945388	-2.571217	0.259144
H	1.504299	-3.424985	0.585347
C	1.29888	-1.854747	-0.686883
O	1.678355	-0.750460	-1.089520
C	0.01755	-2.532499	-1.210641
H	0.105594	-3.612118	-1.067809
C	-0.250045	-2.218206	-2.681176
H	-1.113845	-2.791295	-3.032342
H	0.618812	-2.482904	-3.291790

H	-0.459565	-1.155511	-2.818730
N	-1.114726	-2.103260	-0.383150
H	-1.554406	-1.209849	-0.604798
C	-1.461796	-2.801672	0.722131
O	-0.858463	-3.807649	1.109108
C	-2.729643	-2.306262	1.450216
H	-2.882410	-1.246698	1.230071
C	-2.627366	-2.538508	2.955344
H	-3.552575	-2.231619	3.452260
H	-1.796650	-1.960597	3.372893
H	-2.429818	-3.593708	3.166978
N	-3.896351	-2.997827	0.897360
H	-4.002544	-3.971686	1.150348
C	-4.613384	-2.521372	-0.161871
O	-4.425945	-1.407489	-0.647268
C	-5.690801	-3.448793	-0.698278
H	-5.805806	-4.371008	-0.120978
H	-6.643831	-2.911970	-0.706830
H	-5.450816	-3.705973	-1.735080

-1981.0468434

6c

O	5.041614	-4.350435	-0.380431
C	5.208841	-3.133555	-0.393006
N	4.287993	-2.250119	0.097047
C	3.023700	-2.702758	0.641360
C	1.869852	-1.961135	-0.044335
O	1.972084	-0.770829	-0.373431
N	0.734958	-2.666387	-0.221083
C	-0.495260	-2.082665	-0.741583
C	-1.664497	-2.763281	-0.019324
O	-1.593524	-3.944270	0.328394
N	-2.761194	-1.985125	0.150482
C	-3.963174	-2.500947	0.788433
C	-5.181204	-1.867289	0.105925
O	-5.278458	-0.646352	-0.031367
N	-6.150227	-2.731427	-0.283766
C	-7.381168	-2.300352	-0.928908
C	6.471679	-2.500230	-0.957125
C	2.942359	-2.490647	2.165869
C	-0.619378	-2.271109	-2.264102
C	-3.993373	-2.203415	2.297950
H	7.335325	-2.897758	-0.414046
H	6.470201	-1.409379	-0.893972
H	6.578643	-2.807088	-2.002699
H	4.459056	-1.249529	0.041689
H	2.969705	-3.772983	0.421718
H	3.761458	-3.027224	2.653325
H	1.992368	-2.861595	2.567304
H	3.026416	-1.426022	2.411612
H	0.666608	-3.634169	0.074718
H	-0.491162	-1.016347	-0.515827
H	-1.530929	-1.790441	-2.633999
H	-0.651694	-3.335345	-2.520721
H	0.241634	-1.813944	-2.760515
H	-2.707286	-0.983650	-0.046861
H	-3.950206	-3.584837	0.640338
H	-4.893023	-2.623887	2.762133
H	-3.988064	-1.123020	2.472286
H	-3.114842	-2.648103	2.773791
H	-5.961173	-3.721395	-0.219511
H	-7.612105	-1.288820	-0.592380
H	-7.286239	-2.291562	-2.021722
H	-8.196631	-2.972006	-0.647267
O	-1.972030	0.770853	-0.373532
C	-1.869800	1.961174	-0.044493
N	-0.734888	2.666409	-0.221207
C	0.495351	2.082675	-0.741659
C	1.664573	2.763277	-0.019360
O	1.593621	3.944286	0.328293
N	2.761222	1.985078	0.150565
C	3.963188	2.500858	0.788585
C	5.181239	1.867243	0.106068
O	5.278618	0.646298	-0.031073
N	6.150138	2.731416	-0.283850
C	7.381038	2.300328	-0.929068
C	-3.023663	2.702840	0.641120
N	-4.287934	2.250134	0.096806
H	-4.458912	1.249534	0.041365
C	-5.208994	3.133561	-0.392856
O	-5.041935	4.350461	-0.379987
C	-6.471829	2.500210	-0.956954
C	-2.942356	2.490872	2.165654
H	-0.666560	3.634205	0.074554
C	0.619530	2.271140	-2.264172
H	-3.761475	3.027491	2.653030
H	-1.992381	2.861869	2.567082
H	-3.026411	1.426269	2.411493
H	2.707271	0.983596	-0.046741
C	3.993341	2.203209	2.298076
H	-6.470249	1.409350	-0.893974
H	-6.578920	2.807218	-2.002472
H	-7.335459	2.897578	-0.413734
H	5.960994	3.721376	-0.219756
H	7.285804	2.290737	-2.021851
H	8.196320	2.972492	-0.648134
H	7.612474	1.289116	-0.591914
H	3.988038	1.122800	2.472326
H	4.892976	2.623649	2.762318
H	3.114794	2.647853	2.773929
H	-0.241472	1.814001	-2.760626
H	1.531085	1.790459	-2.634041
H	0.651880	3.335379	-2.520770
H	3.950221	3.584757	0.640569
H	0.491236	1.016353	-0.515917
H	-2.969676	3.773050	0.421401

-1981.0559657

6d

O	-4.843481	2.858094	-0.965492
C	-5.180899	1.726385	-0.595945
N	-4.660494	1.127850	0.506012
C	-3.654883	1.795069	1.335461
C	-2.261017	1.592889	0.704485
O	-1.445750	0.765205	1.126104
N	-2.028942	2.387367	-0.370399
C	-0.891842	2.180243	-1.272207
C	0.381745	2.872660	-0.730207
O	0.855064	3.881909	-1.267969
N	0.933025	2.280413	0.351288
C	2.054911	2.870909	1.087864
C	3.399345	2.506741	0.408853
O	4.220551	1.731989	0.913610
N	3.596376	3.138090	-0.769238
C	4.769586	2.905640	-1.591112
C	-6.243684	0.935543	-1.337001
C	-3.729098	1.288481	2.772077
C	-1.236904	2.684162	-2.670605
C	2.006109	2.437338	2.548636
H	-6.008235	0.932261	-2.405301
H	-7.206391	1.444240	-1.217555
H	-6.340704	-0.092050	-0.976780
H	-4.771608	0.123166	0.646260
H	-3.886526	2.864250	1.296875
H	-4.694830	1.560654	3.209146
H	-2.931384	1.730207	3.376311
H	-3.624672	0.201674	2.804568
H	-2.826486	2.925301	-0.713979
H	-0.690951	1.100936	-1.295861
H	-0.416608	2.476682	-3.362819
H	-1.393781	3.766225	-2.662480
H	-2.143277	2.192163	-3.036945
H	0.488340	1.445124	0.732003
H	1.942210	3.958830	1.010531
H	2.873562	2.824333	3.089890
H	2.007494	1.347404	2.624255
H	1.095187	2.820991	3.018964
H	2.807618	3.667240	-1.143002
H	5.651577	2.827923	-0.950648
H	4.687733	1.981913	-2.179649
H	4.897428	3.746617	-2.277010
O	-4.220363	-1.732259	0.914411
C	-3.399294	-2.506657	0.408881
C	-2.054737	-2.871326	1.087378
N	-3.596569	-3.137114	-0.769648
H	-2.807935	-3.666099	-1.143912
C	-4.769937	-2.904063	-1.591122
H	-5.651510	-2.825246	-0.950234
H	-4.687560	-1.980686	-2.180141

```
H  -4.898862   -3.745255   -2.276562
H  -1.942177   -3.959225    1.009520
C  -2.005562   -2.438428    2.548340
H  -1.094536   -2.822322    3.018270
H  -2.872896   -2.825632    3.089632
H  -2.006907   -1.348526    2.624443
N  -0.932945   -2.280585    0.350862
H  -0.488122   -1.445514    0.731894
C  -0.381927   -2.872312   -0.731047
O  -0.855480   -3.881186   -1.269305
C   0.891724   -2.179818   -1.272800
H   0.690918   -1.100486   -1.296131
C   1.236784   -2.683300   -2.671357
H   2.143174   -2.191206   -3.037525
H   0.416497   -2.475575   -3.363507
H   1.393629   -3.765371   -2.663583
N   2.028787   -2.387348   -0.371035
H   2.826343   -2.925118   -0.714852
C   2.260930   -1.593254    0.704111
O   1.445745   -0.765621    1.126003
C   3.654744   -1.795810    1.335077
H   3.886291   -2.864997    1.296089
C   3.728901   -1.289803    2.771902
H   4.694594   -1.562195    3.208919
H   2.931126   -1.731712    3.375920
H   3.624524   -0.203001    2.804839
N   4.660465   -1.128332    0.505987
H   4.771474   -0.123678    0.646497
C   5.180884   -1.726405   -0.596245
O   4.843234   -2.857821   -0.966450
C   6.244590   -0.935632   -1.336045
H   6.335478    0.094338   -0.981000
H   7.208813   -1.439461   -1.208115
H   6.015060   -0.939511   -2.405570
```

-1981.0671016

7a

```
O,6.4446462665,-4.1234475492,-1.0642728681\
C,7.2768713603,-3.2119890464,-0.9755616107\
N,7.2764131057,-2.3188398024,0.0491445664\
C,6.292534278,-2.4051829155,1.1283325614\
C,4.9779277436,-1.7309904851,0.6768950562\
O,4.6751839557,-0.5792750667,1.0116331051\
N,4.2211007075,-2.5004155306,-0.1399655296\
C,3.0238342053,-1.9988429643,-0.815443064\
C,1.7709852629,-2.3323412685,0.0261827687\
O,1.1113775832,-3.3595972953,-0.1705433667\
N,1.4717619213,-1.4324995986,0.9897111188\
C,0.3551000607,-1.632354505,1.9176374477\
C,-0.9643451865,-1.1736473879,1.2506320302\
O,-1.5144899189,-0.1055772717,1.5339400038\
N,-1.4345510995,-2.0450566551,0.323858813\
C,-2.5890305664,-1.762696273,-0.5254971242\
C,8.3871655694,-3.039036772,-1.9954723642\
C,6.8383203236,-1.7936426552,2.4142763208\
C,2.9126620169,-2.5973212618,-2.215815738\
C,0.6153600007,-0.9049501424,3.2326561819\
H,7.9523531834,-3.052178987,-2.9993845502\
H,9.0653209748,-3.8965139077,-1.9242705259\
H,8.9556926916,-2.1177222915,-1.8462354977\
H,7.8705581732,-1.489890086,-0.0042506026\
H,6.0907170232,-3.4710038054,1.2757466808\
H,7.7543288454,-2.3124728851,2.7124463856\
H,6.1037311893,-1.8848757664,3.2199731073\
H,7.0593209487,-0.7312676544,2.2849715624\
H,4.6450772888,-3.3703739438,-0.4664486398\
H,3.1249712775,-0.9096163604,-0.867811907\
H,2.0561106944,-2.1693123993,-2.7459473738\
H,2.7589464367,-3.6782588669,-2.1576685653\
H,3.8208237513,-2.3910902426,-2.790203716\
H,1.9844845409,-0.5541728594,1.0499367631\
H,0.2830370566,-2.7119173213,2.0884808389\
H,-0.2331744928,-1.0312707437,3.9110016632\
H,0.7634606606,0.1637790011,3.0622142186\
H,1.5126866581,-1.3121111029,3.708703878\
H,-0.8423342244,-2.8453936353,0.0995637908\
O,8.8832405028,0.081660208,-0.4954601078\
C,8.8350748019,1.2042954108,-1.0176844519\
N,7.8549338647,2.0960450892,-0.7652506077\
C,6.7225624,1.8323130004,0.1094659808\
C,5.5581741291,2.6910746233,-0.4085878127\
O,5.7741925334,3.7265438611,-1.0457877973\
N,4.3241573837,2.253551457,-0.0813572697\
C,3.1313795803,3.0152832459,-0.4164806524\
C,1.9948666544,2.5193329181,0.4891645932\
O,2.0785808908,1.4372788104,1.0807096152\
N,0.9143824794,3.3207831596,0.5684989046\
C,-0.3223784548,2.9251976389,1.2358330358\
C,-1.4776928524,3.3667048518,0.3270761789\
O,-1.401915872,4.4158187105,-0.3190284686\
N,-2.5573628144,2.5554260178,0.3280835079\
C,-3.7683341673,2.8861141928,0.4058767336\
C,9.9096047579,1.6534936739,-1.9924590584\
C,7.0353734795,2.1789474883,1.5768013151\
C,2.7511266542,2.8736601529,-1.9045073672\
C,-0.4554848956,3.5789893518,2.6217859702\
H,10.8852126016,1.5707876978,-1.5038948902\
H,9.7738220955,2.6783490017,-2.3495194667\
H,9.9166750456,0.9746551179,-2.8513294242\
H,7.8089629633,2.9864296389,-1.249826718\
H,6.4631493253,0.7744454722,0.0430078451\
H,7.9159662211,1.6186741487,1.9049123444\
H,6.1911509497,1.9124874156,2.2198916266\
H,7.2407685972,3.2494303174,1.6840347726\
H,4.1947535608,1.372265359,0.4172634564\
H,3.3323838984,4.0733377532,-0.2107341015\
H,1.8731169364,3.4811978775,-2.1492307586\
H,2.5277363439,1.8286411235,-2.1453120371\
H,3.5897403974,3.2098161162,-2.5188043701\
H,0.8469236375,4.1684208392,0.0144935811\
H,-0.3154936132,1.8399389687,1.3455556269\
H,-1.3757214556,3.2459469254,3.1130215188\
H,-0.4813199385,4.6702561742,2.532630315\
H,0.3952079996,3.2954235653,3.2489116729\
H,-2.5437416344,1.668026989,0.8313991853\
C,-3.8086833605,-2.559544557,-0.0181023383\
O,-3.9793404757,-3.7456071301,-0.3270616216\
N,-4.6499288227,-1.8748156305,0.7888501856\
C,-5.8238501764,-2.5049830777,1.3993621149\
C,-2.2891376052,-2.1141456683,-1.9833243452\
H,-2.7951587794,-0.6945717698,-0.4202732871\
H,-3.1385885042,-1.8515973863,-2.6231319213\
H,-2.1118027358,-3.1882680508,-2.0879154834\
H,-1.4075418848,-1.5674464108,-2.3316383851\
C,-4.8991628394,2.0093423163,0.1525187022\
O,-4.6473670781,1.0324641073,0.8709850295\
N,-6.1486398978,2.3557368679,-0.2049248116\
C,-7.3202084788,1.5434144236,0.1058930361\
C,-3.6042574808,2.6787280705,-1.92575173\
H,-4.001602432,3.942626522,-0.2261158604\
H,-4.511236051,2.9679629542,-2.4674477985\
H,-3.3870253063,1.6287097442,-2.1499262487\
H,-2.775838521,3.2970884694,-2.2786452032\
H,-4.5208114733,-0.8727292711,0.9300946826\
H,-6.323697007,3.1135747129,-0.8577463557\
C,-6.9818046551,-2.53057781,0.3732466402\
O,-7.9216049344,-1.7255742727,0.4152351192\
N,-6.8630256051,-3.4947075424,-0.5644704844\
C,-7.7926948992,-3.611788298,-1.6750241372\
C,-6.2114902942,-1.7826556984,2.6853632532\
H,-5.5443499635,-3.541285168,1.6165049522\
H,-7.0973035452,-2.2460911343,3.128889368\
H,-6.4405356569,-0.7316313184,2.492533375\
H,-5.3876384806,-1.8355273385,3.4034802322\
C,-8.2195496192,1.5680615136,-1.1397529172\
O,-8.2663322373,2.576056115,-1.8485101306\
N,-8.9431919851,0.4436316671,-1.3387473489\
C,-9.8925282921,0.3154500025,-2.4303508539\
C,-8.0791603879,2.0829104391,1.3298623752\
H,-6.9830026715,0.5274268943,0.3122990242\
H,-8.9278812658,1.4334664442,1.5672264026\
H,-8.4539197264,3.0932722496,1.1350027252\
H,-7.4116659591,2.1151127287,2.1965381634\
H,-9.976189401,1.2861833216,-2.9207948639\
H,-9.5556465041,-0.4255919874,-3.1659933126\
H,-10.876048589,0.0122130195,-2.054192736\
H,-8.8145577962,-3.4910296683,-1.3077148392\
H,-7.6111740047,-2.8527189643,-2.447802884\
H,-7.687718072,-4.6023148353,-2.1233995946\
H,-5.9654085956,-3.9779576741,-0.6039345441\
H,-8.7455524719,-0.3693001996,-0.7556682902\
```

-2970.4042845

7b

```
O,-9.3424050707,-3.2974202151,-0.9562645265\
C,-9.1763765053,-2.1078082647,-0.692416303\
N,-7.934478893,-1.5514926322,-0.5788680755\
C,-6.7485258094,-2.3574739743,-0.7839132371\
C,-5.6121414355,-1.8288589901,0.0971496501\
O,-5.6328263977,-0.6756796661,0.5519345734\
N,-4.5908276319,-2.6821298588,0.3095584528\
C,-3.364485371,-2.3183156155,1.0063449675\
C,-2.2185606397,-3.0798253096,0.3231331742\
O,-2.4269081738,-4.1587106432,-0.2382966771\
N,-1.0001372719,-2.5046790625,0.4207535307\
C,0.1929964134,-3.1585663964,-0.0931235892\
C,1.4061397539,-2.5159731484,0.5943365748\
O,1.3049509382,-1.4317902514,1.1784368133\
N,2.5676018395,-3.1938947338,0.4957244539\
C,3.8428386772,-2.6502319477,0.9532504784\
C,-10.3400345526,-1.1614053456,-0.443237719\
C,-6.3192377282,-2.3924416723,-2.2670912225\
C,-3.427389069,-2.6716707166,2.5032656932\
C,0.3014442932,-3.0437977833,-1.6274105148\
H,-10.8205783977,-1.4387160872,0.5020404752\
H,-11.0802402093,-1.3038882767,1.2360953039\
H,-10.0384619088,-0.1121460732,-0.40006264\
H,-7.8243035642,-0.5558988287,-0.4055847283\
H,-7.0036650182,-3.3784489524,-0.4800758979\
H,-7.1466021033,-2.7911752323,-2.8599701677\
H,-5.4415945074,-3.030776415,-2.4199354931\
H,-6.0832523797,-1.3841378977,-2.6249129842\
H,-4.5748776959,-3.6001319354,-0.1236760517\
H,-3.2114458064,-1.2433111768,0.9013685607\
H,-2.51933409,-2.3342532631,3.0125972011\
H,-3.5302850622,-3.7534482679,2.6393645988\
H,-4.2903210752,-2.1789530567,2.9615360855\
H,-0.879284105,-1.5996286108,0.8769080212\
H,0.140770916,-4.2218307767,0.1701042282\
H,1.1839823732,-3.5720601346,-2.0043560011\
H,0.3698277795,-1.9933147781,-1.9300549885\
H,-0.5887320498,-3.487681941,-2.0792383297\
H,2.6317128204,-4.0478489339,-0.0479077095\
O,-8.2720569249,1.5472798387,-0.5582611474\
C,-7.9671485649,2.7400834996,-0.5702677875\
N,-6.6973373712,3.1786377564,-0.7912736095\
C,-5.5703293784,2.2774752874,-1.0162870205\
C,-4.4472956964,2.669264561,-0.0437038043\
O,-3.8226566651,3.7280097431,-0.1888776608\
N,-4.2181347789,1.7963706955,0.962659902\
C,-3.1477167283,2.0185211265,1.9364794933\
C,-1.8374927548,1.4662364765,1.3339869842\
O,-1.4321672491,0.3256590042,1.5796134453\
N,-1.222699519,2.3163962353,0.4770912213\
C,-0.071398818,1.9265045375,-0.3397265593\
C,1.2378203734,2.3796296506,0.3456834776\
O,1.7267954878,3.4926314389,0.1344036815\
N,1.7814533146,1.4736117733,1.1916483255\
C,3.01695198,1.7535497446,1.9262986735\
C,-9.00879338,3.8221495776,-0.3418986397\
C,-5.0705525391,2.3454049775,-2.4639077176\
C,-3.4811834265,1.3741762906,3.277321635\
C,-0.181934715,2.5288084577,-1.7390928543\
H,-9.7961165281,3.7172675577,-1.094600756\
H,-8.6056677055,4.8383021446,-0.38641251\
H,-9.4704828739,3.6642863453,0.6376859009\
H,-6.4969610483,4.169942793,-0.7785670026\
H,-5.9345075981,1.2762310398,-0.7847664338\
H,-5.8837439312,2.0994098911,-3.1534943968\
H,-4.2539370308,1.6311103386,-2.6220125478\
H,-4.693608536,3.3468667434,-2.6928032328\
H,-4.6966164504,0.8924957695,0.9595774416\
H,-3.0523705494,3.1024413334,2.0508135567\
H,-2.682328934,1.569055187,3.9992847668\
H,-3.5841862887,0.2906678332,3.1795831962\
H,-4.4163113828,1.7863121318,3.6681749722\
H,-1.7087889515,3.1807121067,0.2496822321\
H,-0.0820455312,0.8333561678,-0.3866327353\
H,0.6424202605,2.1798014636,-2.3689334406\
H,-0.1198008321,3.6195787978,-1.6900020445\
H,-1.128106327,2.2388926247,-2.2064694837\
H,1.3922220201,0.5347357124,1.2595293456\
C,4.8787174815,-2.9585712993,-0.134439709\
O,4.8456483584,-4.0298978473,-0.7457172245\
N,5.825319796,-2.0083107142,-0.3268266532\
C,6.8787691449,-2.2086440408,-1.3161241824\
C,4.278471554,-3.268996098,2.2922852679\
H,3.726398568,-1.5731729186,1.0740905838\
H,5.2159404333,-2.8165965836,2.632105008\
H,4.4262164224,-4.3493927232,2.1896806074\
H,3.5108860981,-3.0881910016,3.0509156199\
C,4.2290358811,1.4399673062,1.0168322715\
O,4.857809029,0.3764416972,1.113882711\
N,4.4983868169,2.4023484546,0.1121693325\
C,5.5572170045,2.3157643785,-0.8888256268\
C,3.0574450809,0.9653673153,3.2316583884\
H,3.0184842996,2.8282741402,2.1341851636\
H,3.9953864706,1.1552729414,3.7615303075\
H,2.9820926146,-0.1083678907,3.0434853706\
H,2.2230729097,1.267657953,3.8720284254\
H,5.7126234937,-1.0879995661,0.1059506026\
H,3.8882727666,3.2166795237,0.0936138786\
C,8.0930775699,-1.3707563402,-0.9038512753\
O,7.9939535305,-0.1547777217,-0.7302031965\
N,9.2609261442,-2.0486606639,-0.7853505934\
C,10.522307937,-1.4171473243,-0.4315315568\
C,6.4302717161,-1.8255503905,-2.7370787464\
H,7.1196348628,-3.2764303716,-1.3029319609\
H,7.2296455728,-2.0164925824,-3.4625448898\
H,6.1678812467,-0.764625075,-2.7810212946\
H,5.5597886422,-2.4264283712,-3.012609646\
C,6.646016323,3.3531916541,-0.5503569498\
O,6.3636187483,4.5439283048,-0.4466751864\
N,7.8946694753,2.8388682618,-0.407161975\
C,9.0417498397,3.6716840587,-0.0975684127\
C,4.9978101869,2.5920013712,-2.2899016917\
H,5.9661901898,1.3064319391,-0.8317835779\
H,5.7902444229,2.5008698364,-3.0410591977\
H,4.5966180363,3.6090135385,-2.3420853595\
H,4.2011995126,1.8810628037,-2.5328821701\
H,8.7018283297,4.7080744169,-0.0697444507\
H,9.4708187313,3.4103107928,0.8778007064\
H,9.8215743183,3.5685584648,-0.8620148555\
H,10.866412313,-1.7465947838,0.5554827075\
H,11.2924921312,-1.6510060988,-1.1740353513\
H,10.358795518,-0.3396501867,-0.4088038522\
H,9.2415289292,-3.0520827884,-0.8996143817\
H,8.0194525461,1.8336875314,-0.4717283821\
```

-2970.388321

8a

H	9.796743	-0.668182	-0.473371
N	9.846390	-1.661031	-0.699818
C	8.778218	-2.438838	-0.414462
O	8.657600	-3.622275	-0.740699
C	7.686940	-1.748145	0.417428
N	6.428623	-2.405746	0.085779
H	6.529224	-3.360208	-0.247326
C	5.243942	-1.769822	0.065861
O	5.103469	-0.588558	0.411212
C	4.058428	-2.587380	-0.463285
N	2.840479	-2.027508	0.100258
H	2.843324	-1.035842	0.337606
C	1.706471	-2.752832	0.204744
O	1.597334	-3.924210	-0.168985
C	0.532318	-2.029754	0.880622
N	-0.685148	-2.676719	0.408223
H	-0.556207	-3.631934	0.087445
C	-1.856925	-2.032119	0.258930
O	-2.025923	-0.850969	0.589844
C	-2.979164	-2.843590	-0.401338
C	10.994767	-2.161180	-1.434341
C	8.003238	-1.841266	1.920126
C	4.042845	-2.573370	-2.005445
C	0.659024	-2.090788	2.413320
H	10.890561	-3.242747	-1.530389
H	11.049716	-1.720769	-2.437907
H	11.923500	-1.931136	-0.900383
H	7.617872	-0.696833	0.137134
H	4.136456	-3.625581	-0.123694
H	0.519608	-0.983748	0.571272
H	7.188811	-1.390857	2.495355
H	8.928925	-1.302637	2.145936

```
H   8.118501   -2.886308    2.226772
H   3.185510   -3.151309   -2.360062
H   4.957477   -3.018128   -2.412302
H   3.960171   -1.545902   -2.375435
H  -0.211844   -1.612557    2.871069
H   1.559497   -1.562356    2.741337
H   0.713658   -3.129609    2.755925
O   9.170323    1.129051   -0.014906
C   8.958971    2.303971   -0.349503
N   7.788760    2.927564   -0.107268
C   6.640473    2.275935    0.507112
C   5.391666    3.002117   -0.013865
O   5.452318    4.176874   -0.388653
N   4.252371    2.276796    0.012235
C   2.979120    2.843619   -0.401480
C   1.856880    2.032202    0.258850
O   2.025853    0.851033    0.589710
N   0.685151    2.676857    0.408266
C  -0.532309    2.029938    0.880768
C  -1.706484    2.752914    0.204829
O  -1.597411    3.924295   -0.168913
N  -2.840416    2.027485    0.100226
C  -4.058365    2.587291   -0.463379
C  10.022523    3.123548   -1.058648
C   6.701007    2.324665    2.044478
C   2.814263    2.838458   -1.935180
C  -0.658987    2.091167    2.413458
H  10.932003    3.126338   -0.450080
H   9.719030    4.156127   -1.252585
H  10.267055    2.640667   -2.010444
H   7.612254    3.874537   -0.426540
H   6.613155    1.232096    0.191657
H   7.622406    1.845309    2.387395
H   5.848682    1.790088    2.475065
H   6.685768    3.360720    2.399332
H   4.276581    1.283679    0.242763
H   2.947295    3.880695   -0.051062
H   1.863332    3.294928   -2.230734
H   2.842899    1.812896   -2.318270
H   3.630645    3.410375   -2.383610
H   0.556233    3.632088    0.087533
H  -0.519565    0.983894    0.571556
H  -1.559450    1.562768    2.741559
H  -0.713631    3.130031    2.755933
H   0.211892    1.613004    2.871258
H  -2.843221    1.035833    0.337651
H  -2.947281   -3.880648   -0.050875
C  -2.814370   -2.838486   -1.935046
H  -3.630746   -3.410464   -2.383410
H  -1.863434   -3.294932   -2.230623
H  -2.843067   -1.812944   -2.318178
N  -4.252427   -2.276813    0.012400
H  -4.276695   -1.283676    0.242861
C  -5.391687   -3.002189   -0.013730
O  -5.452282   -4.176945   -0.388535
C  -6.640528   -2.276098    0.507268
H  -6.613154   -1.232203    0.192017
C  -6.701243   -2.325154    2.044615
H  -7.622659   -1.845830    2.387525
H  -5.848939   -1.790711    2.475414
H  -6.686085   -3.361286    2.399246
N  -7.788760   -2.927580   -0.107384
H  -7.612283   -3.874527   -0.426740
C  -8.958891   -2.303877   -0.349707
O  -9.170203   -1.128979   -0.014993
C -10.022383   -3.123235   -1.059192
H  -9.718944   -4.155798   -1.253296
H -10.931967   -3.126075   -0.450780
H -10.266714   -2.640123   -2.010923
H  -4.136452    3.625497   -0.123810
C  -4.042707    2.573258   -2.005540
H  -4.957340    3.017963   -2.412448
H  -3.959957    1.545789   -2.375507
H  -3.185380    3.151237   -2.360109
C  -5.243873    1.769707    0.065739
O  -5.103376    0.588475    0.411178
N  -6.428574    2.405598    0.085552
H  -6.529167    3.360074   -0.247512
C  -7.686886    1.748007    0.417251
H  -7.617915    0.696733    0.136795
C  -8.003014    1.840900    1.920004
H  -8.928660    1.302211    2.145844
H  -8.118263    2.885891    2.226827
H  -7.188505    1.390421    2.495060
C  -8.778236    2.438852   -0.414408
O  -8.657600    3.622299   -0.740590
N  -9.846527    1.661146   -0.699610
H  -9.796889    0.668276   -0.473252
C -10.995032    2.161431   -1.433844
H -11.923694    1.930570   -0.900123
H -11.049753    1.721822   -2.437777
H -10.891266    3.243118   -1.529029
```

-2970.4087901

8c

```
O  -7.452423    3.192574   -0.709371
C  -7.883613    2.056501   -0.476012
N  -7.443919    1.302606    0.564108
C  -6.414264    1.793275    1.483100
C  -5.023875    1.550377    0.859029
O  -4.284772    0.620588    1.203069
N  -4.705405    2.432051   -0.120873
C  -3.577835    2.225314   -1.034678
C  -2.253169    2.743952   -0.424349
O  -1.661721    3.725517   -0.891333
N  -1.792731    2.031954    0.626531
C  -0.616521    2.438937    1.400196
C   0.680406    1.953822    0.709035
O   1.409858    1.077463    1.183966
N   0.946926    2.586758   -0.459632
C   2.025863    2.168044   -1.355126
C  -8.977353    1.435207   -1.325777
C  -6.567853    1.142375    2.853664
C  -3.864269    2.898244   -2.374026
C  -0.728043    1.929448    2.832811
H  -8.704951    1.524324   -2.381519
H  -9.901466    2.004625   -1.179147
H  -9.166521    0.386658   -1.080596
H  -7.632607    0.300161    0.587531
H  -6.561840    2.875349    1.557879
H  -7.523472    1.439104    3.296923
H  -5.758415    1.455516    3.519591
H  -6.544199    0.053752    2.769071
H  -5.449353    3.068488   -0.412674
H  -3.468896    1.140891   -1.171274
H  -3.055169    2.698292   -3.081108
H  -3.933926    3.982907   -2.254437
H  -4.803252    2.522300   -2.792038
H  -2.320453    1.216193    0.936472
H  -0.595951    3.535398    1.384607
H   0.164180    2.201132    3.403323
H  -0.826159    0.841463    2.841899
H  -1.605016    2.371057    3.316552
H   0.250456    3.253706   -0.797741
O  -7.183010   -1.603501    0.690607
C  -6.396905   -2.376484    0.130417
C  -5.107619   -2.906144    0.808193
N  -6.588234   -2.863560   -1.115272
H  -5.822777   -3.405721   -1.517648
C  -7.711255   -2.468859   -1.945360
H  -8.603700   -2.370851   -1.322654
H  -7.536416   -1.510794   -2.453396
H  -7.884595   -3.238037   -2.702072
H  -5.062658   -3.983773    0.611573
C  -5.090469   -2.643039    2.309691
H  -4.226438   -3.138964    2.763227
H  -6.002655   -3.027059    2.774138
H  -5.024676   -1.570980    2.509111
N  -3.920115   -2.317658    0.181052
H  -3.452316   -1.549111    0.659277
C  -3.366022   -2.821863   -0.943057
O  -3.880463   -3.732490   -1.604666
C  -2.025573   -2.168732   -1.355907
H  -2.135423   -1.083773   -1.233133
C  -1.672840   -2.505523   -2.801855
H  -0.723027   -2.036875   -3.077540
H  -2.453418   -2.146137   -3.477997
H  -1.593622   -3.587494   -2.938685
N  -0.947061   -2.588185   -0.460182
H  -0.250414   -3.254751   -0.798647
C  -0.680558   -1.955667    0.708716
O  -1.410153   -1.079617    1.184033
C   0.616478   -2.440771    1.399639
```

H	0.596340	-3.537222	1.383365
H	0.727789	-1.932097	2.832563
H	1.604929	-2.373651	3.316044
H	-0.164341	-2.204446	3.402902
H	0.825476	-0.844081	2.842282
N	1.792546	-2.032884	0.626221
H	2.319364	-1.216607	0.936246
C	2.253319	-2.744140	-0.425010
O	1.662464	-3.725927	-0.892273
C	3.577699	-2.224564	-1.035133
N	4.705429	-2.431824	-0.121642
H	5.449432	-3.067925	-0.414005
C	5.023927	-1.550720	0.858774
O	4.284881	-0.621043	1.203231
C	6.414405	-1.793922	1.482518
H	3.468476	-1.140053	-1.170724
C	3.864092	-2.896249	-2.375117
H	4.803065	-2.519931	-2.792812
H	3.054980	-2.695614	-3.082000
H	3.933708	-3.981022	-2.256527
N	7.443828	-1.301885	0.564016
H	7.631733	-0.299280	0.588028
C	7.883425	-2.054490	-0.477130
O	7.452725	-3.190525	-0.711508
C	8.976519	-1.431709	-1.326628
H	8.704071	-1.520373	-2.382395
H	9.901157	-2.000400	-1.180467
H	9.164746	-0.383157	-1.080751
H	6.562318	-2.876028	1.556051
C	6.567903	-1.144603	2.853856
H	7.523939	-1.441086	3.296363
H	5.758946	-1.459257	3.519660
H	6.543284	-0.055890	2.770708
H	2.136107	1.083107	-1.232631
C	1.673540	2.505117	-2.801116
H	2.454438	2.146075	-3.477085
H	1.594114	3.587103	-2.937680
H	0.723943	2.036299	-3.077255
C	3.365806	2.821740	-0.941529
O	3.879358	3.733770	-1.601853
N	3.920416	2.316503	0.181885
H	3.453706	1.546622	0.658999
C	5.107568	2.905120	0.809583
H	5.061733	3.982940	0.614261
C	5.090779	2.640157	2.310769
H	6.003092	3.023510	2.775508
H	5.024850	1.567850	2.508789
H	4.226867	3.135552	2.765113
C	6.397110	2.377178	0.131002
O	7.183767	1.604027	0.690200
N	6.588043	2.866005	-1.114071
H	5.822115	3.408021	-1.515713
C	7.711300	2.473341	-1.944799
H	8.602774	2.371164	-1.321418
H	7.535533	1.517849	-2.457352
H	7.886767	3.245636	-2.697860

-2970.39883257

Model Structures 9: Cartesian coordinates and total energies (in hartrees, HF/3-21G).

9a

H	9.624774509	-3.3433822299	-0.2599916157
C	9.6334918133	-2.5566324889	0.477412907
C	8.2169903641	-2.2841085386	0.924475414
N	8.0576334905	-1.2432848305	1.761793941
O	7.2525110923	-2.9572901263	0.5550542038
H	10.2319167413	-2.8705171852	1.3254207411
H	10.0792853682	-1.6638352453	0.0552314169
H	8.8435408022	-0.7039957777	2.0564411672
C	6.7574834385	-0.9535349962	2.3589870553
H	6.4323043545	-1.7846786897	2.9667301494
C	6.8603263037	0.3265469617	3.2041863031
H	5.9122330349	0.5189943797	3.6891247407
H	7.1153835678	1.1719234626	2.5806333125
H	7.6113824497	0.1987500731	3.9751812285
C	5.6893441085	-0.7869981699	1.2879852535
N	6.0363808377	-0.0970538987	0.1865491118
O	4.5626906416	-1.2524637417	1.4436106474
H	6.8099479061	0.5393446575	0.2268977999
C	5.0506120056	0.1305193527	-0.8651751503
H	4.2479895946	0.7528273242	-0.510581045
C	5.7382716939	0.7825206659	-2.0782806233
H	5.0406268699	0.8546312086	-2.9023310411
H	6.5826179231	0.179926748	-2.3875137968
H	6.0692897097	1.7782706928	-1.8282137212
C	4.4288090402	-1.1702392031	-1.3401086577
N	5.263789212	-2.2035170255	-1.4862065923
O	3.2412601267	-1.2144619485	-1.6653407233
H	6.1690757762	-2.1785309588	-1.0644483032
C	4.7940891266	-3.4764337786	-2.0177725679
H	4.2484084448	-3.3088504881	-2.9317694501
C	5.9985037252	-4.4011757468	-2.259789448
H	5.6582836634	-5.3740206434	-2.5903350211
H	6.5707929589	-4.5087943858	-1.3485046443
H	6.6346404509	-3.9783785895	-3.0281210153
C	3.8205320838	-4.1410478129	-1.0559486571
N	4.1605159572	-4.1093814551	0.2378861872
O	2.7979973265	-4.6890300346	-1.4604746644
H	4.9931567353	-3.6432082184	0.5352709378
C	3.2692646186	-4.6850092189	1.2401115128
H	3.0962581445	-5.7297132267	1.034420021
C	3.886663962	-4.4897889322	2.6332226837
H	3.2170832908	-4.8700031474	3.3938333205
H	4.066208112	-3.4373272739	2.8066693772
H	4.825648968	-5.026706937	2.6995649931
C	1.9033755652	-4.009398028	1.1835844242
N	1.9214299857	-2.6659472997	1.1252918608
O	0.8576849795	-4.646202226	1.2152233423
H	2.7824948522	-2.1539808169	1.1422230515
C	0.6588407036	-1.943749742	1.0863152809
H	0.0371041364	-2.2085899914	1.9273269052
C	0.9486912241	-0.4378362813	1.0584292178
H	0.0388646452	0.1426564014	0.9638901049
H	1.599328643	-0.2287082622	0.2209364555
H	1.4522615143	-0.1496690385	1.9730054339
C	0.139236232	-2.3202137785	-0.1528768233
N	0.5403554172	-2.3935153542	-1.2986546821
O	-1.3586556882	-2.5032910612	-0.0911762335
H	1.5205815282	-2.1869846499	-1.3329018753
C	-0.1326373095	-2.7566967632	-2.5400895089
H	-0.9203961892	-2.0555653665	-2.7702506234
C	0.90541137	-2.8098505679	-3.6732734022
H	0.418278192	-3.0567087293	-4.6078008615
H	1.6445609361	-3.5633209852	-3.4403268869
H	1.4009886833	-1.8517634892	-3.7679906804
C	-0.8153756335	-4.1170048739	-2.4294234481
N	-0.1201481754	-5.0725601352	-1.811194749
O	-1.9266576931	-4.302941094	-2.9254712136
H	0.8053873766	-4.89297499	-1.4715564709
C	-0.6437860448	-6.4263450273	-1.6828084481
H	-0.8388733789	-6.8548671267	-2.6551250024
C	0.3806962518	-7.2763635259	-0.910462134
H	0.0003748667	-8.2822355135	-0.7933636593
H	0.54870839	-6.8286662579	0.0595603357
H	1.3197814929	-7.3059024886	-1.4503418662
C	-1.9730016459	-6.4847580414	-0.9352654871
N	-2.1913886286	-5.4942930304	-0.0499038571
O	-2.7407739253	-7.4170258317	-1.1150320323
H	-1.4901024483	-4.8036095365	0.1146790638
C	-3.4116772236	-5.413683877	0.7261679017
H	-3.8282164244	-6.4072655686	0.8077297421
C	-3.1115981229	-4.835305801	2.118773147
H	-4.0325749994	-4.7277735862	2.6718957219
H	-2.6455785158	-3.8627899284	2.009653187
H	-2.4341626638	-5.4905579718	2.6510921674
C	-4.4880748923	-4.5430464584	0.0775455911
N	-4.2182863905	-4.0373165748	-1.1440012672
O	-5.536607095	-4.3251860654	0.6733559316
H	-3.3914465607	-4.2960048339	-1.6493349724
C	-5.1810201942	-3.1956896925	-1.8107910252
H	-6.1015832544	-3.21946967	-1.2450982067
H	-5.3822629041	-3.544289394	-2.8153808082
C	-4.7548088978	-1.7457753721	-1.9271931039
N	-3.6111179814	-1.3822634199	-1.3616870952
O	-5.4735690717	-0.9309013978	-2.5337061843
H	-3.0191558731	-2.0322360659	-0.8754342681
C	-3.1511574568	-0.0082336714	-1.4744424092
H	-2.3267172259	0.1307666751	-0.7929705498
H	-2.8299558753	0.21170982	-2.479539318
C	-4.2279158632	0.954748913	-1.0122797916
N	-4.63897486	1.9156932324	-1.8476056636
O	-4.7045136654	0.8589465093	0.1259107607
C	-4.4077401148	1.9849297974	-3.3101904108
H	-4.5073416769	1.0018023911	-3.7377447179
H	-3.4286241886	2.3960382362	-3.5219597868
C	-5.6130946916	2.9005106747	-1.3776808349
H	-5.2689433546	3.3900042498	-0.4808974029
C	-5.738646757	3.8680547577	-2.5733811694
H	-6.6929073601	4.373956983	-2.5783269494
H	-4.9473489464	4.6060908919	-2.5209066729
C	-5.521590875	2.9417592902	-3.7859603145
H	-6.4241554065	2.3781224773	-3.9778931601
H	-5.2408807539	3.4783400101	-4.6810971962
C	-6.9688934462	2.2981721919	-1.0397861395
N	-7.292561386	1.1239130924	-1.5909341029
O	-7.7243168013	2.9190504184	-0.2898819916
H	-6.6206167719	0.5654030381	-2.0891160704
C	-8.5602408883	0.4886581413	-1.2456220253
H	-9.345035155	1.2290901754	-1.2767583146
C	-8.8644407181	-0.6485892589	-2.2326366461
H	-9.7369684349	-1.1876209712	-1.8914293175
H	-8.0184732438	-1.3191492106	-2.2965072433
H	-9.056145777	-0.2437052972	-3.2191129749
C	-8.5862533153	-0.0571818232	0.186950442
N	-7.4053719994	-0.0868208095	0.8275020762
O	-9.6448646006	-0.4275030672	0.6722243496
H	-6.5569494871	0.1563296235	0.359394931
C	-7.2722101125	-0.4473571787	2.228616938
H	-8.2501663117	-0.75041885	2.5761301426
C	6.2495878839	-1.57640807	2.4115025353
H	-6.1267634365	-1.7902046957	3.4633479702
H	-5.293832436	-1.2607903484	2.0089722135
H	-6.5521981886	-2.4692966235	1.8857700748
C	-6.8201387607	0.7649483604	3.0465761925
N	-6.9997550864	1.9804143643	2.4794019026
O	-6.3018066265	0.643722585	4.148117105
H	-7.4367646374	2.0931739563	1.5883852919
C	-6.5675875229	3.1616959156	3.1872250612
H	-6.9266033622	4.0308215743	2.6515783514
H	-6.9512465263	3.1873878781	4.1962612513
C	-5.0520457892	3.2754470763	3.3204945472
N	-4.3192984647	2.567760873	2.4396935258
O	-4.5562136273	4.0108786437	4.1698093823
H	-4.7619784673	1.9841307727	1.7553446582
C	-2.9003992989	2.2946048009	2.6830702265
H	-2.6365710936	2.8006475962	3.5954620844
C	-2.6986769272	0.7847449058	2.819840283
H	-1.6679266072	0.5476196302	3.0570245113
H	-2.9758571381	0.2982758851	1.8980216562
H	-3.3427243591	0.423245828	3.6112280507
C	-2.0953557279	2.8567693587	1.5139907636
N	-1.7754614606	4.1595387208	1.54957898
O	-1.7547733727	2.1576133628	0.5483137993
C	-1.972218952	5.127013468	2.6549730566
H	-1.1315990642	5.0631364524	3.3359188502

```
H -2.8908619509     4.9469042987     3.184577311
C -1.0126012805     4.7462910488     0.4364209228
H -1.5649632857     4.6533121723    -0.4809171238
C -0.827953793      6.2281600997     0.8578948372
H  0.1359977228     6.334522017      1.3340646003
H -0.8884206235     6.8959973808     0.0101030315
C -1.9391216013     6.4620876755     1.9018316124
H -1.7207870481     7.2964911656     2.5535118298
H -2.8917211358     6.6293441672     1.4149513766
C  0.339457226      4.0483539255     0.3808529022
N  0.7430879671     3.467637005     -0.7587051118
O  1.0554519313     4.0474066237     1.3814915417
C -0.0900412099     3.2096172259    -1.9539409121
H -0.0807725112     4.0686783943    -2.6118030261
H -1.0922296168     2.9675865115    -1.6474297773
C  1.9793537101     2.668072968     -0.7421784729
H  1.8621104122     1.847734449     -0.061709539
C  2.0766736868     2.1535311796    -2.1928757342
H  2.5678932108     2.9076259149    -2.7915900669
H  2.6001450342     1.2113818422    -2.2480076705
C  0.6025957433     2.0013806448    -2.6101528982
H  0.4762081733     1.9913297041    -3.6840132317
H  0.2019587142     1.0840996492    -2.1972899175
C  3.200690562      3.5055683645    -0.3745118593
N  3.9081636231     3.1511843375     0.7273852873
O  3.5874038175     4.40905439      -1.1134155339
C  3.4088617467     2.3933330106     1.9058950608
H  3.8808549824     1.4223811044     1.9508350687
H  2.3439102171     2.3015432348     1.867521738
C  5.0625541658     4.002730867      1.1087293039
H  4.843584425      5.0222601852     0.8383196328
C  5.2043348251     3.8317528132     2.6320385205
H  5.981520001      3.1154597694     2.8413517106
H  5.4590203771     4.770070166      3.1037726943
C  3.8312346057     3.291884305      3.0763111059
H  3.8783961114     2.7566099868     4.0150768184
H  3.1094793757     4.0936090424     3.1507016203
C  6.2983443404     3.5406434677     0.342934835
N  6.4450678247     4.1291808559    -0.8556601468
O  7.0873928976     2.6843394972     0.7500804702
H  5.6939319747     4.6991767672    -1.194630112
C  7.6930005143     4.0614716548    -1.5841132227
H  8.0138264765     3.033887346     -1.6855126538
C  7.4970170594     4.7086054829    -2.9672418535
H  8.4091031618     4.6754524904    -3.5473245079
H  7.2051855714     5.7449598249    -2.8475055976
H  6.7190690704     4.1887698861    -3.5119571664
C  8.7680696365     4.8290207483    -0.8077726075
N 10.0207090807     4.6538695332    -1.2950977171
O  8.514804953      5.5377637441     0.1435319602
H 10.7747721293     5.1445695012    -0.863778566
H 10.2271891391     4.0432259117    -2.0523372427
```

-5041.6017237

9b

```
H,9.2381571381,4.1511564419,0.1277954844|
C,9.3568181184,3.2768828913,0.491847855|
C,8.0041534316,2.808947857,-0.9625634826|
N,7.9856512417,1.6744316748,-1.6837030035|
O,6.9471310068,3.4052991088,-0.7100539903|
H,9.9799626312,3.5282663641,-1.3428739695|
H,9.8574170124,2.4988407159,0.072461737|
H,8.8351352722,1.1914925275,-1.8920065062|
C,6.7470298779,1.2031744149,-2.290797274|
H,6.3682983689,1.9442803864,-2.9787997218|
C,6.9985180708,-0.1259579333,-3.0232838035|
H,6.0923348905,-0.4380685156,-3.5276260959|
H,7.294623656,-0.8936923323,-2.3236636644|
H,7.7710144274,0.0076259964,-3.7709362694|
C,5.6574644921,1.028154523,-1.2417627868|
N,6.020127991,0.4876082732,-0.0707566022|
O,4.4926290099,1.3620532343,-1.4788249733|
H,6.8526823665,-0.0723097644,-0.0249998545|
C,5.0335704192,0.2707695049,0.9815743781|
H,4.2946597008,-0.4521198135,0.6782969877|
C,5.7551945472,-0.1962511948,2.2617245028|
H,5.0495928228,-0.270798188,3.0787717476|
H,6.5216497353,0.5181955038,2.5312056484|
H,6.2084389012,-1.1661629284,2.1116026362|
C,4.2877294119,1.5570910698,1.3004539141|
N,5.0393006651,2.6561861442,1.3769153438|
O,3.0750626615,1.5531724522,1.5476184211|
H,5.9730188712,2.6446185533,1.0198498619|
C,4.4747578289,3.9480518593,1.7399659489|
H,3.8768381145,3.8459576174,2.6304996584|
C,5.6151022065,4.9583202143,1.9579654899|
H,5.2108114769,5.9408674373,2.164800842|
H,6.2413843092,5.0019918531,1.0780007294|
H,6.2172851706,4.6498026001,2.8031338649|
C,3.5422387776,4.4469828362,0.647303158|
N,3.9770087461,4.3146021496,-0.6071712433|
O,2.4464823096,4.9513711284,0.9136191255|
H,4.881266194,3.9214780797,-0.7972344673|
C,3.1169044475,4.7074280132,-1.7168390818|
H,2.8586903816,5.7525378904,-1.6455689554|
C,3.8374143171,4.4048078227,-3.0403237823|
H,3.2016002707,4.6566227925,-3.8797394374|
H,4.089320624,3.3544018719,-3.0803161983|
H,4.7469537397,4.9890412042,-3.1063736571|
C,1.8027004177,3.9318671548,-1.6618058033|
N,1.9245196466,2.6188878317,-1.436959663|
O,0.7084530834,4.470234563,-1.8409414368|
H,2.8292733234,2.1852240449,-1.3510071111|
C,0.7392913817,1.7821735619,-1.3708040524|
H,0.1558805429,1.866459679,-2.2752141302|
C,1.1815510148,0.332607709,-1.1265829512|
H,0.3420432209,-0.3448308372,-1.0603159678|
H,1.7527851217,0.2922022381,-0.2105162973|
H,1.8176495616,0.010449093,-1.9404155354|
C,-0.1750712954,2.2387409057,-0.2426192312|
N,0.4141613735,2.5341234136,0.912379954|
O,-1.404733885,2.2993882065,-0.3996600314|
H,1.4019495129,2.3882177806,1.0418254334|
C,-0.3582987238,3.0341623801,2.0414560111|
H,-1.1292543096,2.3326761318,2.3223884588|
C,0.5941614964,3.3043258255,3.2187264131|
H,0.0347929861,3.6448388269,4.0808099532|
H,1.3102629394,4.0588589218,2.9288291665|
H,1.1308529842,2.3995043429,3.4728002183|
C,-1.0909341145,4.3219664827,1.6756591652|
N,-0.3871733964,5.2356360919,1.014314844|
O,-2.2704176062,4.4966729276,2.0082836298|
H,0.5878532147,5.0846683458,0.8104618397|
C,-0.9747182656,6.5165822695,0.643260159|
H,-1.2910759291,7.0630048394,1.5200432926|
C,0.0713605541,7.3187830882,-0.1523604266|
H,-0.3403889394,8.2778538242,-0.4393589924|
H,0.3499816942,6.7547514617,-1.0311516548|
H,0.9547621756,7.4743151313,0.4541022204|
C,-2.2248966545,6.357648841,-0.2168298034|
N,-2.2334548181,5.2832665888,-1.0298917333|
O,-3.1312984669,7.1799133742,-0.1888887282|
H,-1.4149717783,4.7117443159,-1.1107478099|
C,-3.370325854,4.9768125602,-1.870202013|
H,-3.7728391635,5.8878855142,-2.290011178|
C,-2.926696875,4.0142632579,-2.9870764055|
H,-3.7797635714,3.7428743035,-3.5924987512|
H,-2.5008076148,3.127452799,-2.5360249168|
H,-2.1705955442,4.4880749873,-3.600352312|
C,-4.5260791573,4.3072774388,-1.1212684864|
N,-4.3147290922,4.0139666069,0.1700932895|
O,-5.5700786886,4.0382636099,-1.7171882313|
H,-3.4885400553,4.3172797505,0.657968003|
C,-5.2954800769,3.3126014143,0.9544305484|
H,-6.2051334111,3.2262612449,0.3784763162|
H,-5.5101105764,3.8406099132,1.8746423516|
C,-4.8708004319,1.9105777132,1.34799336|
N,-3.7215931596,1.4582288044,0.8820157446|
O,-5.6173483054,1.2281177559,2.0782326654|
H,-3.0858317282,2.0239255948,0.3420920036|
C,-3.25412603,0.1271602268,1.2290064978|
H,-2.4537765438,-0.138996385,0.5583839853|
H,-2.8839183757,0.0986609534,2.239543215|
C,-4.356387905,-0.8833841709,1.0097345886|
N,-4.6687607656,-1.7128124643,2.0070809755|
O,-4.9929426115,-0.9174703636,-0.0579092038|
C,-4.1370914523,-1.7098141048,3.3857321357|
H,-4.1582970327,-0.7103304716,3.7905111023|
```

H,-3.1290815692,-2.1015631691,3.4021887531|
C,-5.6100507442,-2.7955583164,1.7593679019|
H,-5.3398880204,-3.3360526684,0.8670836394|
C,-5.4987894782,-3.6700453272,3.0279514056|
H,-6.4167866666,-4.2025373288,3.2304800902|
H,-4.6971931953,-4.3848832431,2.893233051|
C,-5.1152741598,-2.6499362173,4.1176944046|
H,-5.9919772037,-2.091691416,4.4208634059|
H,-4.6649453681,-3.1091491786,4.9859754057|
C,-7.0287340337,-2.2974571946,1.5636834412|
N,-7.3215165503,-1.0546967071,1.9671638491|
O,-7.8675281728,-3.0327205502,1.0320369678|
H,-6.5983341843,-0.4048136625,2.2343261253|
C,-8.6319829293,-0.5015419354,1.6656334925|
H,-9.393581507,-1.2174375447,1.9344822701|
C,-8.830883561,0.8140148692,2.4365577819|
H,-9.7473105089,1.2857727514,2.1068229828|
H,-7.986266863,1.4640678651,2.2591256428|
H,-8.8900613752,0.6132744561,3.5002292603|
C,-8.8427190759,-0.243027044,0.1676704274|
N,-7.7376686649,-0.2493668842,-0.5861508093|
O,-9.9769125857,-0.0283584903,-0.2549533762|
H,-6.8276619665,-0.3630567875,-0.1759128217|
C,-7.7566195701,-0.1242748547,-2.0318669268|
H,-8.7321589456,0.2288349295,-2.3288600513|
C,-6.6499079228,0.8455506614,-2.4972392929|
H,-6.6181161377,0.860348412,-3.5774094146|
H,-5.6957249543,0.4994474512,-2.1200143654|
H,-6.8077570716,1.848291212,-2.1249993841|
C,-7.5078272092,-1.4653633071,-2.732480647|
N,-7.2839666128,-2.5367686443,-1.9407584681|
O,-7.4805052164,-1.5220444862,-3.9558682445|
H,-7.4765098235,-2.5001287263,-0.9615673399|
C,-6.8434187284,-3.7964520741,-2.4979980563|
H,-7.2763379237,-4.618014566,-1.9435331667|
H,-7.163899407,-3.8625706017,-3.5269004208|
C,-5.3259952468,-4.0097161608,-2.5139888386|
N,-4.569276493,-3.0454507324,-1.977069903|
O,-4.8768290211,-5.034031657,-3.0240346422|
H,-4.9654207999,-2.2679423968,-1.4829992586|
C,-3.1147778527,-3.0485259984,-2.1371289524|
H,-2.8800549005,-3.7702233375,-2.8948989277|
C,-2.6391065845,-1.6505233489,-2.5615501517|
H,-1.5570513709,-1.6246767955,-2.6009692713|
H,-2.9871657559,-0.9212307327,-1.8453248971|
H,-3.0418542881,-1.4098503916,-3.5374104652|
C,-2.4208588393,-3.3932843131,-0.8225897509|
N,-1.7024356424,-4.5229784974,-0.773765383|
O,-2.4847611788,-2.6360930966,0.1493449661|
C,-1.6484719443,-5.611100487,-1.7709771417|
H,-0.9225215257,-5.3807437268,-2.5416033294|
H,-2.6217126214,-5.7725206045,-2.207134615|
C,-0.7359061473,-4.7070731551,0.3041856279|
H,-1.2249196681,-4.6190474461,1.2588768823|
C,-0.1509145219,-6.1296129355,0.0496759221|
H,0.8098201169,-6.050363891,-0.44165169|
H,-0.0186077751,-6.6817665119,0.9684520948|
C,-1.1667774765,-6.7889430174,-0.9080924899|
H,-0.7198232073,-7.5774081406,-1.4967366902|
H,-2.0028982644,-7.1901350758,-0.3491010943|
C,0.3774888165,-3.6671117028,0.1533832951|
N,1.2503269379,-3.5596332795,1.1691153679|
O,0.4801569304,-3.0117968659,-0.882934026|
C,1.1320325424,-4.04754654,2.5590161396|
H,1.9626783581,-4.7040700279,2.7651384214|
H,0.198348013,-4.5542427606,2.7344546214|
C,2.3486367669,-2.5721463564,1.1309034245|
H,2.1322216439,-1.8408939521,0.3796796051|
C,2.3793774674,-1.9736540128,2.5730197568|
H,3.3369613654,-2.1799297151,3.0214219894|
H,2.2152494893,-0.9055749821,2.5450056279|
C,1.2774547232,-2.7364698545,3.3529876419|
H,1.557021021,-2.9085299465,4.3826148358|
H,0.3416592118,-2.1915948025,3.3177565463|
C,3.6606428722,-3.3135680207,0.8671631617|
N,4.2659872925,-3.1646541422,-0.3232209015|
O,4.1521781727,-4.0054960739,1.7585577831|
C,3.6853929837,-2.6504655613,-1.5876480088|
H,4.0179472016,-1.6352622815,-1.7638489059|
H,2.6100832518,-2.6904679858,-1.5603850057|
C,5.5546192431,-3.8607030957,-0.5590572114|
H,5.5049200853,-4.8565338381,-0.1552128715|
C,5.7133348199,-3.863194949,-2.0929297167|
H,6.3647825295,-3.0546155452,-2.3791225383|
H,6.1474410622,-4.791321062,-2.4306067443|
C,4.2891285286,-3.612163242,-2.6273273274|
H,4.2916124416,-3.1899090353,-3.6230694085|
H,3.7195882629,-4.5336079438,-2.636493191|
C,6.693430437,-3.0978614191,0.1148278068|
N,7.0819837095,-3.5728292899,1.3212963635|
O,7.2166531159,-2.1082414562,-0.3945338819|
H,6.5112880244,-4.2826949465,1.7347402279|
C,8.5130381807,-3.6086105678,1.6236696262|
H,8.9235234018,-2.6116192384,1.5647340823|
C,8.7308452676,-4.2119966876,3.0186174992|
H,9.7773770596,-4.2139874096,3.2896728585|
H,8.3722697441,-5.2339816609,3.0503394816|
H,8.194870135,-3.6287808167,3.7557590355|
C,9.1530512811,-4.4771324431,0.5178624655|
N,10.4669779067,-4.6928097476,0.626702177|
O,8.4603070044,-4.9017717383,-0.3990123663|
H,10.9245856837,-5.2555781388,-0.0615714339|
H,11.0265120503,-4.3365076114,1.369707811

-5041.6576877
